# Supplementary material for: Prevention of maternal and neonatal death/infections with a single oral dose of azithromycin in women in labour in low-income and middle-income countries (A-PLUS): a study protocol for a multinational, randomised placebo-controlled clinical trial
Source: BMJ Open. 2023 Aug 30;13(8):e068487. doi: 10.1136/bmjopen-2022-068487 (PMC10471878; doi:10.1136/bmjopen-2022-068487)
Supplement: Author's manuscript [file bmjopen-2022-068487.draft_revisions.pdf]

BMJ Open

BMJ Open is committed to open peer review. As part of this commitment we make the peer review history of every article we publish publicly available.

When an article is published we post the peer reviewers' comments and the authors' responses online. We also post the versions of the paper that were used during peer review. These are the versions that the peer review comments apply to.

The versions of the paper that follow are the versions that were submitted during the peer review process. They are not the versions of record or the final published versions. They should not be cited or distributed as the published version of this manuscript.

BMJ Open is an open access journal and the full, final, typeset and author-corrected version of record of the manuscript is available on our site with no access controls, subscription charges or pay-per-view fees (http://bmjopen.bmj.com).

If you have any questions on BMJ Open's open peer review process please email info.bmjopen@bmj.com

BMJ Open

Prevention of maternal and neonatal death/infections with a single oral dose of azithromycin in women in labor in low- and middle-income countries (A-PLUS): Study protocol for a multi-national, randomized placebo-controlled clinical trial

Journal:	*BMJ Open*
Manuscript ID	bmjopen-2022-068487
Article Type:	Protocol
Date Submitted by the Author:	19-Sep-2022

Complete List of Authors:	Hemingway-Foday, Jennifer; RTI International<br>Tita, Alan; The University of Alabama at Birmingham School of Medicine<br>Chomba, Elwyn; University of Zambia University Teaching Hospital<br>Mwenechanya, Musaku; University of Zambia University Teaching Hospital<br>Mweemba, Trecious; University of Zambia University Teaching Hospital<br>Nolen, Tracy; RTI International<br>Lokangaka, Adrien; University of Kinshasa<br>Tshefu Kitoto, Antoinette; University of Kinshasa<br>Lomendje, Gustave; Kinshasa School of Public Health<br>Hibberd, Patricia; Boston University School of Public Health<br>Patel, Archana; Lata Medical Research Foundation; Datta Meghe Institute of Medical Sciences - Wardha Campus<br>Das, Prabir Kumar; Lata Medical Research Foundation, Nagpur<br>Kurhe, Kunal; Lata Medical Research Foundation<br>Goudar, Shivaprasad S.; KLE Academy of Higher Education and Research<br>Kavi, Avinash; KLE Academy of Higher Education and Research<br>Metgud, Mrityunjay; KLE Academy of Higher Education and Research<br>Saleem, Sarah; The Aga Khan University, Community Health Sciences<br>Tikmani, Shiyam; The Aga Khan University, Community Health Sciences<br>Esamai, Fabian; Moi University School of Medicine<br>Nyongesa, Paul; Moi University School of Medicine, Reproductive Health<br>Sagwe, Amos; Moi University School of Medicine<br>Figueroa, Lester; Institute of Nutrition of Central America and Panama<br>Mazariegos, Manolo; Instituto de Nutricion de Centroamerica y Panama<br>Billah, Sk Masum; International Centre for Diarrhoeal Disease Research, Maternal and Child Health Division; The University of Sydney<br>Haque, Rashidul; International Centre for Diarrhoeal Disease Research Bangladesh, Centre for Communicable Diseases<br>Shahjahan Siraj, Md; International Centre for Diarrhoeal Disease Research Bangladesh<br>Goldenberg, R; Columbia University<br>Bauserman, Melissa; University of North Carolina at Chapel Hill School of Medicine<br>Bose, Carl; The University of North Carolina at Chapel Hill School of Medicine<br>Liechty, Edward A; Indiana University School of Medicine<br>Ekhaguere, Osayame; Indiana University Department of Pediatrics,

Neonatal-Perinatal Medicine
Krebs, Nancy F; University of Colorado Denver
Derman, Richard ; Thomas Jefferson University, Office of Global Affairs
Petri, William A.; University of Virginia
Koso-Thomas, Marion; Eunice Kennedy Shriver National Institute of Child Health and Human Development
McClure, Elizabeth; RTI International
Carlo, Waldemar; The University of Alabama at Birmingham, Pediatrics

Keywords: Maternal medicine < OBSTETRICS, NEONATOLOGY, Protocols & guidelines < HEALTH SERVICES ADMINISTRATION & MANAGEMENT, OBSTETRICS, PUBLIC HEALTH, Clinical trials < THERAPEUTICS

Prevention of maternal and neonatal death/infections with a single oral dose of azithromycin in women in labor in low- and middle-income countries (A-PLUS): Study protocol for a multi-national, randomized placebo-controlled clinical trial

Jennifer J Hemingway-Foday[1], Alan Tita[2], Elwyn Chomba[3], Musaku Mwenchanya[3], Trecious Mweemba[3], Tracy L Nolen[1], Adrien Lokangaka[4], Antoinette Tshefu[4], Gustave Lomendje[4], Patricia L Hibberd[5], Archana Patel[6,7], Prabir Das[6], Kunal Kurhe[6], Shivaprasad S. Goudar[8], Avinash Kavi[8], Mrityunjay Metgud[8], Sarah Saleem[9], Shiyam Sunder Tikmani[9], Fabian Esamai[10], Paul Nyongesa[10], Amos Sagwe[10], Lester Figueroa[11], Manolo Mazariegos[11], Sk Masum Billah[12,13], Rashidul Haque[12], Md Shahjahan Siraj[12], Robert L Goldenberg[14], Melissa Bauserman[15], Carl L Bose[15], Edward A Liechty[16], Osayame Austine Ekhaguere[16], Nancy F. Krebs[17], Richard J Derman[18], William A Petri Jr[19], Marion Koso-Thomas[20], Elizabeth M McClure[1], Waldemar A Carlo[2]

[1]RTI International, Durham, NC, USA
[2]University of Alabama at Birmingham, Birmingham, AL, USA
[3]University Teaching Hospital, Lusaka, Zambia
[4]Kinshasa School of Public Health, Kinshasa, Democratic Republic of Congo
[5]Boston University School of Public Health, Boston, MA, USA
[6]Lata Medical Research Foundation, Nagpur, India
[7]Datta Meghe Institute of Medical Sciences, Wardha, India
[8]KLE Academy Higher Education and Research, J N Medical College Belagavi, Karnataka, India
[9]Aga Khan University, Karachi, Pakistan
[10]Moi University School of Medicine, Eldoret, Kenya
[11]Instituto de Nutrición de Centroamérica y Panamá, Guatemala City, Guatemala
[12]International Centre for Diarrhoeal Disease Research, Dhaka, Bangladesh
[13]University of Sydney, Sydney, NSW, Australia
[14]Columbia University School of Medicine, New York, NY, USA
[15]University of North Carolina at Chapel Hill, Chapel Hill, NC, USA
[16]Indiana School of Medicine, University of Indiana, Indianapolis, IN, USA
[17]University of Colorado School of Medicine, Denver, CO, USA
[18]Thomas Jefferson University, Philadelphia, PA, USA
[19]University of Virginia, Charlottesville, VA, USA
[20]*Eunice Kennedy Shriver* National Institute of Child Health and Human Development, Bethesda, MD,USA

Corresponding Author:
Jennifer J. Hemingway-Foday, MPH, MSW
RTI International
3040 E. Cornwallis Road
Research Triangle Park, NC, 27709
Telephone: (919)-485-2663
Email: hemingway@rti.org

For peer review only - http://bmjopen.bmj.com/site/about/guidelines.xhtml

Word count (excluding title page, abstract, references, figures and tables): 3910

ABSTRACT

Introduction

Maternal and neonatal infections are among the most frequent causes of maternal and neonatal mortality, and current antibiotic strategies have been ineffective in preventing many of these deaths. A randomized clinical trial conducted in a single site in The Gambia showed that treatment with oral dose of 2 g azithromycin vs. placebo for all women in labor reduced certain maternal and neonatal infections. However, it is unknown if this therapy reduces maternal and neonatal sepsis and mortality. In a large, multi-national randomized trial, we will evaluate the impact of azithromycin given in labor to improve maternal and newborn outcomes.

Methods and Analysis

This randomized, placebo-controlled, multicenter clinical trial includes two primary hypotheses, one maternal and one neonatal. The maternal hypothesis is to test whether a single, prophylactic intrapartum oral dose of 2 g azithromycin given to women in labor will reduce maternal death or sepsis. The neonatal hypothesis will test whether this intervention will reduce intrapartum/neonatal death or sepsis. The intervention is a single, prophylactic intrapartum oral dose of 2 g azithromycin, compared with a single intrapartum oral dose of an identical appearing placebo. A total of 34,000 laboring women from eight research sites in sub-Saharan Africa, South Asia, and Latin America will be randomized with one-to-one ratio to intervention/placebo. In addition, we will assess antimicrobial resistance in a sample of women and their newborns.

Ethics and Dissemination

The study protocol (Version 1.6, Dated 15 July 2022) has been reviewed and ethics approval obtained from all the relevant ethical review boards at each research site.

Trial Registration

ClinicalTrials.gov number: NCT03871491

https://clinicaltrials.gov/ct2/show/NCT03871491?term=NCT03871491&draw=2&rank=1

Keywords: sepsis, azithromycin, low and middle-income countries; neonatal mortality; maternal mortality

For peer review only - http://bmjopen.bmj.com/site/about/guidelines.xhtml

ARTICLE SUMMARY

Strengths and limitations of this study

- This study evaluates the use of routine prophylactic azithromycin in women who undergo labor and attempt a vaginal delivery, including those at high risk of infection due to prolonged labor or membrane rupture, which was identified as a top research priority by the World Health Organization (WHO) and other stakeholders.
- The study evaluates a simple, inexpensive intervention that can be scaled up to reduce the burden of both maternal and neonatal mortality due to infections if successful.
- To optimize generalizability, this study will be carried out in a racially, ethnically, and geographically diverse population in seven low-and-middle-income countries.
- In response to global public health concerns about increasing antimicrobial resistance, this study includes an ancillary study to assess antimicrobial resistance and microbiome changes.
- In some locations, azithromycin and other antibiotics are increasingly being used, especially with increasing use of cesarean section, and thus the impact of the outcome may be diminished.

For peer review only

INTRODUCTION

Background

Maternal infection during pregnancy and the puerperium, accounting for 10% of maternal deaths, is a top cause of maternal mortality worldwide.[1] Maternal infections increase neonatal sepsis, a leading cause of neonatal death in low-income countries (LICs).[2-5] Neonatal infection accounts for 15% of neonatal mortality worldwide.[2] According to the World Health Organization (WHO), maternal and neonatal deaths from infections have remained unchanged or increased, whereas other causes of death have declined. [5,6]

Innovative, effective, and scalable interventions are needed to reduce infection-related maternal and neonatal mortality. The evidence backing current WHO guidelines to prevent and treat peripartum infections is limited.[7] Further, current approaches to address neonatal sepsis have had limited impact and alternative antibiotic regimens suggesting comparable effectiveness have not reduced newborn deaths.[8-11]

Risk Factors for Maternal and Neonatal Infection and Sepsis

Cesarean delivery (CD), especially after labor or membrane rupture, is an important risk factor for maternal peripartum infection.[12] Antibiotic prophylaxis for CD is a well-established, effective strategy to reduce infection.[13-16] However, with a prevalence of <5% in LICs, CDs do not account for most maternal sepsis worldwide.[17] Many peripartum infections in LMICs occur in women who delivery vaginally, with women undergoing prolonged labor or membrane rupture at highest risk. Identification and management of prolonged labor and prolonged rupture of membranes are critical to reduce maternal sepsis.[7]

Maternal sepsis also increases risk for neonatal sepsis.[5] Although most pregnancies are not at high-risk for infection, they account for about 50% of maternal and neonatal infections in LMICs.[1] Thus, the WHO has identified evaluation of routine prophylactic antibiotics for women undergoing vaginal birth or with prolonged labor/membrane rupture as a top research priority.[1]

Intrapartum Azithromycin to Prevent Maternal and Neonatal Infections

For peer review only - http://bmjopen.bmj.com/site/about/guidelines.xhtml

A novel approach to prevent maternal and neonatal infection is to target organisms that may be frequent pathogens but are not historically targeted for treatment.[16, 18-21] Azithromycin, which is available as a generic agent, has a bimodal half-life of 70 hours in the non-pregnant population. It is commonly used during pregnancy to treat chlamydia, gonorrhea, and other infections.[22] It provides broad coverage against most common pathogens associated with peripartum infections including gram-positive cocci, genital mycoplasmas and *Ureaplasma* infections, and certain gram-negative Bacilli and anaerobes associated with maternal polymicrobial infections and sepsis.[23] In addition, azithromycin also has activity against Group B *streptococcus* (GBS), a major cause of neonatal sepsis in developed countries and possibly in LICs.[24,25]

Evidence from Prior Studies

In a U.S. randomized clinical trial (RCT), a 50% reduction in maternal peripartum infection resulted from 500 mg intravenous azithromycin added to the standard prophylactic regimen in high-risk women undergoing CD following labor or membrane rupture for ≥4 hours.[18] These results were observed despite universal antibiotics in both treatment groups. Readmission, unscheduled visits, serious adverse events, postpartum fever, or subsequent treatment with antibiotics were also reduced in the azithromycin group. A related cost-analysis estimated that adjunctive azithromycin saved $360 USD per use in high-risk CDs and $143 USD per use in scheduled CDs.[26]

A trial in The Gambia suggested the potential for azithromycin prophylaxis to improve maternal and neonatal outcomes.[19] Among 829 participants randomized to 2 grams (g) of azithromycin vs. placebo before delivery, maternal infections were lower in the azithromycin group, 3.6% vs 9.2%, respectively (RR 0.40, 95% CI, 0.22-0.71, P=0.002). Among newborns, infections were also lower in the azithromycin group (18.1% vs 23.8%; RR 0.76 95% CI, 0.58-0.99; P=0.052). Maternal and neonatal carriage of infectious organisms was lower in the azithromycin group.

Rationale for a trial

Drawing from these findings, the role of a single oral dose of azithromycin (plus usual care) to prevent maternal death or peripartum sepsis and intrapartum/neonatal death or sepsis appears promising. Of interest is the sub-population at the highest risk for infection due to prolonged labor and/or prolonged membrane rupture. Considering the success with a single dose of 2g in The Gambian trial and with 500 mg intravenous for cesarean prophylaxis, as well as the 40% bioavailability of oral azithromycin, 2g seems the appropriate dose. A RCT is the best approach to influence future uptake. Such a RCT in a racially, ethnically, and geographically diverse population could be accomplished through the established infrastructure of the Global Network for Women's and Children's Health Research (Global Network), a multi-country research network with sites in India, Pakistan, Bangladesh, Guatemala, Kenya, Zambia, and the Democratic Republic of Congo (DRC) and an established data coordinating center (DCC).[27]

METHODS AND ANALYSIS

Study Design and Intervention

This study, the Prevention of Maternal and Neonatal Death/Infections with a Single Oral Dose of Azithromycin in Women in Labor in Low- and Middle-Income Countries (A-PLUS), is a masked, placebo-controlled, multicenter, RCT. The intervention is a single 2g dose of oral azithromycin or identical placebo, administered as four 500 mg pills following randomization. Both groups will also receive the local standard of care during labor, delivery, and postpartum.

For peer review only - http://bmjopen.bmj.com/site/about/guidelines.xhtml

Primary Hypothesis
The A-PLUS trial has both a maternal and neonatal primary hypothesis:

- Maternal: A single, prophylactic intrapartum oral dose of 2g azithromycin given to women in labor will reduce *maternal* death or sepsis.
- Neonatal: A single, prophylactic intrapartum oral dose of 2g azithromycin given to women in labor will reduce *intrapartum/neonatal* death or sepsis.

Study Population
Pregnant women laboring in health facilities at eight Global Network sites will be screened by research staff per the eligibility criteria (**Table 1**). The secondary population is a cohort of 5,500 high-risk women, defined as term and preterm (≥28 weeks) pregnant women with prolonged labor (≥18 hours) or prolonged membrane rupture (≥8 hours).

Table 1. Eligibility Criteria

**Inclusion Criteria**
• Pregnant women in labor with gestational age ≥ 28 weeks (by best estimate) who plan to deliver vaginally in a facility
• Admission to health facility with clear plan for spontaneous or induced vaginal delivery
• Presence of one or more live fetus confirmed via a fetal heart rate by Doptone prior to randomization
• Age ≥ 18 years of age (minors 14-17 years eligible in countries where married or pregnant minors or their authorized representatives are legally permitted to give consent)
• Provision of written informed consent
**Exclusion Criteria**
• Non-emancipated minors (as per local regulations)
• Evidence of chorioamnionitis or other infection requiring antibiotic therapy at time of eligibility *[women given single prophylactic antibiotics with no plans to continue after delivery will not be excluded]*
• Arrhythmia or known history of cardiomyopathy
• Allergy to azithromycin or other macrolides that is self-reported or documented in the medical record.
• Any use of azithromycin, erythromycin, or other macrolide in the 3 days or less prior to randomization.
• Plan for Cesarean delivery prior to enrollment
• Preterm labor undergoing management with no immediate plan to proceed to delivery
• Advanced stage of labor (>6 cm or 10 cm cervical dilation per local standards) and pushing or too distressed to understand, confirm, or give informed consent regardless of cervical dilation
• Not capable of giving consent due to other health problems such as obstetric emergencies (for example, antepartum hemorrhage) or mental disorder
• Any medical condition considered a contraindication per the judgement of site investigators
• Previous randomization in the trial

Primary Outcomes
The primary outcomes are:

- Maternal: Incidence of maternal death or sepsis within 42 days post-delivery in the intervention vs. placebo group.
- Neonatal: Incidence of intrapartum/neonatal death or sepsis with 28 days post-delivery in the intervention vs. placebo group.

For peer review only - http://bmjopen.bmj.com/site/about/guidelines.xhtml

We use the 2017 WHO definition of sepsis which includes a suspicion of infection and the presence of organ dysfunction based on clinical findings.[7]

Maternal sepsis is defined as organ dysfunction resulting from suspected or confirmed infection that occurs post-randomization during labor or the postpartum period. This WHO definition is operationalized as suspected or confirmed infection based on the presence of fever or hypothermia plus one or more signs of mild to moderate organ dysfunction including tachycardia, low blood pressure, tachypnea, altered mental status/confusion, reduced urinary output, jaundice, or renal failure.[7, 28-30] Components of peripartum infection considered in diagnosing suspected or confirmed infection include clinical chorioamnionitis, endometritis, wound infections (perineal or cesarean), abdominal or pelvic abscess, mastitis/breast abscess or infection, pyelonephritis, pneumonia, and other bacterial infections (**Table 2).**

Table 2. Specified Infections Considered for Maternal Sepsis Diagnosis

Type of Infection	A-PLUS Trial Definition
Chorioamnionitis	Fever (>100.4°F/38°C) in addition to one or more of the following: fetal tachycardia ≥160 bpm, maternal tachycardia >100 bpm, tender uterus between contractions, or purulent/foul smelling discharge from uterus **prior** to delivery.
Endometritis	Fever (>100.4°F/38°C) in addition to one or more of maternal tachycardia >100 bpm, tender uterine fundus, or purulent/foul smelling discharge from uterus **after** delivery.
Wound infection	Purulent infection (superficial or deep infection including necrotizing fasciitis) of a perineal or Cesarean wound with or without fever. In the absence of purulence, a wound infection requires presence of fever (>100.4°F/38°C) and at least one of the following signs of local infection: pain or tenderness, swelling, heat, or redness around the incision/laceration
Abdominopelvic abscess	Evidence of pus in the abdomen or pelvis noted during open surgery, interventional aspiration or imaging
pneumonia	Fever (>100.4°F/38°C) and clinical symptoms suggestive of lung infection including cough and/or tachypnea (>24 breaths/min) or radiological confirmation
pyelonephritis	Fever (>100.4°F/38°C) and one or more of the following: urinalysis/dip suggestive of infection, costovertebral angle tenderness, or confirmatory urine culture
mastitis/breast abscess or infection	Fever (>100.4°F/38°C) and one or more of the following: breast pain, swelling, warmth, redness, or purulent drainage

Neonatal sepsis is defined as proven or possible serious bacterial infection (pSBI) or pneumonia, or meningitis. PSBI will be determined using WHO criteria of severe chest in-drawing, fever, hypothermia, no movement at all or movement only on stimulation, feeding poorly or not feeding at all, and/or convulsions.[31] Clinical and laboratory signs of infection will also be considered for diagnosis of suspected or confirmed infection.

Centralized adjudication of key infection outcomes will be implemented to standardize the results. In addition to these criteria, reported antibiotic treatment and culture status will also be considered in the adjudication process for maternal and neonatal sepsis.

The individual components of these primary outcomes will be analyzed.

Secondary maternal outcomes

- The primary maternal outcome in a high-risk for infection population
- Incidence of specific maternal infections (Table 2)

For peer review only - http://bmjopen.bmj.com/site/about/guidelines.xhtml

- Subsequent maternal antibiotic therapy after randomization to 42 days postpartum for any reason
- Time from drug administration until initial discharge after delivery
- Maternal readmissions and admissions to special care units within 42 days postpartum
- Maternal unscheduled visit for care
- Maternal gastrointestinal symptoms (nausea, vomiting, diarrhea) or other reported side effects
- Maternal death due to sepsis using the Global Network algorithm for cause of death[32]

Secondary Neonatal Outcomes

- Neonatal deaths due to sepsis and all-cause neonatal death in all participants and in laboring women at high risk of infection
- Other neonatal infections (e.g., eye infection, skin infection, omphalitis, urinary tract infection, respiratory rate ≥60 breaths/minute).
- Neonatal readmissions and admissions to special care units within 42 days postpartum
- Neonatal initial hospital length of stay, defined as time of delivery until initial discharge
- Neonatal unscheduled visit for care
- Neonatal death due to sepsis using the Global Network algorithm for cause of death[33]
- Incidence of pyloric stenosis within 42 days of delivery

Study Procedures

All sites will train research staff in standardized study procedures to administer the intervention and assess outcomes. The sequence of study activities is described (**Figure 1)**. Study procedures are also provided **(Table 4)**.

For peer review only - http://bmjopen.bmj.com/site/about/guidelines.xhtml

For peer review only

Figure 1. Sequence of Study Procedures

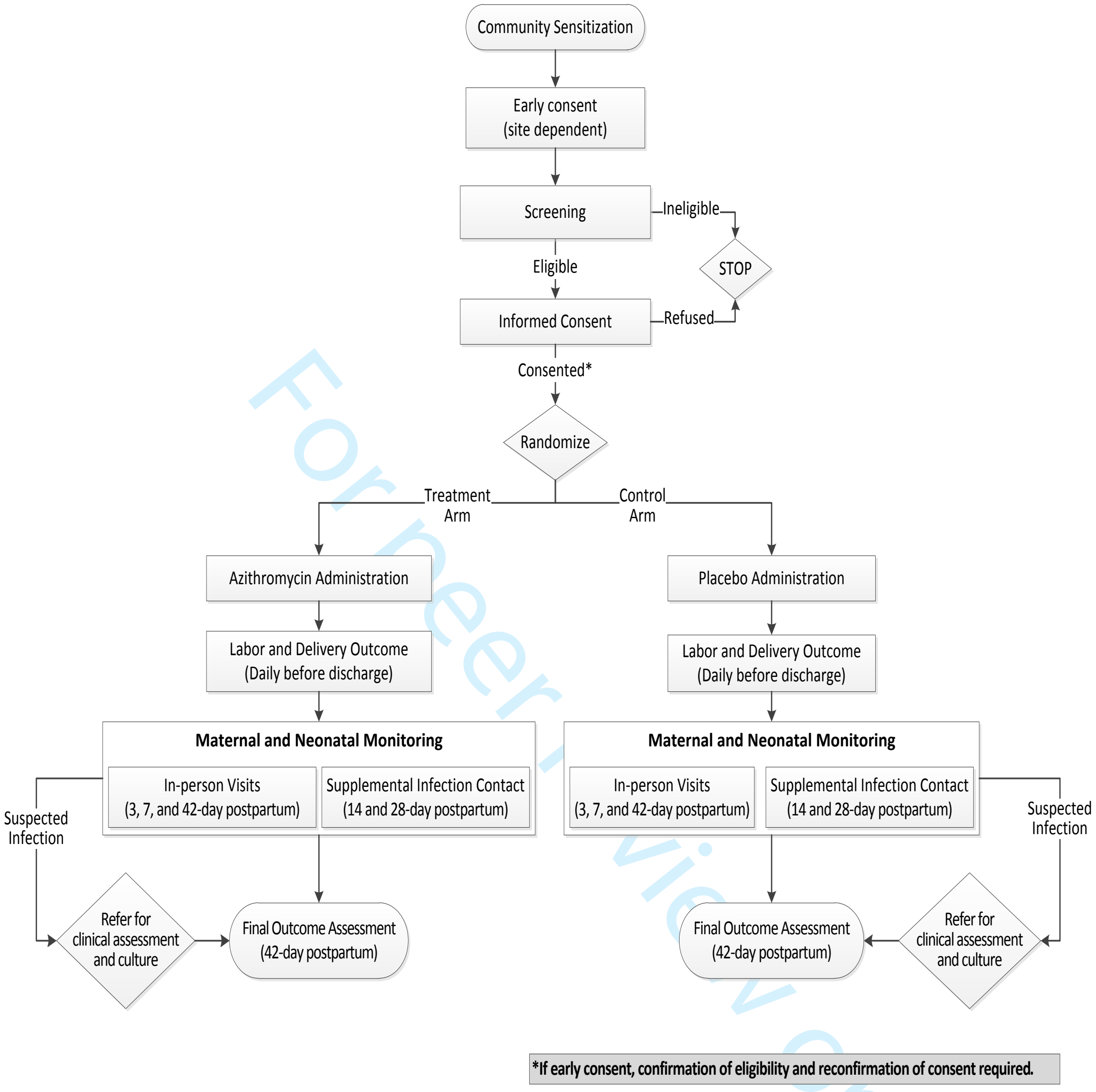

For peer review only

Table 3. Schedule of Study Procedures

	ANC Visits	During Labor/ Before Delivery	After Delivery/ Before Discharge	3-day postpartum (pp)	7-day pp	14-day pp	28-day pp	42-day pp	As Needed
**Community Sensitization**	X								
**Screening**									
▪ *Eligibility confirmation*		X							
▪ *Clinical assessment*		X							
**Consent**		X							
**Randomization**		X							

For peer review only - http://bmjopen.bmj.com/site/about/guidelines.xhtml

	ANC Visits	During Labor/ Before Delivery	After Delivery/ Before Discharge	3-day postpartum (pp)	7-day pp	14-day pp	28-day pp	42-day pp	As Needed
**Drug Administration**		X							
**Baseline Data Collection**									
▪ *Sociodemographic information*		X	X						
▪ *Medical history*		X	X						
▪ *Labor and delivery information*			X						
**Monitoring**									
▪ *Drug side effects*		X	X	X	X			X	X
▪ *Maternal events during labor/delivery*		X	X						
▪ *Neonatal events during labor/delivery*			X						
▪ *Maternal infection/sepsis assessment*			X	X	X			X	X
▪ *Neonatal infection/sepsis assessment*			X	X	X			X	X
▪ *Maternal death*			X	X	X			X	X
▪ *Stillbirth or neonatal death within 28 days of birth*			X	X	X	X	X	X	X
▪ *Infant mortality after 28 days of birth*								X	X
▪ *Pyloric stenosis*			X	X	X			X	X
▪ *Other maternal outcomes*			X	X	X			X	X
▪ *Other neonatal outcomes*			X	X	X			X	X
▪ *Unintended medical visits*			X	X	X			X	X
**Serious Adverse Events**			X	X	X			X	X

Site Preparation

Prior to the trial, sites will conduct an observational pilot study using the RCT's planned infrastructure to characterize current practices and optimize identification of suspected infection. The pilot study data will be used to validate estimates of intrapartum deaths, maternal sepsis, and neonatal sepsis for the RCT’s sample size. Each site will also meet with local health authorities and conduct community sensitization to ensure that study procedures are appropriate for the local context and to encourage engagement at the facility and community level.

Screening

Pregnant women admitted for delivery at pre-defined health facilities will be screened for eligibility by research staff, per harmonized criteria (**Table 1**). If a contraindication to participation is found, the woman will be excluded.

For peer review only - http://bmjopen.bmj.com/site/about/guidelines.xhtml

Consent

All eligible women must provide informed consent for the trial. Consent must be obtained prior to complete cervical dilation or dilation limit approved by local authorities. To minimize burden on the laboring women, sites may obtain initial consent from potential participants during the antenatal period and reconfirm consent at screening. As literacy levels vary, the consent process will include a verbal review of the consent form.

Masking

Both the azithromycin and placebo will be procured from the same manufacturer (Idifarma, Spain). The packaging will be standardized across sites and labeled as: "Azithromycin 2 g or Placebo" with the expiration date and a unique identifier. Clinical and research staff, and participants, will be masked to treatment status unless a serious adverse event potentially related to the treatment requires unmasking. Each site's study pharmacist will monitor randomization, drug supply, and safety.

Randomization

Study drug, sequentially prepared per the randomization sequence in identical packaging, will be dispensed by the study staff. A computer algorithm generated by the DCC will create the random assignment based on randomly permuted block design with randomly varied block sizes known only by the DCC personnel to achieve a 1:1 randomization, stratified by site. Each site will receive a lot of study drug to be distributed sequentially at the participating health facilities.

Monitoring before Discharge (Baseline Assessment)

Routine post-delivery care will be provided by clinical providers, trained to contact research staff in case of suspected or confirmed infection. Research staff will collect demographic characteristics, key clinical measures, and outcomes from randomization until discharge. Data will be abstracted from medical records or collected directly from participants, as relevant. In addition, participants will be educated about the signs and symptoms of infection and encouraged to call research staff with any concerns.

Monitoring after Discharge (Postpartum Follow-up Assessments)

After discharge, research staff will contact participants at the following timepoints:

- In-person visits at 3-day, 7-day, and 42-day postpartum to identify maternal or infant infection, unexpected medical visits, side effects, and other study outcomes. If suspected infection, research staff will collect and send relevant specimens to the local microbiology lab for analysis.
- Supplemental phone contacts at 14-day and 28-day postpartum to review maternal and neonatal signs of infection (WHO criteria). If signs are identified, participants will visit a study facility for further assessment. These supplemental contacts will reinforce the participants' ability to self-assess maternal and neonatal infection and improve identification between the 7-day and 42-day visits.

If indicated, records of unscheduled (non-study) visits to health providers during the follow-up period will be reviewed to ascertain study outcomes. Treating providers may be called if clarification is needed. Readmissions and related diagnoses identified during follow-up visits will be validated through medical record review.

For peer review only - http://bmjopen.bmj.com/site/about/guidelines.xhtml

For peer review only

Safety Monitoring

Surveillance of maternal side effects including nausea, vomiting, and diarrhea/loose stools, abdominal pain, vaginitis, and dizziness potentially associated with azithromycin will be conducted during labor and postpartum. For infants, findings suggestive of pyloric stenosis will be assessed during the follow up visits. Maternal and neonatal surveillance will also include assessment of unintended medical visits, maternal deaths, stillbirths, neonatal death <28 days of birth, and infant death >28 days. Additional maternal and neonatal risks associated with azithromycin use include anaphylaxis, allergic reactions (rash), liver failure, and arrhythmias. Although rare, these side effects will be monitored and reported as serious adverse events. All safety outcomes will be reviewed by the Data Monitoring Committee (DMC) appointed by NICHD.

Analytical Plan

Baseline demographic characteristics and key clinical measures will be compared between the treatment arms using contingency table approaches for categorical variables and analysis of variance models' continuous variables.

P-values presented will be based on two-sided tests unless otherwise specified, adjusted for site. For most analyses, the interaction between treatment and site will be assessed and if significant, also be presented by site. For continuous outcomes, distributional properties will be evaluated and if required, transformations or non-parametric tests employed. Additional details for potential covariate adjustments in secondary analyses or handling violations of analytic method assumptions will be detailed in the statistical analysis plan.

Three key populations are of interest for study analyses:

1. The Intention to Treat (ITT) population will include all women randomized and their infants. Analyses of this cohort will be conducted based on randomized treatment.
2. The high risk for infection sub-group will include all women in the ITT and their infants' meeting criteria for being high risk (i.e., prolonged labor and/or rupture of membranes) at randomization. Analyses of this cohort will be conducted based on randomized treatment.
3. The as-treated population will include all randomized participants that receive any study drug during the study and their infants. Analyses of this cohort will be conducted based on treatment received.

The final determination of analysis population membership will be via a masked data review prior to final study analyses to address any potential anomalous cases that may arise in this large study population (e.g., randomization/treatment of a woman who is discharged prior to delivery due to false labor or unresponsiveness to induction).

Primary Analysis

Incidence of maternal death or sepsis and intrapartum/neonatal death or sepsis will be compared separately between the treatment arms using generalized linear models fit with each binary outcome separately as the outcome measure. Estimates of RR and associated 95% CIs will be reported. The model will include terms for treatment and site. As randomization occurs at the pregnancy level and approximately 1-2% of pregnancies are anticipated to be multiple gestations, models for neonatal outcomes will account for correlation among multiples assuming an exchangeable covariance structure. For the two primary outcomes, analyses will be conducted using the ITT population and the p-values

For peer review only - http://bmjopen.bmj.com/site/about/guidelines.xhtml

associated with the treatment term will be used to formally test each of the two primary hypotheses at the alpha=0.05 level.

As secondary analyses of the primary outcomes, assuming an overall treatment effect is observed, the models will be run including region (Africa, Latin America, or Asia) and a treatment by region interaction term. If the interaction term has a p<0.1, effects will be reported by region with treatment effect within region tested at the 0.025 level.

Additional exploratory models will also be run including individually: 1) a treatment-by-site interaction term, 2) any other antibiotic use during labor (yes or no) and its interaction with treatment, and 3) mode of delivery (cesarean or vaginal) and its interaction with treatment. If the interaction term for any of these models has a p<0.1, then effects will also be reported by the relevant subgroups. These exploratory secondary models will also include any demographic or clinical variables found to differ significantly between the treatment arms in the preliminary analyses described above.

From each final model, estimates of relative risk associated with treatment will be obtained including unadjusted estimates of risk from the primary model as well as estimates of risk adjusted for potential confounders from the secondary analyses.

Secondary Analyses: Women at high-risk for infection cohort

The major secondary aim is assessing the two primary outcomes (i.e., incidence of maternal death or sepsis and incidence of intrapartum/neonatal death or sepsis) in the high-risk cohort. These analyses will also assess if the treatment effect differs between the high-risk sub-group vs. non-high-risk women, which include all women and their infants in the ITT population that delivered prior to meeting high-risk criteria. Specifically, the primary analysis of both the maternal and neonatal primary outcomes will be run including a treatment by risk status interaction term and exclude data from individuals meeting high-risk criteria after randomization.

Secondary Analyses: Other Secondary Outcomes

Other maternal and neonatal binary outcomes will be analyzed using the approaches described for the primary analysis for the ITT population and approaches detailed for the secondary analysis for the HR cohort. A similar process with generalized linear models employing an appropriate link function will be used to analyze the outcomes of maternal and neonatal initial hospital length of stay. Binary safety outcomes will also be analyzed using the approaches detailed for the primary analysis. These analyses will be conducted using the as-treated population.

Sample Size for Primary Outcome

Sample size estimates were generated to evaluate the potential benefits of peripartum prophylactic azithromycin in two population cohorts of women. The first population comprises all women delivering in facilities.

Power calculations for the overall study population were generated for the primary maternal and neonatal outcome measures. For each, estimates of the required sample size needed to detect a risk reduction of 20%, 25%, and 30% were generated for power of 0.8, 0.85, and 0.9. The risk of sepsis or maternal death was assumed to be 3%. For the neonatal outcome, the underlying risk of the combined outcome was estimated to be 8% and 14% based on Global Network data. [34] We assumed that the risk of sepsis not resulting in death is approximately 4% to 10% (**Table 4**). We will also test the neonatal risk

For peer review only - http://bmjopen.bmj.com/site/about/guidelines.xhtml

separately in south Asia and sub-Saharan Africa (**Table 4**). Approximately 37.5% of randomized mothers will be from sub-Saharan Africa and 50% will be from Asia, reflecting the rates observed in the Global Network.[34,35].

Table 4. Sample Sizes

**5A. Sample Size for the Overall Population (alpha=0.05)**				
		Evaluable Sample Size per Arm		
Baseline Risk	Risk Reduction	Power=0.80	Power=0.85	Power=0.90
3%	20%	11455	13103	15334
3%	25%	7133	8159	9548
3%	30%	4815	5508	6446
8%	20%	4096	4686	5483
8%	25%	2554	2921	3419
8%	30%	1727	1975	2311
14%	20%	2204	2521	2950
14%	25%	1377	1575	1842
14%	30%	932	1066	1247
**5B. Sample Size for High-risk Group (alpha = 0.05)**				
6%	20%	5568	6369	7453
6%	25%	3470	3969	4644
6%	30%	2344	2681	3138
**5C. Sample Size by Region (alpha =0.025)**				
8%	20%	4961	5607	6477
8%	25%	3093	3496	4038
8%	30%	2091	2363	2729
14%	20%	2669	3017	3485
14%	25%	1677	1884	2176
14%	30%	1129	1276	1474

For the primary neonatal outcome of interest of intrapartum/neonatal sepsis or death, assuming that the loss to follow-up will be in the 2% to 3% range, this sample size of 34,000 will provide 90% power to detect a 25% reduction in neonatal mortality and sepsis in the sub-Saharan African region and will provide 90% power to detect a 20% reduction in Asia assuming the baseline risk is at least 8%. For the primary maternal outcome of maternal death or sepsis, the sample size will provide 90% power to detect a 20% reduction from 3% in the population aggregated across all study sites.

Data Monitoring Plan and Stopping Rules

Each site will report data, including adverse events, to the Global Network's DCC (RTI International). The data will be used to evaluate protocol adherence and site performance (e.g., recruitment, loss to follow-up, data quality). The DCC will provide standardized progress reports to NICHD and the site investigators monthly to monitor outcome variables and adverse events.

Oversight of the trial will be handled by two principal groups: (1) a protocol-focused Steering Committee (**Table 5**); and (2) a DMC, designated by NICHD to ensure safe and ethical treatment of study participants through bi-annual (at minimum) review of data on participant safety and study progress/futility. One formal interim analysis of efficacy and futility will be conducted by the DMC during the study.

For peer review only - http://bmjopen.bmj.com/site/about/guidelines.xhtml

Table 5. A-PLUS Steering Committee

A-PLUS CENTRAL STUDY TEAM	
A-PLUS Lead Study Site (University of Alabama-Birmingham/University Teaching Hospital, Lusaka, Zambia)	
Waldemar A. Carlo, MD<br>Principal Investigator<br>University of Alabama at Birmingham, United States	Alan Tita, MD, PhD<br>A-PLUS Lead Investigator<br>University of Alabama at Birmingham, United States
Elwyn Chomba, MBChB, DCH, MRCP<br>Senior Foreign Investigator<br>University Teaching Hospital, Lusaka, Zambia	Musaku Mwenechanya<br>Country Coordinator<br>University Teaching Hospital, Lusaka, Zambia
Trecious Mweemba<br>A-PLUS Coordinator<br>University Teaching Hospital, Lusaka, Zambia	
RTI International (Global Network Data Coordinating Center)	
Elizabeth M. McClure, PhD<br>Principal Investigator<br>RTI International	Tracy Nolen, DrPh<br>Co-Principal Investigator and Senior Statistician<br>RTI International
Jennifer J. Hemingway-Foday, MPH, MSW<br>A-PLUS Protocol Manager<br>RTI International	
Eunice Kennedy Shriver National Institute of Child Health and Human Development (NICHD)	
Marion Koso-Thomas, MD, MPH<br>Medical Officer, Global Network for Women's and Children's Health	
**A-PLUS RESEARCH SITES**	
Democratic Republic of Congo (Kinshasa School of Public Health/University of North Carolina)	
Antoinette Tshefu, M.D, Ph.D., M.P.H<br>Senior Foreign Investigator<br>Kinshasa School of Public Health, Kinshasa, DRC	Carl L. Bose, M.D.<br>Principal Investigator<br>University of North Carolina School of Medicine, U.S.
Adrien Lokangaka, M.D., M.P.H.<br>Country Coordinator<br>Kinshasa School of Public Health	Gustave Lomendje<br>A-PLUS Coordinator<br>Kinshasa School of Public Health
Guatemala (Instituto de Nutricion de Centro America y Panama [INCAP]/University of Colorado)	
Manolo Mazariegos, MD, MPH<br>Senior Foreign Investigator,<br>INCAP, Guatemala City, Guatemala	Nancy F. Krebs, MD<br>Principal Investigator,<br>University of Colorado Health Science Center
Lester Figueroa<br>Country Coordinator<br>INCAP, Guatemala City, Guatemala	
**Bangladesh (International Centre for Diarrhoeal Disease Research [ICDDR,b]/University of Virginia)**	
Rashidul Haque, MD<br>Senior Foreign Investigator<br>ICDDR,b	William Petri, MD<br>Principal Investigator<br>University of Virginia
Sk Masum Billah<br>Country Coordinator<br>ICDDR,b	Md Shahjahan Siraj<br>A-PLUS Coordinator<br>ICDDR,b
**Belagavi, India (KLE University's JN Medical College/Thomas Jefferson University)**	
Shivaprasad S. Goudar MD, MHPE<br>Senior Foreign Investigator<br>KLE University's J N Medical College	Richard Derman, MD, MPH<br>Principal Investigator<br>Thomas Jefferson University

For peer review only - http://bmjopen.bmj.com/site/about/guidelines.xhtml

Avinash Kavi<br>A-PLUS Coordinator<br>KLE University's J N Medical College	Mrityunjay Metgud<br>A-PLUS Coordinator<br>KLE University's J N Medical College
**Pakistan (Aga Khan University/Columbia University)**	
Sarah Saleem, MD<br>Senior Foreign Investigator<br>Aga Khan University	Robert L. Goldenberg, MD<br>Principal Investigator<br>Columbia University
Shiyam Sunder Tikmani<br>A-PLUS Coordinator<br>Aga Khan University	
**Nagpur, India (Lata Medical Research Foundation/Boston University)**	
Archana Patel, MD, DNB, MSCE<br>Senior Foreign Investigator<br>Lata Medical Research Foundation	Patricia L. Hibberd, MD, PhD<br>Principal Investigator<br>Boston University School of Public Health
Prabir Das<br>Country Coordinator<br>Lata Medical Research Foundation	Kunal Kurhe<br>A-PLUS Coordinator<br>Lata Medical Research Foundation
**Kenya (Moi University School of Medicine/University of Indiana School of Medicine)**	
Fabian Esamai, MBChB, MMed, PhD<br>Senior Foreign Investigator<br>Moi University School of Medicine, Eldoret, Kenya	Edward A. Liechty, MD<br>Principal Investigator<br>Indiana University School of Medicine
Paul Nyongesa<br>Country Coordinator<br>Moi University School of Medicine, Eldoret, Kenya	Amos Sagwe<br>A-PLUS Coordinator<br>Moi University School of Medicine, Eldoret, Kenya
Osayame Austine Ekhaguere<br>Co-Investigator<br>Indiana University School of Medicine	

Adverse Event Monitoring Plan

Adverse events will be reported and submitted to the DCC (and IRBs) who will report these cumulative masked data to the DMC in the bi-annual safety reviews. Safety reports will be reviewed internally by the DCC quarterly and the DMC chair will be notified if any potential safety signals are identified to allow for more frequent DMC monitoring if needed.

ETHICS AND DISSEMINATION

Ethics Approval and Consent to Participate

The A-PLUS study protocol has been reviewed and approved by the relevant ethics committees and regulatory authorities at each research site, including the Institutional Review Board at the University of Alabama at Birmingham, which serves as the lead site, and the DCC at RTI International. The DMC has reviewed the study protocol and will continue to review throughout the enrollment period. The study was registered (clinicaltrials.gov NCT03871491).

All research staff responsible for obtaining informed consent will be trained and certified in the protection of human subjects and the study-specific consent procedures. A model written informed consent form, developed according to the requirements of the U.S. Office for Human Research Protection (OHRP), is provided to each site; The model consent form may be modified to conform to local standards, but the OHRP required elements must be maintained.

For peer review only - http://bmjopen.bmj.com/site/about/guidelines.xhtml

Potential Risks and Benefits to Participation

There are several potential direct and indirect benefits of this trial. Emerging data suggest that intrapartum azithromycin reduces maternal and neonatal infection. As infections are a frequent cause of maternal and neonatal deaths, there is a possibility that mortality could be reduced at the participating sites, as well as worldwide.

An ongoing concern for peripartum and perinatal antibiotic prophylaxis is the selection of resistant organisms including azithromycin-resistant organisms leading to resistant infections, and concern that disruption of gut and other flora (microbiome) in women and particularly in neonates may lead to adverse events including allergic reactions, rash and childhood asthma.[16,28,36] There is a paucity of data to address concerns that disturbances in the establishment of the indigenous intestinal microbiome caused by antibiotic exposure in early life or CD, either directly or through modifications of breast microbiome, may increase risk of immune-mediated and inflammatory conditions later in life.[37-40] In response to these global public health concerns, this protocol includes an ancillary study to monitor for antimicrobial resistance and changes to the maternal and newborn microbiome.

Dissemination

Following completion of primary data collection, we will disseminate findings through international meetings and high impact journals. If the results are positive, we will facilitate the change in practice at participating sites and approach the WHO to instigate guideline updates to reflect the study findings.

After completion of the study, the clean and de-identified dataset will be retained by RTI International and included in a de-identified public use database managed by NICHD Data and Specimen Hub (DASH) in compliance with the U.S. National Institutes of Health (NIH) Public Access Policy. Each site will also maintain a copy of their site-specific database.

For peer review only

For peer review only - http://bmjopen.bmj.com/site/about/guidelines.xhtml

References

1. WHO recommendations for the prevention and treatment of maternal peripartum infections. [Internet]. World Health Organization. World Health Organization; 2015 [retrieved 2018 Aug 22]. Available from: https://www.who.int/publications/i/item/9789241549363
2. Liu L, Oza S, Hogan D, Perin J, Rudan I, Lawn JE, et al. Global, regional, and national causes of child mortality in 2000-13, with projections to inform post-2015 priorities: an updated systematic review. Lancet 2015;385(9966), 430-440.
3. Fleischmann-Struzek C, Goldfarb DM, Schlattmann P, Schlapbach LJ, Reinhart K, Kissoon N. The global burden of paediatric and neonatal sepsis: a systematic review. Lancet Respir Med. 2018;6(3):223–30.
4. Fleischmann C, Reichert F, Cassini A, Horner R, Harder T, Markwart R, et al. Global incidence and mortality of neonatal sepsis: a systematic review and meta-analysis. Arch Dis Child. 2021;106(8):745-52.
5. Baguiya A, Bonet M, Cecatti JG, Brizuela V, Curteanu A, Minkauskiene M, et al; WHO Global Maternal Sepsis Study (GLOSS) Research Group; GLOSS research group. Perinatal outcomes among births to women with infection during pregnancy. Arch Dis Child. 2021;106(10):946-53.
6. Brizuela V, Cuesta C, Bartolelli G, Abdosh AA, Abou Malham S, Assarag B, et al; WHO GLOSS Research Group. Availability of facility resources and services and infection-related maternal outcomes in the WHO Global Maternal Sepsis Study: a cross-sectional study. Lancet Glob Health. 2021;9(9):e1252-e1261.
7. World Health Organization. (2017). Statement on maternal sepsis. Retrieved August 22, 2018, from http://www.who.int/reproductivehealth/publications/maternal_perinatal_health/maternalsepsis-statement/en/
8. Tshefu, A., Lokangaka, A., Ngaima, S., Engmann, C., Esamai, F., Gisore, P., . . . African Neonatal Sepsis Trial (AFRINEST) group. Simplified antibiotic regimens compared with injectable procaine benzylpenicillin plus gentamicin for treatment of neonates and young infants with clinical signs of possible serious bacterial infection when referral is not possible: a randomised, open-label, equivalence trial. Lancet 2015; 385(9979), 1767-1776.
9. Baqui AH, Saha SK, Ahmed AS, Shahidullah M, Quasem I, Roth DE, et al. Projahnmo Study Group in Bangladesh. Safety and efficacy of alternative antibiotic regimens compared with 7 day injectable procaine benzylpenicillin and gentamicin for outpatient treatment of neonates and young infants with clinical signs of severe infection when referral is not possible: a randomised, open-label, equivalence trial. Lancet Global Health 2015; 3(5), e279-287.
10. Mir F, Nisar I, Tikmani SS, Baloch B, Shakoor S, Jehan F, et al. Simplified antibiotic regimens for treatment of clinical severe infection in the outpatient setting when referral is not possible for young infants in Pakistan (Simplified Antibiotic Therapy Trial [SATT]): a randomised, open-label, equivalence trial. Lancet Global Health 2017; 5(2), e177-e185.
11. Zaidi AK, Tikmani SS, Sultana S, Baloch B, Kazi M, Rehman H, et al. Simplified antibiotic regimens for the management of clinically diagnosed severe infections in newborns and young infants in first-level facilities in Karachi, Pakistan: study design for an outpatient randomized controlled equivalence trial. Pediatric Infectious Disease Journal 2013; 32 Suppl 1(Suppl 1), S19-25.
12. World Health Organization. WHO Statement on Caesarean Section Rates. 2015. Retrieved August 22, 2018, from

For peer review only - http://bmjopen.bmj.com/site/about/guidelines.xhtml

http://www.who.int/reproductivehealth/publications/maternal_perinatal_health/cs-statement/en/
13. Mackeen AD, Packard RE, Ota E, Berghella V, Baxter JK. Timing of intravenous prophylactic antibiotics for preventing postpartum infectious morbidity in women undergoing cesarean delivery. Cochrane Database of Systematic Reviews 2014. 12(12), CD009516.
14. Smaill FM, Grivell RM. Antibiotic prophylaxis versus no prophylaxis for preventing infection after cesarean section. Cochrane Database of Systematic Reviews 2014. 10, CD007482.
15. Gyte GML, Dou L, Vazquez JC. Different classes of antibiotics given to women routinely for preventing infection at caesarean section. Cochrane Database of Systematic Reviews. 2014; 11(11), CD008726.
16. Tita ATN, Rouse DJ, Blackwell S, Saade GR, Spong CY, Andrews WW. Evolving concepts in antibiotic prophylaxis for cesarean delivery: A systematic review. Obstetrics and Gynecology. 2009;113(3):675–82
17. Harrison MS, Saleem S, Ali S, Pasha O, Chomba E, Carlo WA, et al. A Prospective, Population-Based Study of Trends in Operative Vaginal Delivery Compared to Cesarean Delivery Rates in Low- and Middle-Income Countries, 2010-2016. Am J Perinatol. 2019;36(7):730-736.
18. Tita ATN, Boggess K, et al. Adjunctive azithromycin prophylaxis for cesarean delivery. New Eng J Med 2015. 375:1231-1241.
19. Oluwalana C, Camara B, Bottomley C, Goodier S, Bojang A, Kampmann B et al. Azithromycin in labor lowers clinical infections in mothers and newborns: a double-blind trial. Pediatrics 2017, 139(2).
20. Hume-Nixon M, Quach A, Reyburn R, Nguyen C, Steer A, Russell F. A Systematic Review and meta-analysis of the effect of administration of azithromycin during pregnancy on perinatal and neonatal outcomes. EClinicalMedicine. 2021;40:101123.
21. Hallamaa L, Cheung YB, Maleta K, Luntamo M, Ashorn U, Gladstone M, Kulmala T, Mangani C, Ashorn P. Child Health Outcomes After Presumptive Infection Treatment in Pregnant Women: A Randomized Trial. Pediatrics. 2018;141(3):e20172459.
22. Fitzgibbon A, Clooney L, Broderick D, Eogan M, McCallion N, Drew RJ. Erythromycin compared to amoxicillin and azithromycin for antimicrobial prophylaxis for preterm premature rupture of the membranes: a retrospective study. J Obstet Gynaecol. 2021 May;41(4):569-572.
23. Sutton AL, Acosta EP, Larson KB, Kerstner-Wood CD, Tita AT, Biggio JR. Perinatal pharmacokinetics of azithromycin for cesarean prophylaxis. Am J Obstet Gynecol 2018; 212(6), 812.
24. Russell NJ, Seale AC, O'Driscoll M, O'Sullivan C, Bianchi-Jassir F, Gonzalez-Guarin J, et al; GBS Maternal Colonization Investigator Group. Maternal Colonization With Group B Streptococcus and Serotype Distribution Worldwide: Systematic Review and Meta-analyses. Clin Infect Dis. 2017 Nov 6;65(suppl_2):S100-S111.
25. Rac H, Gould AP, Eiland LS, Griffin B, McLaughlin M, Stover KR, Bland CM, Bookstaver PB. Common Bacterial and Viral Infections: Review of Management in the Pregnant Patient. Ann Pharmacother. 2019 Jun;53(6):639-651.
26. Harper LM, Kilgore M, Szychowski JM, Andrews WW, Tita ATN. Economic evaluation of adjunctive azithromycin prophylaxis for cesarean delivery. Obstet Gynec 2017; 130(2), 328-334.
27. McClure EM, Garces AL, Hibberd PL, Moore JL, Goudar SS, Saleem S, et al. The Global Network Maternal Newborn Health Registry: a multi-country, community-based registry of pregnancy outcomes. Reprod Health. 2020;17(Suppl 2):184.

For peer review only - http://bmjopen.bmj.com/site/about/guidelines.xhtml

28. Bonet, M., Souza, J. P., Abalos, E., Fawole, B., Knight, M., Kouanda, S., et al. The global maternal sepsis study and awareness campaign (GLOSS): study protocol. Reprod Health 2018; 15(1), 16.
29. Albright, C.M., Has, P., Rouse D.J., Hughes B.L. Internal Validation of the Sepsis in Obstetrics Score to Identify Risk of Morbidity from Sepsis in Pregnancy. Obstetric Anesthesia Digest 2018; 38 (1).
30. Bowyer L., Robinson H.L., Barrett H., Crozier T.M., Giles M., Idel I., et al. SOMANZ guidelines for the investigation and management sepsis in pregnancy. Aust. N. Z. J. Obstet. Gynaecol. 2017;57:540–51.
31. World Health Organization. Guideline: Managing Possible Serious Bacterial Infection in Young Infants When Referral is not Feasible. 2015. (Retrieved August 18, 2020 from https://apps.who.int/iris/bitstream/handle/10665/181426/9789241509268_eng.pdf)
32. Pasha O, McClure EM, Saleem S, Tikmani SS, Lokangaka A, Tshefu A, et al. A prospective cause of death classification system for maternal deaths in low and middle-income countries: results from the Global Network Maternal Newborn Health Registry. BJOG. 2018;125(9):1137-43.
33. Garces AL, McClure EM, Pérez W, Hambidge KM, Krebs NF, Figueroa L, et al. The Global Network Neonatal Cause of Death algorithm for low-resource settings. Acta Paediatr. 2017;106(6):904-11.
34. Goudar SS, Goco N, Somannavar MS, Kavi A, Vernekar SS, Tshefu A, et al. Institutional deliveries and stillbirth and neonatal mortality in the Global Network's Maternal and Newborn Health Registry. Reprod Health. 2020 Dec 17;17(Suppl 3):179.
35. Hoffman MK, Goudar SS, Kodkany, B. S., Goco, N., Koso-Thomas, M., Miodovnik, M., et al. A description of the methods of the aspirin supplementation for pregnancy indicated risk reduction in nulliparas (ASPIRIN) study. BMC Pregnancy and Childbirth 2017; 17(1), 135.
36. Roca A, O. C., Bojang A, Camara B, Kampmann B, Bailey R, Demba A, Bottomley C, D'Alessandro U. (2016). Oral azithromycin given during labour decreases bacterial carriage in the mothers and their offspring: a double-blind randomized trial. *Clin Microbiol Infect. , 22*(6), e1-9.
37. Roberts, S., Maccato, M., Faro, S., & Pinell, P. (1993). The microbiology of post-cesarean wound morbidity. *Obstetrics and Gynecology, 81*(3), 383-386.
38. Rosene, K., Eschenbach, D. A., Tompkins, L. S., Kenny, G. E., & Watkins, H. (1986). Polymicrobial early postpartum endometritis with facultative and anaerobic bacteria, genital mycoplasmas, and Chlamydia trachomatis: treatment with piperacillin or cefoxitin. *Journal of Infectious Diseases, 153*(6), 1028-1037.
39. Andrews, W. W., Shah, S. R., Goldenberg, R. L., Cliver, S. P., Hauth, J. C., & Cassell, G. H. (1995). Association of post-cesarean delivery endometritis with colonization of the chorioamnion by Ureaplasma urealyticum. *Obstetrics and Gynecology, 85*(4), 509-514. https://doi.org/10.1016/0029-7844(94)00436-H
40. Keski-Nisula, L., Kirkinen, P., Katila, M. L., Ollikainen, M., Suonio, S., & Saarikoski, S. (1997). Amniotic fluid U. urealyticum colonization: significance for maternal peripartal infections at term. *American Journal of Perinatology, 14*(3), 151-156.

For peer review only - http://bmjopen.bmj.com/site/about/guidelines.xhtml

ACKNOWLEDGEMENTS

The authors would like to acknowledge the A-PLUS research teams for their many contributions to this work:

Kinshasa School of Public Health, Kinshasa, Democratic Republic of Congo: Michel Kalonji and Miyalu Junior; *University of North Carolina, Chapel Hill, North Carolina, United States*: Jackie Patterson; *University Teaching Hospital, Lusaka, Zambia:* Ernest Banda, Mwansa Chimfwembe, Ruth Nakazwe, and Abigail Mwapule; *Instituto de Nutrición de Centroamérica y Panamá, Guatemala City, Guatemala*: Maynor Manrique; *University of Colorado School of Medicine, Denver, CO*, USA: Jamie Westcott; *International Centre for Diarrhoeal Disease Research, Dhaka, Bangladesh*: Qazi Sadeq-ur-Rahman, Amita Farzana, Farhana Jahan, Zarin Tasnim Maliha, and Lolit Singh; *University of Virginia, Charlottesville, Virginia, United States:* Chris Chisolm; *KLE Academy Higher Education and Research, J N Medical College Belagavi, Karnataka, India:* Sheetal Harakuni, Manjunath Somannavar, and Kadappa Beniwadi; *Thomas Jefferson University, Philadelphia, PA, USA:* Frances Jaeger; *Aga Khan University, Karachi, Pakistan:* Zaheer Habib, Imran Ahmed, Farnaz Naqvi, Naija Ghanchi, and Saleem Jessani; *Lata Medical Research Foundation, Nagpur, India:* Vaishali Khedikar, Savita Bhargav, Samreen Sadaf, and Chaitali Gedam; Moi University School of Medicine, Eldoret, Kenya; Milsort Kemboi, Anderson Misati, and Kevin Otieno; *Indiana School of Medicine, University of Indiana, Indianapolis, IN, USA*: Sheri Bucher; *RTI International, Research Triangle Park, North Carolina, United States:* Anna Aceituno, Kay Jackson, Jean Kim, Janet Moore, Suchita Parepalli, Marissa Trotta, and Alexis Williams.

AUTHOR'S CONTRIBUTIONS

JHF wrote the first draft, revised and reviewed the final version with WAC, AT, and EMM. TN developed the statistical analyses plan; all co-authors provided input into the study protocol and reviewed the final manuscript.

FUNDING STATEMENT

This A-PLUS study is supported by the following grants from the *Eunice Kennedy Shriver* National Institute of Child Health and Human Development (NICHD) and the Bill & Melinda Gates Foundation (BMGF) through grants to the Foundation for the National Institute of Health (FNIH):

Institution	NICHD Grant Number	BMGF Grant Number
University of North Carolina at Chapel Hill – C. Bose	U10 HD076465	
University of Alabama at Birmingham – W. Carlo	U10 HD078437	
University of Colorado – N. Krebs	U10 HD076474	
Thomas Jefferson University – R. Derman	U10 HD076457	
Columbia University – R. Goldenberg	U10 HD078438	
Boston University – P. Hibberd	U10 HD078439	
Indiana University – E. Liechty	U10 HD076461	
RTI International – E. McClure	U01 HD040636	MCCL19APT

COMPETING INTERESTS STATEMENT

The authors declare that they have no competing interests.

For peer review only - http://bmjopen.bmj.com/site/about/guidelines.xhtml

EXCLUSIVE LICENCE STATEMENT:

I, the Submitting Author has the right to grant and does grant on behalf of all authors of the Work (as defined in the below author licence), an exclusive licence and/or a non-exclusive licence for contributions from authors who are: i) UK Crown employees; ii) where BMJ has agreed a CC-BY licence shall apply, and/or iii) in accordance with the terms applicable for US Federal Government officers or employees acting as part of their official duties; on a worldwide, perpetual, irrevocable, royalty-free basis to BMJ Publishing Group Ltd ("BMJ") its licensees and where the relevant Journal is co-owned by BMJ to the co-owners of the Journal, to publish the Work in BMJ Open and any other BMJ products and to exploit all rights, as set out in our licence.

The Submitting Author accepts and understands that any supply made under these terms is made by BMJ to the Submitting Author unless you are acting as an employee on behalf of your employer or a postgraduate student of an affiliated institution which is paying any applicable article publishing charge ("APC") for Open Access articles. Where the Submitting Author wishes to make the Work available on an Open Access basis (and intends to pay the relevant APC), the terms of reuse of such Open Access shall be governed by a Creative Commons licence – details of these licences and which Creative Commons licence will apply to this Work are set out in our licence referred to above.

For peer review only

For peer review only - http://bmjopen.bmj.com/site/about/guidelines.xhtml

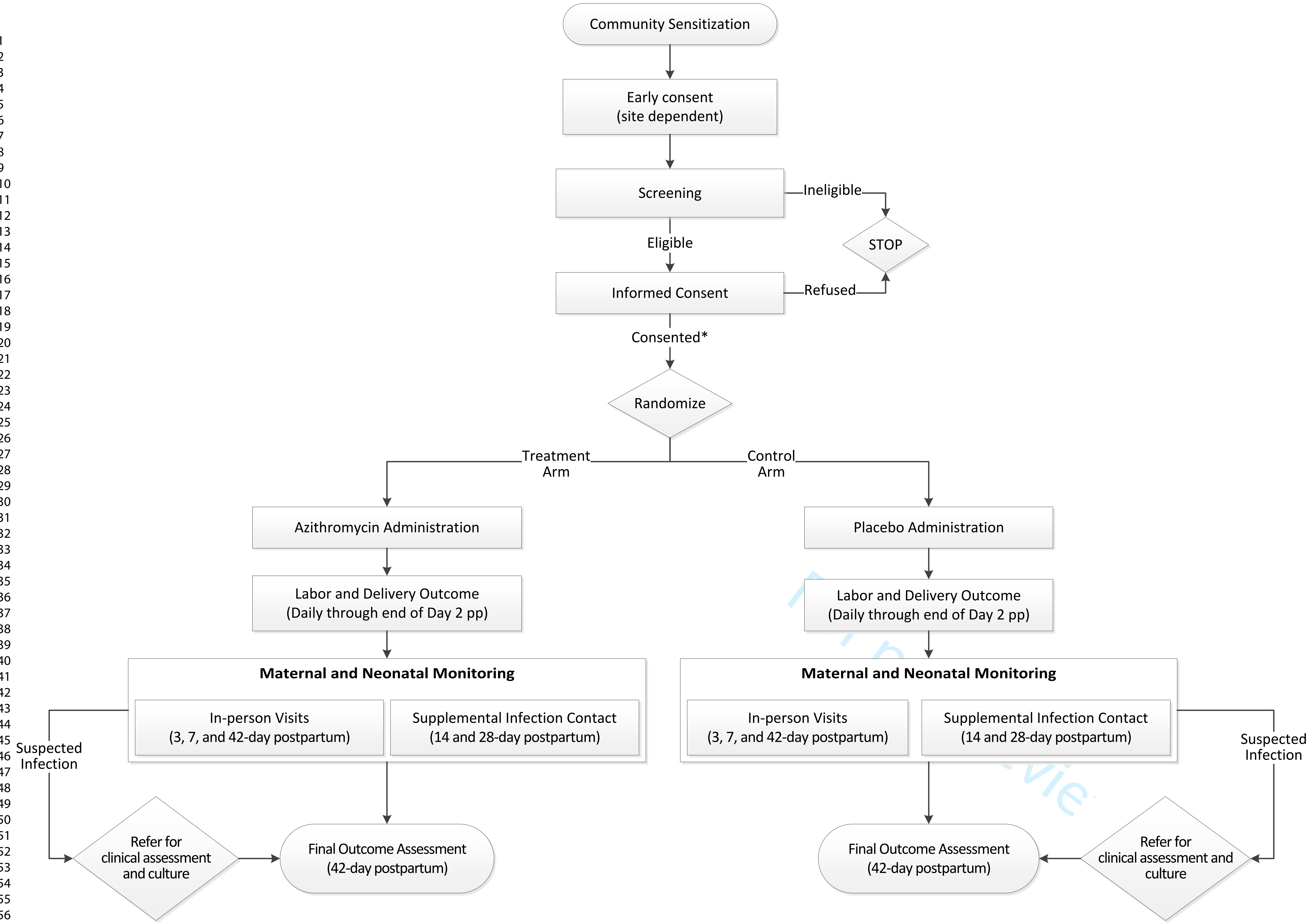

***If early consent, confirmation of eligibility and reconfirmation of consent required.**

Prevention of maternal and neonatal death/infections with a single oral dose of azithromycin in women in labor (in low- and middle-income countries): A randomized controlled trial

ClinicalTrials.gov number: NCT03871491

Lead Study Investigator(s):
Alan T.N. Tita, MD, PhD, Center for Women's Reproductive Health and Maternal-Fetal Medicine Division, UAB
Wally Carlo, MD, Neonatology Division, UAB
Elwyn Chomba, MBChB, DCH, MRCP, University Teaching Hospital

Subcommittee Members:
Marion Koso-Thomas, MD, NICHD
Tracy Nolen, DrPh, RTI International
Elizabeth McClure, PhD, RTI International
Jennifer J. Hemingway-Foday, MPH, RTI International

Version Number: Version 1.6

Version Date: July 15, 2022

Funding: Eunice Kennedy Shriver National Institute of Child Health and Human Development and The Bill & Melinda Gates Foundation

For peer review only

For peer review only - http://bmjopen.bmj.com/site/about/guidelines.xhtml

Version Tracking

Version	Date	Authors	Comments
1.0	Sep 27, 2018	A-PLUS WG	This version will be used for IRB/ERC submissions.
1.1	Dec 19, 2018	A-PLUS WG	Internal version to document and review proposed revisions from GN investigators.
1.2	Dec 20, 2018	A-PLUS WG and GN Investigators	At request of GN investigators, revisions made to the following sections: Inclusion and exclusion criteria (Section 2.2); Consent procedures (Section 2.4); Description of data forms (Section 4.1); Sample informed consent form (Appendix 2); and in-text references.<br>**This version will be used for IRB/ERC submissions after December 20, 2018.**
1.3	January 24, 2020	A-PLUS WG	Revisions made based on feedback from pilot study and requests from GN investigators. The following sections have been revised:<br>• Section 1 (Statement of Problem), including 1.2 and 1.3 (Primary and Secondary Specific Aims) and 1.5 (Rationale/Justification)<br>• Section 2 (Methods), including 2.1 (Inclusion Criteria); 2.4.7 (Monitoring after Discharge); 2.5 (Primary Outcomes); 2.6 (Other Maternal Outcomes); 2.7 (Other Neonatal Outcomes) and 2.8 (Safety Monitoring)<br>• Section 3 (Analytical Plan)<br>• Section 4.1 (Data Forms)<br>• Section 5 (References)<br>• Figure 1. Flowchart of Study Activities<br>• Appendix 2. Sample Informed Consent<br>• Appendix 3. Schedule of Study Procedures
1.4	January 29, 2020	A-PLUS WG	<u>Additional revisions made to:</u><br>• Section 1.3 (Secondary Specific Aims)<br>• Section 2.5 (Primary outcomes)<br>• Section 2 (Methods), including 2.6 (Other Maternal Outcomes) and 2.7 (Other Neonatal Outcomes)<br>• Section 3 (Analytical Plan, including 1.2 (Secondary Analysis-Women at High Risk for Infection Cohort) 3.2.1 (Sample Size for Primary Outcome) and 3.2.2 (Sample Size for High-risk Women)<br>**This version will be submitted to IRBs/ERCs at all GN sites.**
1.5	February 09, 2021	A-PLUS WG	<u>Updates made based on recommendations from the Data Monitoring Committee:</u><br>• In Section 2.5 (Primary Outcome), removed urinary tract infection, omphalitis, eye infection, and skin infection from neonatal sepsis definition, removed fast breathing from the pSBI definition, and changed the pSBI fever criteria to 38 C/100.4 F.<br>• In Section 2.7 (Other Neonatal Outcomes), added urinary tract infection, omphalitis, and fast breathing to the "other neonatal infection" outcome.<br><u>Other updates:</u><br>• In Section 3.5.1 (Reporting Adverse Events), made minor text changes to align with revised AE reporting procedures. [Refer to A-PLUS Technical Memo #2]<br>• In Section 3.5.4 (Interim/Adverse Event Monitoring Plan), made minor text revisions to clarify that the interim analysis will be conducted in both the all-comers and high-risk cohort. This revision was made at the request of the Boston University IRB.<br>• In list of Azithromycin Investigators, updated SFI for GN06-Guatemala to Manolo Mazariegos.<br>**This version will be submitted to IRBs/ERCs at all GN sites.**
1.6	12 July, 2022	A-PLUS WG	High-risk definition updated to "Prolonged labor [≥18 hours] and/or prolonged membrane rupture [≥8 hours]". The following sections have been updated to reflect this change:<br>• Abstract-*Secondary Outcomes*<br>• Section 1.3 (Secondary Specific Aims)<br>• Section 1.5 (Rationale/Justification)<br>• Section 1.6.1 (The Gambian and Cameroon Trials) *[Note: new reference added to this section, with full citation in reference list; as a result, all subsequent references have been renumbered]*<br>• Section 2.2.2 (Exclusion Criteria)<br>• Section 3 (Analytical Plan)<br>• Section 3.1.2 (Secondary Analysis-Women at High Risk for Infection Cohort)<br>• Section 3.2.2 (Sample Size for High-risk Women)<br>• Appendix 2 (Sample Consent)<br>Other minor updates were made to the list of "Azithromycin Investigators" to reflect staffing changes since protocol V. 1.5 was released.<br>**This version will be submitted to IRBs/ERCs at all GN sites.**

For peer review only - http://bmjopen.bmj.com/site/about/guidelines.xhtml

Table of Contents

Azithromycin Investigators 6

ACRONYMS 9

Abstract 10

1 Statement of Problem 11

1.1 Primary Hypotheses 11

1.2 Primary Specific Aims 11

1.3 Secondary Specific Aims 11

1.4 Background 12

1.4.1 Prevention of Maternal and Neonatal Deaths from Infections 12

1.4.2 Risks for Maternal and Neonatal Infections 12

1.4.3 Intrapartum Azithromycin to Prevent Maternal and Neonatal Infection 13

1.5 Rationale/Justification 13

1.6 Previous Studies 14

1.6.1 The Gambian and Cameroon Trials 14

1.6.2 The US Trial and Cost Analysis 14

1.6.3 Other Important Considerations 15

2 Methods 16

2.1 Study Design 17

2.2 Study Population/Location 17

2.2.1 Inclusion Criteria 17

2.2.2 Exclusion Criteria 17

2.3 Study Intervention and Comparison 18

2.4 Detailed Study Procedures 18

2.4.1 Community Sensitization 18

2.4.2 Screening 18

For peer review only

For peer review only - http://bmjopen.bmj.com/site/about/guidelines.xhtml

2.4.3 Consent 18
2.4.4 Masking 19
2.4.5 Randomization Procedures 20
2.4.6 Monitoring before Discharge 20
2.4.7 Monitoring after Discharge 20
2.4.8 Sequence of Study Activities 21
2.5 Primary Outcomes 22
2.6 Other Maternal Outcomes 22
2.7 Other Neonatal Outcomes 23
2.8 Safety Monitoring 23
2.9 Site Preparation 24
2.10 Potential Risks and Benefits to Participants 24
3 Analytical plan 25
3.1 Statistical Analysis Plan 25
3.1.1 Primary Analyses 25
3.1.2 Secondary Analyses – Women at High Risk for Infection Cohort 26
3.1.3 Secondary Analyses – Other Secondary Outcomes 26
3.2 Sample Size 27
3.2.1 Sample Size for Primary Outcome 27
3.2.2 Sample Size for High-Risk Women 28
3.3 Available Population 29
3.4 Projected Recruitment Time 29
3.5 Study Monitoring Plan 29
3.5.1 Reporting Serious Adverse Events 29
3.5.2 Method and Timing for Reporting Serious Adverse Events 30
3.5.3 Data Monitoring Plan and Stopping Rules 30
3.5.4 Interim/Adverse Event Monitoring Plan 31
3.5.5 Risks/Benefits 32
3.6 Quality Control 32

For peer review only

For peer review only - http://bmjopen.bmj.com/site/about/guidelines.xhtml

3.6.1 Training 32

3.6.2 Study Monitoring 32

3.6.3 Drug Quality Assurance and Monitoring 32

3.6.4 Plan for Sustaining Intervention 33

4 Data Management Procedures 33

4.1 Data Forms 33

5 References 35

Appendix 1. Description of Participating Global Network Sites 38

Appendix 2. Sample Informed Consent 40

Appendix 3. Schedule of Study Procedures 44

For peer review only

For peer review only - http://bmjopen.bmj.com/site/about/guidelines.xhtml

AZITHROMYCIN INVESTIGATORS

Central Team: Global Network Site 03 (Zambia)

Waldemar A. Carlo, MD
Principal Investigator
University of Alabama at Birmingham
Birmingham, Alabama
wcarlo@peds.uab.edu

Alan T.N. Tita, MD, PhD
Azithromycin Protocol Lead Investigator
University of Alabama at Birmingham
Birmingham, Alabama
atita@uabmc.edu

Elwyn Chomba, MBChB, DCH, MRCP
Senior Foreign Investigator
University Teaching Hospital
Lusaka, Zambia
chombaelwyn@gmail.com

Site Investigators

Global Network Site 02 (Democratic Republic of Congo)

Carl Bose, M.D.
Principal Investigator
University of North Carolina School of Medicine
Chapel Hill, North Carolina
cbose@med.unc.edu

Antoinette Tshefu, MD, PhD, MPH
Senior Foreign Investigator
Kinshasa School of Public Health
antotshe@yahoo.com

Global Network Site 03 (Zambia)

Wally Carlo, MD
Principal Investigator
University of Alabama at Birmingham
Birmingham, Alabama
Wcarlo@PEDS.UAB.EDU

Elwyn Chomba, MBChB, DCH, MRCP
Senior Foreign Investigator
University Teaching Hospital
Lusaka, Zambia
chombaelwyn@gmail.com

Global Network Site 06 (Guatemala)

Nancy Krebs, MD
Principal Investigator
UCHSC
Denver, Colorado
Nancy.krebs@ucdenver.edu

For peer review only

For peer review only - http://bmjopen.bmj.com/site/about/guidelines.xhtml

Manolo Mazariegos, MD
Senior Foreign Investigator
INCAP
Guatemala City, Guatemala
mmazariegos@incap.int

Global Network Site 07 (Bangladesh)

William Petri, MD
Principal Investigator
University of Virginia
Charlottesville, Virginia
wap3g@virginia.edu

Rashidul Haque, MD
Senior Foreign Investigator
ICDDR,B
Dhaka, Bangladesh
rhaque@icddrb.org

Global Network Site 08 (Belagavi, India)

Richard Derman, MD, MPH
Principal Investigator
Thomas Jefferson University
Philadelphia, PA
richard.derman@jefferson.edu

Shivaprasad S. Goudar MD, MHPE
Senior Foreign Investigator
KLE University's J N Medical College
Belgaum, India
sgoudar@jnmc.edu

Global Network Site 09 (Pakistan)

Robert L. Goldenberg, MD
Principal Investigator
Columbia University
New York, New York
rlg88@columbia.edu

Sarah Saleem, MD
Senior Foreign Investigator
Aga Khan University
Karachi, Pakistan
sarah.saleem@aku.edu

Global Network Site 11 (Nagpur, India)

Patricia L. Hibberd, MD, PhD
Principal Investigator
Boston University
Boston, Massachusetts
plh0@bu.edu

Archana Patel, MD, DNB, MSCE, PhD
Senior Foreign Investigator

For peer review only

For peer review only - http://bmjopen.bmj.com/site/about/guidelines.xhtml

Lata Medical Research Foundation
Nagpur, India
Dr_apatel@yahoo.com

Global Network Site 12 (Kenya)

Ed Liechty, MD
Principal Investigator
Indiana University School of Medicine
Indianapolis, Indiana
eliecht@iupui.edu

Fabian Esamai, MBChB, MMed, MPH, PhD
Senior Foreign Investigator
Moi University School of Medicine
Eldoret, Kenya
fesamai2007@gmail.com

National Institute of Child Health and Human Development (NICHD)

Marion Koso-Thomas, MD, MPH
Medical Officer, Global Network for Women's and Children's Health
Eunice Kennedy Shriver National Institute of Child Health and Human Development (NICHD)
kosomari@mail.nih.gov

RTI International

Elizabeth McClure, PhD
Principal Investigator, Data Coordinating Center
RTI International, Durham, NC
mcclure@rti.org

Tracy Nolen, DrPh
Senior Statistician, Data Coordinating Center
tnolen@rti.org

Jennifer J. Hemingway-Foday, MPH, MSW
Protocol Manager for Azithromycin Protocol, Data Coordinating Center
hemingway@rti.org

For peer review only

For peer review only - http://bmjopen.bmj.com/site/about/guidelines.xhtml

ACRONYMS

**AC**	All-comers
**ACOG**	American Congress of Obstetricians and Gynecologists
**ANC**	Antenatal Care
**BP**	Blood pressure
**DCC**	Data Coordinating Center
**DMC**	Data monitoring committee
**DMS**	Data Management System
**DRC**	Democratic Republic of Congo
**ERC**	Ethical review Committee
**FIGO**	International Federation of Gynecology and Obstetrics
**FDA**	Food and Drug Administration
**GA**	Gestational age
**GI**	Gastrointestinal
**GN**	Global Network for Women's and Children's Health Research
**HR**	High-risk
**IRB**	Institutional Review Board
**JAMA**	Journal of the American Medical Association
**ITT**	Intention to treat
**LIC**	Low-income country
**LMIC**	Low- and middle-income countries
**LMP**	Last menstrual period
**MNH**	Maternal and Newborn Health
**NEJM**	New England Journal of Medicine
**NICHD**	Eunice Kennedy Shriver National Institute of Child Health and Human Development
**NIH**	National Institutes of Health
**NNT**	Number needed to treat
**OHRP**	U.S. Office of Research Protections
**PI**	Principal Investigator
**pSBI**	possible serious bacterial infection
**RCT**	Randomized controlled trial
**RTI**	Research Triangle Institute International
**SAE**	Serious adverse event
**SC**	Steering Committee
**SFI**	Senior Foreign Investigator
**SMFM**	Society for Maternal-Fetal Medicine
**UAB**	University of Alabama at Birmingham
**WHO**	World Health Organization

For peer review only

For peer review only - http://bmjopen.bmj.com/site/about/guidelines.xhtml

ABSTRACT

Background: Maternal and neonatal infections are among the most frequent causes of maternal and neonatal deaths, and current antibiotic strategies have not been effective in preventing many of these deaths. Recently, a randomized clinical trial conducted in a single site in The Gambia showed that treatment with oral dose of 2 g azithromycin vs. placebo for all women in labor reduced certain maternal and neonatal infections. However, it is unknown if this therapy reduces maternal and neonatal sepsis and mortality.

Hypotheses: The trial includes two primary hypotheses, a maternal hypothesis and a neonatal hypothesis. First, a single, prophylactic intrapartum oral dose of 2 g azithromycin given to women in labor will reduce maternal death or sepsis. Second, a single, prophylactic intrapartum oral dose of 2 g azithromycin given to women in labor will reduce intrapartum/neonatal death or sepsis.

Study Design Type: Randomized, placebo-controlled, parallel multicenter clinical trial. Women in labor will be randomized with one-to-one ratio to intervention/placebo.

Population: Pregnant women in labor at ≥28 weeks gestational age with a live fetus pregnancy who plan to deliver vaginally in a facility. Women with evidence of chorioamnionitis or other infection requiring antibiotic therapy (not prophylaxis) at time of eligibility, allergy to azithromycin, use of azithromycin, erythromycin or other macrolide within 3 days of enrollment, known arrhythmia or cardiomyopathy, or plan for cesarean section delivery prior to enrollment will be excluded.

Intervention: A single, prophylactic intrapartum oral dose of 2 g azithromycin.

Comparison: A single intrapartum oral dose of an identical appearing placebo

Outcomes:
Primary outcomes: 1) Incidence of maternal death or sepsis and 2) incidence of intrapartum/neonatal death or sepsis.

Secondary outcomes: Individual components of the primary outcomes (maternal death, maternal sepsis, intrapartum/neonatal death, neonatal sepsis), neonatal death/sepsis, neonatal deaths due to sepsis, and all-cause neonatal death; the primary maternal and neonatal outcome in a high-risk for infection population; specific maternal infections; use of subsequent maternal antibiotic therapy; pyloric stenosis; health care resource utilization; culture-positive infections and resistance; in an ancillary surveillance study, incidence of antimicrobial resistance and microbiome diversity.

For peer review only

For peer review only - http://bmjopen.bmj.com/site/about/guidelines.xhtml

1 STATEMENT OF PROBLEM

1.1 Primary Hypotheses

This study will have two primary hypotheses, one with a maternal focus and one with a neonatal focus. First, a single, prophylactic intrapartum oral dose of 2 g azithromycin given to women in labor in low and middle-income settings will reduce maternal death or sepsis. Second, a single, prophylactic intrapartum oral dose of 2 g azithromycin given to women in labor in low and middle-income settings will reduce intrapartum/neonatal death or sepsis.

1.2 Primary Specific Aims

To test the effectiveness of a single dose of prophylactic intrapartum azithromycin compared to placebo in reducing the risk of the composite outcome of maternal death or sepsis. To separately test the effectiveness of a single oral dose of intrapartum azithromycin prophylaxis (2 g) compared to placebo in reducing the risk of the composite outcome of intrapartum/neonatal death or sepsis. Both groups will receive the routine or usual care provided at the facility during and after labor.

1.3 Secondary Specific Aims

The single dose of intrapartum azithromycin prophylaxis (2 g) will be compared to placebo to accomplish the following secondary aims:

- <u>Main Secondary Aim for Women at High-Risk for Infection Cohort:</u>
    a. To evaluate whether the risk of maternal death or sepsis differs among laboring women with and without high-risk for infection (high risk is defined as women with prolonged labor [≥18 hours] and/or prolonged membrane rupture [≥8 hours] at time of randomization).
- <u>Other secondary specific Aims:</u>
    b. To evaluate the effectiveness in reducing the risk of individual components of the primary composite endpoints (i.e., maternal death, maternal sepsis, intrapartum/neonatal death, neonatal sepsis including specifically all-cause neonatal deaths and neonatal deaths due to sepsis).
    c. To evaluate the effectiveness on the risk of intrapartum/neonatal death or sepsis in infants of laboring women at high-risk for infection because of prolonged labor (≥18 hours) and/or prolonged membrane rupture (≥8 hours).
    d. To evaluate the effectiveness on the risk of maternal infections including clinical chorioamnionitis, endometritis, wound infections (perineal or Cesarean), abdominal or pelvic abscess, mastitis/breast abscess or infection, pyelonephritis, pneumonia and other bacterial infections in all laboring women as well as in those at high-risk for infection.
    e. To evaluate the effectiveness in reducing the use of subsequent maternal antibiotic therapy from randomization to 42 days postpartum for any reason in all laboring women as well as in those at high-risk for infection.
    f. To compare the use of health care resources. Use of health care resources will be measured in terms of maternal and neonatal duration of hospital stay, unscheduled visits, readmission, and admission to special care units/intensive care units in all laboring women and newborns as well as in those at high-risk for infection.
    g. To assess maternal GI symptoms (e.g., nausea, vomiting, and diarrhea) and other reported side effects, as well as infant pyloric stenosis and type of culture positive infections

For peer review only - http://bmjopen.bmj.com/site/about/guidelines.xhtml

(antimicrobial resistance and maternal and infant microbiome diversity are compared in an ancillary study).

h. To determine whether the effect on each of the primary outcomes differs by region (Africa, Latin America or Asia), any other antibiotic use during labor, and mode of delivery (cesarean or vaginal). Note that antibiotic use will not include use to treat any infections diagnosed after randomization as those will be outcomes.

1.4 Background

Maternal and neonatal infections are among the most frequent causes of maternal and neonatal death. Maternal infection during pregnancy and the puerperium account for approximately 10% of the global burden of maternal deaths. This places maternal infection among the top five causes of maternal mortality worldwide.[1] Neonatal infection is the third most common cause of neonatal mortality and accounts for about 16% of neonatal mortality worldwide.[2] Furthermore, maternal and neonatal deaths from infections are not decreasing compared with deaths from other frequent causes of mortality.[1,2] There is a need for innovative simple effective interventions that can be scaled up to reduce the burden of both maternal and neonatal mortality due to infections.

1.4.1 Prevention of Maternal and Neonatal Deaths from Infections

Current strategies to prevent maternal and neonatal deaths from infections are insufficient. Current approaches to prevention, identification, and treatment of neonatal sepsis have had limited impact. According to the WHO, maternal deaths from infection have remained unchanged or increased in some instances, whereas deaths from other causes have reduced. The evidence backing current WHO guidelines for prevention and treatment of peripartum infections is generally graded as low or very low quality.[1] Efforts for early identification and treatment of neonatal infection are important but neonatal deaths due to infection continue to be very prevalent. Recent studies of alternative antibiotic regimens that do not require a full course of intravenous antimicrobial therapy have shown comparable effectiveness but have not reduced deaths from infections.[3-6] Among research priorities, a WHO guideline panel identified the evaluation of the role of routine prophylactic antibiotics in women who undergo a normal vaginal birth as well as in those at high risk for infection due to prolonged labor or membrane rupture.[1]

1.4.2 Risks for Maternal and Neonatal Infections

Maternal and neonatal infections by maternal risk including cesarean delivery. While the role of antibiotic prophylaxis for cesarean delivery is well-established, in many low-resource settings, many maternal infections occur after vaginal deliveries with high-risk for infections. Cesarean delivery especially after labor or membrane rupture is the strongest risk factor for maternal peripartum infection including endometritis, wound infection and sepsis (increasing risk by up to 5 to 10 times compared to vaginal delivery).[7] The fraction of maternal infection attributable to cesarean delivery is rather low because of the low cesarean delivery rate of 5-10% or lower, especially in the Africa region, compared with 20-30% or higher in the US and many high income countries.[8] Therefore, strategies that address maternal peripartum infection and sepsis in the developing world should focus as well on identifying and preventing (as well as treating) infection in women who have a vaginal birth, particularly those who are at high-risk. There is an increased risk of infection in women who undergo prolonged labor ≥18 hours (at least 2 fold) or membrane rupture ≥6 hours (at least 2-3 fold) compared to women who do not experience these risk factors.[1] These risk factors identify a large group of women who are at the highest

For peer review only - http://bmjopen.bmj.com/site/about/guidelines.xhtml

risk for maternal peripartum infections (including chorioamnionitis, endometritis, perineal wound infection and post-cesarean surgical site infections) and sepsis after a vaginal or cesarean delivery and also place newborns at increased risk for sepsis. However, even though 80-90% of pregnancies in labor are not at this highest-risk for infection depending on criteria used, they may account for about 50% of maternal and neonatal infections in LMICs. Thus, testing antibiotic strategies during labor in LMICs should include both the high-risk and lower risk women as highlighted in research priorities from the WHO guideline panel.[1]

Antibiotic Prophylaxis for Women who deliver by Cesarean section. The appropriate use of antibiotic prophylaxis for cesarean delivery and antiseptic agents are among the most effective preventive interventions as highlighted by the WHO guidelines for maternal peripartum infections.[1] Antibiotic prophylaxis, preferably prior to incision, in particular, among several strategies, effectively reduces the risk of infection and the associated high health care and personal costs.[1,9-11] However, the prevalence of cesarean sections is low, in many low-resource settings, particularly in sub-Saharan Africa, and thus, do not account for the large burden of the maternal and neonatal infections worldwide.

1.4.3 Intrapartum Azithromycin to Prevent Maternal and Neonatal Infection

Azithromycin: a novel approach to maternal and neonatal infections. A novel approach to maternal and neonatal infection is to target organisms that may be very frequent pathogens but that historically have not been the target of antimicrobial treatment.[12] A multicenter randomized clinical trial (RCT) of azithromycin prophylaxis added to the standard prophylactic regimen (cephalosporin) in women who underwent cesarean delivery following labor or membrane rupture for at least 4 hours in the US showed that maternal infection was reduced by about 50%.[13] A single center RCT in a low-income country (LIC) setting suggested that azithromycin prophylaxis may improve maternal and neonatal outcomes. In an RCT in The Gambia that included all women in labor, treatment with 2 g of azithromycin vs. placebo before delivery reduced maternal and neonatal infections.[14] Therefore, we propose to evaluate the effectiveness of a single oral dose of azithromycin as an intrapartum prophylactic agent for maternal and neonatal infection and death. We will also monitor the potential side effects of this intervention.

1.5 Rationale/Justification

Maternal infection and sepsis is a priority to reduce maternal and neonatal deaths. Compared to postpartum hemorrhage and preeclampsia/eclampsia, maternal infection has received less attention as a major cause of maternal death; proportionally it accounts for increasing deaths.[1] The WHO and other global health authorities identified maternal infection/sepsis as a priority problem to reduce maternal deaths. In addition, maternal infection significantly increases the risk of neonatal sepsis which is one of the leading causes of neonatal death in LICs.[15] Most recently, a NEJM perspective article highlighted the WHO resolution issuing a clarion call recognizing “Sepsis” as a global health priority.[16] Drawing from our findings on azithromycin prophylaxis for cesarean delivery in the US[13] and data from another preliminary trial in The Gambia, Africa,[14] we propose to evaluate the role of a single oral dose of azithromycin (plus usual care) to prevent maternal death or peripartum sepsis and intrapartum/neonatal death or sepsis in laboring women as well as the targeted sub-population of those at the highest risk for infection because they have prolonged labor (≥18 hours) and/or prolonged membrane rupture (≥8 hours).

Azithromycin is available as a generic agent with easy storage requirements. It has a bimodal half-life of up to 70 hours in the non-pregnant population. Although its pharmacokinetic characteristics are not as

For peer review only - http://bmjopen.bmj.com/site/about/guidelines.xhtml

well studied in the pregnant population, it is commonly used during pregnancy for treatment of chlamydia and other infections. Azithromycin covers a broad spectrum of bacteria (including gram-positive cocci, genital ureaplasmas and mycoplasmas, and certain gram-negative bacilli and anaerobes) that are associated with maternal infections which are often polymicrobial (chorioamnionitis, endometritis, and perineal/cesarean wound infection) and sepsis. In addition to the aforementioned organisms which may play a role in neonatal infection, azithromycin also has activity against Group B streptococcus which is a major cause of neonatal sepsis in developed countries and may be implicated in LICs and low-resources settings as well. Therefore, a successful prophylaxis intervention is likely to reduce infections and death and may also reduce health care costs and need for prolonged antibiotic therapy to treat infections which may be associated with resistance. Indeed, the recent WHO Guidelines on peripartum infection articulated the following among research priorities: what are the benefits of initiating prophylactic antibiotics among women undergoing uncomplicated vaginal birth and among those at high risk such as after prolonged rupture of membranes?[1] A JHPIEGO consultative meeting on enhancing the focus on maternal infections suggested that "Attention to identification and prompt management of prolonged labor and prolonged rupture of membranes is critical to reduce disease and death due to maternal sepsis."[15,16,17]

1.6 Previous Studies

1.6.1 The Gambian and Cameroon Trials

Data from a single center trial in a LIC setting suggest the potential for azithromycin prophylaxis to improve maternal and neonatal outcomes. Among 829 Gambian mothers (randomized to 2 g of azithromycin vs. placebo before delivery) and their 830 newborns, maternal infections were lower in the azithromycin group (3.6% vs 9.2%; relative risk [RR], 0.40; 95% confidence interval [CI], 0.22-0.71; P = 0.002).[14] Among newborns, the overall prevalence of infections was also lower in the azithromycin group (18.1% vs 23.8%; RR, 0.76; 95% CI, 0.58-0.99; P = 0.052).[14] Maternal and neonatal carriage of infectious organisms was lower in the azithromycin group.[18] A test of concept trial focusing on high-risk women in Cameroon (labor ≥18 hours or membrane rupture ≥8 hours at randomization was underpowered by suggested lower maternal infection in women who received azithromycin.[19]

1.6.2 The US Trial and Cost Analysis

In a multicenter US RCT, it was demonstrated a further 50% reduction in the risk of maternal peripartum infection by adding a single 500 mg intravenous dose of azithromycin to the standard prophylactic regimen (a single intravenous dose of cefazolin 1-2 g or ampicillin) in the highest-risk group of women who undergo cesarean delivery following labor or membrane rupture for at least 4 hours.[13] These results were observed despite universal use of other antibiotics in both arms of the trial. Specifically, the 95% without penicillin allergy had a cephalosporin (mainly cefazolin) for usual cesarean prophylaxis; the remainder that gentamicin and clindamycin. In addition, 25-30% received a penicillin for GBS prophylaxis. Infection was significantly lower in the azithromycin group compared to the placebo group; 6.1% vs. 12%; RR =0.51 (95 % CI 0.38 to 0.68); p<0.001. Specifically, adjunctive azithromycin use was associated with significant reductions in the risks of endometritis (3.8% vs. 6.1%, RR=0.62, 95% CI: 0.42-0.92; p=0.02) and wound infections (2.4% vs. 6.6%, RR=0.35, 95% CI: 0.22-0.56; p<0.001). The number needed to treat (NNT) to prevent one infection was 17 for any infection, 43 for endometritis and 24 for wound infection. Other maternal outcomes including need for readmission or unscheduled visits for any reason or specifically for infection (decreased by up to 50%), serious adverse events, postpartum fever,

For peer review only - http://bmjopen.bmj.com/site/about/guidelines.xhtml

or subsequent treatment with antibiotics were also significantly less common with azithromycin prophylaxis. Short-term perinatal/infant outcomes including deaths, sepsis, and other serious neonatal morbidities were rare in this developed country population and did not differ between groups.[13] In a related cost-analysis report, it was estimated that use of adjunctive azithromycin saves approximately $360 for each use in unscheduled (high-risk) cesarean deliveries such as those studied in the RCT and $143 per use in scheduled or pre-labor cesarean delivery.[20] The results suggesting cost-savings were robust across wide ranges of baseline risk of infection and treatment effect size.[20]Thus, in the US alone, adjunctive azithromycin prophylaxis for cesarean delivery could lead to $350M in cost-savings/year due to avoided infection. These works built upon, and are supported by, over 20 years of research on maternal infections at UAB and elsewhere.[21-31] Azithromycin provides coverage against the most common pathogens identified in association with peripartum infections including genital mycoplasmas and ureaplasmas (when specific methods are utilized to identify them).

1.6.3 Other Important Considerations

A recurrent concern regarding the potential routine use of azithromycin in a large population is antibiotic resistance. Monitoring for characteristics suggestive of resistant infection will be important to incorporate in the protocol. However, a number of factors mitigate concerns about antibiotic resistance: (a) the design of the trial using a single prophylactic dose (as opposed to recurrent treatment doses) of antibiotic, (b) surveillance of maternal clinical cultures up to 6 weeks in the trial in cesareans in the US revealed that positive wound cultures overall and those positive for resistant organisms were significantly less frequent in the azithromycin group;[13] (c) if successful, prophylaxis will reduce the risk of infection and actually reduce the overall frequency of use of antibiotics to treat infection (a result in the US trial as stated above). It is estimated that women in the high-risk for infection group as defined will account for about 10-20% of laboring women. In principle, the use of single prophylactic dose (as opposed to multiple) minimizes the likelihood of antimicrobial resistance but samples will be taken to determine antimicrobial resistance.[32]

Azithromycin is currently recommended to treat or prevent several infections in pregnancy including gonorrhea (1 g po), chlamydia (1 g po), and Mycobacterium avium complex prophylaxis (600 mg twice/week or 1.2 g weekly po). Azithromycin is sometimes used for perioperative prophylaxis in patients at risk for endocarditis (500 mg po). Considering the success with a single dose of 2 g in the Gambian trial, our success with 500 mg IV for cesarean prophylaxis, and the 40% bioavailability of oral azithromycin, we propose to use 2 g po of azithromycin for the proposed intervention. The best approach to this evaluation in order to influence future uptake into clinical practice is an RCT. The primary maternal outcome will be maternal death or sepsis within 42 days after delivery. The primary neonatal outcome will be intrapartum/neonatal death or sepsis within 28 days after birth (defined by WHO criteria).[33]

Azithromycin is *pregnancy category B* – animal studies using maternally toxic doses showed no fetal harm. Limited studies suggest azithromycin is excreted in human milk in a sustained fashion.[34] There are no specific drug-drug interactions warranting dose adjustments when given with other medications. Elimination is by both hepatic and renal route, and no specific adjustments are mandated for patients with renal or hepatic insufficiency. The long elimination 1/2-life of 68 hours is due to extensive uptake and subsequent release of drugs from tissues. The only absolute contraindications are rare: known hypersensitivity reaction to azithromycin, erythromycin or other macrolide antibiotic or history of

For peer review only - http://bmjopen.bmj.com/site/about/guidelines.xhtml

cholestatic jaundice due to azithromycin. Potential adverse events include very rare (<1%) allergic hypersensitivity (mild and severe skin reactions – Stevens Johnson Syndrome and toxic epidermal necrolysis, angioedema and anaphylaxis) and clostridium difficile-associated diarrhea. With multi-day azithromycin therapy, 0.6% of patients discontinued azithromycin due to side effects. With single 1-2 gram doses, gastrointestinal symptoms (nausea 5-18%, diarrhea/loose stools 7-14%, abdominal pain 5-7%, vomiting 2-7%, and dyspepsia 1%) as well as vaginitis 2% and dizziness (1%) were the most commonly reported side effects. No other side effect occurred with a frequency greater than 1%.

Azithromycin has been associated with hypertrophic pyloric stenosis in some observational studies. In a retrospective study, oral azithromycin exposure during the first 14 days after birth was associated with an increased incidence of pyloric stenosis.[35] However, an increased risk of pyloric stenosis was not reported in infants following a single dose of azithromycin to women in labor in randomized trials in the US and Gambia.[13-14, 16] The much larger sample size of the proposed study affords the opportunity to further explore this question.

The FDA in 2013 issued an advisory regarding concerns about potential for rare life-threatening arrhythmias with azithromycin use particularly among those with preexisting cardiovascular risk. The information was based on an observational study of older, ill patients who received multiple courses of oral azithromycin over 5 days.[36] The findings are not applicable to our current study population for several reasons: the population is much younger, generally without cardiac co-morbidities, a single dose rather than cumulative doses of oral azithromycin over 5 days is being studied, additional studies of younger, healthy patients did not suggest an increased cardiovascular risk[37]; and patients with arrhythmia or known history of cardiomyopathy will be excluded. In addition, the prior study of 2013 women in the US did not suggest a cardiovascular safety signal, and the potential reduction in severe infection may exceed the excess risk of severe arrhythmia with a single dose of azithromycin.[13]

2 METHODS

Pregnant women in labor at study health facilities who appear to be ≥28 weeks gestational age by best clinical estimate with a live fetus pregnancy will be screened for eligibility (see **Section 2.2**) by research staff starting at the time of admission and continuously during the hospitalization. Gestational age will be determined using the "best estimate" algorithm that is currently used in the GN MNH registry. Those eligible will be consented. Those consented will be randomized to receive a single dose of azithromycin 2 g or identical placebo to be given and directly observed during labor. Because of the ease of storage of azithromycin, sequentially prepared medications in identical packages following the randomization sequence will be available and dispensed by the study staff. The rest of the care will be provided according to the local standard of care. Women will be followed up until discharge and surveillance maintained (in-person), with visits at 3 days, 7 days, and 42 days after delivery as has generally been done in the Global Network to ascertain study outcomes.

We will apply the recently recommended "simple and actionable" WHO definition of sepsis in a 2017 statement endorsed by multiple international organizations. The definition of sepsis includes a suspicion of infection and the presence of organ dysfunction based on clinical findings.[15]

To further ensure more objective ascertainment of the sepsis outcome, we propose that the DCC will prospectively define a process for defining criteria for a masked centralized review and adjudication of all

For peer review only - http://bmjopen.bmj.com/site/about/guidelines.xhtml

cases of infection to ensure conformity with the proposed definition. This approach of adjudication is applied to validate infection in many trials including our recently completed ASPIRIN trial.[38] This approach to adjudication has some similarities to the near-miss classification for adjudication in women delivering in the Global Network sites that was done recently.[39]

2.1 Study Design

This study is a masked, placebo-controlled RCT. The investigational regimen is 2 g of azithromycin and the comparison arm is an identical placebo which is given orally. Both groups will also receive the standard of care during labor, delivery and postpartum per local standards.

2.2 Study Population/Location

Pregnant women laboring in health facilities of the eight Global Network sites/other health facilities will be eligible. The Global Network sites are described in **Appendix 1**. Health facilities may include any hospitals and health centers where women routinely deliver within the study sites.

2.2.1 Inclusion Criteria

- Pregnant women in labor ≥28 weeks GA (by best estimate) with a pregnancy with one or more live fetuses who plan to deliver vaginally in a facility.
- Admitted to health facility with clear plan for spontaneous or induced delivery.
- Live fetus must be confirmed via presence of a fetal heart rate prior to randomization.
- ≥18 years of age or minors 14-17 years of age in countries where married or pregnant minors (or their authorized representatives) are legally permitted to give consent.
- Have provided written informed consent [Note: written informed consent may be obtained during antenatal care, but verbal re-confirmation may be needed (per local regulations) at the time of randomization].

2.2.2 Exclusion Criteria

- Non-emancipated minors (as per local regulations)
- Evidence of chorioamnionitis or other infection requiring antibiotic therapy at time of eligibility (however, women given single prophylactic antibiotics with no plans to continue after delivery should not be excluded).
- Arrhythmia or known history of cardiomyopathy.
- Allergy to azithromycin or other macrolides that is self-reported or documented in the medical record.
- Any use of azithromycin, erythromycin, or other macrolide in the 3 days or less prior to randomization.
- Plan for cesarean delivery prior to randomization.
- Preterm labor undergoing management with no immediate plan to proceed to delivery.
- Advanced stage of labor (>6 cm or 10 cm cervical dilation per local standards) and pushing **or** too distressed to understand, confirm, or give informed consent **regardless** of cervical dilation.
- Are not capable of giving consent due to other health problems such as obstetric emergencies (for example, antepartum haemorrhage) or mental disorder.
- Any other medical conditions that may be considered a contraindication per the judgment of the site investigator.
- Previous randomization in the trial.

For peer review only - http://bmjopen.bmj.com/site/about/guidelines.xhtml

Sites may choose to obtain written consent during antenatal care from pregnant women of the age of consent who plan to deliver vaginally in a facility and have no known medical exclusions. In this case, study staff must fully assess and confirm eligibility at the time of randomization. It may also be necessary to verbally re-confirm consent at the time of randomization, if required by local regulations. If it is determined that the study needs to be extended to accrue the planned sample size for HR cohort, then women who deliver prior to meeting HR criteria for randomization will be excluded.

2.3 Study Intervention and Comparison

The study intervention is a single 2 g dose of directly observed oral azithromycin, to be administered as four 500 mg pills or tablets directly after randomization. By random allocation, participants will receive 2 g of oral azithromycin vs. placebo. We will design the placebo with the assistance of a reputable pharmacy/pharmaceutical company, using identical capsules or pills containing azithromycin 2 g or a matching placebo (non-antimicrobial agent) to accomplish masking. All of the participants' obstetric care will be similar for all both arms and consist of the routinely available care at each center.

2.4 Detailed Study Procedures

2.4.1 Community Sensitization

Local health providers will receive sensitization about the study to foster communication and collaboration at the facilities where enrollment will take place. In addition, pregnant women and their families in the enrollment area will receive information about the study during antenatal care (ANC) visits to facilitate recruitment and comprehension during the consenting process. Sites may choose to consent women during these sensitization sessions, prior to labor; however, confirmation of eligibility and consent will be required during the screening process described in **Section 2.4.2 and 2.4.3**.

2.4.2 Screening

Women in labor in defined health facilities (both hospitals and health centers) will be identified by research staff. A brief review of eligibility will be made to determine whether the patient is in labor and does not meet any of the exclusion criteria. If a contraindication to participation in the trial is found, the woman will be excluded from the trial at this point.

2.4.3 Consent

Before a woman participates in the trial, the research staff must obtain her informed consent to voluntarily take part in the study. Consent will be obtained from women ≥18 years of age or minors 14-17 years of age in countries where married or pregnant minors (or their authorized representatives) are legally permitted to give consent. When enrolling minors, we will follow the in-country policies for human research protection and the guidelines approved by the local ethical review committees (ERCs). In the case of pregnant minors, this may require that written consent is obtained from her parents/guardians or husband, with written assent from the minor.

Potential participants will be screened and enrolled in the study upon admission for delivery; therefore, consent will be obtained during labor. Consent should be obtained as early as possible during the intrapartum period and must be obtained prior to the cervical dilation limit approved by local authorities (e.g. either > 6 cm or 10 cm) and/or pushing, as assessed from clinical exam by health facility staff. It is not feasible to wait because the intervention must be given before delivery. Research sites

For peer review only - http://bmjopen.bmj.com/site/about/guidelines.xhtml

may choose to obtain initial consent during ANC; however, in this situation, confirmation of consent will be required during the screening and enrollment process.

If consent is obtained during labor, study staff will make necessary accommodations to ensure that the laboring women can comprehend the information presented during the consent process. Potential participants who present in labor can take as much time as needed to consider participation while in labor and will be able to discuss the study with family/friends if desired before deciding on participation. If the participant cannot read, the form will be read aloud to her by a person unaffiliated with the study. Alternatively, the Research Coordinator or a designate may read the consent, but in the presence of a witness who is unaffiliated with the research study. Potential participants will be given an opportunity to discuss the study procedures and ask questions. Additional details are provided in the study Manual of Procedures.

Fair balance will be maintained while describing the risks and benefits of participation in the study. No undue pressure will be placed on the potential participant to enroll in the trial. It will further be explained that lack of participation will not affect the usual and anticipated standard of care. As the literacy levels will vary and may be a challenge, the consent process will include a verbal review of the consent form.

After the potential participant has read the consent form, but before she signs, the research staff will show her a sample study pill and confirm that she is willing and able to take the study pill as prescribed. Only if she is willing to commit to taking the pill will she be enrolled; otherwise, this will be recorded as a refusal of consent. Following review of the consent, the potential participant (or parent/guardian) will be asked to sign the form. If the potential participant (or parent/guardian) is unable to sign her name, she will be asked to use her thumbprint to indicate written approval. In both cases, the unaffiliated person will also sign the consent form. Both the research staff and the study participant retain signed copies of the form.

An eligible woman may refuse to participate in the trial at the time of recruitment. This will be recorded in the Screening and Recruitment Form. She may also choose to withdraw from the study at any time after enrollment. This will be recorded on the Withdrawal/Termination Form.

All research staff responsible for obtaining consent will be trained and certified in the protection of human subjects and the study-specific consent procedures. A model written informed consent form, developed according to the requirements of the U.S. Office of Research Protections (OHRP), is found in **appendix 2**. Each site may modify the model consent to conform to local standards, but the OHRP required elements must be maintained. The research sites will also be responsible for translating the consent form into the appropriate language(s) for their local context.

Global Network countries with legislation regarding the need to videotape consents will comply with the country regulations; however, this is not part of the consent form requirements. This will not be required by protocol but rather decided by each site so as to comply with local rules and regulations.

2.4.4 Masking

Both the azithromycin and placebo will be procured from the same manufacturer. The packaging will be standardized across sites and will be labeled as: “Azithromycin 2 g or Placebo”, with the expiration data and a unique identifier. A certificate of authenticity will likewise be provided.

For peer review only - http://bmjopen.bmj.com/site/about/guidelines.xhtml

Clinical and research staff as well as the women will be masked to treatment status unless there is a serious adverse event potentially related to the treatment modality that requires unmasking for safety reasons. There will be one pharmacist at each site who will monitor randomization, drug supply, and safety. Under the instruction of the DCC, the study pharmacist will be trained and authorized to apply un-masking procedures, if concerns about randomization or participant safety are identified.

2.4.5 Randomization Procedures

Randomization of participants will be carried out to obtain a 1:1 allocation ratio between the treatment and placebo arms. Randomization will be stratified by site. A computer algorithm generated by the data coordinating center (DCC) will create the random assignment to one of the treatment arms based on randomly permuted block design with randomly varied block sizes. The block sizes will be known only by the DCC personnel. Each site will receive a lot of the study drug to be distributed sequentially at the participating health facilities which are randomizing women for the study site.

2.4.6 Monitoring before Discharge

Routine post-delivery care will be provided to participants by their clinical providers who will be masked to the study interventions. Research staff (also masked to study medications) trained in obstetric and perinatal outcomes abstraction will be responsible for collecting research data from participant medical records or directly from participants, as relevant, before discharge. Data needed to determine sepsis will include temperature (fever or hypothermia) essentially, plus one or more of heart rate (tachycardia), blood pressure (hypotension), respiratory rate (tachypnea or distress), clinical exam (jaundice or altered mental status) and urine output (low) and a suspicion of infection by the clinical provider or research team. Maternal and neonatal outcomes will be evaluated in the hospital following the delivery (on an ongoing basis until discharge). All participants will be asked to agree to maternal/infant medical record release to abstract information for outcome assessment as part of the consent form. In addition, participants will be educated about the signs and symptoms of infection and other study outcomes and encouraged to call the research team with any concerns.

2.4.7 Monitoring after Discharge

After discharge, study staff will contact participants at the following timepoints:

- In-person visits will be conducted at 3-day, 7-day, and 42-day postpartum to identify maternal or infant infection, unexpected medical visits, side effects, and other study outcomes. WHO criteria for infections will be used and included in the data collection forms. In the case of suspected infection, research staff will collect specimens (e.g. blood, urine, or pus from wound or drained abscesses), as is feasible, in order to identify individual infectious agents.

- Supplemental phone contacts will be conducted at 14-day and 28-day postpartum to review maternal and neonatal signs of infection using WHO criteria. If signs of infection are identified during the review, participants will be asked to visit a study facility for further assessment. These supplemental contacts will reinforce the participants' ability to self-assess for signs of maternal and neonatal infection and improve identification of infection between the 7-day and 42-day postpartum visits. If phone contact is not feasible, in-person visits may be conducted.

If indicated, records of unscheduled visits to any health facility prior to the routine study visits will be obtained and reviewed to ascertain study outcomes. Treating providers may also be called if clarification is needed. Based on the scheduled visits, study staff will identify any unscheduled visits or readmissions

For peer review only - http://bmjopen.bmj.com/site/about/guidelines.xhtml

that occurred in the interim. Readmissions and related diagnoses identified during follow-up visits will be then be validated through medical record review.

2.4.8 Sequence of Study Activities

The sequence of study activities is described in **Figure 1**. A detailed scheduled of study procedures is found in **Appendix 3**.

Figure 1. Flowchart of Study Activities

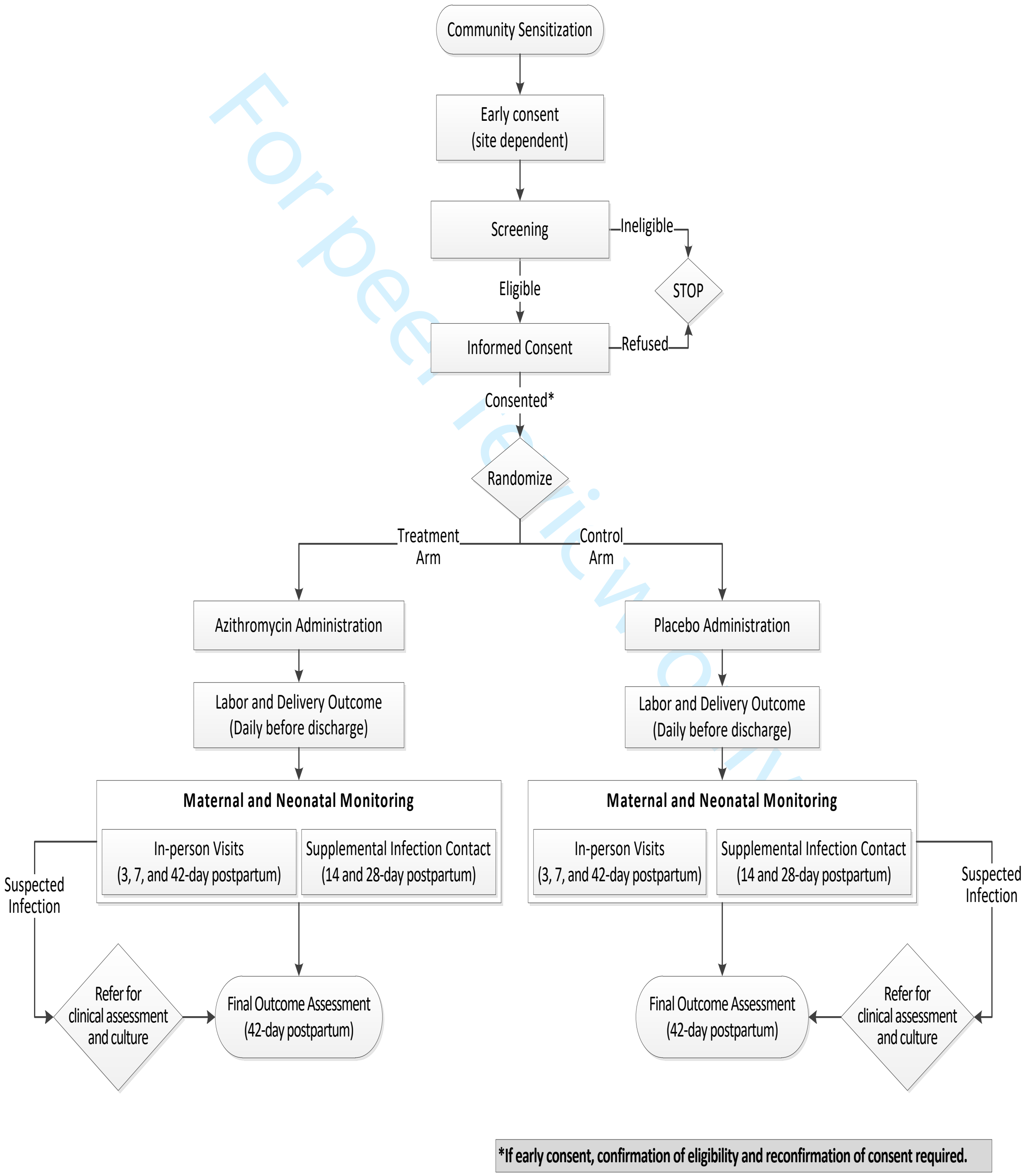

For peer review only - http://bmjopen.bmj.com/site/about/guidelines.xhtml

2.5 Primary Outcomes

The primary outcomes are:

- Maternal: Incidence of maternal death or sepsis within 6 weeks (42 days) post-delivery in intervention vs. placebo group.
- Neonatal: Incidence of intrapartum/neonatal death or sepsis within 4 weeks (28 days) post-delivery in intervention vs. placebo group.

Maternal sepsis is a life-threatening condition defined as organ dysfunction resulting from suspected or confirmed infection that occurs post-randomization during labor or the postpartum period. This WHO definition, endorsed by multiple global health organizations, will be operationalized as suspected or confirmed infection based on the presence of fever (>100.4° F/38° C) or hypothermia (<96.8° F/36° C) plus one or more signs of mild to moderate organ dysfunction including tachycardia (≥120 bpm), low BP (systolic <90 mm Hg), tachypnea (>24 breaths/min), altered mental status/confusion, reduced urinary output (<500 ml over 24 hours), jaundice, or renal failure (>1.2 mg/dl). [15,40,41,42] Components of peripartum infection which will be considered in making the diagnosis of suspected or confirmed infection include clinical chorioamnionitis, endometritis, wound infections (perineal or Cesarean), abdominal or pelvic abscess, mastitis/breast abscess or infection, pyelonephritis, pneumonia, and other bacterial infection as defined in Section 2.6 below.

Neonatal sepsis is defined as a newborn with proven or possible serious bacterial infection (pSBI) or pneumonia, or meningitis. Possible serious bacterial infection will be determined using WHO criteria of pSBI defined as severe chest in-drawing, fever (temperature ≥ 100.4° F/38.0 °C), hypothermia (temperature <95.9°F/35.5 °C), no movement at all or movement only on stimulation, feeding poorly or not feeding at all, and/or convulsions.[33] Clinical and laboratory signs of infection will also be considered in making the diagnosis of suspected or confirmed infection.

Centralized masked adjudication of key infection outcomes will be implemented by the DCC and investigators during the trial to standardize and enhance the reproducibility of trial results. In addition to the above specified criteria, reported antibiotic treatment and culture status will also be considered as part of the adjudication process in making the diagnoses for both maternal and neonatal sepsis. Local site investigators will review and validate the diagnosis for all participants identified to have the primary outcomes applying pre-specified criteria. These reviews will be conducted masked to treatment group. A second investigator from a different site will review the participant's information and make a final outcome determination. If this does not agree with the determination of the local site investigator, then the adjudicating investigator will discuss with the local investigator to reach a consensus, failing which the study PIs will make the final call. This process will be coordinated by the DCC.

The individual components of these primary outcomes are also of interest and will be analyzed.

2.6 Other Maternal Outcomes

a. Chorioamnionitis: Fever (>100.4°F/38°C) in addition to one or more of the following: fetal tachycardia ≥160 bpm, maternal tachycardia >100 bpm, tender uterus between contractions, or purulent/foul smelling discharge from uterus **prior** to delivery.
b. Endometritis: Fever (>100.4°F/38°C) in addition to one or more of maternal tachycardia >100 bpm, tender uterine fundus, or purulent/foul smelling discharge from uterus **after** delivery.

For peer review only - http://bmjopen.bmj.com/site/about/guidelines.xhtml

c. Other infections: Wound infection refers to purulent infection (superficial or deep infection including necrotizing fasciitis) of a perineal or Cesarean wound with or without fever. In the absence of purulence, a wound infection requires presence of fever (>100.4°F/38°C) and at least one of the following signs of local infection: pain or tenderness, swelling, heat, or redness around the incision/laceration; abdominopelvic abscess is evidence of pus in the abdomen or pelvis noted during open surgery, interventional aspiration or imaging; pneumonia refers to fever (>100.4°F/38°C) and clinical symptoms suggestive of lung infection including cough and/or tachypnea (>24 breaths/min) or radiological confirmation; pyelonephritis refers to fever (>100.4°F/38°C) and one or more of the following: urinalysis/dip suggestive of infection, costovertebral angle tenderness, or confirmatory urine culture; Mastitis/breast abscess or infection refers to fever (>100.4°F/38°C) and one or more of the following: breast pain, swelling, warmth, redness, or purulent drainage.
d. Use of subsequent maternal antibiotic therapy after randomization to 42 days postpartum for any reason.
e. Time from drug administration until initial discharge after delivery (time may vary by site).
f. Maternal readmission within 42 days of delivery.
g. Maternal admission to special care units.
h. Maternal unscheduled visit for care.
i. Maternal GI symptoms including nausea, vomiting, and diarrhea and other reported side effects.
j. Maternal death due to sepsis using the Global Network algorithm for cause of death.

2.7 Other Neonatal Outcomes

a. Other neonatal infections (e.g., eye infection, skin infection, omphalitis, urinary tract infection, respiratory rate ≥60 breaths/minute).
b. Neonatal initial hospital length of stay, defined as time of delivery until initial discharge (time may vary by site).
c. Neonatal readmission within 42 days of delivery.
d. Neonatal admission to special care units.
e. Neonatal unscheduled visit for care.
f. Neonatal death due to sepsis using the Global Network algorithm for causes of death.
g. Pyloric stenosis within 42 days of delivery, defined as clinical suspicion based on severe vomiting leading to death, surgical intervention (pyloromyotomy) as verified from medical records, or radiological confirmation.

2.8 Safety Monitoring

Surveillance of maternal side effects including nausea, vomiting, and diarrhea/loose stools, abdominal pain, vaginitis, and dizziness potentially associated with azithromycin will be conducted during labor and postpartum. For infants, findings suggestive of pyloric stenosis will be assessed during the follow up visits. Maternal and neonatal surveillance will also include assessment of unintended medical visits, maternal deaths, stillbirths, neonatal death within 28 days of birth, and infant death after 28 days of birth. Additional maternal and neonatal risks associated with azithromycin use include anaphylaxis, allergic reactions (rash), liver failure, and arrhythmias. Although rare, these side effects will be monitored and reported as a serious adverse event. All safety outcomes will be reviewed at least twice a year by the Data Monitoring Committee (DMC) appointed by NICHD.

For peer review only - http://bmjopen.bmj.com/site/about/guidelines.xhtml

2.9 Site Preparation

In preparation for study implementation, the site investigators will meet with local health authorities and conduct community sensitization activities to ensure that study procedures are appropriate for the local context and to encourage commitment and engagement at the facility and community level. Site preparation activities will focus on:

- Disseminating study objectives to local health authorities and other stakeholders
- Identifying and hiring study staff;
- Developing site-specific procedures for safety monitoring procedures;
- Exploring locally-acceptable methods to monitor and improve follow-up visit compliance;
- Identifying potential implementation challenges and developing culturally-appropriate solutions;
- Training research staff in the implementation of the study procedures, follow-up and ascertainment of infections.

2.10 Potential Risks and Benefits to Participants

There are several potential direct and indirect benefits of this trial. In developing countries, including those of GN partners, fetal and neonatal deaths due to infections and maternal and neonatal infections are common. If intrapartum azithromycin reduces maternal and fetal/neonatal mortality or infections, many deaths could be reduced in the GN sites as well as worldwide.

Emerging data suggest that intrapartum azithromycin reduces maternal and neonatal infection. It is not known if deaths could be prevented but as infections are one of the most frequent causes of maternal and neonatal deaths, there is a possibility that mortality could be reduced.

An ongoing concern for peripartum and perinatal antibiotic prophylaxis is the selection of resistant organisms including azithromycin-resistant organisms leading to resistant infections, and there is concern that disruption of gut and other flora (microbiome) in women and particularly in neonates may lead adverse events including increased allergic reactions, rash and childhood asthma.[12,18,40] In the trial from Gambia of a single oral dose of azithromycin during labor, higher prevalence of colonization with S. aureus azithromycin resistance observed among women and their babies four weeks after treatment had waned 12 months later and azithromycin did not induce other antibiotic resistance to S. pneumoniae or S. aureus; resistance data from actual infections were not available. There was a 7% vs. 21% prevalence of any bacteria in breast milk in those receiving azithromycin vs. placebo [4-5]. In the US trial of 2013 of adjunctive azithromycin in 2013 women who underwent unscheduled cesarean delivery, culture positive maternal infections (1.4% vs. 3.6%) and infections with at least one resistant bacterial species (1% vs. 2.4%) were significantly less frequent in the azithromycin group. Azithromycin-resistant organisms were identified in only 7 (3 vs. 4) participants. There is a paucity of data to address theoretical concerns that disturbances in the establishment of the indigenous intestinal microbiome caused by antibiotic exposure in early life or cesarean delivery, either directly or through modifications of breast microbiome, may increase risk of immune-mediated and inflammatory conditions such as atopic disorders, inflammatory bowel disease and obesity later in life.[27-30] While the potential for important benefits in both the mother and infant likely outweighs the likelihood and effects of antibiotic resistance and microbiome changes, these should be monitored. In addition to monitoring resistance patterns in isolates from clinical infections, antimicrobial and microbiome surveillance of a subset of enrolled participants are included in an ancillary protocol.

For peer review only - http://bmjopen.bmj.com/site/about/guidelines.xhtml

3 ANALYTICAL PLAN

Baseline demographic characteristics and key clinical measures will be compared between the women in the two treatment arms using contingency table approaches for categorical variables and analysis of variance models' continuous variables.

For summaries of study data, categorical measures will be summarized in tables listing the frequency and the percentage of participants; continuous data will be summarized by presenting mean, standard deviation, median and range; and ordinal data will be summarized by only presenting median and range. P-values presented will be based on two-sided tests unless otherwise specified and generally adjusted for randomization factor of site. For most analyses, the interaction between treatment and site will be assessed and if significant, results will also be presented by site. For continuous outcomes, distributional properties will be evaluated and if required, transformations or non-parametric tests will be employed. Additional details for potential covariate adjustments in secondary analyses or handling violations of analytic method assumptions will be detailed in the statistical analysis plan.

Three key populations are of interest for study analyses:

1. The Intention to Treat (ITT) population will include all women randomized and their infants. Analyses of this cohort will be conducted based on randomized treatment.
2. The High-Risk for Infection (HR) sub-group will include all women in the ITT and their infants meeting criteria for being high risk (i.e., prolonged labor [≥18 hours] and/or rupture of membranes [≥8 hours]) at the time of randomization. Analyses of this cohort will be conducted based on randomized treatment.
3. The As Treated population will include all randomized participants that receive any study drug during the study and their infants. Analyses of this cohort will be conducted based on treatment received.

The final determination of analysis population membership will be via a masked data review prior to final study analyses in order to address any potential anomalous cases that may arise in this large of a study population (e.g., randomization/treatment of a woman who is discharged prior to delivery due to false labor or unresponsiveness to induction).

3.1 Statistical Analysis Plan

3.1.1 Primary Analyses

Incidence of maternal death or sepsis and intrapartum/neonatal death or sepsis will be compared between the two treatment arms using generalized linear models. These generalized linear models will be fit with each binary outcome separately as the outcome measure. Estimates of relative risk and associated 95% confidence intervals will be reported. The model will include terms for treatment and site. As randomization occurs at the pregnancy level and approximately 1-2% of pregnancies are anticipated to be multiple gestations, models for neonatal outcomes will account for correlation among multiples assuming an exchangeable covariance structure. For the two primary outcomes, these analyses will be conducted using the ITT population and the p-values associated with the treatment term will be used to formally test each of the two primary hypotheses at the alpha = 0.05 level.

As secondary analyses of the primary outcomes, assuming an overall treatment effect is observed, the models will be run including region (Africa, Latin America, or Asia) and a treatment by region interaction

For peer review only - http://bmjopen.bmj.com/site/about/guidelines.xhtml

term. If the interaction term has a p<0.1, then effects will be reported by region with treatment effect within region tested at the 0.025 level.

Additional exploratory models will also be run including individually: 1) a treatment by site interaction term, 2) any other antibiotic use during labor (yes or no) and its interaction with treatment, and 3) mode of delivery (cesarean or vaginal) and its interaction with treatment. If the interaction term for any of these models has a p<0.1, then effects will also be reported by the relevant subgroups. These exploratory secondary models will also include any demographic or clinical variables found to differ significantly between the treatment arms in the preliminary analyses described above.

From each final model, estimates of relative risk associated with treatment will be obtained including unadjusted estimates of risk from the primary model as well as estimates of risk adjusted for potential confounders from the secondary analyses.

3.1.2 Secondary Analyses – Women at High Risk for Infection Cohort

The major secondary aim is assessing the two primary outcomes (i.e., incidence of maternal death or sepsis and incidence of intrapartum/neonatal death or sepsis) in the women at high risk for infection cohort. These analyses will also assess if the treatment effect differs between the HR cohort vs. non-high-risk women where non-high-risk women comprise all women and their infants in the ITT population that delivered prior to meeting criteria for high-risk (i.e. they delivered after < 18 hours of labor and < 8 hours post-rupture of membranes relative to time of randomization). Specifically, the model for the primary analysis of both the maternal and neonatal primary outcomes will be run including a treatment by risk status interaction term and excluding any data from individuals that meet high-risk criteria after randomization.

The exclusion of data from individuals meeting high risk criteria between randomization and delivery is intended to provide the most distinct groups regarding the HR risk cohort to non-high-risk women comparison. As this exclusion is based on a post-randomization event, sensitivity analyses will be conducted to examine any treatment group differences in meeting high risk criteria post randomization and if there is a difference, to determine the potential magnitude of impact this difference has on treatment effect. An exploratory analysis will also be completed that assesses if there is a difference in treatment effect between women randomized prior to high-risk classification vs. those randomized after high-risk classification. Finally, an exploratory analysis will be conducted that assesses the definition of high-risk to determine if there is a different cut-off that identifies a group of individuals with a greater treatment effect both in relation to randomization and delivery.

3.1.3 Secondary Analyses – Other Secondary Outcomes

Other maternal and neonatal binary outcomes including: the individual components of the primary outcomes, neonatal deaths due to sepsis, maternal infections (clinical chorioamnionitis, endometritis, wound infections (perineal or subsequent cesarean), pyelonephritis and pneumonia), use of subsequent maternal antibiotic therapy, pyloric stenosis and occurrences of maternal or neonatal readmission or admission into special care unit will be analyzed using the approaches detailed in **Section 3.1.1** for the ITT population and approaches detailed in **Section 3.1.2** for the HR cohort. A similar process with generalized linear models employing an appropriate link function will be used to analyze the outcomes of maternal and neonatal initial hospital length of stay.

For peer review only

For peer review only - http://bmjopen.bmj.com/site/about/guidelines.xhtml

Binary safety outcomes, e.g., nausea, vomiting, and diarrhea, will also be analyzed using the approaches detailed in **Section 3.1.1**. These analyses will be conducted using the As Treated population.

3.2 Sample Size

3.2.1 Sample Size for Primary Outcome

Sample size estimates were generated to evaluate the potential benefits of peripartum prophylactic azithromycin to reduce the risk of adverse maternal and neonatal outcomes in two population cohorts of women in low and low-middle income settings. The first population of interest comprises all women delivering in facilities (overall).

Power calculations for the study in the overall study population were generated for two primary outcome measures, one being the risk of maternal death or sepsis among women in the target population and the other being intrapartum /neonatal death or sepsis in infants delivered by women in the target population. For each of these outcome measures estimates of the required sample size needed to detect a risk reduction of 20%, 25%, and 30% were generated for power of 0.8, 0.85, and 0.9. The risk of sepsis or maternal death was assumed to be 3%. That number is slightly higher than the current risk in the GN population, which is slightly less than 2%. However, we anticipate that with active surveillance rather than passive reporting based on the new WHO definition of maternal sepsis (designed to catch more cases of sepsis), the risk will be at least 3%. For the neonatal outcome, the underlying risk of the combined outcome of intrapartum stillbirth, neonatal death, or sepsis was assumed to be between approximately 8% and 14%. This estimate was based on recent data from the GN indicating that the risk of intrapartum stillbirth is approximately 1.8% and the risk of neonatal death during the first 28 days after delivery is 2.3%; we assumed that the risk of sepsis not resulting in death is approximately 4% to 10%. The resulting required evaluable sample sizes are shown in **Table 1** below.

Table 1. Sample Sizes for the Overall Population, Alpha=0.05

		Evaluable Sample Size per Arm		
Baseline Risk	Risk Reduction	Power=0.80	Power=0.85	Power=0.90
3%	20%	11455	13103	15334
3%	25%	7133	8159	9548
3%	30%	4815	5508	6446
8%	20%	4096	4686	5483
8%	25%	2554	2921	3419
8%	30%	1727	1975	2311
14%	20%	2204	2521	2950
14%	25%	1377	1575	1842
14%	30%	932	1066	1247

The sample sizes shown above assume that the hypothesis test of interest is for the overall population and that the Type 1 error is controlled at the neonatal and maternal hypothesis level via testing each at an alpha = 0.05 level. Interest has been expressed for the overall study for being able to test the neonatal risk separately in south Asia and sub-Saharan Africa. Controlling the Type I error rate at the 0.025 level for each of those two areas within this sub-analysis of neonatal risk will be conducted to avoid multiple comparison concerns. The resulting sample sizes for that comparison are shown in Table 2 below. Note that this sample size would be the size required separately for the African and Asian sites.

For peer review only - http://bmjopen.bmj.com/site/about/guidelines.xhtml

It is planned that each site will enroll approximately equal number of participants. As such, approximately 37.5% of randomized mothers will be from sub-Saharan Africa and 50% will be from Asia. This planned enrollment distribution is approximately equivalent to the rates of in-facility deliveries observed in the GN registry database. Specifically, approximately a third of the facility deliveries are conducted in sub-Saharan Africa and between 45% and 50% of the facility deliveries are conducted in Asia. These estimates are also consistent with the enrollment rates for the ASPIRIN study that is currently being conducted at these sites. Therefore, to get the total study sample size required, the numbers in the table would need to be multiplied by 3 to achieve reasonable power for the African sites.

Table 2. Sample Sizes Within Region, Alpha=0.025

		Evaluable Sample Size per Arm		
Baseline risk	Risk Reduction	Power=0.80	Power=0.85	Power=0.90
8%	20%	4961	5607	6477
8%	25%	3093	3496	4038
8%	30%	2091	2363	2729
14%	20%	2669	3017	3485
14%	25%	1677	1884	2176
14%	30%	1129	1276	1474

Given the above information, we propose a sample size of 34,000 participants for the overall study. For the primary neonatal outcome of interest of intrapartum/neonatal sepsis or death, assuming that the loss to follow-up will be in the 2% to 3% range (consistent with the current ASPIRIN trial and the Global Network Maternal and Newborn Health registry), this sample size will be sufficient to provide 90% power to detect a 25% reduction in neonatal mortality and sepsis in the sub-Saharan African region and will provide 90% power to detect a 20% reduction in Asia assuming the baseline risk is at least 8%. For the primary maternal outcome of maternal death or sepsis, the sample size will provide 90% power to detect a 20% reduction from 3% in the population aggregated across all study sites.

3.2.2 Sample Size for High-Risk Women

The second population of interest comprises the cohort of high-risk population of women delivering in facilities with high risk being defined as term and preterm pregnant women who experience prolonged labor or prolonged membrane rupture. As a major secondary aim, sample size estimates for the high-risk cohort were also obtained to ensure adequate power for this analysis.

The primary objective of the high-risk component of the study is to test the effectiveness of a single oral dose of intrapartum azithromycin prophylaxis compared to placebo (all receive usual care) in reducing the risk of maternal sepsis or death in high-risk laboring women. To estimate the sample size required for this component of the study, we assumed conservatively that the underlying risk of the combined outcome in the target population is 6%. Comparable to the other study estimates of the required sample size needed to detect a risk reduction of 20%, 25%, and 30% were generated for power of 0.8, 0.85, and 0.9. Because the interest for this study is in testing the hypothesis overall rather than by region, the estimates were generated using an alpha of 0.05. The results of these calculations are shown in **Table 3** below.

For peer review only

For peer review only - http://bmjopen.bmj.com/site/about/guidelines.xhtml

Table 3. Sample Sizes for the High-Risk Cohort, Alpha=0.05

Baseline risk	Risk Reduction	Evaluable Sample Size per Arm		
		Power=0.80	Power=0.85	Power=0.90
6%	20%	5568	6369	7453
6%	25%	3470	3969	4644
6%	30%	2344	2681	3138

Because a risk reduction of at least 30% is expected in this population, we propose a sample size for this HR cohort of 5,500 women. That sample size will be sufficient to detect a 30% risk reduction with a power of 0.85 with an assumed 2% to 3% loss to follow-up. Assuming that 20% of the women are at high risk, the overall study sample size of 34,000 should allow for sufficient enrollment into the HR cohort.

If the proportion of "high-risk" women is smaller than anticipated, the target enrollment may be modified. A smaller sample size should still provide sufficient power for plausible potential underlying scenarios. For example, a sample size of approximately 4,000 high risk women will be sufficient to detect a 33% risk reduction with a power of 0.82 assuming a baseline risk of at least 6% as well as a 35% risk reduction with a power of 0.80 assuming a baseline risk of at least 5%.

3.3 Available Population

There are no competing protocols ongoing in the GN. Assuming conservatively that 50% of women in labor (30,000 facility births per year) meet eligibility criteria for entry and are enrolled in the trial, approximately 15,000 women/infant dyad will be enrolled per year.

To reach the enrollment target of 34,000, each study site will aim to recruit an equal number of study participants (n=4,250 per site); however, recruitment will be monitored and if a site does not meet targets, adjustments may be made. No site will be permitted to recruit more than 20% of the overall study sample site.

Based on historic GN data, we anticipate that the target of 5,500 high-risk women will be enrolled during the trial. However, through ongoing monitoring, the DCC will assess the number of women enrolled in the "high-risk" group and may modify target enrollment as needed to ensure at least 5,500 high-risk women are enrolled.

3.4 Projected Recruitment Time

The projected study timeline is 36 months or less. This includes the following:

- <u>0-6 months:</u> Finalize protocol, forms. Obtain approvals, train staff, and obtain/ship study drug/placebo to sites.
- <u>7-31 months:</u> Enroll participants (exact period of enrollment may vary by site)
- <u>32-36 months:</u> Complete follow-up; data cleaning and primary analyses.

3.5 Study Monitoring Plan

3.5.1 Reporting Serious Adverse Events

Serious Adverse events (SAEs) will be monitored continuously for any event that meets the following criteria:

- Results in neonatal/fetal or maternal death;
- Is life-threatening;

For peer review only - http://bmjopen.bmj.com/site/about/guidelines.xhtml

- Requires hospitalization or prolongs existing hospitalization;
- Results in persistent or significant disability or incapacity;
- Suspicion of pyloric stenosis within 42 days postpartum (prolonged vomiting leading to death or surgery)
- Any other serious or unexpected adverse event that the study investigator(s) feels should be reported.

3.5.2 Method and Timing for Reporting Serious Adverse Events

The Senior Foreign Investigator (SFI) must report the following SAEs by emailing or faxing a copy of the appropriate study form to RTI as follows:

<u>Within 48 hours of SFI's notification of the event:</u>

- All deaths (maternal, intrapartum stillbirths, neonatal)
- All SAEs with a definite or suspected/probable relationship to the intervention

<u>Within 7 days of SFI's notification of the event:</u>

- All life-threatening events;
- All SAEs considered to have a probable or possible relationship to the intervention;
- All emailed or faxed forms should also be entered into the DMS and transmitted within 7 days as a back-up to ensure no SAE is missed.

<u>Additional reporting procedures include:</u>

- RTI will forward all SAEs to the US-based Principal Investigator (PI) and NIH for further assessment of relationship to study intervention. The PI and SFI will be responsible for reporting to their respective IRB and other regulatory authorities per their institutional policy.
- RTI will be responsible for reporting SAEs to the DMC bi-annually at a minimum. The frequency of reporting to the DMC may be increased if the reported events or interim data reviews by the DMC indicate that more frequent safety monitoring is needed.
- Any SAE considered unrelated to the intervention is not required to be reported in an expedited manner. These events should be entered into the data management system and transmitted per routine procedures.

3.5.3 Data Monitoring Plan and Stopping Rules

All the Global Network sites will report data to the Global Network Data Coordinating Center, located at RTI International. The data will be used to evaluate protocol adherence and site performance (e.g., recruitment, loss to follow-up, data quality). The DCC will provide standardized progress reports to NICHD and the site investigators on a monthly basis to monitor outcome variables and adverse events.

Oversight of the trial will be handled by two principal groups with different focuses:

1. **<u>Protocol-focused Steering Committee (SC):</u>** The SC is comprised of the Central Study Team from the University of Alabama at Birmingham and the University of Zambia, NICHD, the DCC, and investigators from each of the participating sites (see Azithromycin Investigators on page 8). The Central Study Team, with assistance from NICHD and the DCC, will have primary responsibility for overall study design, development of study materials and procedures, and oversight of study implementation. They will meet via conference call bi-weekly to monitor study progress and ensure

For peer review only - http://bmjopen.bmj.com/site/about/guidelines.xhtml

proper implementation of the trial. The Site Investigators will be responsible for providing guidance on study design, developing site-specific implementation plans, ensuring study staff are properly trained, and providing oversight of the study at the site level. The SC will convene via conference call at least once per quarter and will meet in person twice a year to discuss study design and implementation issues. Members of the Central Study team, NICHD, and RTI will also conduct site visits, as the budget allows, to bolster enthusiasm, provide hands-on training and education to the participating staff, and address site-specific issues, if any.

2. **Data Monitoring Committee (DMC)**: The DMC, a standing group that monitors all NICHD-funded Global Network studies, will be responsible for ensuring safe and ethical treatment of study participants through monitoring of the study. The membership will include, at a minimum, a statistician, obstetrician, pediatrician, and an expert in international health. The DMC designated by NICHD will review the data collected at approximate 6-month intervals throughout the course of the study. The DMC reports, which are prepared by the Data coordinating center, will include information on study enrollment rates and participant progress through the study, participant compliance with protocol-specified treatment regimens, protocol violations, adverse events, and efficacy outcomes. The focus of the DMC review will be on monitoring participant safety and study progress/futility. Data on treatment effectiveness will also be presented to frame the DMC discussions on safety and futility. Additionally, one formal interim analyses of efficacy is planned as detailed in **Section 3.5.4**. The DMC will be charged with monitoring adverse events and side effects from azithromycin. All known associated side effects and specific obstetric, fetal, or neonatal concerns will be considered reportable to the DMC. The study will be reviewed by the DMC bi-annually at a minimum but may be reviewed more frequently if concerns are raised about participant safety or about adequate process of the study.

3.5.4 Interim/Adverse Event Monitoring Plan

The DMC designated by NICHD will complete safety reviews of the data bi-annually at minimum during the intervention phase or as often as they decide. Safety reports will be reviewed internally by the DCC quarterly and the DMC chair will be notified if any potential safety signals are identified to allow for more frequent DMC monitoring if needed. Adverse events will the reported and submitted to the DCC (and IRBs) who will report these cumulative masked data to the DMC in the safety reviews. The DMC recommendations about study continuation will be distributed to the IRBs. The DMC will forward their monitoring activities to the project officer representing the NICHD.

Additionally, one formal interim analysis of efficacy and futility will be conducted during the study. Interim analyses will be conducted in both all-comers and high-risk cohort assessing both the primary maternal and neonatal outcomes use the primary analytic approach detailed in **Section 3.1.1 and 3.1.2**. As the maternal and neonatal hypotheses are both of equal importance as well as the treatment effect within the two cohorts, the DMC will not be able to recommend early termination for efficacy unless significance is observed for both outcomes in all comers and the direction and magnitude of effect in the high-risk cohort is consistent with the effect observed in all comers. Cut-off p-values for testing for efficacy at this interim analysis will be determined based on a correction for multiple comparisons to ensure an overall alpha of 0.05. The futility assessment will be based on an analysis of conditional power. The details of the timing of the interim analysis as well as the approaches for correcting for multiplicity and analyzing conditional power will be determined in collaboration with the DMC prior to initiation of study enrollment. Safety and efficacy of the azithromycin will be reviewed and compared

For peer review only - http://bmjopen.bmj.com/site/about/guidelines.xhtml

with data from the placebo group according to a data center plan. The primary outcome rate will be calculated, and the data center will develop guidelines for interim analysis decision-making (e.g. the O'Brien-Fleming boundary table).

3.5.5 Risks/Benefits

Based on the preliminary data, there are few risks to this intervention especially given the single oral dose. Severe risks include anaphylaxis and allergic reactions (rash) and liver failure which are rare with azithromycin based on the product insert and our long history of use of prophylactic azithromycin at UAB since 2000. In 2013, the FDA issued an advisory regarding concerns about potential for rare life-threatening arrhythmias with azithromycin use particularly among those with preexisting cardiovascular risk. To minimize the risk of life-threatening side effects, those with arrythmia, known history of cardiomyopathy, or a known allergy to azithromycin will be excluded. With single 1-2 gram doses, gastrointestinal symptoms (nausea 5-18%, diarrhea/loose stools 7-14%, abdominal pain 5-7%, vomiting 2-7%, and dyspepsia 1%) as well as vaginitis 2% and dizziness (1%) were the most commonly reported side effects. These side effects will be monitored.

3.6 Quality Control

3.6.1 Training

All study personnel must participate in training on the proper implementation of study procedures and the ethics of conducting research with human subjects before beginning any research activity. The SFI and project coordinator will ensure that all study personnel receive the appropriate training and obtain the required certification. RTI will be responsible for developing a certification test. The SFI and project manager will be responsible for overseeing the certification process.

3.6.2 Study Monitoring

Major monitoring responsibilities of the PI/SFI, assisted by the country coordinator, are (1) confirming proper IRB approval; (2) monitoring delivery of the study intervention; (3) assessing and evaluating quality of study implementation; (4) ensuring compliance with the intervention, including proper randomization; (5) evaluating accuracy, precision, and completeness of data collected, entered, and transmitted, along with the DCC; (6) ensuring that all personnel are fulfilling their obligations; (7) maintaining staff morale and enthusiasm; (8) maintaining communication and handling ad hoc problems; (9) ensuring inter-site consistency; and (10) proposing improvements to monitoring activities.

NICHD and the DCC staff will conduct site visits as needed. These visits will include review of individual participant records, including supporting data, to ensure protection of study participants, compliance with the protocol, and accuracy and completeness of records. The SFI/PI will make study documents (e.g., logbooks, data forms, staff training certificates) and pertinent hospital/clinic records readily available for inspection by the local IRB, site monitors, and the NICHD for confirmation of the study data.

3.6.3 Drug Quality Assurance and Monitoring

The study drug manufacturer will have a Good Manufacturing Practices designation vetted by the FDA and a certificate of authenticity will be provided. Each site will adapt best practice guidelines for drug shipment and storage to the needs and infrastructure of their local environment. Study staff will be trained in on the drug shipment and storage plan to ensure that best practices are maintained at all times. Additionally, participants will receive detailed instruction on proper storage of the study drug at

For peer review only

For peer review only - http://bmjopen.bmj.com/site/about/guidelines.xhtml

home. Drug stability information will be maintained throughout the study. For quality assurance, a sample of pills from each site will be randomly selected and tested for bioavailability at multiple time points during the study period. A sample from each batch will be tested.

3.6.4 Plan for Sustaining Intervention

We will plan to present abstracts to reputable international obstetric meetings (e.g. SMFM, ACOG or FIGO) and manuscripts within 3-6 months of completion of the primary data collection to high impact journals such as the New England Journal of Medicine, JAMA, or the Lancet. If the results are positive, we will facilitate the change in practice at participating sites and also approach the WHO to instigate guideline updates to reflect the study findings.

4 Data Management Procedures

Data will be collected both prospectively and from existing medical records, using hard copy forms or Android Tablets. Regardless of data capture methodology, all data will be kept confidential. Each participant will be assigned a unique study ID which will be used to identify the participant. Only the screening log will contain the name (which is not transmitted). If hard copy forms are used, they will be retained in a secure location for possible editing or queries at the central data entry site. Data will be entered into computers using the Data Management System (DMS) developed by RTI and the assigned study number. The DMS will also allow site staff to produce project reports and backup the study database. Electronic data will be transferred from each data management computer to a single Research Unit Data Center in each country, creating a complete data repository. At least once a week, data will be transmitted from the Research Unit Data Center to the DCC at RTI, where the central database will be located. The DCC will conduct training on data collection procedures and the DMS system, as needed.

Precision and accuracy of actual data collected will be checked by chart review (random 5%) and internal procedures using the computer program. Monthly audits and incomplete data reports will be performed by a review team consisting of at least the SFI and country coordinator. Data editing and error resolution will be performed monthly. In addition, a sample of participants will be visited to confirm participation, with procedures determined per site. These activities will be shared between the site and the DCC. The timing of data collection is found in the schedule of study procedures (**Appendix 3).**

4.1 Data Forms

The following forms will be used for this study:

Form Name	Purpose	Key Data Elements	Data Source
**Contact and Scheduling Form**	To facilitate participant follow-up by documenting contact information and projected study visit schedule	Contact information, date of enrollment, date of follow-up visits	Participant interview, study calendar
**Screening and Enrollment Form**	To determine eligibility and record consent status	Screening date, review and confirmation of inclusion/exclusion criteria, consent status/date.	Participant interview/report, clinical assessment, provider report, medical records
**Randomization Form**	To confirm eligibility and track randomization	Eligibility confirmation, randomization date/time, drug administration date/time, any problems with drug administration (e.g., vomiting, dropped med, etc).	Study records, participant interview and observation

For peer review only - http://bmjopen.bmj.com/site/about/guidelines.xhtml

Form Name	Purpose	Key Data Elements	Data Source
**Maternal Baseline Data Form**	To collect additional maternal information	• Demographic and baseline clinical data: admission date/time, EDD, GA, age, height, weight, pregnancy and medical history, etc.<br>• Events during labor and delivery: timing of onset of labor, prolonged labor, premature rupture of membranes, date/time/type of membrane rupture, date/time/type of delivery, indication for induction (if applicable), vital signs during labor (temp, HR, RR, BP), complications, etc.<br>• Events after delivery: complications, vital signs, etc.<br>• Infection assessment pre- and post-delivery<br>• Antibiotic treatment pre- and post-delivery<br>• Discharge information	Participant interview/report, provider report, medical records
**Neonatal Baseline Data Form**	To collect information about the infant directly after delivery.	• Sex<br>• Birthweight<br>• Delivery outcome (live or stillbirth)<br>• Complications<br>• Infection assessment<br>• Discharge information: date/time, status (discharged, transferred, died)	Participant interview/report, provider report, medical records
**Maternal and Neonatal Follow-up Form**	To collect maternal and infant health status during follow-up visits at 3 days, 7 days and 42 days after delivery.	• Timing follow-up<br>• Maternal and infant status since discharge: general status, symptoms, indication of infection, clinic visits, hospitalizations, antibiotic use<br>• Temperature	In-person participant interview/report and clinical exam
**Supplemental Infection Contact Form**	To document signs of maternal or neonatal infection and provide referrals if indicated.	• Maternal and neonatal infection danger signs.<br>• Referral information.	Participant interview/report by phone
**Maternal and Neonatal Unscheduled Medical Visit**	To collect information about clinical events reported during follow-up visits	Reason for medical visit, details about medical visit	Participant interview/report, provider report, clinical exam, medical records
**Specimen Collection and Result Form**	To track specimen collection when there is indication of maternal or infant infection	Date/time of specimen collection; reason for collection; location of wound/infection; tracking information for shipping/storing specimen; results	Study documentation if collected and/or tested by study; medical records if collected and/or tested by health facility.
**Serious Adverse Events**	To record fatal, life-threatening, or any other serious, unexpected adverse event	Date/time of event, date/time of resolution, nature of adverse event, management of adverse event, attribution to study (yes/no).	Participant interview/report, provider report, medical records
**Final Study Status**	To document final study status	Final study status (e.g., completion, withdrawal, lost-to-follow-up), Date of final study status. If withdrawal, provide reason.	Participant report, medical records
**Protocol Deviation**	To record protocol deviations and corrective actions	Date and nature of deviation/violation, corrective action.	Participant report, study records, medical records
**Outcome Adjudication**	To validate cases of infection using standard study definitions	Primary and secondary outcomes	Study documentation, provider reports/interviews, medical records

For peer review only - http://bmjopen.bmj.com/site/about/guidelines.xhtml

5 References

1. World Health Organization. (2015). WHO recommendations for the prevention and treatment of maternal peripartum infections. Retrieved August 22, 2018, from http://www.who.int/reproductivehealth/publications/maternal_perinatal_health/peripartum-infections-guidelines
2. Liu, L., Oza, S., Hogan, D., Perin, J., Rudan, I., Lawn, J. E., . . . Black, R. E. (2015). Global, regional, and national causes of child mortality in 2000-13, with projections to inform post-2015 priorities: an updated systematic review. *The Lancet, 385*(9966), 430-440.
3. Tshefu, A., Lokangaka, A., Ngaima, S., Engmann, C., Esamai, F., Gisore, P., . . . African Neonatal Sepsis Trial (AFRINEST) group. (2015). Simplified antibiotic regimens compared with injectable procaine benzylpenicillin plus gentamicin for treatment of neonates and young infants with clinical signs of possible serious bacterial infection when referral is not possible: a randomised, open-label, equivalence trial. *Lancet, 385*(9979), 1767-1776.
4. Baqui, A. H., Saha, S. K., Ahmed, A. S., Shahidullah, M., Quasem, I., Roth, D. E., . . . Projahnmo Study Group in Bangladesh. (2015). Safety and efficacy of alternative antibiotic regimens compared with 7 day injectable procaine benzylpenicillin and gentamicin for outpatient treatment of neonates and young infants with clinical signs of severe infection when referral is not possible: a randomised, open-label, equivalence trial. *Lancet Global Health, 3*(5), e279-287.
5. Mir, F., Nisar, I., Tikmani, S. S., Baloch, B., Shakoor, S., Jehan, F., . . . Zaidi, A. K. (2017). Simplified antibiotic regimens for treatment of clinical severe infection in the outpatient setting when referral is not possible for young infants in Pakistan (Simplified Antibiotic Therapy Trial [SATT]): a randomised, open-label, equivalence trial. *Lancet Global Health, 5*(2), e177-e185.
6. Zaidi, A. K., Tikmani, S. S., Sultana, S., Baloch, B., Kazi, M., Rehman, H., . . . Cousens, S. (2013). Simplified antibiotic regimens for the management of clinically diagnosed severe infections in newborns and young infants in first-level facilities in Karachi, Pakistan: study design for an outpatient randomized controlled equivalence trial. *Pediatric Infectious Disease Journal, 32 Suppl 1*(Suppl 1), S19-25. https://doi.org/10.1097/INF.0b013e31829ff7aa
7. Gibbs, R. S. (1980). Clinical risk factors for puerperal infection. *Obstetrics and Gynecology, 55*(5 Suppl), 178S-184S.
8. World Health Organization. (2015). WHO Statement on Caesarean Section Rates. Retrieved August 22, 2018, from http://www.who.int/reproductivehealth/publications/maternal_perinatal_health/cs-statement/en/
9. Mackeen, A. D., Packard, R. E., Ota, E., Berghella, V., & Baxter, J. K. (2014). Timing of intravenous prophylactic antibiotics for preventing postpartum infectious morbidity in women undergoing cesarean delivery. *Cochrane Database of Systematic Reviews, 12*(12), CD009516. https://doi.org/10.1002/14651858.CD009516.pub2
10. Smaill, F. M., & Grivell, R. M. (2014). Antibiotic prophylaxis versus no prophylaxis for preventing infection after cesarean section. *Cochrane Database of Systematic Reviews, 10*, CD007482. https://doi.org/10.1002/14651858.CD007482.pub3
11. Gyte, G. M. L., Dou, L. X., & Vazquez, J. C. (2014). Different classes of antibiotics given to women routinely for preventing infection at caesarean section. *Cochrane Database of Systematic Reviews, 11*(11). https://doi.org/10.1002/14651858.CD008726.pub2
12. Tita, A. T. N., Rouse, D. J., Blackwell, S., Saade, G. R., Spong, C. Y., & Andrews, W. W. (2009). Evolving concepts in antibiotic prophylaxis for cesarean delivery: a systematic review. *Obstetrics and Gynecology, 133*(3), 675-682.
13. Tita ATN, S. J., Boggess K, et al. . (2016). Adjunctive azithromycin prophylaxis for cesarean delivery. *New England Journal of Medicine, 375*, 1231-1241.
14. Oluwalana, C., Camara, B., Bottomley, C., Goodier, S., Bojang, A., Kampmann, B., . . . Roca, A. (2017). Azithromycin in labor lowers clinical infections in mothers and newborns: a double-blind trial. *Pediatrics, 139*(2). https://doi.org/10.1542/peds.2016-2281

For peer review only - http://bmjopen.bmj.com/site/about/guidelines.xhtml

15. World Health Organization. (2017). Statement on maternal sepsis. Retrieved Ausust 22, 2018, from http://www.who.int/reproductivehealth/publications/maternal_perinatal_health/maternalsepsis-statement/en/
16. Reinhart K, D. R., Kissoon N, Machado FR, Schachter RD, Finfer S. . (2017). Recognizing sepsis as a global health priority - a WHO resolution. *New England Journal of Medicine, 377*(5), 414-417.
17. van Dillen, J., Zwart, J., Schutte, J., & van Roosmalen, J. (2010). Maternal sepsis: epidemiology, etiology and outcome. *Current Opinion in Infectious Diseases, 23*(3), 249-254. https://doi.org/10.1097/QCO.0b013e328339257c
18. Roca A, O. C., Bojang A, Camara B, Kampmann B, Bailey R, Demba A, Bottomley C, D'Alessandro U. (2016). Oral azithromycin given during labour decreases bacterial carriage in the mothers and their offspring: a double-blind randomized trial. *Clin Microbiol Infect. , 22*(6), e1-9.
19. Subramaniam A, Ye Y, Mbah R, Mbunwe DM, Pekwarake S, Bunwi EY, Fondzeyuf A, Ngong MG, Dionne-Odom J, Harper LM, Jauk VC, Carlo WA, Halle-Ekane G, Szychowski JM, Tih P, Tita AT. Single Dose of Oral Azithromycin With or Without Amoxicillin to Prevent Peripartum Infection in Laboring, High-Risk Women in Cameroon: A Randomized Controlled Trial. Obstet Gynecol. 2021;138(5):703-713. PMID: 34619734.
20. Harper, L. M., Kilgore, M., Szychowski, J. M., Andrews, W. W., & Tita, A. T. N. (2017). Economic evaluation of adjunctive azithromycin prophylaxis for cesarean delivery. *Obstetrics and Gynecology, 130*(2), 328-334. https://doi.org/10.1097/AOG.0000000000002129
21. Andrews, W. W., Hauth, J. C., Cliver, S. P., Savage, K., & Goldenberg, R. L. (2003). Randomized clinical trial of extended spectrum antibiotic prophylaxis with coverage for Ureaplasma urealyticum to reduce post-cesarean delivery endometritis. *Obstetrics and Gynecology, 101*(6), 1183-1189.
22. Tita AT, H. J., Grimes A, Owen J, Stamm AM, Andrews WW. . (2008). Decreasing incidence of post-cesarean endometritis with extended-spectrum antibiotic prophylaxis. *Obstetrics and Gynecology, 111*(1), 51-56.
23. Tita, A. T., Owen, J., Stamm, A., Hauth, J. C., & Andrews, W. W. (2008). Impact of extended-spectrum antibiotic prophylaxis on incidence of post-cesarean surgical wound infection. *American Journal of Obstetrics and Gynecology, 199*(3), 303.
24. Watts, D. H., Eschenbach, D. A., & Kenny, G. E. (1989). Early postpartum endometritis: the role of bacteria, genital mycoplasmas, and Chlamydia trachomatis. *Obstetrics and Gynecology, 73*(1), 52-60.
25. Hoyme, U. B., Kiviat, N., & Eschenbach, D. A. (1986). Microbiology and treatment of late postpartum endometritis. *Obstetrics and Gynecology, 68*(2), 226-232.
26. Emmons, S. L., Krohn, M., Jackson, M., & Eschenbach, D. A. (1988). Development of wound infections among women undergoing cesarean-section. *Obstetrics and Gynecology, 72*(4), 559-564.
27. Roberts, S., Maccato, M., Faro, S., & Pinell, P. (1993). The microbiology of post-cesarean wound morbidity. *Obstetrics and Gynecology, 81*(3), 383-386.
28. Rosene, K., Eschenbach, D. A., Tompkins, L. S., Kenny, G. E., & Watkins, H. (1986). Polymicrobial early postpartum endometritis with facultative and anaerobic bacteria, genital mycoplasmas, and Chlamydia trachomatis: treatment with piperacillin or cefoxitin. *Journal of Infectious Diseases, 153*(6), 1028-1037.
29. Andrews, W. W., Shah, S. R., Goldenberg, R. L., Cliver, S. P., Hauth, J. C., & Cassell, G. H. (1995). Association of post-cesarean delivery endometritis with colonization of the chorioamnion by Ureaplasma urealyticum. *Obstetrics and Gynecology, 85*(4), 509-514. https://doi.org/10.1016/0029-7844(94)00436-H
30. Keski-Nisula, L., Kirkinen, P., Katila, M. L., Ollikainen, M., Suonio, S., & Saarikoski, S. (1997). Amniotic fluid U. urealyticum colonization: significance for maternal peripartal infections at term. *American Journal of Perinatology, 14*(3), 151-156.
31. Yoon, B. H., Romero, R., Park, J. S., Chang, J. W., Kim, Y. A., Kim, J. C., & Kim, K. S. (1998). Microbial invasion of the amniotic cavity with Ureaplasma urealyticum is associated with a robust host response in fetal, amniotic, and maternal compartments. *American Journal of Obstetrics and Gynecology, 179*(5), 1254-1260. https://doi.org/Doi 10.1016/S0002-9378(98)70142-5
32. Ledger, W. J. (2006). Prophylactic antibiotics in obstetrics-gynecology: a current asset, a future liability? *Expert Review of Anti-Infective Therapy, 4*(6), 957-964. https://doi.org/10.1586/14787210.4.6.957
33. World Health Organization. (2015). Guideline: Managing Possible Serious Bacterial Infection in Young Infants When Referral is not Feasible. Retrieved August 22, 2018, from

For peer review only - http://bmjopen.bmj.com/site/about/guidelines.xhtml

http://apps.who.int/iris/bitstream/handle/10665/181426/9789241509268_eng.pdf;jsessionid=F74B4376
89D1CB960E2544DDF53FE5A6?sequence=1

34. Sutton, A. L., Acosta, E. P., Larson, K. B., Kerstner-Wood, C. D., Tita, A. T., & Biggio, J. R. (2015). Perinatal pharmacokinetics of azithromycin for cesarean prophylaxis. *American Journal of Obstetrics and Gynecology, 212*(6), 812.

35. Eberly, M. E., Eide, M. B., Thompson, J. L., & Nylund, C. M. (2015). Azithromycin in Early Infancy and Pyloric Stenosis. *Pediatrics, 135*(3), 483. https://doi.org/10.1542/peds.2014-2026

36. Ray, W. A., Murray, K. T., Hall, K., Arbogast, P. G., & Stein, C. M. (2012). Azithromycin and the risk of cardiovascular death. *New England Journal of Medicine, 366*(20), 1881-1890.

37. Svanström, H., Pasternak, B., & Hviid, A. (2013). Use of azithromycin and death from cardiovascular causes. *New England Journal of Medicine, 368*(18), 1704-1701. https://doi.org/10.1056/NEJMoa1300799

38. Hoffman, M. K., Goudar, S. S., Kodkany, B. S., Goco, N., Koso-Thomas, M., Miodovnik, M., . . . Derman, R. J. (2017). A description of the methods of the aspirin supplementation for pregnancy indicated risk reduction in nulliparas (ASPIRIN) study. *BMC Pregnancy and Childbirth, 17*(1), 135. https://doi.org/10.1186/s12884-017-1312-x

39. Goldenberg, R. L., Saleem, S., Ali, S., Moore, J. L., Lokangako, A., Tshefu, A., . . . McClure, E. M. (2017). Maternal near miss in low-resource areas. *International Journal of Gynaecology and Obstetrics, 138*(3), 347-355. https://doi.org/10.1002/ijgo.12219

40. Bonet, M., Souza, J. P., Abalos, E., Fawole, B., Knight, M., Kouanda, S., . . . Metin Gulmezoglu, A. (2018). The global maternal sepsis study and awareness campaign (GLOSS): study protocol. *Reprod Health, 15*(1), 16. https://doi.org/10.1186/s12978-017-0437-8

41. Albright, C.M., Has, P., Rouse D.J., Hughes B.L. (2018). Internal Validation of the Sepsis in Obstetrics Score to Identify Risk of Morbidity from Sepsis in Pregnancy. Obstetric Anesthesia Digest, 38 (1). https://doi.org/10.1097/01.aoa.0000529967.01647.fb.

42. Bowyer L., Robinson H.L., Barrett H., Crozier T.M., Giles M., Idel I., Lowe S., Lust K., Marnoch C.A., Morton M.R., et al. SOMANZ guidelines for the investigation and management sepsis in pregnancy. Aust. N. Z. J. Obstet. Gynaecol. 2017;57:540–551. doi: 10.1111/ajo.12646.

For peer review only

For peer review only - http://bmjopen.bmj.com/site/about/guidelines.xhtml

Appendix 1. Description of Participating Global Network Sites

The Global Network for Women's and Children's Health Research (GN) was created as a private-public partnership between the U.S. National Institutes of Health (NIH) and the Bill and Melinda Gates Foundation in response to the alarming rates of morbidity and mortality in women and children and the lack of research expertise and infrastructure in the developing world. Its mission is to expand scientific knowledge, develop research infrastructures, and improve health outcomes by building research partnerships to conduct research on feasible, cost-effective, sustainable interventions to address the major causes of perinatal morbidity and mortality of women and children in the developing world. It is currently funded only by the Eunice Kennedy Shriver National Institute of Child Health and Human Development (NICHD).

The current configuration of the GN is comprised of eight multidisciplinary research sites in seven developing countries (Bangladesh, Democratic Republic of Congo, Guatemala, India, Kenya, Pakistan, and Zambia), each with an established collaboration between an institution in the U.S. and one or more in the developing country. Each site has a U.S.-based senior principal investigator (PI) and a senior foreign investigator (SFI) based in the developing world, who lead a team of in-country research staff.

In 2005, the GN implemented its first multicounty protocol, the First Breath (FB) study, a community-based cluster trial to determine whether education and training in the American Academy of Pediatrics Neonatal Resuscitation Program (NRP) and the Essential Newborn Care Program (ENC) of the World Health Organization (WHO) reduced neonatal mortality (7 days) more than education and training in ENC alone. More than 3,700 birth attendants from 100 GN communities with > 150,000 deliveries were taught the appropriate procedures and provided with the necessary equipment to resuscitate infants at birth. Communities were randomized to ENC plus NRP or continued ENC.

The GN has continued to build on the capacity developed in the FB trial through the implementation of more than 10 additional multicounty protocols to address priority research needs to improve maternal and child health in low-resource settings. Current projects include:

- **The Maternal Newborn Health Registry** is a prospective, population-based study of pregnancies and their outcomes in low-middle income countries (DRC, Guatemala, India, Pakistan, Bangladesh, Zambia and Kenya). All pregnant women in participating clusters are registered and their outcomes tracked for 6 weeks post-delivery. The primary purpose of this prospective, population-based observational study of approximately 60,000 women per year is to quantify and understand the trends in pregnancy services and outcomes over time in defined, low-resource geographic clusters. The goal is to provide population-based statistics on stillbirths, neonatal and maternal mortality as the basis of health care policy. The data from the registry also provide the mortality and morbidity outcomes for Global Network trials and help investigators plan future studies for the Global Network.
- Preterm birth remains the leading cause of neonatal mortality and long-term disability throughout the developed and developing world. A growing body of evidence suggests that 1st

For peer review only - http://bmjopen.bmj.com/site/about/guidelines.xhtml

trimester administration of low dose aspirin can reduce the rate of PTB substantially. The **ASPIRIN Study** is a prospective, randomized, placebo-controlled, double-masked, multi-center clinical trial to examine whether low dose aspirin initiated between 6 0/7 weeks- 12 6/7 weeks gestation reduces the risk of preterm birth. The study has enrolled 11,920 women across seven sites in Africa, Asia, and Latin America.

- Attention is increasingly directed to the role of maternal nutrition during the 1st trimester for normal growth and development during the first thousand days, from conception to the child's second birthday. The primary hypothesis of the **Women First: Preconception Maternal Nutrition study** is that for women in poor communities, a comprehensive maternal nutrition intervention commencing at least 3 months prior to conception and continuing throughout pregnancy, will be associated with a significantly greater newborn length than for offspring whose mothers start to receive the same intervention at 12 weeks gestation or who do not receive the intervention at all. The results of this trial will make a major contribution to refining evidence-based strategies for maternal nutrition supplementation and evaluating the cost-benefits of extending such strategies beyond pregnancy to virtually all women of child-bearing age, including adolescent girls.

For peer review only

For peer review only - http://bmjopen.bmj.com/site/about/guidelines.xhtml

APPENDIX 2. SAMPLE INFORMED CONSENT

Global Network for Women's & Children's Health Research

Single oral dose of azithromycin 2 gm in laboring women to prevent neonatal infection/death and maternal peripartum infection/death

INVESTIGATORS:
[LIST SITE INVESTIGATORS]

SPONSOR:
The Eunice Kennedy Shriver National Institutes of Child Health and Human Development (NICHD)

You are being asked to participate in a research study for pregnant mothers. This study is funded by the U.S. National Institutes of Health and the Bill and Melinda Gates Foundation. This form provides you with information about the study so that you can decide whether you would like to participate. A member of the research team will describe the study to you and answer all of your questions. Please read the information below and ask questions about anything you don't understand before deciding whether or not to take part. You may also request that the research staff read the form to you.

What is the purpose of the study?
The purpose of this study is to learn whether an antibiotic, called azithromycin, given orally (by mouth) to pregnant women during labor can reduce the risk of infection for the woman and her baby.

Who will be in the study?
A total of 34,000 women will be enrolled in this study from eight sites in sub-Saharan Africa, South Asia, and Latin America. If needed, up to 2000 additional women across all sites who meet high-risk criteria will be enrolled (defined as 8 hours or more of membrane rupture before delivery or 18 hours or more of labor). In [insert site name], no more than [insert max sample size] will be enrolled.

You qualify for this study if you are a pregnant woman of the legal age of consent who is in labor with one or more live fetuses and a pregnancy ≥28 weeks, plan to deliver vaginally in a health facility, have no known infections that require antibiotics, have no known problems taking azithromycin or similar antibiotics (such as amoxicillin), and have not used azithromycin, erythromycin, or similar antibiotic in the past 3 days. We will ask you some questions about your pregnancy and health status to make sure you qualify to participate.

What will happen if I join this study?
Before participating, you will be provided with information about the study procedures and given an opportunity to ask questions. If you qualify and agree to participate, you will be asked to sign this form to indicate your consent.

If you agree to participate, you will be assigned to either the treatment group or the control group. The assignment is made randomly, like flipping a coin or choosing a grain of rice from a bag. The women in the treatment group will take four 500 mg azithromycin pills. For comparison, the women in the control group will take four pills that look identical to azithromycin but do not contain any medication. This will

For peer review only - http://bmjopen.bmj.com/site/about/guidelines.xhtml

allow the researchers to compare how well azithromycin works to prevent infection in mothers and their babies. Neither you nor the study staff will know whether you are assigned to the treatment or control group.

After you have been assigned to a treatment group, you will be given 4 small 500 mg pills to take by mouth. A member of the study team will watch you take the pills. After you take the pills, you will receive care during your labor, delivery, and recovery from the health facility staff, according to the local standard of care. It will take approximately 30 minutes to complete the consent form and take the pills.

While you are at the health facility to deliver your baby, the study team will also collect:

- Information about your health status during labor, at the time of delivery, and after delivery. This includes collecting your temperature and vital signs, such as heartrate and blood pressure;
- Information about your baby's health status during and after delivery. This includes collecting your baby's temperature and vital signs, such as heart rate and blood pressure;
- If you or your baby develop an infection before you are discharged from the health facility, a specimen will be collected to help identify the bacteria that is causing the infection. This may be blood sample, a urine sample, or a sample taken from the site of the infection (for example, pus from a wound or breast milk in the case of a breast infection).

It will take no more than 30 minutes to collect health information about you and your baby. In the case of suspected infection, it may take an additional 30 minutes to collect samples from you or your baby.

After delivery, a member of the study team will visit you a total of three times to assess the health status of you and your baby. These visits will be scheduled to take place at your home or a health facility at 3 days, 1 week (7 days) and 6 weeks (42 days) after delivery. If you or your baby are hospitalized at the scheduled time, the study team will visit you in the hospital. During the visit, the study team will assess you and your baby for current or past signs of infection. This will include collecting health information and measuring temperature for you and your baby. Each visit will take approximately 30 minutes.

A member of the study team will also contact you by phone at 14 days and 28 days after delivery to review the signs and symptoms of infection. If you do not have a phone, the study team will arrange to visit you at home or at the study facility. Each phone contact will take approximately 15 minutes.

If a sign of infection is identified in you or your baby during the in-person visits or phone contact, you will be referred a health facility for further assessment. You will also be asked to provide a sample, such as blood, pus, or urine, to help identify the bacteria causing the infection. The samples will be labelled with a study identification number in place of your name or your baby's name. Only dedicated members of our study team will have access to the samples.

To ensure that we have accurate and complete information about the health of you and your baby, we will access and collect information from the medical records at the health facilities where you and your baby have received care. By agreeing to participate in this study, you are also agreeing to give permission for the study staff to access your medical records. We will take precautions to protect the information that is collected from your medical records. Only study staff will have access to this information. To further protect you and your baby, all of your information will be coded with a number in place of your name.

For peer review only

The local research staff have been selected because of their skills, knowledge, and familiarity with your community. The research staff are here to support you during the study and should be contacted between visits if you have any questions or concerns.

What are the risks and discomforts

Azithromycin is a sometimes used to treat infections in pregnant women and children. Research shows that the risks of taking azithromycin are minimal. A commonly reported side effect is gastric discomfort (nausea, stomach pain, diarrhea, vomiting); however, you will be given pills with a special coating which will help prevent stomach discomfort. There is also a small but rare risk that azithromycin could cause arrythmia (irregular or abnormal heartbeat) or an allergic reaction; therefore, you will not be able to participate if you have a known history of heart problems or have had a bad reaction to azithromycin or a similar drug in the past.

If it is necessary to take a sample because of infection, you or your baby may feel temporary discomfort, but this will only last a few seconds. To minimize this, we will ensure research staff are well trained in the procedure.

Another possible risk of participating in this study is that your name and personal information may be seen by persons who are not part of the project. To prevent this, you will be given an identification number that will be used in place of your name on all study documents.

Information from this research study will be retained by [local institution] and RTI International in the United States (U.S.) and in the future may be included in a de-identified public use database managed by NICHD Data and Specimen Hub (DASH) in compliance with the U.S. National Institutes of Health (NIH) Public Access Policy. De-identified means that you and your baby will not be individually identified by name or other personal identifiers in the database. Your full name or any address details will not be included. Information released will not identify you or your baby's participation in this research study.

What are the benefits of participating?

You will not receive any money from participating in this study, but your participation may provide important information that can be used in the future to prevent infection in mothers and babies. Also, there is preliminary information suggesting that the use of azithromycin in pregnancy can reduce the risk of maternal and infant infection.

If new information about the benefits or risks of azithromycin use in pregnancy becomes available during the study, this information will be given to you by [Insert name of Senior Investigator] or his/her staff.

Will I have to pay for anything?

It will not cost you anything to be in the study.

Is my participation voluntary?

Taking part in this study is voluntary. You have the right to refuse to participate or to withdraw your participation at any time. If you refuse or decide to withdraw, you will not lose any benefits or rights to which you are entitled. These actions will not have any negative effect on the health care you receive from your local health providers. You will still receive your normal medical care.

Can I be removed from this study?

For peer review only - http://bmjopen.bmj.com/site/about/guidelines.xhtml

You will be withdrawn from the study if the research staff thinks that your participation may cause harm to you or your baby. The research staff may also remove you from the study for other reasons at their discretion. Also, the sponsor may stop the study at any time.

What will happen if you are injured by this research?

Although the risk of injury is expected to be very low, all research involves a chance that something bad might happen to you. Despite all safety measures, your participation could result in a reaction or injury. If you or your infant is injured as a result of your participation, you will be provided with emergency care by the study and referred to a doctor for ongoing care, if needed. Ongoing care will not be paid for by the study. [Insert name of Research Institution] and NICHD have not set aside funds to pay you for any such reactions, injuries or related medical care. However, by signing this form, you do not give up any of your legal rights.

What should you do if you have additional questions?

If you have questions about this study or a project-related injury, you should contact [investigator contact]. If you have questions about your or your baby's rights as a project participant, please contact [insert ethics committee contact].

Agreement to be in this study

I have read this paper about the study or it was read to me. I understand the possible risks and benefits of this study. I know that being in this study is voluntary and I choose to be in this study. I understand I will get a copy of this consent form.

Signature (or thumbprint):_______________________________________ Date:_______________

(Mother)

Print Name:____

(Mother)

Signature (or thumbprint):_______________________________________ Date:_______________

(Parent/Guardian/Husband)

Print Name:____

(Parent/Guardian/Husband)

Future Contact

We may wish to contact you in the future about participating in other related research studies. Please indicate below if you are willing to be contacted.

☐ Yes, I agree to be contacted

☐ No, I do not agree to be contacted

For peer review only - http://bmjopen.bmj.com/site/about/guidelines.xhtml

APPENDIX 3. SCHEDULE OF STUDY PROCEDURES

	ANC Visits	During Labor/ Before Delivery	After Delivery/ Before Discharge	3-day postpartum (pp)	7-day pp	14-day pp	28-day pp	42-day pp	As Needed
**Community Sensitization**	X								
**Screening**									
• *Eligibility confirmation*		X							
• *Clinical assessment*		X							
**Consent**		X							
**Randomization**		X							
**Drug Administration**		X							
**Baseline Data Collection**									
• *Sociodemographic information*		X	X						
• *Medical history*		X	X						
• *Labor and delivery information*			X						
**Monitoring**									
• *Drug side effects*		X	X	X	X			X	X
• *Maternal events during labor/delivery*		X	X						
• *Neonatal events during labor/delivery*			X						
• *Maternal infection/sepsis assessment*			X	X	X			X	X
• *Neonatal infection/sepsis assessment*			X	X	X			X	X
• *Maternal death*			X	X	X			X	X
• *Stillbirth or neonatal death within 28 days of birth*			X	X	X	X	X	X	X
• *Infant mortality after 28 days of birth*								X	X
• *Pyloric stenosis*			X	X	X			X	X
• *Other maternal outcomes*			X	X	X			X	X
• *Other neonatal outcomes*			X	X	X			X	X
• *Unintended medical visits*			X	X	X			X	X
**Serious Adverse Events**			X	X	X			X	X

For peer review only - http://bmjopen.bmj.com/site/about/guidelines.xhtml

Reporting checklist for protocol of a clinical trial.

Based on the SPIRIT guidelines.

Instructions to authors

Complete this checklist by entering the page numbers from your manuscript where readers will find each of the items listed below.

Your article may not currently address all the items on the checklist. Please modify your text to include the missing information. If you are certain that an item does not apply, please write "n/a" and provide a short explanation.

Upload your completed checklist as an extra file when you submit to a journal.

In your methods section, say that you used the SPIRITreporting guidelines, and cite them as:

Chan A-W, Tetzlaff JM, Gøtzsche PC, Altman DG, Mann H, Berlin J, Dickersin K, Hróbjartsson A, Schulz KF, Parulekar WR, Krleža-Jerić K, Laupacis A, Moher D. SPIRIT 2013 Explanation and Elaboration: Guidance for protocols of clinical trials. BMJ. 2013;346:e7586

		Reporting Item	Page Number
**Administrative information**			
Title	#1	Descriptive title identifying the study design, population, interventions, and, if applicable, trial acronym	1, 4
Trial registration	#2a	Trial identifier and registry name. If not yet registered,	2, 15

For peer review only

		name of intended registry	
Trial registration: data set	#2b	All items from the World Health Organization Trial Registration Data Set	N/A
Protocol version	#3	Date and version identifier	2
Funding	#4	Sources and types of financial, material, and other support	20
Roles and responsibilities: contributorship	#5a	Names, affiliations, and roles of protocol contributors	14, 15
Roles and responsibilities: sponsor contact information	#5b	Name and contact information for the trial sponsor	14
Roles and responsibilities: sponsor and funder	#5c	Role of study sponsor and funders, if any, in study design; collection, management, analysis, and interpretation of data; writing of the report; and the decision to submit the report for publication, including whether they will have ultimate authority over any of these activities	13
Roles and responsibilities: committees	#5d	Composition, roles, and responsibilities of the coordinating centre, steering committee, endpoint adjudication committee, data management team, and other individuals or groups overseeing the trial, if	13

For peer review only

		applicable (see Item 21a for data monitoring committee)	
**Introduction**			
Background and rationale	#6a	Description of research question and justification for undertaking the trial, including summary of relevant studies (published and unpublished) examining benefits and harms for each intervention	4
Background and rationale: choice of comparators	#6b	Explanation for choice of comparators	4
Objectives	#7	Specific objectives or hypotheses	4, 5
Trial design	#8	Description of trial design including type of trial (eg, parallel group, crossover, factorial, single group), allocation ratio, and framework (eg, superiority, equivalence, non-inferiority, exploratory)	4
**Methods: Participants, interventions, and outcomes**			
Study setting	#9	Description of study settings (eg, community clinic, academic hospital) and list of countries where data will be collected. Reference to where list of study sites can be obtained	4, 5
Eligibility criteria	#10	Inclusion and exclusion criteria for participants. If	5

For peer review only

		applicable, eligibility criteria for study centres and individuals who will perform the interventions (eg, surgeons, psychotherapists)	
Interventions: description	#11a	Interventions for each group with sufficient detail to allow replication, including how and when they will be administered	4, 5, 7-11
Interventions: modifications	#11b	Criteria for discontinuing or modifying allocated interventions for a given trial participant (eg, drug dose change in response to harms, participant request, or improving / worsening disease)	N/A
Interventions: adherance	#11c	Strategies to improve adherence to intervention protocols, and any procedures for monitoring adherence (eg, drug tablet return; laboratory tests)	9-11
Interventions: concomitant care	#11d	Relevant concomitant care and interventions that are permitted or prohibited during the trial	N/A (standard of care)
Outcomes	#12	Primary, secondary, and other outcomes, including the specific measurement variable (eg, systolic blood pressure), analysis metric (eg, change from baseline, final value, time to event), method of aggregation (eg, median, proportion), and time point for each outcome. Explanation of the clinical relevance of chosen efficacy and harm outcomes is strongly recommended	5-7
Participant timeline	#13	Time schedule of enrolment, interventions (including any	7-9

For peer review only

		run-ins and washouts), assessments, and visits for participants. A schematic diagram is highly recommended (see Figure)	
Sample size	#14	Estimated number of participants needed to achieve study objectives and how it was determined, including clinical and statistical assumptions supporting any sample size calculations	12-13
Recruitment	#15	Strategies for achieving adequate participant enrolment to reach target sample size	9-11
**Methods: Assignment of interventions (for controlled trials)**			
Allocation: sequence generation	#16a	Method of generating the allocation sequence (eg, computer-generated random numbers), and list of any factors for stratification. To reduce predictability of a random sequence, details of any planned restriction (eg, blocking) should be provided in a separate document that is unavailable to those who enrol participants or assign interventions	10
Allocation concealment mechanism	#16b	Mechanism of implementing the allocation sequence (eg, central telephone; sequentially numbered, opaque, sealed envelopes), describing any steps to conceal the sequence until interventions are assigned	10

For peer review only

Allocation: implementation	#16c	Who will generate the allocation sequence, who will enrol participants, and who will assign participants to interventions	10
Blinding (masking)	#17a	Who will be blinded after assignment to interventions (eg, trial participants, care providers, outcome assessors, data analysts), and how	10
Blinding (masking): emergency unblinding	#17b	If blinded, circumstances under which unblinding is permissible, and procedure for revealing a participant's allocated intervention during the trial	10
**Methods: Data collection, management, and analysis**			
Data collection plan	#18a	Plans for assessment and collection of outcome, baseline, and other trial data, including any related processes to promote data quality (eg, duplicate measurements, training of assessors) and a description of study instruments (eg, questionnaires, laboratory tests) along with their reliability and validity, if known. Reference to where data collection forms can be found, if not in the protocol	7, 8, 10
Data collection plan: retention	#18b	Plans to promote participant retention and complete follow-up, including list of any outcome data to be collected for participants who discontinue or deviate from	7-11

For peer review only

		intervention protocols	
Data management	#19	Plans for data entry, coding, security, and storage, including any related processes to promote data quality (eg, double data entry; range checks for data values). Reference to where details of data management procedures can be found, if not in the protocol	10
Statistics: outcomes	#20a	Statistical methods for analysing primary and secondary outcomes. Reference to where other details of the statistical analysis plan can be found, if not in the protocol	11-13
Statistics: additional analyses	#20b	Methods for any additional analyses (eg, subgroup and adjusted analyses)	12
Statistics: analysis population and missing data	#20c	Definition of analysis population relating to protocol non-adherence (eg, as randomised analysis), and any statistical methods to handle missing data (eg, multiple imputation)	11
**Methods: Monitoring**			
Data monitoring: formal committee	#21a	Composition of data monitoring committee (DMC); summary of its role and reporting structure; statement of whether it is independent from the sponsor and competing interests; and reference to where further details about its charter can be found, if not in the protocol. Alternatively, an explanation of why a DMC is not needed	11, 13

For peer review only

Data monitoring: interim analysis	#21b	Description of any interim analyses and stopping guidelines, including who will have access to these interim results and make the final decision to terminate the trial	13
Harms	#22	Plans for collecting, assessing, reporting, and managing solicited and spontaneously reported adverse events and other unintended effects of trial interventions or trial conduct	11
Auditing	#23	Frequency and procedures for auditing trial conduct, if any, and whether the process will be independent from investigators and the sponsor	N/A
**Ethics and dissemination**			
Research ethics approval	#24	Plans for seeking research ethics committee / institutional review board (REC / IRB) approval	2, 15
Protocol amendments	#25	Plans for communicating important protocol modifications (eg, changes to eligibility criteria, outcomes, analyses) to relevant parties (eg, investigators, REC / IRBs, trial participants, trial registries, journals, regulators)	15, 16
Consent or assent	#26a	Who will obtain informed consent or assent from potential trial participants or authorised surrogates, and how (see Item 32)	10, 15
Consent or assent:	#26b	Additional consent provisions for collection and use of	N/A

For peer review only

ancillary studies		participant data and biological specimens in ancillary studies, if applicable	
Confidentiality	#27	How personal information about potential and enrolled participants will be collected, shared, and maintained in order to protect confidentiality before, during, and after the trial	10, 15
Declaration of interests	#28	Financial and other competing interests for principal investigators for the overall trial and each study site	20
Data access	#29	Statement of who will have access to the final trial dataset, and disclosure of contractual agreements that limit such access for investigators	16
Ancillary and post trial care	#30	Provisions, if any, for ancillary and post-trial care, and for compensation to those who suffer harm from trial participation	N/A
Dissemination policy: trial results	#31a	Plans for investigators and sponsor to communicate trial results to participants, healthcare professionals, the public, and other relevant groups (eg, via publication, reporting in results databases, or other data sharing arrangements), including any publication restrictions	16
Dissemination policy: authorship	#31b	Authorship eligibility guidelines and any intended use of professional writers	N/A
Dissemination policy: reproducible	#31c	Plans, if any, for granting public access to the full protocol, participant-level dataset, and statistical code	16

For peer review only

research

Appendices

Informed consent materials	#32	Model consent form and other related documentation given to participants and authorised surrogates	N/A
Biological specimens	#33	Plans for collection, laboratory evaluation, and storage of biological specimens for genetic or molecular analysis in the current trial and for future use in ancillary studies, if applicable	16

Notes:

- 11d: N/A (standard of care) The SPIRIT Explanation and Elaboration paper is distributed under the terms of the Creative Commons Attribution License CC-BY-NC. This checklist was completed on 19. September 2022 using https://www.goodreports.org/, a tool made by the EQUATOR Network in collaboration with Penelope.ai

For peer review only

BMJ Open

Prevention of maternal and neonatal death/infections with a single oral dose of azithromycin in women in labor in low- and middle-income countries (A-PLUS): Study protocol for a multi-national, randomized placebo-controlled clinical trial

Journal:	*BMJ Open*
Manuscript ID	bmjopen-2022-068487.R1
Article Type:	Protocol
Date Submitted by the Author:	16-Apr-2023

Complete List of Authors:	Hemingway-Foday, Jennifer; RTI International<br>Tita, Alan; The University of Alabama at Birmingham School of Medicine<br>Chomba, Elwyn; University of Zambia University Teaching Hospital<br>Mwenechanya, Musaku; University of Zambia University Teaching Hospital<br>Mweemba, Trecious; University of Zambia University Teaching Hospital<br>Nolen, Tracy; RTI International<br>Lokangaka, Adrien; University of Kinshasa<br>Tshefu Kitoto, Antoinette; University of Kinshasa<br>Lomendje, Gustave; Kinshasa School of Public Health<br>Hibberd, Patricia; Boston University School of Public Health<br>Patel, Archana; Lata Medical Research Foundation; Datta Meghe Institute of Medical Sciences - Wardha Campus<br>Das, Prabir Kumar; Lata Medical Research Foundation, Nagpur<br>Kurhe, Kunal; Lata Medical Research Foundation<br>Goudar, Shivaprasad S.; KLE Academy of Higher Education and Research<br>Kavi, Avinash; KLE Academy of Higher Education and Research<br>Metgud, Mrityunjay; KLE Academy of Higher Education and Research<br>Saleem, Sarah; The Aga Khan University, Community Health Sciences<br>Tikmani, Shiyam; The Aga Khan University, Community Health Sciences<br>Esamai, Fabian; Moi University School of Medicine<br>Nyongesa, Paul; Moi University School of Medicine, Reproductive Health<br>Sagwe, Amos; Moi University School of Medicine<br>Figueroa, Lester; Institute of Nutrition of Central America and Panama<br>Mazariegos, Manolo; Instituto de Nutricion de Centroamerica y Panama<br>Billah, Sk Masum; International Centre for Diarrhoeal Disease Research, Maternal and Child Health Division; The University of Sydney<br>Haque, Rashidul; International Centre for Diarrhoeal Disease Research Bangladesh, Centre for Communicable Diseases<br>Shahjahan Siraj, Md; International Centre for Diarrhoeal Disease Research Bangladesh<br>Goldenberg, R; Columbia University<br>Bauserman, Melissa; University of North Carolina at Chapel Hill School of Medicine<br>Bose, Carl; The University of North Carolina at Chapel Hill School of Medicine<br>Liechty, Edward A; Indiana University School of Medicine<br>Ekhaguere, Osayame; Indiana University Department of Pediatrics,

	Neonatal-Perinatal Medicine<br>Krebs, Nancy F; University of Colorado Denver<br>Derman, Richard ; Thomas Jefferson University, Office of Global Affairs<br>Petri, William A.; University of Virginia<br>Koso-Thomas, Marion; Eunice Kennedy Shriver National Institute of Child Health and Human Development<br>McClure, Elizabeth; RTI International<br>Carlo, Waldemar; The University of Alabama at Birmingham, Pediatrics
**Primary Subject Heading**:	Global health
Secondary Subject Heading:	Public health, Obstetrics and gynaecology, Paediatrics
Keywords:	Maternal medicine < OBSTETRICS, NEONATOLOGY, Protocols & guidelines < HEALTH SERVICES ADMINISTRATION & MANAGEMENT, OBSTETRICS, PUBLIC HEALTH, Clinical trials < THERAPEUTICS

SCHOLARONE™
Manuscripts

Prevention of maternal and neonatal death/infections with a single oral dose of azithromycin in women in labor in low- and middle-income countries (A-PLUS): Study protocol for a multi-national, randomized placebo-controlled clinical trial

Jennifer J Hemingway-Foday[1], Alan Tita[2], Elwyn Chomba[3], Musaku Mwenechanya[3], Trecious Mweemba[3], Tracy L Nolen[1], Adrien Lokangaka[4], Antoinette Tshefu[4], Gustave Lomendje[4], Patricia L Hibberd[5], Archana Patel[6,7], Prabir Das[6], Kunal Kurhe[6], Shivaprasad S. Goudar[8], Avinash Kavi[8], Mrityunjay Metgud[8], Sarah Saleem[9], Shiyam Sunder Tikmani[9], Fabian Esamai[10], Paul Nyongesa[10], Amos Sagwe[10], Lester Figueroa[11], Manolo Mazariegos[11], Sk Masum Billah[12,13], Rashidul Haque[12], Md Shahjahan Siraj[12], Robert L Goldenberg[14], Melissa Bauserman[15], Carl L Bose[15], Edward A Liechty[16], Osayame Austine Ekhaguere[16], Nancy F. Krebs[17], Richard J Derman[18], William A Petri Jr[19], Marion Koso-Thomas[20], Elizabeth M McClure[1], Waldemar A Carlo[2]

[1]RTI International, Durham, NC, USA
[2]University of Alabama at Birmingham, Birmingham, AL, USA
[3]University Teaching Hospital, Lusaka, Zambia
[4]Kinshasa School of Public Health, Kinshasa, Democratic Republic of Congo
[5]Boston University School of Public Health, Boston, MA, USA
[6]Lata Medical Research Foundation, Nagpur, India
[7]Datta Meghe Institute of Medical Sciences, Wardha, India
[8]KLE Academy Higher Education and Research, J N Medical College Belagavi, Karnataka, India
[9]Aga Khan University, Karachi, Pakistan
[10]Moi University School of Medicine, Eldoret, Kenya
[11]Instituto de Nutrición de Centroamérica y Panamá, Guatemala City, Guatemala
[12]International Centre for Diarrhoeal Disease Research, Dhaka, Bangladesh
[13]University of Sydney, Sydney, NSW, Australia
[14]Columbia University School of Medicine, New York, NY, USA
[15]University of North Carolina at Chapel Hill, Chapel Hill, NC, USA
[16]Indiana School of Medicine, University of Indiana, Indianapolis, IN, USA
[17]University of Colorado School of Medicine, Denver, CO, USA
[18]Thomas Jefferson University, Philadelphia, PA, USA
[19]University of Virginia, Charlottesville, VA, USA
[20]*Eunice Kennedy Shriver* National Institute of Child Health and Human Development, Bethesda, MD,USA

Corresponding Author:
Jennifer J. Hemingway-Foday, MPH, MSW
RTI International
3040 E. Cornwallis Road
Research Triangle Park, NC, 27709
Telephone: (919)-485-2663
Email: hemingway@rti.org

For peer review only - http://bmjopen.bmj.com/site/about/guidelines.xhtml

Word count (excluding title page, abstract, references, figures and tables): 3998

ABSTRACT

Introduction

Maternal and neonatal infections are among the most frequent causes of maternal and neonatal mortality, and current antibiotic strategies have been ineffective in preventing many of these deaths. A randomized clinical trial conducted in a single site in The Gambia showed that treatment with oral dose of 2 g azithromycin vs. placebo for all women in labor reduced certain maternal and neonatal infections. However, it is unknown if this therapy reduces maternal and neonatal sepsis and mortality. In a large, multi-national randomized trial, we will evaluate the impact of azithromycin given in labor to improve maternal and newborn outcomes.

Methods and Analysis

This randomized, placebo-controlled, multicenter clinical trial includes two primary hypotheses, one maternal and one neonatal. The maternal hypothesis is to test whether a single, prophylactic intrapartum oral dose of 2 g azithromycin given to women in labor will reduce maternal death or sepsis. The neonatal hypothesis will test whether this intervention will reduce intrapartum/neonatal death or sepsis. The intervention is a single, prophylactic intrapartum oral dose of 2 g azithromycin, compared with a single intrapartum oral dose of an identical appearing placebo. A total of 34,000 laboring women from eight research sites in sub-Saharan Africa, South Asia, and Latin America will be randomized with one-to-one ratio to intervention/placebo. In addition, we will assess antimicrobial resistance in a sample of women and their newborns.

Ethics and Dissemination

The study protocol has been reviewed and ethics approval obtained from all the relevant ethical review boards at each research site.

Trial Registration

ClinicalTrials.gov number: NCT03871491

https://clinicaltrials.gov/ct2/show/NCT03871491?term=NCT03871491&draw=2&rank=1

Keywords: sepsis, azithromycin, low and middle-income countries; neonatal mortality; maternal mortality

For peer review only - http://bmjopen.bmj.com/site/about/guidelines.xhtml

STRENGTHS AND LIMITATIONS OF THIS STUDY

- This study evaluates the use of routine prophylactic azithromycin in women who undergo labor and attempt a vaginal delivery, including those at high risk of infection due to prolonged labor or membrane rupture, which was identified as a top research priority by the World Health Organization (WHO) and other stakeholders.
- The study evaluates a simple, inexpensive intervention that can be scaled up to reduce the burden of both maternal and neonatal mortality due to infections if successful.
- To optimize generalizability, this study will be carried out in a racially, ethnically, and geographically diverse population in seven low-and-middle-income countries.
- In response to global public health concerns about increasing antimicrobial resistance, this study includes an ancillary study to assess antimicrobial resistance and microbiome changes.
- In some locations, azithromycin and other antibiotics are increasingly being used, especially with increasing use of cesarean section, and thus the impact of the outcome may be diminished.

INTRODUCTION

Background

Maternal infection during pregnancy and the puerperium, accounting for 10% of maternal deaths, is a top cause of maternal mortality worldwide.[1] Maternal infections increase neonatal sepsis, a leading cause of neonatal death in low-income countries (LICs).[2-5] Neonatal infection accounts for 15% of neonatal mortality worldwide.[2] According to the World Health Organization (WHO), maternal and neonatal deaths from infections have remained unchanged or increased, whereas other causes of death have declined. [5,6]

Innovative, effective, and scalable interventions are needed to reduce infection-related maternal and neonatal mortality. The evidence backing current WHO guidelines to prevent and treat peripartum infections is limited.[7] Further, current approaches to address neonatal sepsis have had limited impact and alternative antibiotic regimens suggesting comparable effectiveness have not reduced newborn deaths.[8-11]

Risk Factors for Maternal and Neonatal Infection and Sepsis

Cesarean delivery (CD), especially after labor or membrane rupture, is an important risk factor for maternal peripartum infection.[12] Antibiotic prophylaxis for CD is a well-established, effective strategy to reduce infection.[13-16] However, with a prevalence of <5% in LICs, CDs do not account for most maternal sepsis worldwide.[17] Many peripartum infections in low-and-middle-income countries (LMICs) occur in women who deliver vaginally, with women undergoing prolonged labor or membrane rupture at highest risk. Although most pregnancies are not at high-risk for infection due to prolonged labor or membrane rupture, pregnancies where this does occur account for about 50% of maternal and neonatal infections in LMICs.[1] Therefore, identification and management of prolonged labor and/or membrane rupture are critical to reduce maternal sepsis.[7] Thus, the WHO has identified evaluation of routine prophylactic antibiotics for women undergoing vaginal birth or with prolonged labor/membrane rupture as a top research priority.[1]

Intrapartum Azithromycin to Prevent Maternal and Neonatal Infections

For peer review only - http://bmjopen.bmj.com/site/about/guidelines.xhtml

A novel approach to prevent maternal and neonatal infection is to target organisms that may be frequent pathogens but are not historically treatment targets.[16, 18-21] Azithromycin, which is available as a generic agent, has a bimodal half-life of 70 hours in the non-pregnant population. It is commonly used during pregnancy to treat chlamydia, gonorrhea, and other infections.[22] It provides broad coverage against most common pathogens associated with peripartum infections including gram-positive cocci, genital mycoplasmas and *Ureaplasma* infections, and certain gram-negative Bacilli and anaerobes associated with maternal polymicrobial infections and sepsis.[23] In addition, azithromycin also has activity against Group B *streptococcus* (GBS), a major cause of neonatal sepsis in developed countries and possibly in LICs.[24,25]

Evidence from Prior Studies

In a U.S. randomized clinical trial (RCT), a 50% reduction in maternal peripartum infection resulted from 500 mg intravenous azithromycin added to the standard prophylactic regimen in high-risk women undergoing CD following labor or membrane rupture ≥4 hours.[18] These results were observed despite universal antibiotics in both treatment groups. Readmission, unscheduled visits, serious adverse events, postpartum fever, or subsequent antibiotic treatment were also reduced in the azithromycin group. A related cost-analysis estimated that adjunctive azithromycin saved $360 USD per use in high-risk CDs and $143 USD per use in scheduled CDs.[26]

A trial in The Gambia suggested the potential for azithromycin prophylaxis to improve maternal and neonatal outcomes.[19] Among 829 participants randomized to 2 grams (g) of azithromycin vs. placebo before delivery, maternal infections were lower in the azithromycin group, 3.6% vs 9.2%, respectively (RR 0.40, 95% CI, 0.22-0.71, P=0.002). Among newborns, infections were also lower in the azithromycin group (18.1% vs 23.8%; RR 0.76 95% CI, 0.58-0.99; P=0.052). Maternal and neonatal carriage of infectious organisms was lower in the azithromycin group.

Rationale for a trial

Drawing from these findings, the role of a single oral dose of azithromycin (plus usual care) to prevent maternal death or peripartum sepsis and intrapartum/neonatal death or sepsis appears promising. Of interest is the sub-population at highest risk for infection due to prolonged labor and/or prolonged membrane rupture. Considering the success with a single 2g dose in The Gambian trial, which is bioequivalent to the 500 mg intravenous dose used successfully in the US trial, as well as the 40% bioavailability of oral azithromycin, 2g is considered the most appropriate dose for the proposed intervention. A RCT is the best design to evaluate efficacy and provide evidence for future policy and clinical practice decisions. Such a RCT in a racially, ethnically, and geographically diverse population could be accomplished through the established infrastructure of the Global Network for Women's and Children's Health Research (Global Network), a multi-country research network with sites in India, Pakistan, Bangladesh, Guatemala, Kenya, Zambia, and the Democratic Republic of Congo (DRC) and an established data coordinating center (DCC).[27]

METHODS AND ANALYSIS

Study Design and Intervention

This study, the Prevention of Maternal and Neonatal Death/Infections with a Single Oral Dose of Azithromycin in Women in Labor in Low- and Middle-Income Countries (A-PLUS), is a masked, placebo-controlled, multicenter, RCT. The intervention is a single 2g dose of oral azithromycin or identical placebo, administered as four 500 mg pills following randomization. Both groups will also receive the

For peer review only - http://bmjopen.bmj.com/site/about/guidelines.xhtml

local standard of care during labor, delivery, and postpartum. The trial adheres to the Standard Protocol Items: Recommendations for Interventional Trials (SPIRIT) statement [28].

Primary Hypothesis
The A-PLUS trial has both a maternal and neonatal primary hypothesis:

- Maternal: A single, prophylactic intrapartum oral dose of 2g azithromycin given to women in labor will reduce *maternal* death or sepsis.
- Neonatal: A single, prophylactic intrapartum oral dose of 2g azithromycin given to women in labor will reduce *intrapartum/neonatal* death or sepsis.

Study Population
Pregnant women laboring in health facilities at eight Global Network sites will be screened by research staff per the eligibility criteria (**Table 1**). The secondary population is a cohort of 5,500 high-risk women, defined as term and preterm (≥28 weeks) pregnant women with prolonged labor (≥18 hours) or prolonged membrane rupture (≥8 hours). A cohort of mother-infant dyads will also be randomly selected for an ancillary study to assess antimicrobial resistance and microbiome changes. Recruitment took place from September 2020- August 2022 and data collection for the primary outcome was completed in October 2022. Data collection for the ancillary study of antimicrobial resistance is expected to be complete by August 2023.

Table 1. Eligibility Criteria

**Inclusion Criteria**
• Pregnant women in labor with gestational age ≥ 28 weeks (by best estimate) who plan to deliver vaginally in a facility
• Admission to health facility with clear plan for spontaneous or induced vaginal delivery
• Presence of one or more live fetus confirmed via a fetal heart rate by Doptone prior to randomization
• Age ≥ 18 years of age (minors 14-17 years eligible in countries where married or pregnant minors or their authorized representatives are legally permitted to give consent)
• Provision of written informed consent
**Exclusion Criteria**
• Non-emancipated minors (as per local regulations)
• Evidence of chorioamnionitis or other infection requiring antibiotic therapy at time of eligibility *[women given single prophylactic antibiotics with no plans to continue after delivery will not be excluded]*
• Arrhythmia or known history of cardiomyopathy
• Allergy to azithromycin or other macrolides that is self-reported or documented in the medical record.
• Any use of azithromycin, erythromycin, or other macrolide in the 3 days or less prior to randomization.
• Plan for Cesarean delivery prior to enrollment
• Preterm labor undergoing management with no immediate plan to proceed to delivery
• Advanced stage of labor (>6 cm or 10 cm cervical dilation per local standards) and pushing or too distressed to understand, confirm, or give informed consent regardless of cervical dilation
• Not capable of giving consent due to other health problems such as obstetric emergencies (for example, antepartum hemorrhage) or mental disorder
• Any medical condition considered a contraindication per the judgement of site investigators
• Previous randomization in the trial

For peer review only - http://bmjopen.bmj.com/site/about/guidelines.xhtml

Patient and Public Involvement
Patients and/or the public were not involved in study design, conduct, or reporting.

Primary Outcomes
The primary outcomes are:

- Maternal: Incidence of maternal death or sepsis within 42 days post-delivery in the intervention vs. placebo group.
- Neonatal: Incidence of intrapartum/neonatal death or sepsis with 28 days post-delivery in the intervention vs. placebo group.

We use the 2017 WHO definition of sepsis which includes a suspicion of infection and the presence of organ dysfunction based on clinical findings.[7]

Maternal sepsis is defined as organ dysfunction resulting from suspected or confirmed infection that occurs post-randomization during labor or the postpartum period. This WHO definition is operationalized as suspected or confirmed infection based on the presence of fever or hypothermia plus one or more signs of mild to moderate organ dysfunction including tachycardia, low blood pressure, tachypnea, altered mental status/confusion, reduced urinary output, jaundice, or renal failure.[7, 29-31] Components of peripartum infection considered in diagnosing suspected or confirmed infection include clinical chorioamnionitis, endometritis, wound infections (perineal or cesarean), abdominal or pelvic abscess, mastitis/breast abscess or infection, pyelonephritis, pneumonia, and other bacterial infections (**Table 2).**

Table 2. Specified Infections Considered for Maternal Sepsis Diagnosis

Type of Infection	A-PLUS Trial Definition
Chorioamnionitis	Fever (>100.4°F/38°C) in addition to one or more of the following: fetal tachycardia ≥160 bpm, maternal tachycardia >100 bpm, tender uterus between contractions, or purulent/foul smelling discharge from uterus **prior** to delivery.
Endometritis	Fever (>100.4°F/38°C) in addition to one or more of maternal tachycardia >100 bpm, tender uterine fundus, or purulent/foul smelling discharge from uterus **after** delivery.
Wound infection	Purulent infection (superficial or deep infection including necrotizing fasciitis) of a perineal or Cesarean wound with or without fever. In the absence of purulence, a wound infection requires presence of fever (>100.4°F/38°C) and at least one of the following signs of local infection: pain or tenderness, swelling, heat, or redness around the incision/laceration
Abdominopelvic abscess	Evidence of pus in the abdomen or pelvis noted during open surgery, interventional aspiration or imaging
pneumonia	Fever (>100.4°F/38°C) and clinical symptoms suggestive of lung infection including cough and/or tachypnea (>24 breaths/min) or radiological confirmation
pyelonephritis	Fever (>100.4°F/38°C) and one or more of the following: urinalysis/dip suggestive of infection, costovertebral angle tenderness, or confirmatory urine culture
mastitis/breast abscess or infection	Fever (>100.4°F/38°C) and one or more of the following: breast pain, swelling, warmth, redness, or purulent drainage

Neonatal sepsis is defined as proven or possible serious bacterial infection (pSBI) or pneumonia, or meningitis. PSBI will be determined using WHO criteria of severe chest in-drawing, fever, hypothermia, no movement at all or movement only on stimulation, feeding poorly or not feeding at all, and/or convulsions.[32] Clinical and laboratory signs of infection will also be considered for diagnosis.

For peer review only - http://bmjopen.bmj.com/site/about/guidelines.xhtml

Centralized adjudication of key infection outcomes will be implemented to standardize the results. Reported antibiotic treatment and culture status will also be considered for outcome adjudication.

The individual components of these primary outcomes will be analyzed.

Secondary maternal outcomes

- The primary maternal outcome in a high-risk for infection population
- Incidence of specific maternal infections (Table 2)
- Subsequent maternal antibiotic therapy after randomization to 42 days postpartum for any reason
- Time from drug administration until initial discharge after delivery
- Maternal readmissions and admissions to special care units within 42 days postpartum
- Maternal unscheduled visit for care
- Maternal gastrointestinal symptoms (nausea, vomiting, diarrhea) or other reported side effects
- Maternal death due to sepsis using the Global Network algorithm for cause of death[33]

Secondary Neonatal Outcomes

- Neonatal deaths due to sepsis and all-cause neonatal death in all participants and in laboring women at high risk of infection
- Other neonatal infections (e.g., eye infection, skin infection, omphalitis, urinary tract infection, respiratory rate ≥60 breaths/minute).
- Neonatal readmissions and admissions to special care units within 42 days postpartum
- Neonatal initial hospital length of stay, defined as time of delivery until initial discharge
- Neonatal unscheduled visit for care
- Neonatal death due to sepsis using the Global Network algorithm for cause of death[34]
- Incidence of pyloric stenosis within 42 days of delivery

Study Procedures

All sites will train research staff in standardized study procedures to administer the intervention and assess outcomes. The sequence of study activities is described (**Figure 1)**. Study procedures are also provided (**Table 3)**.

Site Preparation

Prior to the trial, sites conducted an observational pilot study using the RCT's planned infrastructure to characterize current practices and optimize identification of suspected infection. The pilot study data was used to validate estimates of intrapartum deaths, maternal sepsis, and neonatal sepsis for the RCT's sample size. Each site also met with local health authorities and conducted community sensitization to ensure that study procedures were appropriate for the local context and to encourage facility and community-level engagement.

Screening

Pregnant women admitted for delivery at pre-defined health facilities will be screened for eligibility by research staff, per harmonized criteria (**Table 1**). If a contraindication to participation is found, the woman will be excluded.

For peer review only - http://bmjopen.bmj.com/site/about/guidelines.xhtml

Table 3. Schedule of Study Procedures

	ANC Visits	During Labor/ Pre-delivery	Post-delivery/ Pre-discharge	3-day post-partum (pp)	7-day pp	14-day pp	28-day pp	42-day pp	As Needed
**Community Sensitization**	X								
**Screening**									
▪ *Eligibility confirmation*		X							
▪ *Clinical assessment*		X							
**Consent**		X							
**Randomization**		X							
**Drug Administration**		X							
**Baseline Data Collection**									
▪ *Sociodemographic information*		X	X						
▪ *Medical history*		X	X						
▪ *Labor & delivery (L&D) information*			X						
**Monitoring**									
▪ *Drug side effects*		X	X	X	X			X	X
▪ *Maternal events during L&D*		X	X						
▪ *Neonatal events during L&D*			X						
▪ *Maternal infection/sepsis*			X	X	X			X	X
▪ *Neonatal infection/sepsis*			X	X	X			X	X
▪ *Maternal death*			X	X	X			X	X
▪ *Stillbirth or neonatal death within 28 days of birth*			X	X	X	X	X	X	X
▪ *Infant mortality ≥ 28 days of birth*								X	X
▪ *Pyloric stenosis*			X	X	X			X	X
▪ *Other maternal outcomes*			X	X	X			X	X
▪ *Other neonatal outcomes*			X	X	X			X	X
▪ *Unintended medical visits*			X	X	X			X	X
**Serious Adverse Events**			X	X	X			X	X

Consent

Research staff at each site are responsible for obtaining informed consent prior to complete cervical dilation or dilation limit approved by local authorities. Participants who are randomly selected to participate in the ancillary study on antimicrobial resistance will also be consented at this time. To minimize burden on the laboring women, sites may obtain initial consent from potential participants during the antenatal period and reconfirm consent at screening. As literacy levels vary, the consent form will be reviewed verbally. All research staff responsible for consent procedures will be trained and certified in the protection of human subjects and the study-specific consent procedures.

Masking

Both the azithromycin and placebo will be procured from the same manufacturer (Idifarma, Spain). The packaging will be standardized across sites and labeled as: "Azithromycin 2 g or Placebo" with the expiration date and a unique identifier. Clinical and research staff, and participants, will be masked to treatment status unless a serious adverse event potentially related to the treatment requires unmasking. Each site's study pharmacist will monitor randomization, drug supply, and safety.

For peer review only - http://bmjopen.bmj.com/site/about/guidelines.xhtml

Randomization

A computer-generated randomization scheme, created and known only by the DCC, will use a randomly permuted block design with randomly varied block to achieve a 1:1 randomization, stratified by site. Each site will receive study drug directly from the manufacturer, which is sequentially prepared per the randomization scheme in identical packaging. The study drug will be distributed to participating health facilities and dispensed sequentially by study staff.

Monitoring before Discharge (Baseline Assessment)

Routine post-delivery care will be provided by clinical providers, trained to contact research staff in case of suspected or confirmed infection. Research staff will collect demographic characteristics, key clinical measures, and outcomes from randomization until discharge. Data will be abstracted from medical records or collected directly from participants, as relevant. All data will be directly entered into a secure electronic data capture platform, Research Electronic Data Capture (REDCap). De-identified data will then be transmitted to the DCC. In addition, participants will be educated on signs and symptoms of infection and encouraged to call research staff with any concerns.

Monitoring after Discharge (Postpartum Follow-up Assessments)

After discharge, research staff will contact participants at the following timepoints:

- In-person visits at 3-, 7-, and 42-day postpartum to identify maternal or infant infection, unexpected medical visits, side effects, and other study outcomes. If suspected infection, research staff will collect and send relevant specimens to the local microbiology lab for analysis.
- Supplemental phone contacts at 14- and 28-day postpartum to review maternal and neonatal signs of infection (WHO criteria). If signs are identified, participants will visit a study facility for further assessment. These supplemental contacts will reinforce the participants' ability to self-assess maternal and neonatal infection and improve identification between the 7- and 42-day visits.

If indicated, records of non-study visits to health providers during the follow-up period will be reviewed to ascertain study outcomes. Providers may be called if clarification is needed. Readmissions and related diagnoses identified during follow-up visits will be validated through medical record review. All data will be entered into REDCap and de-identified data will be transmitted to the DCC.

Safety Monitoring

Surveillance of maternal side effects including nausea, vomiting, and diarrhea/loose stools, abdominal pain, vaginitis, and dizziness potentially associated with azithromycin will be conducted during labor and postpartum. For infants, findings suggestive of pyloric stenosis will be assessed during the follow up visits. Maternal and neonatal surveillance will also include assessment of unintended medical visits, maternal deaths, stillbirths, neonatal death <28 days of birth, and infant death >28 days. Additional maternal and neonatal risks associated with azithromycin use include anaphylaxis, allergic reactions (rash), liver failure, and arrhythmias. Although rare, these side effects will be monitored and reported as serious adverse events. All safety outcomes will be reviewed by the Data Monitoring Committee (DMC) appointed by NICHD.

Analytical Plan

Baseline demographic characteristics and key clinical measures will be compared between the treatment arms using contingency table approaches for categorical variables and analysis of variance models' continuous variables.

For peer review only - http://bmjopen.bmj.com/site/about/guidelines.xhtml

P-values presented will be based on two-sided tests unless otherwise specified, adjusted for site. For most analyses, the interaction between treatment and site will be assessed and if significant, also be presented by site. For continuous outcomes, distributional properties will be evaluated and if required, transformations or non-parametric tests employed. Additional details for potential covariate adjustments in secondary analyses or handling violations of analytic method assumptions will be detailed in the statistical analysis plan.

Three key populations are of interest for study analyses:

1. The Intention to Treat (ITT) population will be the primary analysis population and will include all women randomized and their infants. Analyses of this cohort will be conducted based on randomized treatment.
2. The high risk for infection sub-group will include all women in the ITT and their infants' meeting criteria for being high risk (i.e., prolonged labor and/or rupture of membranes) at randomization. Analyses of this cohort will be conducted based on randomized treatment.
3. The as-treated population will include all randomized participants that receive any study drug during the study and their infants. Analyses of this cohort will be conducted based on treatment received.

The final determination of analysis population membership will be via a masked data review prior to final study analyses to address any potential anomalous cases that may arise in this large study population (e.g., randomization/treatment of a woman who is discharged prior to delivery due to false labor or unresponsiveness to induction).

Primary Analysis

Incidence of maternal death or sepsis and intrapartum/neonatal death or sepsis will be compared separately between the treatment arms using generalized linear models fit with each binary outcome separately as the outcome measure. Estimates of RR and associated 95% CIs will be reported. The model will include terms for treatment and site. As randomization occurs at the pregnancy level and approximately 1-2% of pregnancies are anticipated to be multiple gestations, models for neonatal outcomes will account for correlation among multiples assuming an exchangeable covariance structure. For the two primary outcomes, analyses will be conducted using the ITT population and the p-values associated with the treatment term will be used to formally test each of the two primary hypotheses at the alpha=0.05 level.

As secondary analyses of the primary outcomes, assuming an overall treatment effect is observed, the models will be run including region (Africa, Latin America, or Asia) and a treatment by region interaction term. If the interaction term has a $p<0.1$, effects will be reported by region with treatment effect within region tested at the 0.025 level.

Additional exploratory models will also be run including individually: 1) a treatment-by-site interaction term, 2) any other antibiotic use during labor (yes or no) and its interaction with treatment, and 3) mode of delivery (cesarean or vaginal) and its interaction with treatment. If the interaction term for any of these models has a $p<0.1$, then effects will also be reported by the relevant subgroups.

From each final model, estimates of relative risk associated with treatment will be obtained including unadjusted estimates of risk from the primary model as well as estimates of risk adjusted for potential confounders from the secondary analyses.

For peer review only - http://bmjopen.bmj.com/site/about/guidelines.xhtml

For peer review only

Secondary Analyses: Women at high-risk for infection cohort

The major secondary aim is assessing the two primary outcomes (i.e., incidence of maternal death or sepsis and incidence of intrapartum/neonatal death or sepsis) in the high-risk cohort. These analyses will also assess if the treatment effect differs between the high-risk sub-group vs. non-high-risk women, which include all women and their infants in the ITT population that delivered prior to meeting high-risk criteria. Specifically, the maternal and neonatal primary outcomes will be modeled including a treatment by risk status interaction term and exclude data from individuals meeting high-risk criteria after randomization.

Secondary Analyses: Other Secondary Outcomes

Other maternal and neonatal binary outcomes will be analyzed using the approaches described for the primary analysis for the ITT population and approaches detailed for the secondary analysis for the HR cohort. A similar process with generalized linear models employing an appropriate link function will be used to analyze the outcomes of maternal and neonatal initial hospital length of stay. Binary safety outcomes will also be analyzed using the approaches detailed for the primary analysis. These analyses will be conducted using the as-treated population.

Sample Size for Primary Outcome

Sample size estimates were generated to evaluate the potential benefits of peripartum prophylactic azithromycin in two population cohorts of women (**Table 4**). The first population comprises all women delivering in facilities.

Table 4. Sample Sizes

**4A. Sample Size for the Overall Population (alpha=0.05)**				
		Evaluable Sample Size per Arm		
Baseline Risk	Risk Reduction	Power=0.80	Power=0.85	Power=0.90
3%	20%	11455	13103	15334
3%	25%	7133	8159	9548
3%	30%	4815	5508	6446
8%	20%	4096	4686	5483
8%	25%	2554	2921	3419
8%	30%	1727	1975	2311
14%	20%	2204	2521	2950
14%	25%	1377	1575	1842
14%	30%	932	1066	1247
**4B. Sample Size for High-risk Group (alpha = 0.05)**				
6%	20%	5568	6369	7453
6%	25%	3470	3969	4644
6%	30%	2344	2681	3138
**4C. Sample Size by Region (alpha =0.025)**				
8%	20%	4961	5607	6477
8%	25%	3093	3496	4038
8%	30%	2091	2363	2729
14%	20%	2669	3017	3485
14%	25%	1677	1884	2176
14%	30%	1129	1276	1474

For peer review only - http://bmjopen.bmj.com/site/about/guidelines.xhtml

Power calculations for the overall study population were generated for the primary maternal and neonatal outcome measures. For each, estimates of the required sample size needed to detect a risk reduction of 20%, 25%, and 30% were generated for power of 0.8, 0.85, and 0.9. The risk of sepsis or maternal death was assumed to be 3%, based on data collected from 2010-2018 through the Global Network's Maternal Newborn Health Registry (MNHR), a prospective, population-based registry of pregnant women and neonates receiving care in defined Global Network catchment areas [27], augmented with active surveillance. For the neonatal outcome, the underlying risk of the combined outcome was estimated to be between 8% and 14% based on Global Network data. [35] We assumed that the risk of sepsis not resulting in death is approximately 4% to 10%. We will also test the neonatal risk separately in south Asia and sub-Saharan Africa. Approximately 37.5% of randomized mothers will be from sub-Saharan Africa and 50% will be from Asia, reflecting the rates observed in the Global Network.[35,36].

For the primary neonatal outcome of interest of intrapartum/neonatal sepsis or death, assuming that the loss to follow-up will be in the 2% to 3% range, this sample size of 34,000 will provide 90% power to detect a 25% reduction in neonatal mortality and sepsis in the sub-Saharan African region and will provide 90% power to detect a 20% reduction in Asia assuming the baseline risk is at least 8%. For the primary maternal outcome of maternal death or sepsis, the sample size will provide 90% power to detect a 20% reduction from 3% in the population aggregated across all study sites. Each site will aim to recruit an equal number of participants (n=4,250 per site) but site-specific sample sizes may be adjusted if targets are not met. No site will be permitted to recruit more than 20% of the overall study sample site.

Data Monitoring Plan and Stopping Rules

Each site will report data, including adverse events, to the Global Network's DCC. The data will be used to evaluate protocol adherence and site performance (e.g., recruitment, loss to follow-up, data quality). The DCC will provide standardized progress reports to NICHD and the site investigators monthly to monitor outcome variables and adverse events.

Trial oversight will be handled by two principal groups: (1) a protocol-focused Steering Committee (**Table 5**); and (2) a DMC, designated by NICHD to ensure safe and ethical treatment of participants through bi-annual (at minimum) review of data on participant safety, study progress, and futility. One formal interim analysis of efficacy and futility will be conducted by the DMC during the study.

Table 5. A-PLUS Steering Committee

**A-PLUS CENTRAL STUDY TEAM**	
A-PLUS Lead Study Site (University of Alabama-Birmingham/University Teaching Hospital, Lusaka, Zambia)	
Waldemar A. Carlo, MD<br>Principal Investigator<br>University of Alabama at Birmingham, United States	Alan Tita, MD, PhD<br>A-PLUS Lead Investigator<br>University of Alabama at Birmingham, United States
Elwyn Chomba, MBChB, DCH, MRCP<br>Senior Foreign Investigator<br>University Teaching Hospital, Lusaka, Zambia	Musaku Mwenechanya<br>Country Coordinator<br>University Teaching Hospital, Lusaka, Zambia
Trecious Mweemba<br>A-PLUS Coordinator<br>University Teaching Hospital, Lusaka, Zambia	
RTI International (Global Network Data Coordinating Center)	
Elizabeth M. McClure, PhD	Tracy Nolen, DrPh

For peer review only - http://bmjopen.bmj.com/site/about/guidelines.xhtml

Principal Investigator<br>RTI International	Co-Principal Investigator and Senior Statistician<br>RTI International
Jennifer J. Hemingway-Foday, MPH, MSW<br>A-PLUS Protocol Manager<br>RTI International	
Eunice Kennedy Shriver National Institute of Child Health and Human Development (NICHD)	
Marion Koso-Thomas, MD, MPH<br>Medical Officer, Global Network for Women's and Children's Health	
**A-PLUS RESEARCH SITES**	
Democratic Republic of Congo (Kinshasa School of Public Health/University of North Carolina)	
Antoinette Tshefu, M.D, Ph.D., M.P.H<br>Senior Foreign Investigator<br>Kinshasa School of Public Health, Kinshasa, DRC	Carl L. Bose, M.D.<br>Principal Investigator<br>University of North Carolina School of Medicine, U.S.
Adrien Lokangaka, M.D., M.P.H.<br>Country Coordinator<br>Kinshasa School of Public Health	Gustave Lomendje<br>A-PLUS Coordinator<br>Kinshasa School of Public Health
Guatemala (Instituto de Nutricion de Centro America y Panama [INCAP]/University of Colorado)	
Manolo Mazariegos, MD, MPH<br>Senior Foreign Investigator,<br>INCAP, Guatemala City, Guatemala	Nancy F. Krebs, MD<br>Principal Investigator,<br>University of Colorado Health Science Center
Lester Figueroa<br>Country Coordinator<br>INCAP, Guatemala City, Guatemala	
**Bangladesh (International Centre for Diarrhoeal Disease Research [ICDDR,b]/University of Virginia)**	
Rashidul Haque, MD<br>Senior Foreign Investigator<br>ICDDR,b	William Petri, MD<br>Principal Investigator<br>University of Virginia
Sk Masum Billah<br>Country Coordinator<br>ICDDR,b	Md Shahjahan Siraj<br>A-PLUS Coordinator<br>ICDDR,b
**Belagavi, India (KLE University's JN Medical College/Thomas Jefferson University)**	
Shivaprasad S. Goudar MD, MHPE<br>Senior Foreign Investigator<br>KLE University's J N Medical College	Richard Derman, MD, MPH<br>Principal Investigator<br>Thomas Jefferson University
Avinash Kavi<br>A-PLUS Coordinator<br>KLE University's J N Medical College	Mrityunjay Metgud<br>A-PLUS Coordinator<br>KLE University's J N Medical College
**Pakistan (Aga Khan University/Columbia University)**	
Sarah Saleem, MD<br>Senior Foreign Investigator<br>Aga Khan University	Robert L. Goldenberg, MD<br>Principal Investigator<br>Columbia University
Shiyam Sunder Tikmani<br>A-PLUS Coordinator<br>Aga Khan University	
**Nagpur, India (Lata Medical Research Foundation/Boston University)**	
Archana Patel, MD, DNB, MSCE<br>Senior Foreign Investigator<br>Lata Medical Research Foundation	Patricia L. Hibberd, MD, PhD<br>Principal Investigator<br>Boston University School of Public Health

For peer review only - http://bmjopen.bmj.com/site/about/guidelines.xhtml

Prabir Das<br>Country Coordinator<br>Lata Medical Research Foundation	Kunal Kurhe<br>A-PLUS Coordinator<br>Lata Medical Research Foundation
**Kenya (Moi University School of Medicine/University of Indiana School of Medicine)**	
Fabian Esamai, MBChB, MMed, PhD<br>Senior Foreign Investigator<br>Moi University School of Medicine, Eldoret, Kenya	Edward A. Liechty, MD<br>Principal Investigator<br>Indiana University School of Medicine
Paul Nyongesa<br>Country Coordinator<br>Moi University School of Medicine, Eldoret, Kenya	Amos Sagwe<br>A-PLUS Coordinator<br>Moi University School of Medicine, Eldoret, Kenya
Osayame Austine Ekhaguere<br>Co-Investigator<br>Indiana University School of Medicine	

Adverse Event Monitoring Plan

Adverse events will be reported and submitted to the DCC (and IRBs) who will report these cumulative masked data to the DMC in the bi-annual safety reviews. Safety reports will be reviewed internally by the DCC quarterly and the DMC chair will be notified if any potential safety signals are identified to allow for more frequent DMC monitoring if needed.

ETHICS AND DISSEMINATION

Ethics Approval and Consent to Participate

The A-PLUS study protocol has been reviewed and approved by the relevant ethics committees and regulatory authorities at each research site, including the Institutional Review Board at the University of Alabama at Birmingham, which serves as the lead site, and the DCC at RTI International *[See supplemental file 1 for current version 1.6, dated 13 July 2022].* The DMC has reviewed the study protocol and will continue to review throughout the enrollment period. The study was registered (clinicaltrials.gov NCT03871491).

All research staff responsible for obtaining informed consent will be trained and certified in the protection of human subjects and the study-specific consent procedures. A model written informed consent form, developed according to the requirements of the U.S. Office for Human Research Protection (OHRP), can be found as Appendix 2 of the trial protocol (see supplemental file 1). The model consent may be modified by each site to conform to local standards, but the OHRP required elements must be maintained.

Potential Risks and Benefits to Participation

There are several potential direct and indirect benefits of this trial. Emerging data suggest that intrapartum azithromycin reduces maternal and neonatal infection. As infections are a frequent cause of maternal and neonatal deaths, there is a possibility that mortality could be reduced at the participating sites, as well as worldwide.

An ongoing concern for peripartum and perinatal antibiotic prophylaxis is the selection of resistant organisms including azithromycin-resistant organisms leading to resistant infections, and concern that disruption of gut and other flora (microbiome) in women and particularly in neonates may lead to adverse events including allergic reactions, rash and childhood asthma.[16,28,37] There is a paucity of data to address concerns that disturbances in the establishment of the indigenous intestinal microbiome

For peer review only - http://bmjopen.bmj.com/site/about/guidelines.xhtml

caused by antibiotic exposure in early life or CD, either directly or through modifications of breast microbiome, may increase risk of immune-mediated and inflammatory conditions later in life.[38-41] In response to these global public health concerns, this protocol includes an ancillary study to monitor for antimicrobial resistance and changes to the maternal and newborn microbiome.

Dissemination

Following completion of primary data collection, we will disseminate findings through international meetings and high impact peer-reviewed journals. Per NIH and Global Network Data Management and Sharing Policies, final research data will also be made publicly available through the NICHD Data and Specimen Hub (DASH) system within one year of publishing the primary manuscript. All data will be de-identified and shared under the assurance of confidentiality and approval from the relevant IRBs. Trial results will also be shared with community members, health workers, health officials, and other stakeholders from the areas where participants were recruited. If the results are positive, we will facilitate practice change at participating sites and approach the WHO to evaluate guideline updates based on study findings.

Figure 1. Study Flowchart

For peer review only

For peer review only - http://bmjopen.bmj.com/site/about/guidelines.xhtml

References

1. WHO recommendations for the prevention and treatment of maternal peripartum infections. [Internet]. World Health Organization. World Health Organization; 2015 [retrieved 2018 Aug 22]. Available from: https://www.who.int/publications/i/item/9789241549363
2. Liu L, Oza S, Hogan D, Perin J, Rudan I, Lawn JE, et al. Global, regional, and national causes of child mortality in 2000-13, with projections to inform post-2015 priorities: an updated systematic review. Lancet 2015;385(9966), 430-440.
3. Fleischmann-Struzek C, Goldfarb DM, Schlattmann P, Schlapbach LJ, Reinhart K, Kissoon N. The global burden of paediatric and neonatal sepsis: a systematic review. Lancet Respir Med. 2018;6(3):223–30.
4. Fleischmann C, Reichert F, Cassini A, Horner R, Harder T, Markwart R, et al. Global incidence and mortality of neonatal sepsis: a systematic review and meta-analysis. Arch Dis Child. 2021;106(8):745-52.
5. Baguiya A, Bonet M, Cecatti JG, Brizuela V, Curteanu A, Minkauskiene M, et al; WHO Global Maternal Sepsis Study (GLOSS) Research Group; GLOSS research group. Perinatal outcomes among births to women with infection during pregnancy. Arch Dis Child. 2021;106(10):946-53.
6. Brizuela V, Cuesta C, Bartolelli G, Abdosh AA, Abou Malham S, Assarag B, et al; WHO GLOSS Research Group. Availability of facility resources and services and infection-related maternal outcomes in the WHO Global Maternal Sepsis Study: a cross-sectional study. Lancet Glob Health. 2021;9(9):e1252-e1261.
7. World Health Organization. (2017). Statement on maternal sepsis. Retrieved August 22, 2018, from http://www.who.int/reproductivehealth/publications/maternal_perinatal_health/maternalsepsis-statement/en/
8. Tshefu, A., Lokangaka, A., Ngaima, S., Engmann, C., Esamai, F., Gisore, P., . . . African Neonatal Sepsis Trial (AFRINEST) group. Simplified antibiotic regimens compared with injectable procaine benzylpenicillin plus gentamicin for treatment of neonates and young infants with clinical signs of possible serious bacterial infection when referral is not possible: a randomised, open-label, equivalence trial. Lancet 2015; 385(9979), 1767-1776.
9. Baqui AH, Saha SK, Ahmed AS, Shahidullah M, Quasem I, Roth DE, et al. Projahnmo Study Group in Bangladesh. Safety and efficacy of alternative antibiotic regimens compared with 7 day injectable procaine benzylpenicillin and gentamicin for outpatient treatment of neonates and young infants with clinical signs of severe infection when referral is not possible: a randomised, open-label, equivalence trial. Lancet Global Health 2015; 3(5), e279-287.
10. Mir F, Nisar I, Tikmani SS, Baloch B, Shakoor S, Jehan F, et al. Simplified antibiotic regimens for treatment of clinical severe infection in the outpatient setting when referral is not possible for young infants in Pakistan (Simplified Antibiotic Therapy Trial [SATT]): a randomised, open-label, equivalence trial. Lancet Global Health 2017; 5(2), e177-e185.
11. Zaidi AK, Tikmani SS, Sultana S, Baloch B, Kazi M, Rehman H, et al. Simplified antibiotic regimens for the management of clinically diagnosed severe infections in newborns and young infants in first-level facilities in Karachi, Pakistan: study design for an outpatient randomized controlled equivalence trial. Pediatric Infectious Disease Journal 2013; 32 Suppl 1(Suppl 1), S19-25.
12. World Health Organization. WHO Statement on Caesarean Section Rates. 2015. Retrieved August 22, 2018, from

For peer review only - http://bmjopen.bmj.com/site/about/guidelines.xhtml

http://www.who.int/reproductivehealth/publications/maternal_perinatal_health/cs-statement/en/

13. Mackeen AD, Packard RE, Ota E, Berghella V, Baxter JK. Timing of intravenous prophylactic antibiotics for preventing postpartum infectious morbidity in women undergoing cesarean delivery. Cochrane Database of Systematic Reviews 2014. 12(12), CD009516.
14. Smaill FM, Grivell RM. Antibiotic prophylaxis versus no prophylaxis for preventing infection after cesarean section. Cochrane Database of Systematic Reviews 2014. 10, CD007482.
15. Gyte GML, Dou L, Vazquez JC. Different classes of antibiotics given to women routinely for preventing infection at caesarean section. Cochrane Database of Systematic Reviews. 2014; 11(11), CD008726.
16. Tita ATN, Rouse DJ, Blackwell S, Saade GR, Spong CY, Andrews WW. Evolving concepts in antibiotic prophylaxis for cesarean delivery: A systematic review. Obstetrics and Gynecology. 2009;113(3):675–82
17. Harrison MS, Saleem S, Ali S, Pasha O, Chomba E, Carlo WA, et al. A Prospective, Population-Based Study of Trends in Operative Vaginal Delivery Compared to Cesarean Delivery Rates in Low- and Middle-Income Countries, 2010-2016. Am J Perinatol. 2019;36(7):730-736.
18. Tita ATN, Boggess K, et al. Adjunctive azithromycin prophylaxis for cesarean delivery. New Eng J Med 2015. 375:1231-1241.
19. Oluwalana C, Camara B, Bottomley C, Goodier S, Bojang A, Kampmann B et al. Azithromycin in labor lowers clinical infections in mothers and newborns: a double-blind trial. Pediatrics 2017, 139(2).
20. Hume-Nixon M, Quach A, Reyburn R, Nguyen C, Steer A, Russell F. A Systematic Review and meta-analysis of the effect of administration of azithromycin during pregnancy on perinatal and neonatal outcomes. EClinicalMedicine. 2021;40:101123.
21. Hallamaa L, Cheung YB, Maleta K, Luntamo M, Ashorn U, Gladstone M, Kulmala T, Mangani C, Ashorn P. Child Health Outcomes After Presumptive Infection Treatment in Pregnant Women: A Randomized Trial. Pediatrics. 2018;141(3):e20172459.
22. Fitzgibbon A, Clooney L, Broderick D, Eogan M, McCallion N, Drew RJ. Erythromycin compared to amoxicillin and azithromycin for antimicrobial prophylaxis for preterm premature rupture of the membranes: a retrospective study. J Obstet Gynaecol. 2021 May;41(4):569-572.
23. Sutton AL, Acosta EP, Larson KB, Kerstner-Wood CD, Tita AT, Biggio JR. Perinatal pharmacokinetics of azithromycin for cesarean prophylaxis. Am J Obstet Gynecol 2018; 212(6), 812.
24. Russell NJ, Seale AC, O'Driscoll M, O'Sullivan C, Bianchi-Jassir F, Gonzalez-Guarin J, et al; GBS Maternal Colonization Investigator Group. Maternal Colonization With Group B Streptococcus and Serotype Distribution Worldwide: Systematic Review and Meta-analyses. Clin Infect Dis. 2017 Nov 6;65(suppl_2):S100-S111.
25. Rac H, Gould AP, Eiland LS, Griffin B, McLaughlin M, Stover KR, Bland CM, Bookstaver PB. Common Bacterial and Viral Infections: Review of Management in the Pregnant Patient. Ann Pharmacother. 2019 Jun;53(6):639-651.
26. Harper LM, Kilgore M, Szychowski JM, Andrews WW, Tita ATN. Economic evaluation of adjunctive azithromycin prophylaxis for cesarean delivery. Obstet Gynec 2017; 130(2), 328-334.
27. McClure EM, Garces AL, Hibberd PL, Moore JL, Goudar SS, Saleem S, et al. The Global Network Maternal Newborn Health Registry: a multi-country, community-based registry of pregnancy outcomes. Reprod Health. 2020;17(Suppl 2):184.

For peer review only - http://bmjopen.bmj.com/site/about/guidelines.xhtml

28. Chan A-W, Tetzlaff JM, Gøtzsche PC, Altman DG, Mann H, Berlin J, Dickersin K, Hróbjartsson A, Schulz KF, Parulekar WR, Krleža-Jerić K, Laupacis A, Moher D. SPIRIT 2013 Explanation and Elaboration: Guidance for protocols of clinical trials. BMJ. 2013;346:e7586
29. Bonet, M., Souza, J. P., Abalos, E., Fawole, B., Knight, M., Kouanda, S., et al. The global maternal sepsis study and awareness campaign (GLOSS): study protocol. Reprod Health 2018; 15(1), 16.
30. Albright, C.M., Has, P., Rouse D.J., Hughes B.L. Internal Validation of the Sepsis in Obstetrics Score to Identify Risk of Morbidity from Sepsis in Pregnancy. Obstetric Anesthesia Digest 2018; 38 (1).
31. Bowyer L., Robinson H.L., Barrett H., Crozier T.M., Giles M., Idel I., et al. SOMANZ guidelines for the investigation and management sepsis in pregnancy. Aust. N. Z. J. Obstet. Gynaecol. 2017;57:540–51.
32. World Health Organization. Guideline: Managing Possible Serious Bacterial Infection in Young Infants When Referral is not Feasible. 2015. (Retrieved August 18, 2020 from https://apps.who.int/iris/bitstream/handle/10665/181426/9789241509268_eng.pdf)
33. Pasha O, McClure EM, Saleem S, Tikmani SS, Lokangaka A, Tshefu A, et al. A prospective cause of death classification system for maternal deaths in low and middle-income countries: results from the Global Network Maternal Newborn Health Registry. BJOG. 2018;125(9):1137-43.
34. Garces AL, McClure EM, Pérez W, Hambidge KM, Krebs NF, Figueroa L, et al. The Global Network Neonatal Cause of Death algorithm for low-resource settings. Acta Paediatr. 2017;106(6):904-11.
35. Goudar SS, Goco N, Somannavar MS, Kavi A, Vernekar SS, Tshefu A, et al. Institutional deliveries and stillbirth and neonatal mortality in the Global Network's Maternal and Newborn Health Registry. Reprod Health. 2020 Dec 17;17(Suppl 3):179.
36. Hoffman MK, Goudar SS, Kodkany, B. S., Goco, N., Koso-Thomas, M., Miodovnik, M., et al. A description of the methods of the aspirin supplementation for pregnancy indicated risk reduction in nulliparas (ASPIRIN) study. BMC Pregnancy and Childbirth 2017; 17(1), 135.
37. Roca A, O. C., Bojang A, Camara B, Kampmann B, Bailey R, Demba A, Bottomley C, D'Alessandro U. (2016). Oral azithromycin given during labour decreases bacterial carriage in the mothers and their offspring: a double-blind randomized trial. *Clin Microbiol Infect. , 22*(6), e1-9.
38. Roberts, S., Maccato, M., Faro, S., & Pinell, P. (1993). The microbiology of post-cesarean wound morbidity. *Obstetrics and Gynecology, 81*(3), 383-386.
39. Rosene, K., Eschenbach, D. A., Tompkins, L. S., Kenny, G. E., & Watkins, H. (1986). Polymicrobial early postpartum endometritis with facultative and anaerobic bacteria, genital mycoplasmas, and Chlamydia trachomatis: treatment with piperacillin or cefoxitin. *Journal of Infectious Diseases, 153*(6), 1028-1037.
40. Andrews, W. W., Shah, S. R., Goldenberg, R. L., Cliver, S. P., Hauth, J. C., & Cassell, G. H. (1995). Association of post-cesarean delivery endometritis with colonization of the chorioamnion by Ureaplasma urealyticum. *Obstetrics and Gynecology, 85*(4), 509-514. https://doi.org/10.1016/0029-7844(94)00436-H
41. Keski-Nisula, L., Kirkinen, P., Katila, M. L., Ollikainen, M., Suonio, S., & Saarikoski, S. (1997). Amniotic fluid U. urealyticum colonization: significance for maternal peripartal infections at term. *American Journal of Perinatology, 14*(3), 151-156.

For peer review only - http://bmjopen.bmj.com/site/about/guidelines.xhtml

ACKNOWLEDGEMENTS

The authors would like to acknowledge the A-PLUS research teams for their many contributions to this work: *Kinshasa School of Public Health, Kinshasa, Democratic Republic of Congo*: Michel Kalonji and Miyalu Junior; *University of North Carolina, Chapel Hill, North Carolina, United States*: Jackie Patterson; *University Teaching Hospital, Lusaka, Zambia:* Ernest Banda, Mwansa Chimfwembe, Ruth Nakazwe, and Abigail Mwapule; *Instituto de Nutrición de Centroamérica y Panamá, Guatemala City, Guatemala*: Maynor Manrique; *University of Colorado School of Medicine, Denver, CO*, USA: Jamie Westcott; *International Centre for Diarrhoeal Disease Research, Dhaka, Bangladesh*: Qazi Sadeq-ur-Rahman, Amita Farzana, Farhana Jahan, Zarin Tasnim Maliha, and Lolit Singh; *University of Virginia, Charlottesville, Virginia, United States:* Chris Chisolm; *KLE Academy Higher Education and Research, J N Medical College Belagavi, Karnataka, India:* Sheetal Harakuni, Manjunath Somannavar, and Kadappa Beniwadi; *Thomas Jefferson University, Philadelphia, PA, USA:* Frances Jaeger; *Aga Khan University, Karachi, Pakistan:* Zaheer Habib, Imran Ahmed, Farnaz Naqvi, Naija Ghanchi, and Saleem Jessani; *Lata Medical Research Foundation, Nagpur, India:* Vaishali Khedikar, Savita Bhargav, Samreen Sadaf, and Chaitali Gedam; Moi University School of Medicine, Eldoret, Kenya; Milsort Kemboi, Anderson Misati, and Kevin Otieno; *Indiana School of Medicine, University of Indiana, Indianapolis, IN, USA*: Sheri Bucher; *RTI International, Research Triangle Park, North Carolina, United States:* Anna Aceituno, Kay Jackson, Jean Kim, Janet Moore, Suchita Parepalli, Marissa Trotta, and Alexis Williams.

Bill & Melinda Gates Foundation– Laura Lamberti

AUTHOR'S CONTRIBUTIONS

AT and WAC conceived of the study and developed the protocol, with input from EMM, TLN, JHF, MKT, and PLH. TLN developed the statistical analyses plan; JHF wrote the first draft of the manuscript, revised, and reviewed the final version with WAC, AT, EMM, MKT, and TLN. EC, MM, TM, AL, ATK, GL, PLH, AP, PD, KK, SSG, AK, MM, SS, SST, FE, PN, AS, LF, MM, SMB, RH, MSS, RLG, MB, CLB, EAL, OAE, NFK, RJD, and WAP provided review, refinement, and approval of the study protocol and the manuscript.

FUNDING STATEMENT

The A-PLUS study is supported through institutional grants from the *Eunice Kennedy Shriver* National Institute of Child Health and Human Development (NICHD) [RTI International (U01 HD040636), University of North Carolina at Chapel Hill (U10 HD076465), University of Alabama at Birmingham (U10 HD078437), University of Colorado (U10 HD076474), Thomas Jefferson University (U10 HD076457), Columbia University (U10 HD078438), Boston University (U10 HD078439), Indiana University (U10 HD076461)] and a grant from the Foundation for the National Institutes of Health [MCCL19APT] through the Maternal, Newborn & Child Health Discovery & Tools initiative of the Bill & Melinda Gates Foundation (BMGF) [INV-008973]. The views expressed in this manuscript are those of the authors and do not necessarily represent the views of the NICHD; the National Institutes of Health; or the U.S. Department of Health and Human Services or the BMGF.

COMPETING INTERESTS STATEMENT

The authors declare that they have no competing interests.

For peer review only - http://bmjopen.bmj.com/site/about/guidelines.xhtml

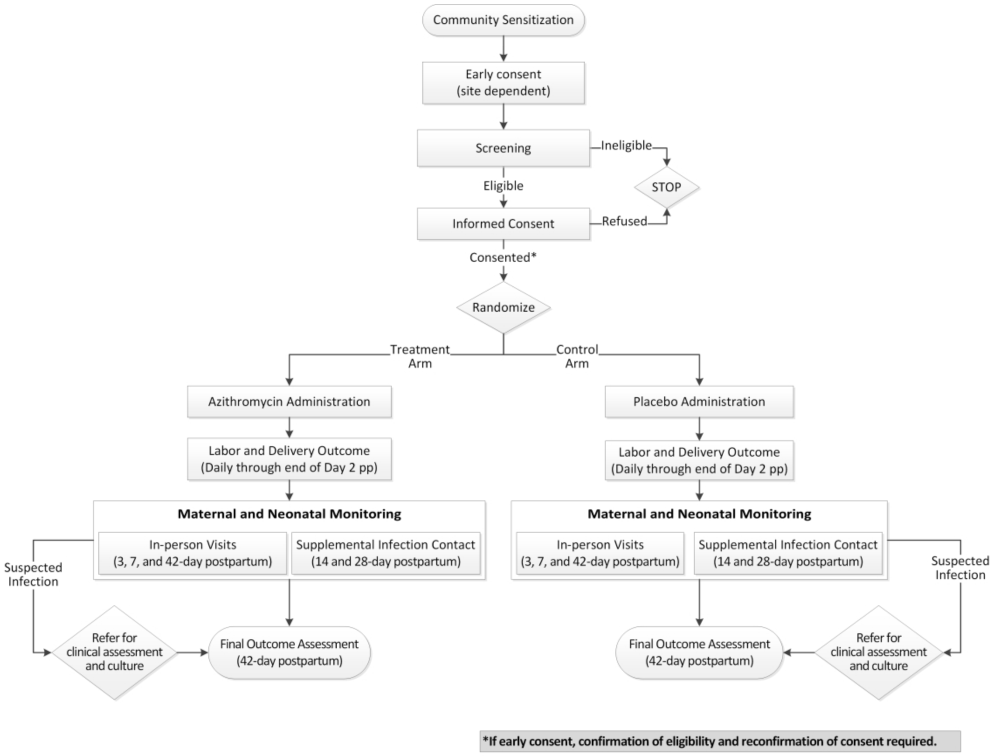

Figure 1. Study Flowchart

772x580mm (118 x 118 DPI)

Prevention of maternal and neonatal death/infections with a single oral dose of azithromycin in women in labor (in low- and middle-income countries): A randomized controlled trial

ClinicalTrials.gov number: NCT03871491

Lead Study Investigator(s): Alan T.N. Tita, MD, PhD, Center for Women's Reproductive Health and Maternal-Fetal Medicine Division, UAB
Wally Carlo, MD, Neonatology Division, UAB
Elwyn Chomba, MBChB, DCH, MRCP, University Teaching Hospital

Subcommittee Members: Marion Koso-Thomas, MD, NICHD
Tracy Nolen, DrPh, RTI International
Elizabeth McClure, PhD, RTI International
Jennifer J. Hemingway-Foday, MPH, RTI International

Version Number: Version 1.6

Version Date: July 15, 2022

Funding: Eunice Kennedy Shriver National Institute of Child Health and Human Development and The Bill & Melinda Gates Foundation

For peer review only

For peer review only - http://bmjopen.bmj.com/site/about/guidelines.xhtml

Version Tracking

Version	Date	Authors	Comments
1.0	Sep 27, 2018	A-PLUS WG	This version will be used for IRB/ERC submissions.
1.1	Dec 19, 2018	A-PLUS WG	Internal version to document and review proposed revisions from GN investigators.
1.2	Dec 20, 2018	A-PLUS WG and GN Investigators	At request of GN investigators, revisions made to the following sections: Inclusion and exclusion criteria (Section 2.2); Consent procedures (Section 2.4); Description of data forms (Section 4.1); Sample informed consent form (Appendix 2); and in-text references.<br>**This version will be used for IRB/ERC submissions after December 20, 2018.**
1.3	January 24, 2020	A-PLUS WG	Revisions made based on feedback from pilot study and requests from GN investigators. The following sections have been revised:<br>• Section 1 (Statement of Problem), including 1.2 and 1.3 (Primary and Secondary Specific Aims) and 1.5 (Rationale/Justification)<br>• Section 2 (Methods), including 2.1 (Inclusion Criteria); 2.4.7 (Monitoring after Discharge); 2.5 (Primary Outcomes); 2.6 (Other Maternal Outcomes); 2.7 (Other Neonatal Outcomes) and 2.8 (Safety Monitoring)<br>• Section 3 (Analytical Plan)<br>• Section 4.1 (Data Forms)<br>• Section 5 (References)<br>• Figure 1. Flowchart of Study Activities<br>• Appendix 2. Sample Informed Consent<br>• Appendix 3. Schedule of Study Procedures
1.4	January 29, 2020	A-PLUS WG	<u>Additional revisions made to:</u><br>• Section 1.3 (Secondary Specific Aims)<br>• Section 2.5 (Primary outcomes)<br>• Section 2 (Methods), including 2.6 (Other Maternal Outcomes) and 2.7 (Other Neonatal Outcomes)<br>• Section 3 (Analytical Plan, including 1.2 (Secondary Analysis-Women at High Risk for Infection Cohort) 3.2.1 (Sample Size for Primary Outcome) and 3.2.2 (Sample Size for High-risk Women)<br>**This version will be submitted to IRBs/ERCs at all GN sites.**
1.5	February 09, 2021	A-PLUS WG	<u>Updates made based on recommendations from the Data Monitoring Committee:</u><br>• In Section 2.5 (Primary Outcome), removed urinary tract infection, omphalitis, eye infection, and skin infection from neonatal sepsis definition, removed fast breathing from the pSBI definition, and changed the pSBI fever criteria to 38 C/100.4 F.<br>• In Section 2.7 (Other Neonatal Outcomes), added urinary tract infection, omphalitis, and fast breathing to the "other neonatal infection" outcome.<br><u>Other updates:</u><br>• In Section 3.5.1 (Reporting Adverse Events), made minor text changes to align with revised AE reporting procedures. [Refer to A-PLUS Technical Memo #2]<br>• In Section 3.5.4 (Interim/Adverse Event Monitoring Plan), made minor text revisions to clarify that the interim analysis will be conducted in both the all-comers and high-risk cohort. This revision was made at the request of the Boston University IRB.<br>• In list of Azithromycin Investigators, updated SFI for GN06-Guatemala to Manolo Mazariegos.<br>**This version will be submitted to IRBs/ERCs at all GN sites.**
1.6	12 July, 2022	A-PLUS WG	High-risk definition updated to "Prolonged labor [≥18 hours] and/or prolonged membrane rupture [≥8 hours]". The following sections have been updated to reflect this change:<br>• Abstract-*Secondary Outcomes*<br>• Section 1.3 (Secondary Specific Aims)<br>• Section 1.5 (Rationale/Justification)<br>• Section 1.6.1 (The Gambian and Cameroon Trials) *[Note: new reference added to this section, with full citation in reference list; as a result, all subsequent references have been renumbered]*<br>• Section 2.2.2 (Exclusion Criteria)<br>• Section 3 (Analytical Plan)<br>• Section 3.1.2 (Secondary Analysis-Women at High Risk for Infection Cohort)<br>• Section 3.2.2 (Sample Size for High-risk Women)<br>• Appendix 2 (Sample Consent)<br>Other minor updates were made to the list of "Azithromycin Investigators" to reflect staffing changes since protocol V. 1.5 was released.<br>**This version will be submitted to IRBs/ERCs at all GN sites.**

For peer review only - http://bmjopen.bmj.com/site/about/guidelines.xhtml

Table of Contents

Azithromycin Investigators 6

ACRONYMS 9

Abstract 10

1 Statement of Problem 11

1.1 Primary Hypotheses 11

1.2 Primary Specific Aims 11

1.3 Secondary Specific Aims 11

1.4 Background 12

1.4.1 Prevention of Maternal and Neonatal Deaths from Infections 12

1.4.2 Risks for Maternal and Neonatal Infections 12

1.4.3 Intrapartum Azithromycin to Prevent Maternal and Neonatal Infection 13

1.5 Rationale/Justification 13

1.6 Previous Studies 14

1.6.1 The Gambian and Cameroon Trials 14

1.6.2 The US Trial and Cost Analysis 14

1.6.3 Other Important Considerations 15

2 Methods 16

2.1 Study Design 17

2.2 Study Population/Location 17

2.2.1 Inclusion Criteria 17

2.2.2 Exclusion Criteria 17

2.3 Study Intervention and Comparison 18

2.4 Detailed Study Procedures 18

2.4.1 Community Sensitization 18

2.4.2 Screening 18

For peer review only - http://bmjopen.bmj.com/site/about/guidelines.xhtml

2.4.3 Consent ... 18
2.4.4 Masking ... 19
2.4.5 Randomization Procedures ... 20
2.4.6 Monitoring before Discharge ... 20
2.4.7 Monitoring after Discharge ... 20
2.4.8 Sequence of Study Activities ... 21
2.5 Primary Outcomes ... 22
2.6 Other Maternal Outcomes ... 22
2.7 Other Neonatal Outcomes ... 23
2.8 Safety Monitoring ... 23
2.9 Site Preparation ... 24
2.10 Potential Risks and Benefits to Participants ... 24
3 Analytical plan ... 25
3.1 Statistical Analysis Plan ... 25
3.1.1 Primary Analyses ... 25
3.1.2 Secondary Analyses – Women at High Risk for Infection Cohort ... 26
3.1.3 Secondary Analyses – Other Secondary Outcomes ... 26
3.2 Sample Size ... 27
3.2.1 Sample Size for Primary Outcome ... 27
3.2.2 Sample Size for High-Risk Women ... 28
3.3 Available Population ... 29
3.4 Projected Recruitment Time ... 29
3.5 Study Monitoring Plan ... 29
3.5.1 Reporting Serious Adverse Events ... 29
3.5.2 Method and Timing for Reporting Serious Adverse Events ... 30
3.5.3 Data Monitoring Plan and Stopping Rules ... 30
3.5.4 Interim/Adverse Event Monitoring Plan ... 31
3.5.5 Risks/Benefits ... 32
3.6 Quality Control ... 32

For peer review only - http://bmjopen.bmj.com/site/about/guidelines.xhtml

3.6.1 Training ... 32

3.6.2 Study Monitoring ... 32

3.6.3 Drug Quality Assurance and Monitoring ... 32

3.6.4 Plan for Sustaining Intervention ... 33

4 Data Management Procedures ... 33

4.1 Data Forms ... 33

5 References ... 35

Appendix 1. Description of Participating Global Network Sites ... 38

Appendix 2. Sample Informed Consent ... 40

Appendix 3. Schedule of Study Procedures ... 44

For peer review only

For peer review only - http://bmjopen.bmj.com/site/about/guidelines.xhtml

AZITHROMYCIN INVESTIGATORS

Central Team: Global Network Site 03 (Zambia)

Waldemar A. Carlo, MD
Principal Investigator
University of Alabama at Birmingham
Birmingham, Alabama
wcarlo@peds.uab.edu

Alan T.N. Tita, MD, PhD
Azithromycin Protocol Lead Investigator
University of Alabama at Birmingham
Birmingham, Alabama
atita@uabmc.edu

Elwyn Chomba, MBChB, DCH, MRCP
Senior Foreign Investigator
University Teaching Hospital
Lusaka, Zambia
chombaelwyn@gmail.com

Site Investigators

Global Network Site 02 (Democratic Republic of Congo)

Carl Bose, M.D.
Principal Investigator
University of North Carolina School of Medicine
Chapel Hill, North Carolina
cbose@med.unc.edu

Antoinette Tshefu, MD, PhD, MPH
Senior Foreign Investigator
Kinshasa School of Public Health
antotshe@yahoo.com

Global Network Site 03 (Zambia)

Wally Carlo, MD
Principal Investigator
University of Alabama at Birmingham
Birmingham, Alabama
Wcarlo@PEDS.UAB.EDU

Elwyn Chomba, MBChB, DCH, MRCP
Senior Foreign Investigator
University Teaching Hospital
Lusaka, Zambia
chombaelwyn@gmail.com

Global Network Site 06 (Guatemala)

Nancy Krebs, MD
Principal Investigator
UCHSC
Denver, Colorado
Nancy.krebs@ucdenver.edu

For peer review only - http://bmjopen.bmj.com/site/about/guidelines.xhtml

Manolo Mazariegos, MD
Senior Foreign Investigator
INCAP
Guatemala City, Guatemala
mmazariegos@incap.int

Global Network Site 07 (Bangladesh)

William Petri, MD
Principal Investigator
University of Virginia
Charlottesville, Virginia
wap3g@virginia.edu

Rashidul Haque, MD
Senior Foreign Investigator
ICDDR,B
Dhaka, Bangladesh
rhaque@icddrb.org

Global Network Site 08 (Belagavi, India)

Richard Derman, MD, MPH
Principal Investigator
Thomas Jefferson University
Philadelphia, PA
richard.derman@jefferson.edu

Shivaprasad S. Goudar MD, MHPE
Senior Foreign Investigator
KLE University's J N Medical College
Belgaum, India
sgoudar@jnmc.edu

Global Network Site 09 (Pakistan)

Robert L. Goldenberg, MD
Principal Investigator
Columbia University
New York, New York
rlg88@columbia.edu

Sarah Saleem, MD
Senior Foreign Investigator
Aga Khan University
Karachi, Pakistan
sarah.saleem@aku.edu

Global Network Site 11 (Nagpur, India)

Patricia L. Hibberd, MD, PhD
Principal Investigator
Boston University
Boston, Massachusetts
plh0@bu.edu

Archana Patel, MD, DNB, MSCE, PhD
Senior Foreign Investigator

For peer review only

Lata Medical Research Foundation
Nagpur, India
Dr_apatel@yahoo.com

Global Network Site 12 (Kenya)

Ed Liechty, MD
Principal Investigator
Indiana University School of Medicine
Indianapolis, Indiana
eliecht@iupui.edu

Fabian Esamai, MBChB, MMed, MPH, PhD
Senior Foreign Investigator
Moi University School of Medicine
Eldoret, Kenya
fesamai2007@gmail.com

National Institute of Child Health and Human Development (NICHD)

Marion Koso-Thomas, MD, MPH
Medical Officer, Global Network for Women's and Children's Health
Eunice Kennedy Shriver National Institute of Child Health and Human Development (NICHD)
kosomari@mail.nih.gov

RTI International

Elizabeth McClure, PhD
Principal Investigator, Data Coordinating Center
RTI International, Durham, NC
mcclure@rti.org

Tracy Nolen, DrPh
Senior Statistician, Data Coordinating Center
tnolen@rti.org

Jennifer J. Hemingway-Foday, MPH, MSW
Protocol Manager for Azithromycin Protocol, Data Coordinating Center
hemingway@rti.org

For peer review only

For peer review only - http://bmjopen.bmj.com/site/about/guidelines.xhtml

ACRONYMS

**AC**	All-comers
**ACOG**	American Congress of Obstetricians and Gynecologists
**ANC**	Antenatal Care
**BP**	Blood pressure
**DCC**	Data Coordinating Center
**DMC**	Data monitoring committee
**DMS**	Data Management System
**DRC**	Democratic Republic of Congo
**ERC**	Ethical review Committee
**FIGO**	International Federation of Gynecology and Obstetrics
**FDA**	Food and Drug Administration
**GA**	Gestational age
**GI**	Gastrointestinal
**GN**	Global Network for Women's and Children's Health Research
**HR**	High-risk
**IRB**	Institutional Review Board
**JAMA**	Journal of the American Medical Association
**ITT**	Intention to treat
**LIC**	Low-income country
**LMIC**	Low- and middle-income countries
**LMP**	Last menstrual period
**MNH**	Maternal and Newborn Health
**NEJM**	New England Journal of Medicine
**NICHD**	Eunice Kennedy Shriver National Institute of Child Health and Human Development
**NIH**	National Institutes of Health
**NNT**	Number needed to treat
**OHRP**	U.S. Office of Research Protections
**PI**	Principal Investigator
**pSBI**	possible serious bacterial infection
**RCT**	Randomized controlled trial
**RTI**	Research Triangle Institute International
**SAE**	Serious adverse event
**SC**	Steering Committee
**SFI**	Senior Foreign Investigator
**SMFM**	Society for Maternal-Fetal Medicine
**UAB**	University of Alabama at Birmingham
**WHO**	World Health Organization

For peer review only

For peer review only - http://bmjopen.bmj.com/site/about/guidelines.xhtml

ABSTRACT

Background: Maternal and neonatal infections are among the most frequent causes of maternal and neonatal deaths, and current antibiotic strategies have not been effective in preventing many of these deaths. Recently, a randomized clinical trial conducted in a single site in The Gambia showed that treatment with oral dose of 2 g azithromycin vs. placebo for all women in labor reduced certain maternal and neonatal infections. However, it is unknown if this therapy reduces maternal and neonatal sepsis and mortality.

Hypotheses: The trial includes two primary hypotheses, a maternal hypothesis and a neonatal hypothesis. First, a single, prophylactic intrapartum oral dose of 2 g azithromycin given to women in labor will reduce maternal death or sepsis. Second, a single, prophylactic intrapartum oral dose of 2 g azithromycin given to women in labor will reduce intrapartum/neonatal death or sepsis.

Study Design Type: Randomized, placebo-controlled, parallel multicenter clinical trial. Women in labor will be randomized with one-to-one ratio to intervention/placebo.

Population: Pregnant women in labor at ≥28 weeks gestational age with a live fetus pregnancy who plan to deliver vaginally in a facility. Women with evidence of chorioamnionitis or other infection requiring antibiotic therapy (not prophylaxis) at time of eligibility, allergy to azithromycin, use of azithromycin, erythromycin or other macrolide within 3 days of enrollment, known arrhythmia or cardiomyopathy, or plan for cesarean section delivery prior to enrollment will be excluded.

Intervention: A single, prophylactic intrapartum oral dose of 2 g azithromycin.

Comparison: A single intrapartum oral dose of an identical appearing placebo

Outcomes:
Primary outcomes: 1) Incidence of maternal death or sepsis and 2) incidence of intrapartum/neonatal death or sepsis.

Secondary outcomes: Individual components of the primary outcomes (maternal death, maternal sepsis, intrapartum/neonatal death, neonatal sepsis), neonatal death/sepsis, neonatal deaths due to sepsis, and all-cause neonatal death; the primary maternal and neonatal outcome in a high-risk for infection population; specific maternal infections; use of subsequent maternal antibiotic therapy; pyloric stenosis; health care resource utilization; culture-positive infections and resistance; in an ancillary surveillance study, incidence of antimicrobial resistance and microbiome diversity.

For peer review only

For peer review only - http://bmjopen.bmj.com/site/about/guidelines.xhtml

1 Statement of Problem

1.1 Primary Hypotheses

This study will have two primary hypotheses, one with a maternal focus and one with a neonatal focus. First, a single, prophylactic intrapartum oral dose of 2 g azithromycin given to women in labor in low and middle-income settings will reduce maternal death or sepsis. Second, a single, prophylactic intrapartum oral dose of 2 g azithromycin given to women in labor in low and middle-income settings will reduce intrapartum/neonatal death or sepsis.

1.2 Primary Specific Aims

To test the effectiveness of a single dose of prophylactic intrapartum azithromycin compared to placebo in reducing the risk of the composite outcome of maternal death or sepsis. To separately test the effectiveness of a single oral dose of intrapartum azithromycin prophylaxis (2 g) compared to placebo in reducing the risk of the composite outcome of intrapartum/neonatal death or sepsis. Both groups will receive the routine or usual care provided at the facility during and after labor.

1.3 Secondary Specific Aims

The single dose of intrapartum azithromycin prophylaxis (2 g) will be compared to placebo to accomplish the following secondary aims:

- Main Secondary Aim for Women at High-Risk for Infection Cohort:
    a. To evaluate whether the risk of maternal death or sepsis differs among laboring women with and without high-risk for infection (high risk is defined as women with prolonged labor [≥18 hours] and/or prolonged membrane rupture [≥8 hours] at time of randomization).
- Other secondary specific Aims:
    b. To evaluate the effectiveness in reducing the risk of individual components of the primary composite endpoints (i.e., maternal death, maternal sepsis, intrapartum/neonatal death, neonatal sepsis including specifically all-cause neonatal deaths and neonatal deaths due to sepsis).
    c. To evaluate the effectiveness on the risk of intrapartum/neonatal death or sepsis in infants of laboring women at high-risk for infection because of prolonged labor (≥18 hours) and/or prolonged membrane rupture (≥8 hours).
    d. To evaluate the effectiveness on the risk of maternal infections including clinical chorioamnionitis, endometritis, wound infections (perineal or Cesarean), abdominal or pelvic abscess, mastitis/breast abscess or infection, pyelonephritis, pneumonia and other bacterial infections in all laboring women as well as in those at high-risk for infection.
    e. To evaluate the effectiveness in reducing the use of subsequent maternal antibiotic therapy from randomization to 42 days postpartum for any reason in all laboring women as well as in those at high-risk for infection.
    f. To compare the use of health care resources. Use of health care resources will be measured in terms of maternal and neonatal duration of hospital stay, unscheduled visits, readmission, and admission to special care units/intensive care units in all laboring women and newborns as well as in those at high-risk for infection.
    g. To assess maternal GI symptoms (e.g., nausea, vomiting, and diarrhea) and other reported side effects, as well as infant pyloric stenosis and type of culture positive infections

For peer review only - http://bmjopen.bmj.com/site/about/guidelines.xhtml

(antimicrobial resistance and maternal and infant microbiome diversity are compared in an ancillary study).

h. To determine whether the effect on each of the primary outcomes differs by region (Africa, Latin America or Asia), any other antibiotic use during labor, and mode of delivery (cesarean or vaginal). Note that antibiotic use will not include use to treat any infections diagnosed after randomization as those will be outcomes.

1.4 Background

Maternal and neonatal infections are among the most frequent causes of maternal and neonatal death. Maternal infection during pregnancy and the puerperium account for approximately 10% of the global burden of maternal deaths. This places maternal infection among the top five causes of maternal mortality worldwide.[1] Neonatal infection is the third most common cause of neonatal mortality and accounts for about 16% of neonatal mortality worldwide.[2] Furthermore, maternal and neonatal deaths from infections are not decreasing compared with deaths from other frequent causes of mortality.[1,2] There is a need for innovative simple effective interventions that can be scaled up to reduce the burden of both maternal and neonatal mortality due to infections.

1.4.1 Prevention of Maternal and Neonatal Deaths from Infections

Current strategies to prevent maternal and neonatal deaths from infections are insufficient. Current approaches to prevention, identification, and treatment of neonatal sepsis have had limited impact. According to the WHO, maternal deaths from infection have remained unchanged or increased in some instances, whereas deaths from other causes have reduced. The evidence backing current WHO guidelines for prevention and treatment of peripartum infections is generally graded as low or very low quality.[1] Efforts for early identification and treatment of neonatal infection are important but neonatal deaths due to infection continue to be very prevalent. Recent studies of alternative antibiotic regimens that do not require a full course of intravenous antimicrobial therapy have shown comparable effectiveness but have not reduced deaths from infections.[3-6] Among research priorities, a WHO guideline panel identified the evaluation of the role of routine prophylactic antibiotics in women who undergo a normal vaginal birth as well as in those at high risk for infection due to prolonged labor or membrane rupture.[1]

1.4.2 Risks for Maternal and Neonatal Infections

Maternal and neonatal infections by maternal risk including cesarean delivery. While the role of antibiotic prophylaxis for cesarean delivery is well-established, in many low-resource settings, many maternal infections occur after vaginal deliveries with high-risk for infections. Cesarean delivery especially after labor or membrane rupture is the strongest risk factor for maternal peripartum infection including endometritis, wound infection and sepsis (increasing risk by up to 5 to 10 times compared to vaginal delivery).[7] The fraction of maternal infection attributable to cesarean delivery is rather low because of the low cesarean delivery rate of 5-10% or lower, especially in the Africa region, compared with 20-30% or higher in the US and many high income countries.[8] Therefore, strategies that address maternal peripartum infection and sepsis in the developing world should focus as well on identifying and preventing (as well as treating) infection in women who have a vaginal birth, particularly those who are at high-risk. There is an increased risk of infection in women who undergo prolonged labor ≥18 hours (at least 2 fold) or membrane rupture ≥6 hours (at least 2-3 fold) compared to women who do not experience these risk factors.[1] These risk factors identify a large group of women who are at the highest

For peer review only - http://bmjopen.bmj.com/site/about/guidelines.xhtml

risk for maternal peripartum infections (including chorioamnionitis, endometritis, perineal wound infection and post-cesarean surgical site infections) and sepsis after a vaginal or cesarean delivery and also place newborns at increased risk for sepsis. However, even though 80-90% of pregnancies in labor are not at this highest-risk for infection depending on criteria used, they may account for about 50% of maternal and neonatal infections in LMICs. Thus, testing antibiotic strategies during labor in LMICs should include both the high-risk and lower risk women as highlighted in research priorities from the WHO guideline panel.[1]

Antibiotic Prophylaxis for Women who deliver by Cesarean section. The appropriate use of antibiotic prophylaxis for cesarean delivery and antiseptic agents are among the most effective preventive interventions as highlighted by the WHO guidelines for maternal peripartum infections.[1] Antibiotic prophylaxis, preferably prior to incision, in particular, among several strategies, effectively reduces the risk of infection and the associated high health care and personal costs.[1,9-11] However, the prevalence of cesarean sections is low, in many low-resource settings, particularly in sub-Saharan Africa, and thus, do not account for the large burden of the maternal and neonatal infections worldwide.

1.4.3 Intrapartum Azithromycin to Prevent Maternal and Neonatal Infection

Azithromycin: a novel approach to maternal and neonatal infections. A novel approach to maternal and neonatal infection is to target organisms that may be very frequent pathogens but that historically have not been the target of antimicrobial treatment.[12] A multicenter randomized clinical trial (RCT) of azithromycin prophylaxis added to the standard prophylactic regimen (cephalosporin) in women who underwent cesarean delivery following labor or membrane rupture for at least 4 hours in the US showed that maternal infection was reduced by about 50%.[13] A single center RCT in a low-income country (LIC) setting suggested that azithromycin prophylaxis may improve maternal and neonatal outcomes. In an RCT in The Gambia that included all women in labor, treatment with 2 g of azithromycin vs. placebo before delivery reduced maternal and neonatal infections.[14] Therefore, we propose to evaluate the effectiveness of a single oral dose of azithromycin as an intrapartum prophylactic agent for maternal and neonatal infection and death. We will also monitor the potential side effects of this intervention.

1.5 Rationale/Justification

Maternal infection and sepsis is a priority to reduce maternal and neonatal deaths. Compared to postpartum hemorrhage and preeclampsia/eclampsia, maternal infection has received less attention as a major cause of maternal death; proportionally it accounts for increasing deaths.[1] The WHO and other global health authorities identified maternal infection/sepsis as a priority problem to reduce maternal deaths. In addition, maternal infection significantly increases the risk of neonatal sepsis which is one of the leading causes of neonatal death in LICs.[15] Most recently, a NEJM perspective article highlighted the WHO resolution issuing a clarion call recognizing "Sepsis" as a global health priority.[16] Drawing from our findings on azithromycin prophylaxis for cesarean delivery in the US[13] and data from another preliminary trial in The Gambia, Africa,[14] we propose to evaluate the role of a single oral dose of azithromycin (plus usual care) to prevent maternal death or peripartum sepsis and intrapartum/neonatal death or sepsis in laboring women as well as the targeted sub-population of those at the highest risk for infection because they have prolonged labor (≥18 hours) and/or prolonged membrane rupture (≥8 hours).

Azithromycin is available as a generic agent with easy storage requirements. It has a bimodal half-life of up to 70 hours in the non-pregnant population. Although its pharmacokinetic characteristics are not as

For peer review only - http://bmjopen.bmj.com/site/about/guidelines.xhtml

well studied in the pregnant population, it is commonly used during pregnancy for treatment of chlamydia and other infections. Azithromycin covers a broad spectrum of bacteria (including gram-positive cocci, genital ureaplasmas and mycoplasmas, and certain gram-negative bacilli and anaerobes) that are associated with maternal infections which are often polymicrobial (chorioamnionitis, endometritis, and perineal/cesarean wound infection) and sepsis. In addition to the aforementioned organisms which may play a role in neonatal infection, azithromycin also has activity against Group B streptococcus which is a major cause of neonatal sepsis in developed countries and may be implicated in LICs and low-resources settings as well. Therefore, a successful prophylaxis intervention is likely to reduce infections and death and may also reduce health care costs and need for prolonged antibiotic therapy to treat infections which may be associated with resistance. Indeed, the recent WHO Guidelines on peripartum infection articulated the following among research priorities: what are the benefits of initiating prophylactic antibiotics among women undergoing uncomplicated vaginal birth and among those at high risk such as after prolonged rupture of membranes?[1] A JHPIEGO consultative meeting on enhancing the focus on maternal infections suggested that "Attention to identification and prompt management of prolonged labor and prolonged rupture of membranes is critical to reduce disease and death due to maternal sepsis."[15,16,17]

1.6 Previous Studies

1.6.1 The Gambian and Cameroon Trials

Data from a single center trial in a LIC setting suggest the potential for azithromycin prophylaxis to improve maternal and neonatal outcomes. Among 829 Gambian mothers (randomized to 2 g of azithromycin vs. placebo before delivery) and their 830 newborns, maternal infections were lower in the azithromycin group (3.6% vs 9.2%; relative risk [RR], 0.40; 95% confidence interval [CI], 0.22-0.71; P = 0.002).[14] Among newborns, the overall prevalence of infections was also lower in the azithromycin group (18.1% vs 23.8%; RR, 0.76; 95% CI, 0.58-0.99; P = 0.052).[14] Maternal and neonatal carriage of infectious organisms was lower in the azithromycin group.[18] A test of concept trial focusing on high-risk women in Cameroon (labor ≥18 hours or membrane rupture ≥8 hours at randomization was underpowered by suggested lower maternal infection in women who received azithromycin.[19]

1.6.2 The US Trial and Cost Analysis

In a multicenter US RCT, it was demonstrated a further 50% reduction in the risk of maternal peripartum infection by adding a single 500 mg intravenous dose of azithromycin to the standard prophylactic regimen (a single intravenous dose of cefazolin 1-2 g or ampicillin) in the highest-risk group of women who undergo cesarean delivery following labor or membrane rupture for at least 4 hours.[13] These results were observed despite universal use of other antibiotics in both arms of the trial. Specifically, the 95% without penicillin allergy had a cephalosporin (mainly cefazolin) for usual cesarean prophylaxis; the remainder that gentamicin and clindamycin. In addition, 25-30% received a penicillin for GBS prophylaxis. Infection was significantly lower in the azithromycin group compared to the placebo group; 6.1% vs. 12%; RR =0.51 (95 % CI 0.38 to 0.68); p<0.001. Specifically, adjunctive azithromycin use was associated with significant reductions in the risks of endometritis (3.8% vs. 6.1%, RR=0.62, 95% CI: 0.42-0.92; p=0.02) and wound infections (2.4% vs. 6.6%, RR=0.35, 95% CI: 0.22-0.56; p<0.001). The number needed to treat (NNT) to prevent one infection was 17 for any infection, 43 for endometritis and 24 for wound infection. Other maternal outcomes including need for readmission or unscheduled visits for any reason or specifically for infection (decreased by up to 50%), serious adverse events, postpartum fever,

For peer review only - http://bmjopen.bmj.com/site/about/guidelines.xhtml

or subsequent treatment with antibiotics were also significantly less common with azithromycin prophylaxis. Short-term perinatal/infant outcomes including deaths, sepsis, and other serious neonatal morbidities were rare in this developed country population and did not differ between groups.[13] In a related cost-analysis report, it was estimated that use of adjunctive azithromycin saves approximately $360 for each use in unscheduled (high-risk) cesarean deliveries such as those studied in the RCT and $143 per use in scheduled or pre-labor cesarean delivery.[20] The results suggesting cost-savings were robust across wide ranges of baseline risk of infection and treatment effect size.[20]Thus, in the US alone, adjunctive azithromycin prophylaxis for cesarean delivery could lead to $350M in cost-savings/year due to avoided infection. These works built upon, and are supported by, over 20 years of research on maternal infections at UAB and elsewhere.[21-31] Azithromycin provides coverage against the most common pathogens identified in association with peripartum infections including genital mycoplasmas and ureaplasmas (when specific methods are utilized to identify them).

1.6.3 Other Important Considerations

A recurrent concern regarding the potential routine use of azithromycin in a large population is antibiotic resistance. Monitoring for characteristics suggestive of resistant infection will be important to incorporate in the protocol. However, a number of factors mitigate concerns about antibiotic resistance: (a) the design of the trial using a single prophylactic dose (as opposed to recurrent treatment doses) of antibiotic, (b) surveillance of maternal clinical cultures up to 6 weeks in the trial in cesareans in the US revealed that positive wound cultures overall and those positive for resistant organisms were significantly less frequent in the azithromycin group;[13] (c) if successful, prophylaxis will reduce the risk of infection and actually reduce the overall frequency of use of antibiotics to treat infection (a result in the US trial as stated above). It is estimated that women in the high-risk for infection group as defined will account for about 10-20% of laboring women. In principle, the use of single prophylactic dose (as opposed to multiple) minimizes the likelihood of antimicrobial resistance but samples will be taken to determine antimicrobial resistance.[32]

Azithromycin is currently recommended to treat or prevent several infections in pregnancy including gonorrhea (1 g po), chlamydia (1 g po), and Mycobacterium avium complex prophylaxis (600 mg twice/week or 1.2 g weekly po). Azithromycin is sometimes used for perioperative prophylaxis in patients at risk for endocarditis (500 mg po). Considering the success with a single dose of 2 g in the Gambian trial, our success with 500 mg IV for cesarean prophylaxis, and the 40% bioavailability of oral azithromycin, we propose to use 2 g po of azithromycin for the proposed intervention. The best approach to this evaluation in order to influence future uptake into clinical practice is an RCT. The primary maternal outcome will be maternal death or sepsis within 42 days after delivery. The primary neonatal outcome will be intrapartum/neonatal death or sepsis within 28 days after birth (defined by WHO criteria).[33]

Azithromycin is *pregnancy category B* – animal studies using maternally toxic doses showed no fetal harm. Limited studies suggest azithromycin is excreted in human milk in a sustained fashion.[34] There are no specific drug-drug interactions warranting dose adjustments when given with other medications. Elimination is by both hepatic and renal route, and no specific adjustments are mandated for patients with renal or hepatic insufficiency. The long elimination 1/2-life of 68 hours is due to extensive uptake and subsequent release of drugs from tissues. The only absolute contraindications are rare: known hypersensitivity reaction to azithromycin, erythromycin or other macrolide antibiotic or history of

For peer review only - http://bmjopen.bmj.com/site/about/guidelines.xhtml

cholestatic jaundice due to azithromycin. Potential adverse events include very rare (<1%) allergic hypersensitivity (mild and severe skin reactions – Stevens Johnson Syndrome and toxic epidermal necrolysis, angioedema and anaphylaxis) and clostridium difficile-associated diarrhea. With multi-day azithromycin therapy, 0.6% of patients discontinued azithromycin due to side effects. With single 1-2 gram doses, gastrointestinal symptoms (nausea 5-18%, diarrhea/loose stools 7-14%, abdominal pain 5-7%, vomiting 2-7%, and dyspepsia 1%) as well as vaginitis 2% and dizziness (1%) were the most commonly reported side effects. No other side effect occurred with a frequency greater than 1%.

Azithromycin has been associated with hypertrophic pyloric stenosis in some observational studies. In a retrospective study, oral azithromycin exposure during the first 14 days after birth was associated with an increased incidence of pyloric stenosis.[35] However, an increased risk of pyloric stenosis was not reported in infants following a single dose of azithromycin to women in labor in randomized trials in the US and Gambia.[13-14, 16] The much larger sample size of the proposed study affords the opportunity to further explore this question.

The FDA in 2013 issued an advisory regarding concerns about potential for rare life-threatening arrhythmias with azithromycin use particularly among those with preexisting cardiovascular risk. The information was based on an observational study of older, ill patients who received multiple courses of oral azithromycin over 5 days.[36] The findings are not applicable to our current study population for several reasons: the population is much younger, generally without cardiac co-morbidities, a single dose rather than cumulative doses of oral azithromycin over 5 days is being studied, additional studies of younger, healthy patients did not suggest an increased cardiovascular risk[37]; and patients with arrhythmia or known history of cardiomyopathy will be excluded. In addition, the prior study of 2013 women in the US did not suggest a cardiovascular safety signal, and the potential reduction in severe infection may exceed the excess risk of severe arrhythmia with a single dose of azithromycin.[13]

2 Methods

Pregnant women in labor at study health facilities who appear to be ≥28 weeks gestational age by best clinical estimate with a live fetus pregnancy will be screened for eligibility (see **Section 2.2**) by research staff starting at the time of admission and continuously during the hospitalization. Gestational age will be determined using the "best estimate" algorithm that is currently used in the GN MNH registry. Those eligible will be consented. Those consented will be randomized to receive a single dose of azithromycin 2 g or identical placebo to be given and directly observed during labor. Because of the ease of storage of azithromycin, sequentially prepared medications in identical packages following the randomization sequence will be available and dispensed by the study staff. The rest of the care will be provided according to the local standard of care. Women will be followed up until discharge and surveillance maintained (in-person), with visits at 3 days, 7 days, and 42 days after delivery as has generally been done in the Global Network to ascertain study outcomes.

We will apply the recently recommended "simple and actionable" WHO definition of sepsis in a 2017 statement endorsed by multiple international organizations. The definition of sepsis includes a suspicion of infection and the presence of organ dysfunction based on clinical findings.[15]

To further ensure more objective ascertainment of the sepsis outcome, we propose that the DCC will prospectively define a process for defining criteria for a masked centralized review and adjudication of all

For peer review only - http://bmjopen.bmj.com/site/about/guidelines.xhtml

cases of infection to ensure conformity with the proposed definition. This approach of adjudication is applied to validate infection in many trials including our recently completed ASPIRIN trial.[38] This approach to adjudication has some similarities to the near-miss classification for adjudication in women delivering in the Global Network sites that was done recently.[39]

2.1 Study Design

This study is a masked, placebo-controlled RCT. The investigational regimen is 2 g of azithromycin and the comparison arm is an identical placebo which is given orally. Both groups will also receive the standard of care during labor, delivery and postpartum per local standards.

2.2 Study Population/Location

Pregnant women laboring in health facilities of the eight Global Network sites/other health facilities will be eligible. The Global Network sites are described in **Appendix 1**. Health facilities may include any hospitals and health centers where women routinely deliver within the study sites.

2.2.1 Inclusion Criteria

- Pregnant women in labor ≥28 weeks GA (by best estimate) with a pregnancy with one or more live fetuses who plan to deliver vaginally in a facility.
- Admitted to health facility with clear plan for spontaneous or induced delivery.
- Live fetus must be confirmed via presence of a fetal heart rate prior to randomization.
- ≥18 years of age or minors 14-17 years of age in countries where married or pregnant minors (or their authorized representatives) are legally permitted to give consent.
- Have provided written informed consent [Note: written informed consent may be obtained during antenatal care, but verbal re-confirmation may be needed (per local regulations) at the time of randomization].

2.2.2 Exclusion Criteria

- Non-emancipated minors (as per local regulations)
- Evidence of chorioamnionitis or other infection requiring antibiotic therapy at time of eligibility (however, women given single prophylactic antibiotics with no plans to continue after delivery should not be excluded).
- Arrhythmia or known history of cardiomyopathy.
- Allergy to azithromycin or other macrolides that is self-reported or documented in the medical record.
- Any use of azithromycin, erythromycin, or other macrolide in the 3 days or less prior to randomization.
- Plan for cesarean delivery prior to randomization.
- Preterm labor undergoing management with no immediate plan to proceed to delivery.
- Advanced stage of labor (>6 cm or 10 cm cervical dilation per local standards) and pushing **or** too distressed to understand, confirm, or give informed consent **regardless** of cervical dilation.
- Are not capable of giving consent due to other health problems such as obstetric emergencies (for example, antepartum haemorrhage) or mental disorder.
- Any other medical conditions that may be considered a contraindication per the judgment of the site investigator.
- Previous randomization in the trial.

For peer review only - http://bmjopen.bmj.com/site/about/guidelines.xhtml

Sites may choose to obtain written consent during antenatal care from pregnant women of the age of consent who plan to deliver vaginally in a facility and have no known medical exclusions. In this case, study staff must fully assess and confirm eligibility at the time of randomization. It may also be necessary to verbally re-confirm consent at the time of randomization, if required by local regulations. If it is determined that the study needs to be extended to accrue the planned sample size for HR cohort, then women who deliver prior to meeting HR criteria for randomization will be excluded.

2.3 Study Intervention and Comparison

The study intervention is a single 2 g dose of directly observed oral azithromycin, to be administered as four 500 mg pills or tablets directly after randomization. By random allocation, participants will receive 2 g of oral azithromycin vs. placebo. We will design the placebo with the assistance of a reputable pharmacy/pharmaceutical company, using identical capsules or pills containing azithromycin 2 g or a matching placebo (non-antimicrobial agent) to accomplish masking. All of the participants' obstetric care will be similar for all both arms and consist of the routinely available care at each center.

2.4 Detailed Study Procedures

2.4.1 Community Sensitization

Local health providers will receive sensitization about the study to foster communication and collaboration at the facilities where enrollment will take place. In addition, pregnant women and their families in the enrollment area will receive information about the study during antenatal care (ANC) visits to facilitate recruitment and comprehension during the consenting process. Sites may choose to consent women during these sensitization sessions, prior to labor; however, confirmation of eligibility and consent will be required during the screening process described in **Section 2.4.2 and 2.4.3**.

2.4.2 Screening

Women in labor in defined health facilities (both hospitals and health centers) will be identified by research staff. A brief review of eligibility will be made to determine whether the patient is in labor and does not meet any of the exclusion criteria. If a contraindication to participation in the trial is found, the woman will be excluded from the trial at this point.

2.4.3 Consent

Before a woman participates in the trial, the research staff must obtain her informed consent to voluntarily take part in the study. Consent will be obtained from women ≥18 years of age or minors 14-17 years of age in countries where married or pregnant minors (or their authorized representatives) are legally permitted to give consent. When enrolling minors, we will follow the in-country policies for human research protection and the guidelines approved by the local ethical review committees (ERCs). In the case of pregnant minors, this may require that written consent is obtained from her parents/guardians or husband, with written assent from the minor.

Potential participants will be screened and enrolled in the study upon admission for delivery; therefore, consent will be obtained during labor. Consent should be obtained as early as possible during the intrapartum period and must be obtained prior to the cervical dilation limit approved by local authorities (e.g. either > 6 cm or 10 cm) and/or pushing, as assessed from clinical exam by health facility staff. It is not feasible to wait because the intervention must be given before delivery. Research sites

For peer review only - http://bmjopen.bmj.com/site/about/guidelines.xhtml

may choose to obtain initial consent during ANC; however, in this situation, confirmation of consent will be required during the screening and enrollment process.

If consent is obtained during labor, study staff will make necessary accommodations to ensure that the laboring women can comprehend the information presented during the consent process. Potential participants who present in labor can take as much time as needed to consider participation while in labor and will be able to discuss the study with family/friends if desired before deciding on participation. If the participant cannot read, the form will be read aloud to her by a person unaffiliated with the study. Alternatively, the Research Coordinator or a designate may read the consent, but in the presence of a witness who is unaffiliated with the research study. Potential participants will be given an opportunity to discuss the study procedures and ask questions. Additional details are provided in the study Manual of Procedures.

Fair balance will be maintained while describing the risks and benefits of participation in the study. No undue pressure will be placed on the potential participant to enroll in the trial. It will further be explained that lack of participation will not affect the usual and anticipated standard of care. As the literacy levels will vary and may be a challenge, the consent process will include a verbal review of the consent form.

After the potential participant has read the consent form, but before she signs, the research staff will show her a sample study pill and confirm that she is willing and able to take the study pill as prescribed. Only if she is willing to commit to taking the pill will she be enrolled; otherwise, this will be recorded as a refusal of consent. Following review of the consent, the potential participant (or parent/guardian) will be asked to sign the form. If the potential participant (or parent/guardian) is unable to sign her name, she will be asked to use her thumbprint to indicate written approval. In both cases, the unaffiliated person will also sign the consent form. Both the research staff and the study participant retain signed copies of the form.

An eligible woman may refuse to participate in the trial at the time of recruitment. This will be recorded in the Screening and Recruitment Form. She may also choose to withdraw from the study at any time after enrollment. This will be recorded on the Withdrawal/Termination Form.

All research staff responsible for obtaining consent will be trained and certified in the protection of human subjects and the study-specific consent procedures. A model written informed consent form, developed according to the requirements of the U.S. Office of Research Protections (OHRP), is found in **appendix 2**. Each site may modify the model consent to conform to local standards, but the OHRP required elements must be maintained. The research sites will also be responsible for translating the consent form into the appropriate language(s) for their local context.

Global Network countries with legislation regarding the need to videotape consents will comply with the country regulations; however, this is not part of the consent form requirements. This will not be required by protocol but rather decided by each site so as to comply with local rules and regulations.

2.4.4 Masking

Both the azithromycin and placebo will be procured from the same manufacturer. The packaging will be standardized across sites and will be labeled as: “Azithromycin 2 g or Placebo”, with the expiration data and a unique identifier. A certificate of authenticity will likewise be provided.

For peer review only - http://bmjopen.bmj.com/site/about/guidelines.xhtml

Clinical and research staff as well as the women will be masked to treatment status unless there is a serious adverse event potentially related to the treatment modality that requires unmasking for safety reasons. There will be one pharmacist at each site who will monitor randomization, drug supply, and safety. Under the instruction of the DCC, the study pharmacist will be trained and authorized to apply un-masking procedures, if concerns about randomization or participant safety are identified.

2.4.5 Randomization Procedures

Randomization of participants will be carried out to obtain a 1:1 allocation ratio between the treatment and placebo arms. Randomization will be stratified by site. A computer algorithm generated by the data coordinating center (DCC) will create the random assignment to one of the treatment arms based on randomly permuted block design with randomly varied block sizes. The block sizes will be known only by the DCC personnel. Each site will receive a lot of the study drug to be distributed sequentially at the participating health facilities which are randomizing women for the study site.

2.4.6 Monitoring before Discharge

Routine post-delivery care will be provided to participants by their clinical providers who will be masked to the study interventions. Research staff (also masked to study medications) trained in obstetric and perinatal outcomes abstraction will be responsible for collecting research data from participant medical records or directly from participants, as relevant, before discharge. Data needed to determine sepsis will include temperature (fever or hypothermia) essentially, plus one or more of heart rate (tachycardia), blood pressure (hypotension), respiratory rate (tachypnea or distress), clinical exam (jaundice or altered mental status) and urine output (low) and a suspicion of infection by the clinical provider or research team. Maternal and neonatal outcomes will be evaluated in the hospital following the delivery (on an ongoing basis until discharge). All participants will be asked to agree to maternal/infant medical record release to abstract information for outcome assessment as part of the consent form. In addition, participants will be educated about the signs and symptoms of infection and other study outcomes and encouraged to call the research team with any concerns.

2.4.7 Monitoring after Discharge

After discharge, study staff will contact participants at the following timepoints:

- In-person visits will be conducted at 3-day, 7-day, and 42-day postpartum to identify maternal or infant infection, unexpected medical visits, side effects, and other study outcomes. WHO criteria for infections will be used and included in the data collection forms. In the case of suspected infection, research staff will collect specimens (e.g. blood, urine, or pus from wound or drained abscesses), as is feasible, in order to identify individual infectious agents.
- Supplemental phone contacts will be conducted at 14-day and 28-day postpartum to review maternal and neonatal signs of infection using WHO criteria. If signs of infection are identified during the review, participants will be asked to visit a study facility for further assessment. These supplemental contacts will reinforce the participants' ability to self-assess for signs of maternal and neonatal infection and improve identification of infection between the 7-day and 42-day postpartum visits. If phone contact is not feasible, in-person visits may be conducted.

If indicated, records of unscheduled visits to any health facility prior to the routine study visits will be obtained and reviewed to ascertain study outcomes. Treating providers may also be called if clarification is needed. Based on the scheduled visits, study staff will identify any unscheduled visits or readmissions

For peer review only - http://bmjopen.bmj.com/site/about/guidelines.xhtml

that occurred in the interim. Readmissions and related diagnoses identified during follow-up visits will be then be validated through medical record review.

2.4.8 Sequence of Study Activities

The sequence of study activities is described in **Figure 1**. A detailed scheduled of study procedures is found in **Appendix 3**.

Figure 1. Flowchart of Study Activities

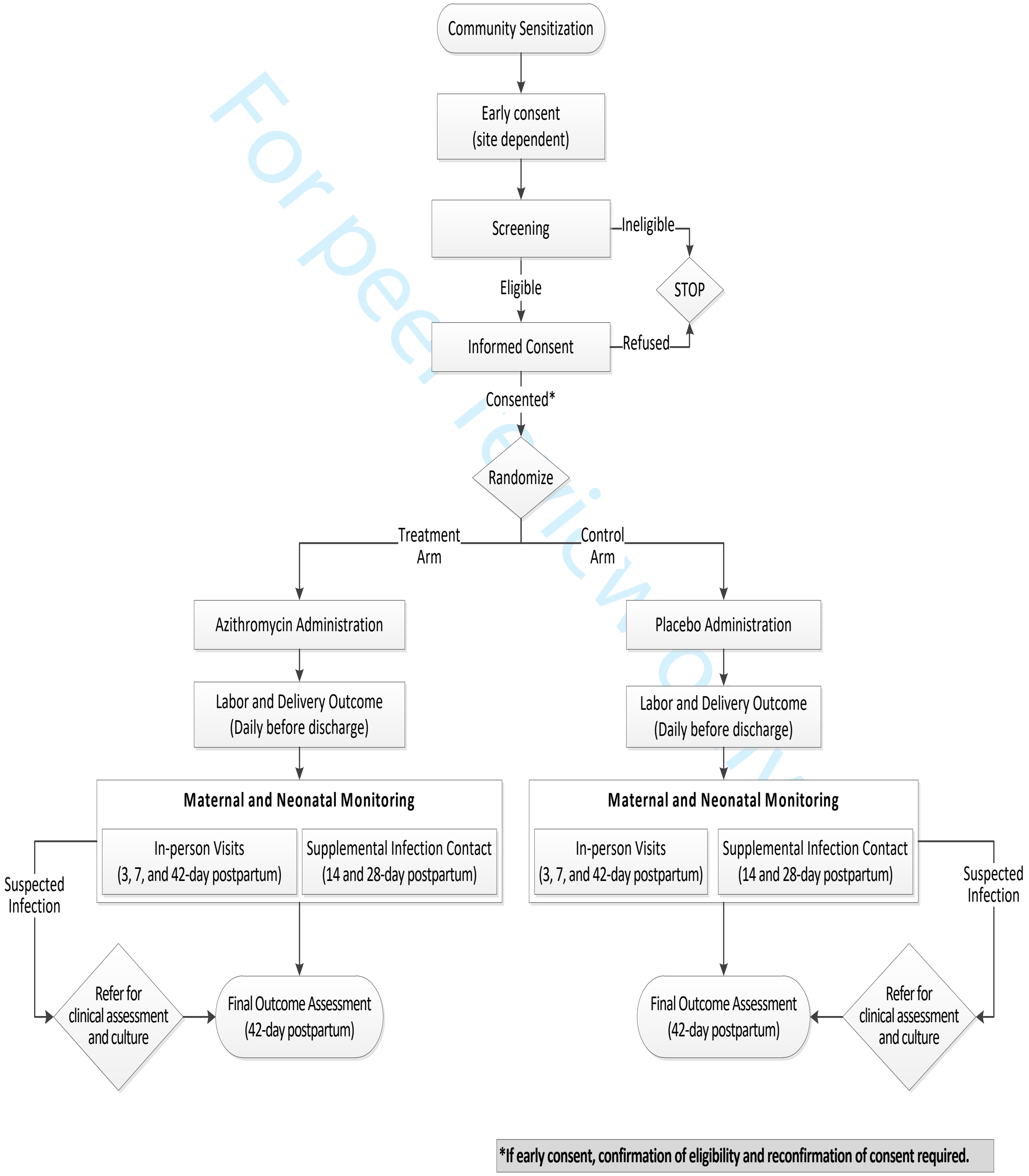

For peer review only - http://bmjopen.bmj.com/site/about/guidelines.xhtml

2.5 Primary Outcomes

The primary outcomes are:

- Maternal: Incidence of maternal death or sepsis within 6 weeks (42 days) post-delivery in intervention vs. placebo group.
- Neonatal: Incidence of intrapartum/neonatal death or sepsis within 4 weeks (28 days) post-delivery in intervention vs. placebo group.

Maternal sepsis is a life-threatening condition defined as organ dysfunction resulting from suspected or confirmed infection that occurs post-randomization during labor or the postpartum period. This WHO definition, endorsed by multiple global health organizations, will be operationalized as suspected or confirmed infection based on the presence of fever (>100.4° F/38° C) or hypothermia (<96.8° F/36° C) plus one or more signs of mild to moderate organ dysfunction including tachycardia (≥120 bpm), low BP (systolic <90 mm Hg), tachypnea (>24 breaths/min), altered mental status/confusion, reduced urinary output (<500 ml over 24 hours), jaundice, or renal failure (>1.2 mg/dl). [15],[40,41,42] Components of peripartum infection which will be considered in making the diagnosis of suspected or confirmed infection include clinical chorioamnionitis, endometritis, wound infections (perineal or Cesarean), abdominal or pelvic abscess, mastitis/breast abscess or infection, pyelonephritis, pneumonia, and other bacterial infection as defined in Section 2.6 below.

Neonatal sepsis is defined as a newborn with proven or possible serious bacterial infection (pSBI) or pneumonia, or meningitis. Possible serious bacterial infection will be determined using WHO criteria of pSBI defined as severe chest in-drawing, fever (temperature ≥ 100.4° F/38.0 °C), hypothermia (temperature <95.9°F/35.5 °C), no movement at all or movement only on stimulation, feeding poorly or not feeding at all, and/or convulsions.[33] Clinical and laboratory signs of infection will also be considered in making the diagnosis of suspected or confirmed infection.

Centralized masked adjudication of key infection outcomes will be implemented by the DCC and investigators during the trial to standardize and enhance the reproducibility of trial results. In addition to the above specified criteria, reported antibiotic treatment and culture status will also be considered as part of the adjudication process in making the diagnoses for both maternal and neonatal sepsis. Local site investigators will review and validate the diagnosis for all participants identified to have the primary outcomes applying pre-specified criteria. These reviews will be conducted masked to treatment group. A second investigator from a different site will review the participant's information and make a final outcome determination. If this does not agree with the determination of the local site investigator, then the adjudicating investigator will discuss with the local investigator to reach a consensus, failing which the study PIs will make the final call. This process will be coordinated by the DCC.

The individual components of these primary outcomes are also of interest and will be analyzed.

2.6 Other Maternal Outcomes

a. Chorioamnionitis: Fever (>100.4°F/38°C) in addition to one or more of the following: fetal tachycardia ≥160 bpm, maternal tachycardia >100 bpm, tender uterus between contractions, or purulent/foul smelling discharge from uterus **prior** to delivery.
b. Endometritis: Fever (>100.4°F/38°C) in addition to one or more of maternal tachycardia >100 bpm, tender uterine fundus, or purulent/foul smelling discharge from uterus **after** delivery.

For peer review only - http://bmjopen.bmj.com/site/about/guidelines.xhtml

c. Other infections: Wound infection refers to purulent infection (superficial or deep infection including necrotizing fasciitis) of a perineal or Cesarean wound with or without fever. In the absence of purulence, a wound infection requires presence of fever (>100.4°F/38°C) and at least one of the following signs of local infection: pain or tenderness, swelling, heat, or redness around the incision/laceration; abdominopelvic abscess is evidence of pus in the abdomen or pelvis noted during open surgery, interventional aspiration or imaging; pneumonia refers to fever (>100.4°F/38°C) and clinical symptoms suggestive of lung infection including cough and/or tachypnea (>24 breaths/min) or radiological confirmation; pyelonephritis refers to fever (>100.4°F/38°C) and one or more of the following: urinalysis/dip suggestive of infection, costovertebral angle tenderness, or confirmatory urine culture; Mastitis/breast abscess or infection refers to fever (>100.4°F/38°C) and one or more of the following: breast pain, swelling, warmth, redness, or purulent drainage.
d. Use of subsequent maternal antibiotic therapy after randomization to 42 days postpartum for any reason.
e. Time from drug administration until initial discharge after delivery (time may vary by site).
f. Maternal readmission within 42 days of delivery.
g. Maternal admission to special care units.
h. Maternal unscheduled visit for care.
i. Maternal GI symptoms including nausea, vomiting, and diarrhea and other reported side effects.
j. Maternal death due to sepsis using the Global Network algorithm for cause of death.

2.7 Other Neonatal Outcomes

a. Other neonatal infections (e.g., eye infection, skin infection, omphalitis, urinary tract infection, respiratory rate ≥60 breaths/minute).
b. Neonatal initial hospital length of stay, defined as time of delivery until initial discharge (time may vary by site).
c. Neonatal readmission within 42 days of delivery.
d. Neonatal admission to special care units.
e. Neonatal unscheduled visit for care.
f. Neonatal death due to sepsis using the Global Network algorithm for causes of death.
g. Pyloric stenosis within 42 days of delivery, defined as clinical suspicion based on severe vomiting leading to death, surgical intervention (pyloromyotomy) as verified from medical records, or radiological confirmation.

2.8 Safety Monitoring

Surveillance of maternal side effects including nausea, vomiting, and diarrhea/loose stools, abdominal pain, vaginitis, and dizziness potentially associated with azithromycin will be conducted during labor and postpartum. For infants, findings suggestive of pyloric stenosis will be assessed during the follow up visits. Maternal and neonatal surveillance will also include assessment of unintended medical visits, maternal deaths, stillbirths, neonatal death within 28 days of birth, and infant death after 28 days of birth. Additional maternal and neonatal risks associated with azithromycin use include anaphylaxis, allergic reactions (rash), liver failure, and arrhythmias. Although rare, these side effects will be monitored and reported as a serious adverse event. All safety outcomes will be reviewed at least twice a year by the Data Monitoring Committee (DMC) appointed by NICHD.

For peer review only - http://bmjopen.bmj.com/site/about/guidelines.xhtml

2.9 Site Preparation

In preparation for study implementation, the site investigators will meet with local health authorities and conduct community sensitization activities to ensure that study procedures are appropriate for the local context and to encourage commitment and engagement at the facility and community level. Site preparation activities will focus on:

- Disseminating study objectives to local health authorities and other stakeholders
- Identifying and hiring study staff;
- Developing site-specific procedures for safety monitoring procedures;
- Exploring locally-acceptable methods to monitor and improve follow-up visit compliance;
- Identifying potential implementation challenges and developing culturally-appropriate solutions;
- Training research staff in the implementation of the study procedures, follow-up and ascertainment of infections.

2.10 Potential Risks and Benefits to Participants

There are several potential direct and indirect benefits of this trial. In developing countries, including those of GN partners, fetal and neonatal deaths due to infections and maternal and neonatal infections are common. If intrapartum azithromycin reduces maternal and fetal/neonatal mortality or infections, many deaths could be reduced in the GN sites as well as worldwide.

Emerging data suggest that intrapartum azithromycin reduces maternal and neonatal infection. It is not known if deaths could be prevented but as infections are one of the most frequent causes of maternal and neonatal deaths, there is a possibility that mortality could be reduced.

An ongoing concern for peripartum and perinatal antibiotic prophylaxis is the selection of resistant organisms including azithromycin-resistant organisms leading to resistant infections, and there is concern that disruption of gut and other flora (microbiome) in women and particularly in neonates may lead adverse events including increased allergic reactions, rash and childhood asthma.[12,18,40] In the trial from Gambia of a single oral dose of azithromycin during labor, higher prevalence of colonization with S. aureus azithromycin resistance observed among women and their babies four weeks after treatment had waned 12 months later and azithromycin did not induce other antibiotic resistance to S. pneumoniae or S. aureus; resistance data from actual infections were not available. There was a 7% vs. 21% prevalence of any bacteria in breast milk in those receiving azithromycin vs. placebo [4-5]. In the US trial of 2013 of adjunctive azithromycin in 2013 women who underwent unscheduled cesarean delivery, culture positive maternal infections (1.4% vs. 3.6%) and infections with at least one resistant bacterial species (1% vs. 2.4%) were significantly less frequent in the azithromycin group. Azithromycin-resistant organisms were identified in only 7 (3 vs. 4) participants. There is a paucity of data to address theoretical concerns that disturbances in the establishment of the indigenous intestinal microbiome caused by antibiotic exposure in early life or cesarean delivery, either directly or through modifications of breast microbiome, may increase risk of immune-mediated and inflammatory conditions such as atopic disorders, inflammatory bowel disease and obesity later in life.[27-30] While the potential for important benefits in both the mother and infant likely outweighs the likelihood and effects of antibiotic resistance and microbiome changes, these should be monitored. In addition to monitoring resistance patterns in isolates from clinical infections, antimicrobial and microbiome surveillance of a subset of enrolled participants are included in an ancillary protocol.

For peer review only - http://bmjopen.bmj.com/site/about/guidelines.xhtml

3 ANALYTICAL PLAN

Baseline demographic characteristics and key clinical measures will be compared between the women in the two treatment arms using contingency table approaches for categorical variables and analysis of variance models' continuous variables.

For summaries of study data, categorical measures will be summarized in tables listing the frequency and the percentage of participants; continuous data will be summarized by presenting mean, standard deviation, median and range; and ordinal data will be summarized by only presenting median and range. P-values presented will be based on two-sided tests unless otherwise specified and generally adjusted for randomization factor of site. For most analyses, the interaction between treatment and site will be assessed and if significant, results will also be presented by site. For continuous outcomes, distributional properties will be evaluated and if required, transformations or non-parametric tests will be employed. Additional details for potential covariate adjustments in secondary analyses or handling violations of analytic method assumptions will be detailed in the statistical analysis plan.

Three key populations are of interest for study analyses:

1. The Intention to Treat (ITT) population will include all women randomized and their infants. Analyses of this cohort will be conducted based on randomized treatment.
2. The High-Risk for Infection (HR) sub-group will include all women in the ITT and their infants meeting criteria for being high risk (i.e., prolonged labor [≥18 hours] and/or rupture of membranes [≥8 hours]) at the time of randomization. Analyses of this cohort will be conducted based on randomized treatment.
3. The As Treated population will include all randomized participants that receive any study drug during the study and their infants. Analyses of this cohort will be conducted based on treatment received.

The final determination of analysis population membership will be via a masked data review prior to final study analyses in order to address any potential anomalous cases that may arise in this large of a study population (e.g., randomization/treatment of a woman who is discharged prior to delivery due to false labor or unresponsiveness to induction).

3.1 Statistical Analysis Plan

3.1.1 Primary Analyses

Incidence of maternal death or sepsis and intrapartum/neonatal death or sepsis will be compared between the two treatment arms using generalized linear models. These generalized linear models will be fit with each binary outcome separately as the outcome measure. Estimates of relative risk and associated 95% confidence intervals will be reported. The model will include terms for treatment and site. As randomization occurs at the pregnancy level and approximately 1-2% of pregnancies are anticipated to be multiple gestations, models for neonatal outcomes will account for correlation among multiples assuming an exchangeable covariance structure. For the two primary outcomes, these analyses will be conducted using the ITT population and the p-values associated with the treatment term will be used to formally test each of the two primary hypotheses at the alpha = 0.05 level.

As secondary analyses of the primary outcomes, assuming an overall treatment effect is observed, the models will be run including region (Africa, Latin America, or Asia) and a treatment by region interaction

For peer review only

For peer review only - http://bmjopen.bmj.com/site/about/guidelines.xhtml

term. If the interaction term has a p<0.1, then effects will be reported by region with treatment effect within region tested at the 0.025 level.

Additional exploratory models will also be run including individually: 1) a treatment by site interaction term, 2) any other antibiotic use during labor (yes or no) and its interaction with treatment, and 3) mode of delivery (cesarean or vaginal) and its interaction with treatment. If the interaction term for any of these models has a p<0.1, then effects will also be reported by the relevant subgroups. These exploratory secondary models will also include any demographic or clinical variables found to differ significantly between the treatment arms in the preliminary analyses described above.

From each final model, estimates of relative risk associated with treatment will be obtained including unadjusted estimates of risk from the primary model as well as estimates of risk adjusted for potential confounders from the secondary analyses.

3.1.2 Secondary Analyses – Women at High Risk for Infection Cohort

The major secondary aim is assessing the two primary outcomes (i.e., incidence of maternal death or sepsis and incidence of intrapartum/neonatal death or sepsis) in the women at high risk for infection cohort. These analyses will also assess if the treatment effect differs between the HR cohort vs. non-high-risk women where non-high-risk women comprise all women and their infants in the ITT population that delivered prior to meeting criteria for high-risk (i.e. they delivered after < 18 hours of labor and < 8 hours post-rupture of membranes relative to time of randomization). Specifically, the model for the primary analysis of both the maternal and neonatal primary outcomes will be run including a treatment by risk status interaction term and excluding any data from individuals that meet high-risk criteria after randomization.

The exclusion of data from individuals meeting high risk criteria between randomization and delivery is intended to provide the most distinct groups regarding the HR risk cohort to non-high-risk women comparison. As this exclusion is based on a post-randomization event, sensitivity analyses will be conducted to examine any treatment group differences in meeting high risk criteria post randomization and if there is a difference, to determine the potential magnitude of impact this difference has on treatment effect. An exploratory analysis will also be completed that assesses if there is a difference in treatment effect between women randomized prior to high-risk classification vs. those randomized after high-risk classification. Finally, an exploratory analysis will be conducted that assesses the definition of high-risk to determine if there is a different cut-off that identifies a group of individuals with a greater treatment effect both in relation to randomization and delivery.

3.1.3 Secondary Analyses – Other Secondary Outcomes

Other maternal and neonatal binary outcomes including: the individual components of the primary outcomes, neonatal deaths due to sepsis, maternal infections (clinical chorioamnionitis, endometritis, wound infections (perineal or subsequent cesarean), pyelonephritis and pneumonia), use of subsequent maternal antibiotic therapy, pyloric stenosis and occurrences of maternal or neonatal readmission or admission into special care unit will be analyzed using the approaches detailed in **Section 3.1.1** for the ITT population and approaches detailed in **Section 3.1.2** for the HR cohort. A similar process with generalized linear models employing an appropriate link function will be used to analyze the outcomes of maternal and neonatal initial hospital length of stay.

For peer review only - http://bmjopen.bmj.com/site/about/guidelines.xhtml

Binary safety outcomes, e.g., nausea, vomiting, and diarrhea, will also be analyzed using the approaches detailed in **Section 3.1.1**. These analyses will be conducted using the As Treated population.

3.2 Sample Size

3.2.1 Sample Size for Primary Outcome

Sample size estimates were generated to evaluate the potential benefits of peripartum prophylactic azithromycin to reduce the risk of adverse maternal and neonatal outcomes in two population cohorts of women in low and low-middle income settings. The first population of interest comprises all women delivering in facilities (overall).

Power calculations for the study in the overall study population were generated for two primary outcome measures, one being the risk of maternal death or sepsis among women in the target population and the other being intrapartum /neonatal death or sepsis in infants delivered by women in the target population. For each of these outcome measures estimates of the required sample size needed to detect a risk reduction of 20%, 25%, and 30% were generated for power of 0.8, 0.85, and 0.9. The risk of sepsis or maternal death was assumed to be 3%. That number is slightly higher than the current risk in the GN population, which is slightly less than 2%. However, we anticipate that with active surveillance rather than passive reporting based on the new WHO definition of maternal sepsis (designed to catch more cases of sepsis), the risk will be at least 3%. For the neonatal outcome, the underlying risk of the combined outcome of intrapartum stillbirth, neonatal death, or sepsis was assumed to be between approximately 8% and 14%. This estimate was based on recent data from the GN indicating that the risk of intrapartum stillbirth is approximately 1.8% and the risk of neonatal death during the first 28 days after delivery is 2.3%; we assumed that the risk of sepsis not resulting in death is approximately 4% to 10%. The resulting required evaluable sample sizes are shown in **Table 1** below.

Table 1. Sample Sizes for the Overall Population, Alpha=0.05

		Evaluable Sample Size per Arm		
Baseline Risk	Risk Reduction	Power=0.80	Power=0.85	Power=0.90
3%	20%	11455	13103	15334
3%	25%	7133	8159	9548
3%	30%	4815	5508	6446
8%	20%	4096	4686	5483
8%	25%	2554	2921	3419
8%	30%	1727	1975	2311
14%	20%	2204	2521	2950
14%	25%	1377	1575	1842
14%	30%	932	1066	1247

The sample sizes shown above assume that the hypothesis test of interest is for the overall population and that the Type 1 error is controlled at the neonatal and maternal hypothesis level via testing each at an alpha = 0.05 level. Interest has been expressed for the overall study for being able to test the neonatal risk separately in south Asia and sub-Saharan Africa. Controlling the Type I error rate at the 0.025 level for each of those two areas within this sub-analysis of neonatal risk will be conducted to avoid multiple comparison concerns. The resulting sample sizes for that comparison are shown in Table 2 below. Note that this sample size would be the size required separately for the African and Asian sites.

For peer review only - http://bmjopen.bmj.com/site/about/guidelines.xhtml

It is planned that each site will enroll approximately equal number of participants. As such, approximately 37.5% of randomized mothers will be from sub-Saharan Africa and 50% will be from Asia. This planned enrollment distribution is approximately equivalent to the rates of in-facility deliveries observed in the GN registry database. Specifically, approximately a third of the facility deliveries are conducted in sub-Saharan Africa and between 45% and 50% of the facility deliveries are conducted in Asia. These estimates are also consistent with the enrollment rates for the ASPIRIN study that is currently being conducted at these sites. Therefore, to get the total study sample size required, the numbers in the table would need to be multiplied by 3 to achieve reasonable power for the African sites.

Table 2. Sample Sizes Within Region, Alpha=0.025

		Evaluable Sample Size per Arm		
Baseline risk	Risk Reduction	Power=0.80	Power=0.85	Power=0.90
8%	20%	4961	5607	6477
8%	25%	3093	3496	4038
8%	30%	2091	2363	2729
14%	20%	2669	3017	3485
14%	25%	1677	1884	2176
14%	30%	1129	1276	1474

Given the above information, we propose a sample size of 34,000 participants for the overall study. For the primary neonatal outcome of interest of intrapartum/neonatal sepsis or death, assuming that the loss to follow-up will be in the 2% to 3% range (consistent with the current ASPIRIN trial and the Global Network Maternal and Newborn Health registry), this sample size will be sufficient to provide 90% power to detect a 25% reduction in neonatal mortality and sepsis in the sub-Saharan African region and will provide 90% power to detect a 20% reduction in Asia assuming the baseline risk is at least 8%. For the primary maternal outcome of maternal death or sepsis, the sample size will provide 90% power to detect a 20% reduction from 3% in the population aggregated across all study sites.

3.2.2 Sample Size for High-Risk Women

The second population of interest comprises the cohort of high-risk population of women delivering in facilities with high risk being defined as term and preterm pregnant women who experience prolonged labor or prolonged membrane rupture. As a major secondary aim, sample size estimates for the high-risk cohort were also obtained to ensure adequate power for this analysis.

The primary objective of the high-risk component of the study is to test the effectiveness of a single oral dose of intrapartum azithromycin prophylaxis compared to placebo (all receive usual care) in reducing the risk of maternal sepsis or death in high-risk laboring women. To estimate the sample size required for this component of the study, we assumed conservatively that the underlying risk of the combined outcome in the target population is 6%. Comparable to the other study estimates of the required sample size needed to detect a risk reduction of 20%, 25%, and 30% were generated for power of 0.8, 0.85, and 0.9. Because the interest for this study is in testing the hypothesis overall rather than by region, the estimates were generated using an alpha of 0.05. The results of these calculations are shown in **Table 3** below.

For peer review only - http://bmjopen.bmj.com/site/about/guidelines.xhtml

Table 3. Sample Sizes for the High-Risk Cohort, Alpha=0.05

Baseline risk	Risk Reduction	Evaluable Sample Size per Arm		
		Power=0.80	Power=0.85	Power=0.90
6%	20%	5568	6369	7453
6%	25%	3470	3969	4644
6%	30%	2344	2681	3138

Because a risk reduction of at least 30% is expected in this population, we propose a sample size for this HR cohort of 5,500 women. That sample size will be sufficient to detect a 30% risk reduction with a power of 0.85 with an assumed 2% to 3% loss to follow-up. Assuming that 20% of the women are at high risk, the overall study sample size of 34,000 should allow for sufficient enrollment into the HR cohort.

If the proportion of "high-risk" women is smaller than anticipated, the target enrollment may be modified. A smaller sample size should still provide sufficient power for plausible potential underlying scenarios. For example, a sample size of approximately 4,000 high risk women will be sufficient to detect a 33% risk reduction with a power of 0.82 assuming a baseline risk of at least 6% as well as a 35% risk reduction with a power of 0.80 assuming a baseline risk of at least 5%.

3.3 Available Population

There are no competing protocols ongoing in the GN. Assuming conservatively that 50% of women in labor (30,000 facility births per year) meet eligibility criteria for entry and are enrolled in the trial, approximately 15,000 women/infant dyad will be enrolled per year.

To reach the enrollment target of 34,000, each study site will aim to recruit an equal number of study participants (n=4,250 per site); however, recruitment will be monitored and if a site does not meet targets, adjustments may be made. No site will be permitted to recruit more than 20% of the overall study sample site.

Based on historic GN data, we anticipate that the target of 5,500 high-risk women will be enrolled during the trial. However, through ongoing monitoring, the DCC will assess the number of women enrolled in the "high-risk" group and may modify target enrollment as needed to ensure at least 5,500 high-risk women are enrolled.

3.4 Projected Recruitment Time

The projected study timeline is 36 months or less. This includes the following:

- 0-6 months: Finalize protocol, forms. Obtain approvals, train staff, and obtain/ship study drug/placebo to sites.
- 7-31 months: Enroll participants (exact period of enrollment may vary by site)
- 32-36 months: Complete follow-up; data cleaning and primary analyses.

3.5 Study Monitoring Plan

3.5.1 Reporting Serious Adverse Events

Serious Adverse events (SAEs) will be monitored continuously for any event that meets the following criteria:

- Results in neonatal/fetal or maternal death;
- Is life-threatening;

For peer review only - http://bmjopen.bmj.com/site/about/guidelines.xhtml

- Requires hospitalization or prolongs existing hospitalization;
- Results in persistent or significant disability or incapacity;
- Suspicion of pyloric stenosis within 42 days postpartum (prolonged vomiting leading to death or surgery)
- Any other serious or unexpected adverse event that the study investigator(s) feels should be reported.

3.5.2 Method and Timing for Reporting Serious Adverse Events

The Senior Foreign Investigator (SFI) must report the following SAEs by emailing or faxing a copy of the appropriate study form to RTI as follows:

Within 48 hours of SFI's notification of the event:

- All deaths (maternal, intrapartum stillbirths, neonatal)
- All SAEs with a definite or suspected/probable relationship to the intervention

Within 7 days of SFI's notification of the event:

- All life-threatening events;
- All SAEs considered to have a probable or possible relationship to the intervention;
- All emailed or faxed forms should also be entered into the DMS and transmitted within 7 days as a back-up to ensure no SAE is missed.

Additional reporting procedures include:

- RTI will forward all SAEs to the US-based Principal Investigator (PI) and NIH for further assessment of relationship to study intervention. The PI and SFI will be responsible for reporting to their respective IRB and other regulatory authorities per their institutional policy.
- RTI will be responsible for reporting SAEs to the DMC bi-annually at a minimum. The frequency of reporting to the DMC may be increased if the reported events or interim data reviews by the DMC indicate that more frequent safety monitoring is needed.
- Any SAE considered unrelated to the intervention is not required to be reported in an expedited manner. These events should be entered into the data management system and transmitted per routine procedures.

3.5.3 Data Monitoring Plan and Stopping Rules

All the Global Network sites will report data to the Global Network Data Coordinating Center, located at RTI International. The data will be used to evaluate protocol adherence and site performance (e.g., recruitment, loss to follow-up, data quality). The DCC will provide standardized progress reports to NICHD and the site investigators on a monthly basis to monitor outcome variables and adverse events.

Oversight of the trial will be handled by two principal groups with different focuses:

1. **Protocol-focused Steering Committee (SC):** The SC is comprised of the Central Study Team from the University of Alabama at Birmingham and the University of Zambia, NICHD, the DCC, and investigators from each of the participating sites (see Azithromycin Investigators on page 8). The Central Study Team, with assistance from NICHD and the DCC, will have primary responsibility for overall study design, development of study materials and procedures, and oversight of study implementation. They will meet via conference call bi-weekly to monitor study progress and ensure

For peer review only - http://bmjopen.bmj.com/site/about/guidelines.xhtml

proper implementation of the trial. The Site Investigators will be responsible for providing guidance on study design, developing site-specific implementation plans, ensuring study staff are properly trained, and providing oversight of the study at the site level. The SC will convene via conference call at least once per quarter and will meet in person twice a year to discuss study design and implementation issues. Members of the Central Study team, NICHD, and RTI will also conduct site visits, as the budget allows, to bolster enthusiasm, provide hands-on training and education to the participating staff, and address site-specific issues, if any.

2. **Data Monitoring Committee (DMC)**: The DMC, a standing group that monitors all NICHD-funded Global Network studies, will be responsible for ensuring safe and ethical treatment of study participants through monitoring of the study. The membership will include, at a minimum, a statistician, obstetrician, pediatrician, and an expert in international health. The DMC designated by NICHD will review the data collected at approximate 6-month intervals throughout the course of the study. The DMC reports, which are prepared by the Data coordinating center, will include information on study enrollment rates and participant progress through the study, participant compliance with protocol-specified treatment regimens, protocol violations, adverse events, and efficacy outcomes. The focus of the DMC review will be on monitoring participant safety and study progress/futility. Data on treatment effectiveness will also be presented to frame the DMC discussions on safety and futility. Additionally, one formal interim analyses of efficacy is planned as detailed in **Section 3.5.4**. The DMC will be charged with monitoring adverse events and side effects from azithromycin. All known associated side effects and specific obstetric, fetal, or neonatal concerns will be considered reportable to the DMC. The study will be reviewed by the DMC bi-annually at a minimum but may be reviewed more frequently if concerns are raised about participant safety or about adequate process of the study.

3.5.4 Interim/Adverse Event Monitoring Plan

The DMC designated by NICHD will complete safety reviews of the data bi-annually at minimum during the intervention phase or as often as they decide. Safety reports will be reviewed internally by the DCC quarterly and the DMC chair will be notified if any potential safety signals are identified to allow for more frequent DMC monitoring if needed. Adverse events will the reported and submitted to the DCC (and IRBs) who will report these cumulative masked data to the DMC in the safety reviews. The DMC recommendations about study continuation will be distributed to the IRBs. The DMC will forward their monitoring activities to the project officer representing the NICHD.

Additionally, one formal interim analysis of efficacy and futility will be conducted during the study. Interim analyses will be conducted in both all-comers and high-risk cohort assessing both the primary maternal and neonatal outcomes use the primary analytic approach detailed in **Section 3.1.1 and 3.1.2**. As the maternal and neonatal hypotheses are both of equal importance as well as the treatment effect within the two cohorts, the DMC will not be able to recommend early termination for efficacy unless significance is observed for both outcomes in all comers and the direction and magnitude of effect in the high-risk cohort is consistent with the effect observed in all comers. Cut-off p-values for testing for efficacy at this interim analysis will be determined based on a correction for multiple comparisons to ensure an overall alpha of 0.05. The futility assessment will be based on an analysis of conditional power. The details of the timing of the interim analysis as well as the approaches for correcting for multiplicity and analyzing conditional power will be determined in collaboration with the DMC prior to initiation of study enrollment. Safety and efficacy of the azithromycin will be reviewed and compared

For peer review only - http://bmjopen.bmj.com/site/about/guidelines.xhtml

with data from the placebo group according to a data center plan. The primary outcome rate will be calculated, and the data center will develop guidelines for interim analysis decision-making (e.g. the O'Brien-Fleming boundary table).

3.5.5 Risks/Benefits

Based on the preliminary data, there are few risks to this intervention especially given the single oral dose. Severe risks include anaphylaxis and allergic reactions (rash) and liver failure which are rare with azithromycin based on the product insert and our long history of use of prophylactic azithromycin at UAB since 2000. In 2013, the FDA issued an advisory regarding concerns about potential for rare life-threatening arrhythmias with azithromycin use particularly among those with preexisting cardiovascular risk. To minimize the risk of life-threatening side effects, those with arrythmia, known history of cardiomyopathy, or a known allergy to azithromycin will be excluded. With single 1-2 gram doses, gastrointestinal symptoms (nausea 5-18%, diarrhea/loose stools 7-14%, abdominal pain 5-7%, vomiting 2-7%, and dyspepsia 1%) as well as vaginitis 2% and dizziness (1%) were the most commonly reported side effects. These side effects will be monitored.

3.6 Quality Control

3.6.1 Training

All study personnel must participate in training on the proper implementation of study procedures and the ethics of conducting research with human subjects before beginning any research activity. The SFI and project coordinator will ensure that all study personnel receive the appropriate training and obtain the required certification. RTI will be responsible for developing a certification test. The SFI and project manager will be responsible for overseeing the certification process.

3.6.2 Study Monitoring

Major monitoring responsibilities of the PI/SFI, assisted by the country coordinator, are (1) confirming proper IRB approval; (2) monitoring delivery of the study intervention; (3) assessing and evaluating quality of study implementation; (4) ensuring compliance with the intervention, including proper randomization; (5) evaluating accuracy, precision, and completeness of data collected, entered, and transmitted, along with the DCC; (6) ensuring that all personnel are fulfilling their obligations; (7) maintaining staff morale and enthusiasm; (8) maintaining communication and handling ad hoc problems; (9) ensuring inter-site consistency; and (10) proposing improvements to monitoring activities.

NICHD and the DCC staff will conduct site visits as needed. These visits will include review of individual participant records, including supporting data, to ensure protection of study participants, compliance with the protocol, and accuracy and completeness of records. The SFI/PI will make study documents (e.g., logbooks, data forms, staff training certificates) and pertinent hospital/clinic records readily available for inspection by the local IRB, site monitors, and the NICHD for confirmation of the study data.

3.6.3 Drug Quality Assurance and Monitoring

The study drug manufacturer will have a Good Manufacturing Practices designation vetted by the FDA and a certificate of authenticity will be provided. Each site will adapt best practice guidelines for drug shipment and storage to the needs and infrastructure of their local environment. Study staff will be trained in on the drug shipment and storage plan to ensure that best practices are maintained at all times. Additionally, participants will receive detailed instruction on proper storage of the study drug at

For peer review only - http://bmjopen.bmj.com/site/about/guidelines.xhtml

home. Drug stability information will be maintained throughout the study. For quality assurance, a sample of pills from each site will be randomly selected and tested for bioavailability at multiple time points during the study period. A sample from each batch will be tested.

3.6.4 Plan for Sustaining Intervention

We will plan to present abstracts to reputable international obstetric meetings (e.g. SMFM, ACOG or FIGO) and manuscripts within 3-6 months of completion of the primary data collection to high impact journals such as the New England Journal of Medicine, JAMA, or the Lancet. If the results are positive, we will facilitate the change in practice at participating sites and also approach the WHO to instigate guideline updates to reflect the study findings.

4 DATA MANAGEMENT PROCEDURES

Data will be collected both prospectively and from existing medical records, using hard copy forms or Android Tablets. Regardless of data capture methodology, all data will be kept confidential. Each participant will be assigned a unique study ID which will be used to identify the participant. Only the screening log will contain the name (which is not transmitted). If hard copy forms are used, they will be retained in a secure location for possible editing or queries at the central data entry site. Data will be entered into computers using the Data Management System (DMS) developed by RTI and the assigned study number. The DMS will also allow site staff to produce project reports and backup the study database. Electronic data will be transferred from each data management computer to a single Research Unit Data Center in each country, creating a complete data repository. At least once a week, data will be transmitted from the Research Unit Data Center to the DCC at RTI, where the central database will be located. The DCC will conduct training on data collection procedures and the DMS system, as needed.

Precision and accuracy of actual data collected will be checked by chart review (random 5%) and internal procedures using the computer program. Monthly audits and incomplete data reports will be performed by a review team consisting of at least the SFI and country coordinator. Data editing and error resolution will be performed monthly. In addition, a sample of participants will be visited to confirm participation, with procedures determined per site. These activities will be shared between the site and the DCC. The timing of data collection is found in the schedule of study procedures (**Appendix 3).**

4.1 Data Forms

The following forms will be used for this study:

Form Name	Purpose	Key Data Elements	Data Source
**Contact and Scheduling Form**	To facilitate participant follow-up by documenting contact information and projected study visit schedule	Contact information, date of enrollment, date of follow-up visits	Participant interview, study calendar
**Screening and Enrollment Form**	To determine eligibility and record consent status	Screening date, review and confirmation of inclusion/exclusion criteria, consent status/date.	Participant interview/report, clinical assessment, provider report, medical records
**Randomization Form**	To confirm eligibility and track randomization	Eligibility confirmation, randomization date/time, drug administration date/time, any problems with drug administration (e.g., vomiting, dropped med, etc).	Study records, participant interview and observation

For peer review only - http://bmjopen.bmj.com/site/about/guidelines.xhtml

Form Name	Purpose	Key Data Elements	Data Source
**Maternal Baseline Data Form**	To collect additional maternal information	• Demographic and baseline clinical data: admission date/time, EDD, GA, age, height, weight, pregnancy and medical history, etc.<br>• Events during labor and delivery: timing of onset of labor, prolonged labor, premature rupture of membranes, date/time/type of membrane rupture, date/time/type of delivery, indication for induction (if applicable), vital signs during labor (temp, HR, RR, BP), complications, etc.<br>• Events after delivery: complications, vital signs, etc.<br>• Infection assessment pre- and post-delivery<br>• Antibiotic treatment pre- and post-delivery<br>• Discharge information	Participant interview/report, provider report, medical records
**Neonatal Baseline Data Form**	To collect information about the infant directly after delivery.	• Sex<br>• Birthweight<br>• Delivery outcome (live or stillbirth)<br>• Complications<br>• Infection assessment<br>• Discharge information: date/time, status (discharged, transferred, died)	Participant interview/report, provider report, medical records
**Maternal and Neonatal Follow-up Form**	To collect maternal and infant health status during follow-up visits at 3 days, 7 days and 42 days after delivery.	• Timing follow-up<br>• Maternal and infant status since discharge: general status, symptoms, indication of infection, clinic visits, hospitalizations, antibiotic use<br>• Temperature	In-person participant interview/report and clinical exam
**Supplemental Infection Contact Form**	To document signs of maternal or neonatal infection and provide referrals if indicated.	• Maternal and neonatal infection danger signs.<br>• Referral information.	Participant interview/report by phone
**Maternal and Neonatal Unscheduled Medical Visit**	To collect information about clinical events reported during follow-up visits	Reason for medical visit, details about medical visit	Participant interview/report, provider report, clinical exam, medical records
**Specimen Collection and Result Form**	To track specimen collection when there is indication of maternal or infant infection	Date/time of specimen collection; reason for collection; location of wound/infection; tracking information for shipping/storing specimen; results	Study documentation if collected and/or tested by study; medical records if collected and/or tested by health facility.
**Serious Adverse Events**	To record fatal, life-threatening, or any other serious, unexpected adverse event	Date/time of event, date/time of resolution, nature of adverse event, management of adverse event, attribution to study (yes/no).	Participant interview/report, provider report, medical records
**Final Study Status**	To document final study status	Final study status (e.g., completion, withdrawal, lost-to-follow-up), Date of final study status. If withdrawal, provide reason.	Participant report, medical records
**Protocol Deviation**	To record protocol deviations and corrective actions	Date and nature of deviation/violation, corrective action.	Participant report, study records, medical records
**Outcome Adjudication**	To validate cases of infection using standard study definitions	Primary and secondary outcomes	Study documentation, provider reports/interviews, medical records

For peer review only - http://bmjopen.bmj.com/site/about/guidelines.xhtml

5 References

1. World Health Organization. (2015). WHO recommendations for the prevention and treatment of maternal peripartum infections. Retrieved August 22, 2018, from http://www.who.int/reproductivehealth/publications/maternal_perinatal_health/peripartum-infections-guidelines
2. Liu, L., Oza, S., Hogan, D., Perin, J., Rudan, I., Lawn, J. E., . . . Black, R. E. (2015). Global, regional, and national causes of child mortality in 2000-13, with projections to inform post-2015 priorities: an updated systematic review. *The Lancet, 385*(9966), 430-440.
3. Tshefu, A., Lokangaka, A., Ngaima, S., Engmann, C., Esamai, F., Gisore, P., . . . African Neonatal Sepsis Trial (AFRINEST) group. (2015). Simplified antibiotic regimens compared with injectable procaine benzylpenicillin plus gentamicin for treatment of neonates and young infants with clinical signs of possible serious bacterial infection when referral is not possible: a randomised, open-label, equivalence trial. *Lancet, 385*(9979), 1767-1776.
4. Baqui, A. H., Saha, S. K., Ahmed, A. S., Shahidullah, M., Quasem, I., Roth, D. E., . . . Projahnmo Study Group in Bangladesh. (2015). Safety and efficacy of alternative antibiotic regimens compared with 7 day injectable procaine benzylpenicillin and gentamicin for outpatient treatment of neonates and young infants with clinical signs of severe infection when referral is not possible: a randomised, open-label, equivalence trial. *Lancet Global Health, 3*(5), e279-287.
5. Mir, F., Nisar, I., Tikmani, S. S., Baloch, B., Shakoor, S., Jehan, F., . . . Zaidi, A. K. (2017). Simplified antibiotic regimens for treatment of clinical severe infection in the outpatient setting when referral is not possible for young infants in Pakistan (Simplified Antibiotic Therapy Trial [SATT]): a randomised, open-label, equivalence trial. *Lancet Global Health, 5*(2), e177-e185.
6. Zaidi, A. K., Tikmani, S. S., Sultana, S., Baloch, B., Kazi, M., Rehman, H., . . . Cousens, S. (2013). Simplified antibiotic regimens for the management of clinically diagnosed severe infections in newborns and young infants in first-level facilities in Karachi, Pakistan: study design for an outpatient randomized controlled equivalence trial. *Pediatric Infectious Disease Journal, 32 Suppl 1*(Suppl 1), S19-25. https://doi.org/10.1097/INF.0b013e31829ff7aa
7. Gibbs, R. S. (1980). Clinical risk factors for puerperal infection. *Obstetrics and Gynecology, 55*(5 Suppl), 178S-184S.
8. World Health Organization. (2015). WHO Statement on Caesarean Section Rates. Retrieved August 22, 2018, from http://www.who.int/reproductivehealth/publications/maternal_perinatal_health/cs-statement/en/
9. Mackeen, A. D., Packard, R. E., Ota, E., Berghella, V., & Baxter, J. K. (2014). Timing of intravenous prophylactic antibiotics for preventing postpartum infectious morbidity in women undergoing cesarean delivery. *Cochrane Database of Systematic Reviews, 12*(12), CD009516. https://doi.org/10.1002/14651858.CD009516.pub2
10. Smaill, F. M., & Grivell, R. M. (2014). Antibiotic prophylaxis versus no prophylaxis for preventing infection after cesarean section. *Cochrane Database of Systematic Reviews, 10*, CD007482. https://doi.org/10.1002/14651858.CD007482.pub3
11. Gyte, G. M. L., Dou, L. X., & Vazquez, J. C. (2014). Different classes of antibiotics given to women routinely for preventing infection at caesarean section. *Cochrane Database of Systematic Reviews, 11*(11). https://doi.org/10.1002/14651858.CD008726.pub2
12. Tita, A. T. N., Rouse, D. J., Blackwell, S., Saade, G. R., Spong, C. Y., & Andrews, W. W. (2009). Evolving concepts in antibiotic prophylaxis for cesarean delivery: a systematic review. *Obstetrics and Gynecology, 133*(3), 675-682.
13. Tita ATN, S. J., Boggess K, et al. . (2016). Adjunctive azithromycin prophylaxis for cesarean delivery. *New England Journal of Medicine, 375*, 1231-1241.
14. Oluwalana, C., Camara, B., Bottomley, C., Goodier, S., Bojang, A., Kampmann, B., . . . Roca, A. (2017). Azithromycin in labor lowers clinical infections in mothers and newborns: a double-blind trial. *Pediatrics, 139*(2). https://doi.org/10.1542/peds.2016-2281

For peer review only - http://bmjopen.bmj.com/site/about/guidelines.xhtml

15. World Health Organization. (2017). Statement on maternal sepsis. Retrieved Ausust 22, 2018, from http://www.who.int/reproductivehealth/publications/maternal_perinatal_health/maternalsepsis-statement/en/
16. Reinhart K, D. R., Kissoon N, Machado FR, Schachter RD, Finfer S. . (2017). Recognizing sepsis as a global health priority - a WHO resolution. *New England Journal of Medicine, 377*(5), 414-417.
17. van Dillen, J., Zwart, J., Schutte, J., & van Roosmalen, J. (2010). Maternal sepsis: epidemiology, etiology and outcome. *Current Opinion in Infectious Diseases, 23*(3), 249-254. https://doi.org/10.1097/QCO.0b013e328339257c
18. Roca A, O. C., Bojang A, Camara B, Kampmann B, Bailey R, Demba A, Bottomley C, D'Alessandro U. (2016). Oral azithromycin given during labour decreases bacterial carriage in the mothers and their offspring: a double-blind randomized trial. *Clin Microbiol Infect. , 22*(6), e1-9.
19. Subramaniam A, Ye Y, Mbah R, Mbunwe DM, Pekwarake S, Bunwi EY, Fondzeyuf A, Ngong MG, Dionne-Odom J, Harper LM, Jauk VC, Carlo WA, Halle-Ekane G, Szychowski JM, Tih P, Tita AT. Single Dose of Oral Azithromycin With or Without Amoxicillin to Prevent Peripartum Infection in Laboring, High-Risk Women in Cameroon: A Randomized Controlled Trial. Obstet Gynecol. 2021;138(5):703-713. PMID: 34619734.
20. Harper, L. M., Kilgore, M., Szychowski, J. M., Andrews, W. W., & Tita, A. T. N. (2017). Economic evaluation of adjunctive azithromycin prophylaxis for cesarean delivery. *Obstetrics and Gynecology, 130*(2), 328-334. https://doi.org/10.1097/AOG.0000000000002129
21. Andrews, W. W., Hauth, J. C., Cliver, S. P., Savage, K., & Goldenberg, R. L. (2003). Randomized clinical trial of extended spectrum antibiotic prophylaxis with coverage for Ureaplasma urealyticum to reduce post-cesarean delivery endometritis. *Obstetrics and Gynecology, 101*(6), 1183-1189.
22. Tita AT, H. J., Grimes A, Owen J, Stamm AM, Andrews WW. . (2008). Decreasing incidence of post-cesarean endometritis with extended-spectrum antibiotic prophylaxis. *Obstetrics and Gynecology, 111*(1), 51-56.
23. Tita, A. T., Owen, J., Stamm, A., Hauth, J. C., & Andrews, W. W. (2008). Impact of extended-spectrum antibiotic prophylaxis on incidence of post-cesarean surgical wound infection. *American Journal of Obstetrics and Gynecology, 199*(3), 303.
24. Watts, D. H., Eschenbach, D. A., & Kenny, G. E. (1989). Early postpartum endometritis: the role of bacteria, genital mycoplasmas, and Chlamydia trachomatis. *Obstetrics and Gynecology, 73*(1), 52-60.
25. Hoyme, U. B., Kiviat, N., & Eschenbach, D. A. (1986). Microbiology and treatment of late postpartum endometritis. *Obstetrics and Gynecology, 68*(2), 226-232.
26. Emmons, S. L., Krohn, M., Jackson, M., & Eschenbach, D. A. (1988). Development of wound infections among women undergoing cesarean-section. *Obstetrics and Gynecology, 72*(4), 559-564.
27. Roberts, S., Maccato, M., Faro, S., & Pinell, P. (1993). The microbiology of post-cesarean wound morbidity. *Obstetrics and Gynecology, 81*(3), 383-386.
28. Rosene, K., Eschenbach, D. A., Tompkins, L. S., Kenny, G. E., & Watkins, H. (1986). Polymicrobial early postpartum endometritis with facultative and anaerobic bacteria, genital mycoplasmas, and Chlamydia trachomatis: treatment with piperacillin or cefoxitin. *Journal of Infectious Diseases, 153*(6), 1028-1037.
29. Andrews, W. W., Shah, S. R., Goldenberg, R. L., Cliver, S. P., Hauth, J. C., & Cassell, G. H. (1995). Association of post-cesarean delivery endometritis with colonization of the chorioamnion by Ureaplasma urealyticum. *Obstetrics and Gynecology, 85*(4), 509-514. https://doi.org/10.1016/0029-7844(94)00436-H
30. Keski-Nisula, L., Kirkinen, P., Katila, M. L., Ollikainen, M., Suonio, S., & Saarikoski, S. (1997). Amniotic fluid U. urealyticum colonization: significance for maternal peripartal infections at term. *American Journal of Perinatology, 14*(3), 151-156.
31. Yoon, B. H., Romero, R., Park, J. S., Chang, J. W., Kim, Y. A., Kim, J. C., & Kim, K. S. (1998). Microbial invasion of the amniotic cavity with Ureaplasma urealyticum is associated with a robust host response in fetal, amniotic, and maternal compartments. *American Journal of Obstetrics and Gynecology, 179*(5), 1254-1260. https://doi.org/Doi 10.1016/S0002-9378(98)70142-5
32. Ledger, W. J. (2006). Prophylactic antibiotics in obstetrics-gynecology: a current asset, a future liability? *Expert Review of Anti-Infective Therapy, 4*(6), 957-964. https://doi.org/10.1586/14787210.4.6.957
33. World Health Organization. (2015). Guideline: Managing Possible Serious Bacterial Infection in Young Infants When Referral is not Feasible. Retrieved August 22, 2018, from

For peer review only - http://bmjopen.bmj.com/site/about/guidelines.xhtml

http://apps.who.int/iris/bitstream/handle/10665/181426/9789241509268_eng.pdf;jsessionid=F74B437689D1CB960E2544DDF53FE5A6?sequence=1

34. Sutton, A. L., Acosta, E. P., Larson, K. B., Kerstner-Wood, C. D., Tita, A. T., & Biggio, J. R. (2015). Perinatal pharmacokinetics of azithromycin for cesarean prophylaxis. *American Journal of Obstetrics and Gynecology, 212*(6), 812.
35. Eberly, M. E., Eide, M. B., Thompson, J. L., & Nylund, C. M. (2015). Azithromycin in Early Infancy and Pyloric Stenosis. *Pediatrics, 135*(3), 483. https://doi.org/10.1542/peds.2014-2026
36. Ray, W. A., Murray, K. T., Hall, K., Arbogast, P. G., & Stein, C. M. (2012). Azithromycin and the risk of cardiovascular death. *New England Journal of Medicine, 366*(20), 1881-1890.
37. Svanström, H., Pasternak, B., & Hviid, A. (2013). Use of azithromycin and death from cardiovascular causes. *New England Journal of Medicine, 368*(18), 1704-1701. https://doi.org/10.1056/NEJMoa1300799
38. Hoffman, M. K., Goudar, S. S., Kodkany, B. S., Goco, N., Koso-Thomas, M., Miodovnik, M., . . . Derman, R. J. (2017). A description of the methods of the aspirin supplementation for pregnancy indicated risk reduction in nulliparas (ASPIRIN) study. *BMC Pregnancy and Childbirth, 17*(1), 135. https://doi.org/10.1186/s12884-017-1312-x
39. Goldenberg, R. L., Saleem, S., Ali, S., Moore, J. L., Lokangako, A., Tshefu, A., . . . McClure, E. M. (2017). Maternal near miss in low-resource areas. *International Journal of Gynaecology and Obstetrics, 138*(3), 347-355. https://doi.org/10.1002/ijgo.12219
40. Bonet, M., Souza, J. P., Abalos, E., Fawole, B., Knight, M., Kouanda, S., . . . Metin Gulmezoglu, A. (2018). The global maternal sepsis study and awareness campaign (GLOSS): study protocol. *Reprod Health, 15*(1), 16. https://doi.org/10.1186/s12978-017-0437-8
41. Albright, C.M., Has, P., Rouse D.J., Hughes B.L. (2018). Internal Validation of the Sepsis in Obstetrics Score to Identify Risk of Morbidity from Sepsis in Pregnancy. Obstetric Anesthesia Digest, 38 (1). https://doi.org/10.1097/01.aoa.0000529967.01647.fb.
42. Bowyer L., Robinson H.L., Barrett H., Crozier T.M., Giles M., Idel I., Lowe S., Lust K., Marnoch C.A., Morton M.R., et al. SOMANZ guidelines for the investigation and management sepsis in pregnancy. Aust. N. Z. J. Obstet. Gynaecol. 2017;57:540–551. doi: 10.1111/ajo.12646.

For peer review only

APPENDIX 1. DESCRIPTION OF PARTICIPATING GLOBAL NETWORK SITES

The Global Network for Women's and Children's Health Research (GN) was created as a private-public partnership between the U.S. National Institutes of Health (NIH) and the Bill and Melinda Gates Foundation in response to the alarming rates of morbidity and mortality in women and children and the lack of research expertise and infrastructure in the developing world. Its mission is to expand scientific knowledge, develop research infrastructures, and improve health outcomes by building research partnerships to conduct research on feasible, cost-effective, sustainable interventions to address the major causes of perinatal morbidity and mortality of women and children in the developing world. It is currently funded only by the Eunice Kennedy Shriver National Institute of Child Health and Human Development (NICHD).

The current configuration of the GN is comprised of eight multidisciplinary research sites in seven developing countries (Bangladesh, Democratic Republic of Congo, Guatemala, India, Kenya, Pakistan, and Zambia), each with an established collaboration between an institution in the U.S. and one or more in the developing country. Each site has a U.S.-based senior principal investigator (PI) and a senior foreign investigator (SFI) based in the developing world, who lead a team of in-country research staff.

In 2005, the GN implemented its first multicounty protocol, the First Breath (FB) study, a community-based cluster trial to determine whether education and training in the American Academy of Pediatrics Neonatal Resuscitation Program (NRP) and the Essential Newborn Care Program (ENC) of the World Health Organization (WHO) reduced neonatal mortality (7 days) more than education and training in ENC alone. More than 3,700 birth attendants from 100 GN communities with > 150,000 deliveries were taught the appropriate procedures and provided with the necessary equipment to resuscitate infants at birth. Communities were randomized to ENC plus NRP or continued ENC.

The GN has continued to build on the capacity developed in the FB trial through the implementation of more than 10 additional multicounty protocols to address priority research needs to improve maternal and child health in low-resource settings. Current projects include:

- **<u>The Maternal Newborn Health Registry</u>** is a prospective, population-based study of pregnancies and their outcomes in low-middle income countries (DRC, Guatemala, India, Pakistan, Bangladesh, Zambia and Kenya). All pregnant women in participating clusters are registered and their outcomes tracked for 6 weeks post-delivery. The primary purpose of this prospective, population-based observational study of approximately 60,000 women per year is to quantify and understand the trends in pregnancy services and outcomes over time in defined, low-resource geographic clusters. The goal is to provide population-based statistics on stillbirths, neonatal and maternal mortality as the basis of health care policy. The data from the registry also provide the mortality and morbidity outcomes for Global Network trials and help investigators plan future studies for the Global Network.
- Preterm birth remains the leading cause of neonatal mortality and long-term disability throughout the developed and developing world. A growing body of evidence suggests that 1st

For peer review only - http://bmjopen.bmj.com/site/about/guidelines.xhtml

trimester administration of low dose aspirin can reduce the rate of PTB substantially. The **ASPIRIN Study** is a prospective, randomized, placebo-controlled, double-masked, multi-center clinical trial to examine whether low dose aspirin initiated between 6 0/7 weeks- 12 6/7 weeks gestation reduces the risk of preterm birth. The study has enrolled 11,920 women across seven sites in Africa, Asia, and Latin America.

- Attention is increasingly directed to the role of maternal nutrition during the 1st trimester for normal growth and development during the first thousand days, from conception to the child's second birthday. The primary hypothesis of the **Women First: Preconception Maternal Nutrition study** is that for women in poor communities, a comprehensive maternal nutrition intervention commencing at least 3 months prior to conception and continuing throughout pregnancy, will be associated with a significantly greater newborn length than for offspring whose mothers start to receive the same intervention at 12 weeks gestation or who do not receive the intervention at all. The results of this trial will make a major contribution to refining evidence-based strategies for maternal nutrition supplementation and evaluating the cost-benefits of extending such strategies beyond pregnancy to virtually all women of child-bearing age, including adolescent girls.

For peer review only

For peer review only - http://bmjopen.bmj.com/site/about/guidelines.xhtml

APPENDIX 2. SAMPLE INFORMED CONSENT

Global Network for Women's & Children's Health Research

Single oral dose of azithromycin 2 gm in laboring women to prevent neonatal infection/death and maternal peripartum infection/death

INVESTIGATORS:
[LIST SITE INVESTIGATORS]

SPONSOR:
The Eunice Kennedy Shriver National Institutes of Child Health and Human Development (NICHD)

You are being asked to participate in a research study for pregnant mothers. This study is funded by the U.S. National Institutes of Health and the Bill and Melinda Gates Foundation. This form provides you with information about the study so that you can decide whether you would like to participate. A member of the research team will describe the study to you and answer all of your questions. Please read the information below and ask questions about anything you don't understand before deciding whether or not to take part. You may also request that the research staff read the form to you.

What is the purpose of the study?
The purpose of this study is to learn whether an antibiotic, called azithromycin, given orally (by mouth) to pregnant women during labor can reduce the risk of infection for the woman and her baby.

Who will be in the study?
A total of 34,000 women will be enrolled in this study from eight sites in sub-Saharan Africa, South Asia, and Latin America. If needed, up to 2000 additional women across all sites who meet high-risk criteria will be enrolled (defined as 8 hours or more of membrane rupture before delivery or 18 hours or more of labor). In [insert site name], no more than [insert max sample size] will be enrolled.

You qualify for this study if you are a pregnant woman of the legal age of consent who is in labor with one or more live fetuses and a pregnancy ≥28 weeks, plan to deliver vaginally in a health facility, have no known infections that require antibiotics, have no known problems taking azithromycin or similar antibiotics (such as amoxicillin), and have not used azithromycin, erythromycin, or similar antibiotic in the past 3 days. We will ask you some questions about your pregnancy and health status to make sure you qualify to participate.

What will happen if I join this study?
Before participating, you will be provided with information about the study procedures and given an opportunity to ask questions. If you qualify and agree to participate, you will be asked to sign this form to indicate your consent.

If you agree to participate, you will be assigned to either the treatment group or the control group. The assignment is made randomly, like flipping a coin or choosing a grain of rice from a bag. The women in the treatment group will take four 500 mg azithromycin pills. For comparison, the women in the control group will take four pills that look identical to azithromycin but do not contain any medication. This will

For peer review only - http://bmjopen.bmj.com/site/about/guidelines.xhtml

allow the researchers to compare how well azithromycin works to prevent infection in mothers and their babies. Neither you nor the study staff will know whether you are assigned to the treatment or control group.

After you have been assigned to a treatment group, you will be given 4 small 500 mg pills to take by mouth. A member of the study team will watch you take the pills. After you take the pills, you will receive care during your labor, delivery, and recovery from the health facility staff, according to the local standard of care. It will take approximately 30 minutes to complete the consent form and take the pills.

While you are at the health facility to deliver your baby, the study team will also collect:

- Information about your health status during labor, at the time of delivery, and after delivery. This includes collecting your temperature and vital signs, such as heartrate and blood pressure;
- Information about your baby's health status during and after delivery. This includes collecting your baby's temperature and vital signs, such as heart rate and blood pressure;
- If you or your baby develop an infection before you are discharged from the health facility, a specimen will be collected to help identify the bacteria that is causing the infection. This may be blood sample, a urine sample, or a sample taken from the site of the infection (for example, pus from a wound or breast milk in the case of a breast infection).

It will take no more than 30 minutes to collect health information about you and your baby. In the case of suspected infection, it may take an additional 30 minutes to collect samples from you or your baby.

After delivery, a member of the study team will visit you a total of three times to assess the health status of you and your baby. These visits will be scheduled to take place at your home or a health facility at 3 days, 1 week (7 days) and 6 weeks (42 days) after delivery. If you or your baby are hospitalized at the scheduled time, the study team will visit you in the hospital. During the visit, the study team will assess you and your baby for current or past signs of infection. This will include collecting health information and measuring temperature for you and your baby. Each visit will take approximately 30 minutes.

A member of the study team will also contact you by phone at 14 days and 28 days after delivery to review the signs and symptoms of infection. If you do not have a phone, the study team will arrange to visit you at home or at the study facility. Each phone contact will take approximately 15 minutes.

If a sign of infection is identified in you or your baby during the in-person visits or phone contact, you will be referred a health facility for further assessment. You will also be asked to provide a sample, such as blood, pus, or urine, to help identify the bacteria causing the infection. The samples will be labelled with a study identification number in place of your name or your baby's name. Only dedicated members of our study team will have access to the samples.

To ensure that we have accurate and complete information about the health of you and your baby, we will access and collect information from the medical records at the health facilities where you and your baby have received care. By agreeing to participate in this study, you are also agreeing to give permission for the study staff to access your medical records. We will take precautions to protect the information that is collected from your medical records. Only study staff will have access to this information. To further protect you and your baby, all of your information will be coded with a number in place of your name.

For peer review only - http://bmjopen.bmj.com/site/about/guidelines.xhtml

The local research staff have been selected because of their skills, knowledge, and familiarity with your community. The research staff are here to support you during the study and should be contacted between visits if you have any questions or concerns.

What are the risks and discomforts

Azithromycin is a sometimes used to treat infections in pregnant women and children. Research shows that the risks of taking azithromycin are minimal. A commonly reported side effect is gastric discomfort (nausea, stomach pain, diarrhea, vomiting); however, you will be given pills with a special coating which will help prevent stomach discomfort. There is also a small but rare risk that azithromycin could cause arrythmia (irregular or abnormal heartbeat) or an allergic reaction; therefore, you will not be able to participate if you have a known history of heart problems or have had a bad reaction to azithromycin or a similar drug in the past.

If it is necessary to take a sample because of infection, you or your baby may feel temporary discomfort, but this will only last a few seconds. To minimize this, we will ensure research staff are well trained in the procedure.

Another possible risk of participating in this study is that your name and personal information may be seen by persons who are not part of the project. To prevent this, you will be given an identification number that will be used in place of your name on all study documents.

Information from this research study will be retained by [local institution] and RTI International in the United States (U.S.) and in the future may be included in a de-identified public use database managed by NICHD Data and Specimen Hub (DASH) in compliance with the U.S. National Institutes of Health (NIH) Public Access Policy. De-identified means that you and your baby will not be individually identified by name or other personal identifiers in the database. Your full name or any address details will not be included. Information released will not identify you or your baby's participation in this research study.

What are the benefits of participating?

You will not receive any money from participating in this study, but your participation may provide important information that can be used in the future to prevent infection in mothers and babies. Also, there is preliminary information suggesting that the use of azithromycin in pregnancy can reduce the risk of maternal and infant infection.

If new information about the benefits or risks of azithromycin use in pregnancy becomes available during the study, this information will be given to you by [Insert name of Senior Investigator] or his/her staff.

Will I have to pay for anything?

It will not cost you anything to be in the study.

Is my participation voluntary?

Taking part in this study is voluntary. You have the right to refuse to participate or to withdraw your participation at any time. If you refuse or decide to withdraw, you will not lose any benefits or rights to which you are entitled. These actions will not have any negative effect on the health care you receive from your local health providers. You will still receive your normal medical care.

Can I be removed from this study?

For peer review only - http://bmjopen.bmj.com/site/about/guidelines.xhtml

You will be withdrawn from the study if the research staff thinks that your participation may cause harm to you or your baby. The research staff may also remove you from the study for other reasons at their discretion. Also, the sponsor may stop the study at any time.

What will happen if you are injured by this research?

Although the risk of injury is expected to be very low, all research involves a chance that something bad might happen to you. Despite all safety measures, your participation could result in a reaction or injury. If you or your infant is injured as a result of your participation, you will be provided with emergency care by the study and referred to a doctor for ongoing care, if needed. Ongoing care will not be paid for by the study. [Insert name of Research Institution] and NICHD have not set aside funds to pay you for any such reactions, injuries or related medical care. However, by signing this form, you do not give up any of your legal rights.

What should you do if you have additional questions?

If you have questions about this study or a project-related injury, you should contact [investigator contact]. If you have questions about your or your baby's rights as a project participant, please contact [insert ethics committee contact].

Agreement to be in this study

I have read this paper about the study or it was read to me. I understand the possible risks and benefits of this study. I know that being in this study is voluntary and I choose to be in this study. I understand I will get a copy of this consent form.

Signature (or thumbprint):____ Date:________________

(Mother)

Print Name:____

(Mother)

Signature (or thumbprint):____ Date:________________

(Parent/Guardian/Husband)

Print Name:____

(Parent/Guardian/Husband)

Future Contact

We may wish to contact you in the future about participating in other related research studies. Please indicate below if you are willing to be contacted.

☐ Yes, I agree to be contacted

☐ No, I do not agree to be contacted

For peer review only - http://bmjopen.bmj.com/site/about/guidelines.xhtml

For peer review only

Appendix 3. Schedule of Study Procedures

	ANC Visits	During Labor/ Before Delivery	After Delivery/ Before Discharge	3-day postpartum (pp)	7-day pp	14-day pp	28-day pp	42-day pp	As Needed
**Community Sensitization**	X								
**Screening**									
• *Eligibility confirmation*		X							
• *Clinical assessment*		X							
**Consent**		X							
**Randomization**		X							
**Drug Administration**		X							
**Baseline Data Collection**									
• *Sociodemographic information*		X	X						
• *Medical history*		X	X						
• *Labor and delivery information*			X						
**Monitoring**									
• *Drug side effects*		X	X	X	X			X	X
• *Maternal events during labor/delivery*		X	X						
• *Neonatal events during labor/delivery*			X						
• *Maternal infection/sepsis assessment*			X	X	X			X	X
• *Neonatal infection/sepsis assessment*			X	X	X			X	X
• *Maternal death*			X	X	X			X	X
• *Stillbirth or neonatal death within 28 days of birth*			X	X	X	X	X	X	X
• *Infant mortality after 28 days of birth*								X	X
• *Pyloric stenosis*			X	X	X			X	X
• *Other maternal outcomes*			X	X	X			X	X
• *Other neonatal outcomes*			X	X	X			X	X
• *Unintended medical visits*			X	X	X			X	X
**Serious Adverse Events**			X	X	X			X	X

For peer review only - http://bmjopen.bmj.com/site/about/guidelines.xhtml

BMJ Open

Prevention of maternal and neonatal death/infections with a single oral dose of azithromycin in women in labor in low- and middle-income countries (A-PLUS): Study protocol for a multi-national, randomized placebo-controlled clinical trial

Journal:	*BMJ Open*
Manuscript ID	bmjopen-2022-068487.R2
Article Type:	Protocol
Date Submitted by the Author:	12-May-2023

Complete List of Authors:	Hemingway-Foday, Jennifer; RTI International<br>Tita, Alan; The University of Alabama at Birmingham School of Medicine<br>Chomba, Elwyn; University of Zambia University Teaching Hospital<br>Mwenechanya, Musaku; University of Zambia University Teaching Hospital<br>Mweemba, Trecious; University of Zambia University Teaching Hospital<br>Nolen, Tracy; RTI International<br>Lokangaka, Adrien; University of Kinshasa<br>Tshefu Kitoto, Antoinette; University of Kinshasa<br>Lomendje, Gustave; Kinshasa School of Public Health<br>Hibberd, Patricia; Boston University School of Public Health<br>Patel, Archana; Lata Medical Research Foundation; Datta Meghe Institute of Higher Education & Research (Deemed to be University)<br>Das, Prabir Kumar; Lata Medical Research Foundation, Nagpur<br>Kurhe, Kunal; Lata Medical Research Foundation<br>Goudar, Shivaprasad S.; KLE Academy of Higher Education and Research<br>Kavi, Avinash; KLE Academy of Higher Education and Research<br>Metgud, Mrityunjay; KLE Academy of Higher Education and Research<br>Saleem, Sarah; The Aga Khan University, Community Health Sciences<br>Tikmani, Shiyam; The Aga Khan University, Community Health Sciences<br>Esamai, Fabian; Moi University School of Medicine<br>Nyongesa, Paul; Moi University School of Medicine, Reproductive Health<br>Sagwe, Amos; Moi University School of Medicine<br>Figueroa, Lester; Institute of Nutrition of Central America and Panama<br>Mazariegos, Manolo; Instituto de Nutricion de Centroamerica y Panama<br>Billah, Sk Masum; International Centre for Diarrhoeal Disease Research, Maternal and Child Health Division; The University of Sydney<br>Haque, Rashidul; International Centre for Diarrhoeal Disease Research Bangladesh, Centre for Communicable Diseases<br>Shahjahan Siraj, Md; International Centre for Diarrhoeal Disease Research Bangladesh<br>Goldenberg, R; Columbia University<br>Bauserman, Melissa; University of North Carolina at Chapel Hill School of Medicine<br>Bose, Carl; The University of North Carolina at Chapel Hill School of Medicine<br>Liechty, Edward A; Indiana University School of Medicine<br>Ekhaguere, Osayame; Indiana University Department of Pediatrics,

	Neonatal-Perinatal Medicine<br>Krebs, Nancy F; University of Colorado Denver<br>Derman, Richard ; Thomas Jefferson University, Office of Global Affairs<br>Petri, William A.; University of Virginia<br>Koso-Thomas, Marion; Eunice Kennedy Shriver National Institute of Child Health and Human Development<br>McClure, Elizabeth; RTI International<br>Carlo, Waldemar; The University of Alabama at Birmingham, Pediatrics
**Primary Subject Heading**:	Global health
Secondary Subject Heading:	Public health, Obstetrics and gynaecology, Paediatrics
Keywords:	Maternal medicine < OBSTETRICS, NEONATOLOGY, OBSTETRICS, PUBLIC HEALTH, Clinical trials < THERAPEUTICS

SCHOLARONE™
Manuscripts

Prevention of maternal and neonatal death/infections with a single oral dose of azithromycin in women in labor in low- and middle-income countries (A-PLUS): Study protocol for a multi-national, randomized placebo-controlled clinical trial

Jennifer J Hemingway-Foday[1], Alan Tita[2], Elwyn Chomba[3], Musaku Mwenechanya[3], Trecious Mweemba[3], Tracy L Nolen[1], Adrien Lokangaka[4], Antoinette Tshefu[4], Gustave Lomendje[4], Patricia L Hibberd[5], Archana Patel[6,7], Prabir Das[6], Kunal Kurhe[6], Shivaprasad S. Goudar[8], Avinash Kavi[8], Mrityunjay Metgud[8], Sarah Saleem[9], Shiyam Sunder Tikmani[9], Fabian Esamai[10], Paul Nyongesa[10], Amos Sagwe[10], Lester Figueroa[11], Manolo Mazariegos[11], Sk Masum Billah[12,13], Rashidul Haque[12], Md Shahjahan Siraj[12], Robert L Goldenberg[14], Melissa Bauserman[15], Carl L Bose[15], Edward A Liechty[16], Osayame Austine Ekhaguere[16], Nancy F. Krebs[17], Richard J Derman[18], William A Petri Jr[19], Marion Koso-Thomas[20], Elizabeth M McClure[1], Waldemar A Carlo[2]

[1]RTI International, Durham, NC, USA
[2]University of Alabama at Birmingham, Birmingham, AL, USA
[3]University Teaching Hospital, Lusaka, Zambia
[4]Kinshasa School of Public Health, Kinshasa, Democratic Republic of Congo
[5]Boston University School of Public Health, Boston, MA, USA
[6]Lata Medical Research Foundation, Nagpur, India
[7]Datta Meghe Institute of Medical Sciences, Wardha, India
[8]KLE Academy Higher Education and Research, J N Medical College Belagavi, Karnataka, India
[9]Aga Khan University, Karachi, Pakistan
[10]Moi University School of Medicine, Eldoret, Kenya
[11]Instituto de Nutrición de Centroamérica y Panamá, Guatemala City, Guatemala
[12]International Centre for Diarrhoeal Disease Research, Dhaka, Bangladesh
[13]University of Sydney, Sydney, NSW, Australia
[14]Columbia University School of Medicine, New York, NY, USA
[15]University of North Carolina at Chapel Hill, Chapel Hill, NC, USA
[16]Indiana School of Medicine, University of Indiana, Indianapolis, IN, USA
[17]University of Colorado School of Medicine, Denver, CO, USA
[18]Thomas Jefferson University, Philadelphia, PA, USA
[19]University of Virginia, Charlottesville, VA, USA
[20]*Eunice Kennedy Shriver* National Institute of Child Health and Human Development, Bethesda, MD,USA

Corresponding Author:
Jennifer J. Hemingway-Foday, MPH, MSW
RTI International
3040 E. Cornwallis Road
Research Triangle Park, NC, 27709
Telephone: (919)-485-2663
Email: hemingway@rti.org

For peer review only - http://bmjopen.bmj.com/site/about/guidelines.xhtml

Word count (excluding title page, abstract, references, figures and tables): 3998

ABSTRACT

Introduction

Maternal and neonatal infections are among the most frequent causes of maternal and neonatal mortality, and current antibiotic strategies have been ineffective in preventing many of these deaths. A randomized clinical trial conducted in a single site in The Gambia showed that treatment with oral dose of 2 g azithromycin vs. placebo for all women in labor reduced certain maternal and neonatal infections. However, it is unknown if this therapy reduces maternal and neonatal sepsis and mortality. In a large, multi-national randomized trial, we will evaluate the impact of azithromycin given in labor to improve maternal and newborn outcomes.

Methods and Analysis

This randomized, placebo-controlled, multicenter clinical trial includes two primary hypotheses, one maternal and one neonatal. The maternal hypothesis is to test whether a single, prophylactic intrapartum oral dose of 2 g azithromycin given to women in labor will reduce maternal death or sepsis. The neonatal hypothesis will test whether this intervention will reduce intrapartum/neonatal death or sepsis. The intervention is a single, prophylactic intrapartum oral dose of 2 g azithromycin, compared with a single intrapartum oral dose of an identical appearing placebo. A total of 34,000 laboring women from eight research sites in sub-Saharan Africa, South Asia, and Latin America will be randomized with one-to-one ratio to intervention/placebo. In addition, we will assess antimicrobial resistance in a sample of women and their newborns.

Ethics and Dissemination

The study protocol has been reviewed and ethics approval obtained from all the relevant ethical review boards at each research site. Results will be disseminated via peer-reviewed journals and national and international scientific forums.

Trial Registration

ClinicalTrials.gov number: NCT03871491

https://clinicaltrials.gov/ct2/show/NCT03871491?term=NCT03871491&draw=2&rank=1

Keywords: sepsis, azithromycin, low and middle-income countries; neonatal mortality; maternal mortality

For peer review only - http://bmjopen.bmj.com/site/about/guidelines.xhtml

STRENGTHS AND LIMITATIONS OF THIS STUDY

- This study evaluates the use of routine prophylactic azithromycin in women who undergo labor and attempt a vaginal delivery, including those at high risk of infection due to prolonged labor or membrane rupture, which was identified as a top research priority by the World Health Organization (WHO) and other stakeholders.
- The study evaluates a simple, inexpensive intervention that can be scaled up to reduce the burden of both maternal and neonatal mortality due to infections if successful.
- To optimize generalizability, this study will be carried out in a racially, ethnically, and geographically diverse population in seven low-and-middle-income countries.
- In response to global public health concerns about increasing antimicrobial resistance, this study includes an ancillary study to assess antimicrobial resistance and microbiome changes.
- In some locations, azithromycin and other antibiotics are increasingly being used, especially with increasing use of cesarean section, and thus the impact of the outcome may be diminished.

INTRODUCTION

Background

Maternal infection during pregnancy and the puerperium, accounting for 10% of maternal deaths, is a top cause of maternal mortality worldwide.[1] Maternal infections increase neonatal sepsis, a leading cause of neonatal death in low-income countries (LICs).[2-5] Neonatal infection accounts for 15% of neonatal mortality worldwide.[2] According to the World Health Organization (WHO), maternal and neonatal deaths from infections have remained unchanged or increased, whereas other causes of death have declined. [5,6]

Innovative, effective, and scalable interventions are needed to reduce infection-related maternal and neonatal mortality. The evidence backing current WHO guidelines to prevent and treat peripartum infections is limited.[7] Further, current approaches to address neonatal sepsis have had limited impact and alternative antibiotic regimens suggesting comparable effectiveness have not reduced newborn deaths.[8-11]

Risk Factors for Maternal and Neonatal Infection and Sepsis

Cesarean delivery (CD), especially after labor or membrane rupture, is an important risk factor for maternal peripartum infection.[12] Antibiotic prophylaxis for CD is a well-established, effective strategy to reduce infection.[13-16] However, with a prevalence of <5% in LICs, CDs do not account for most maternal sepsis worldwide.[17] Many peripartum infections in low-and-middle-income countries (LMICs) occur in women who deliver vaginally, with women undergoing prolonged labor or membrane rupture at highest risk. Although most pregnancies are not at high-risk for infection due to prolonged labor or membrane rupture, pregnancies where this does occur account for about 50% of maternal and neonatal infections in LMICs.[1] Therefore, identification and management of prolonged labor and/or membrane rupture are critical to reduce maternal sepsis.[7] Thus, the WHO has identified evaluation of routine prophylactic antibiotics for women undergoing vaginal birth or with prolonged labor/membrane rupture as a top research priority.[1]

Intrapartum Azithromycin to Prevent Maternal and Neonatal Infections

For peer review only - http://bmjopen.bmj.com/site/about/guidelines.xhtml

A novel approach to prevent maternal and neonatal infection is to target organisms that may be frequent pathogens but are not historically treatment targets.[16, 18-21] Azithromycin, which is available as a generic agent, has a bimodal half-life of 70 hours in the non-pregnant population. It is commonly used during pregnancy to treat chlamydia, gonorrhea, and other infections.[22] It provides broad coverage against most common pathogens associated with peripartum infections including gram-positive cocci, genital mycoplasmas and *Ureaplasma* infections, and certain gram-negative Bacilli and anaerobes associated with maternal polymicrobial infections and sepsis.[23] In addition, azithromycin also has activity against Group B *streptococcus* (GBS), a major cause of neonatal sepsis in developed countries and possibly in LICs.[24,25]

Evidence from Prior Studies

In a U.S. randomized clinical trial (RCT), a 50% reduction in maternal peripartum infection resulted from 500 mg intravenous azithromycin added to the standard prophylactic regimen in high-risk women undergoing CD following labor or membrane rupture ≥4 hours.[18] These results were observed despite universal antibiotics in both treatment groups. Readmission, unscheduled visits, serious adverse events, postpartum fever, or subsequent antibiotic treatment were also reduced in the azithromycin group. A related cost-analysis estimated that adjunctive azithromycin saved $360 USD per use in high-risk CDs and $143 USD per use in scheduled CDs.[26]

A trial in The Gambia suggested the potential for azithromycin prophylaxis to improve maternal and neonatal outcomes.[19] Among 829 participants randomized to 2 grams (g) of azithromycin vs. placebo before delivery, maternal infections were lower in the azithromycin group, 3.6% vs 9.2%, respectively (RR 0.40, 95% CI, 0.22-0.71, P=0.002). Among newborns, infections were also lower in the azithromycin group (18.1% vs 23.8%; RR 0.76 95% CI, 0.58-0.99; P=0.052). Maternal and neonatal carriage of infectious organisms was lower in the azithromycin group.

Rationale for a trial

Drawing from these findings, the role of a single oral dose of azithromycin (plus usual care) to prevent maternal death or peripartum sepsis and intrapartum/neonatal death or sepsis appears promising. Of interest is the sub-population at highest risk for infection due to prolonged labor and/or prolonged membrane rupture. Considering the success with a single 2g dose in The Gambian trial, which is bioequivalent to the 500 mg intravenous dose used successfully in the US trial, as well as the 40% bioavailability of oral azithromycin, 2g is considered the most appropriate dose for the proposed intervention. A RCT is the best design to evaluate efficacy and provide evidence for future policy and clinical practice decisions. Such a RCT in a racially, ethnically, and geographically diverse population could be accomplished through the established infrastructure of the Global Network for Women's and Children's Health Research (Global Network), a multi-country research network with sites in India, Pakistan, Bangladesh, Guatemala, Kenya, Zambia, and the Democratic Republic of Congo (DRC) and an established data coordinating center (DCC).[27]

METHODS AND ANALYSIS

Study Design and Intervention

This study, the Prevention of Maternal and Neonatal Death/Infections with a Single Oral Dose of Azithromycin in Women in Labor in Low- and Middle-Income Countries (A-PLUS), is a masked, placebo-controlled, multicenter, RCT. The intervention is a single 2g dose of oral azithromycin or identical placebo, administered as four 500 mg pills following randomization. Both groups will also receive the

For peer review only - http://bmjopen.bmj.com/site/about/guidelines.xhtml

local standard of care during labor, delivery, and postpartum. The trial adheres to the Standard Protocol Items: Recommendations for Interventional Trials (SPIRIT) statement [28].

Primary Hypothesis

The A-PLUS trial has both a maternal and neonatal primary hypothesis:

- Maternal: A single, prophylactic intrapartum oral dose of 2g azithromycin given to women in labor will reduce *maternal* death or sepsis.
- Neonatal: A single, prophylactic intrapartum oral dose of 2g azithromycin given to women in labor will reduce *intrapartum/neonatal* death or sepsis.

Study Population

Pregnant women laboring in health facilities at eight Global Network sites will be screened by research staff per the eligibility criteria (**Table 1**). The Global Network sites are described in Appendix 1 of the trial protocol (see supplemental file 1). The secondary population is a cohort of 5,500 high-risk women, defined as term and preterm (≥28 weeks) pregnant women with prolonged labor (≥18 hours) or prolonged membrane rupture (≥8 hours). A cohort of mother-infant dyads will also be randomly selected for an ancillary study to assess antimicrobial resistance and microbiome changes. Recruitment took place from September 2020- August 2022 and data collection for the primary outcome was completed in October 2022. Data collection for the ancillary study of antimicrobial resistance is expected to be complete by August 2023.

Table 1. Eligibility Criteria

**Inclusion Criteria**
• Pregnant women in labor with gestational age ≥ 28 weeks (by best estimate) who plan to deliver vaginally in a facility
• Admission to health facility with clear plan for spontaneous or induced vaginal delivery
• Presence of one or more live fetus confirmed via a fetal heart rate by Doptone prior to randomization
• Age ≥ 18 years of age (minors 14-17 years eligible in countries where married or pregnant minors or their authorized representatives are legally permitted to give consent)
• Provision of written informed consent*
**Exclusion Criteria**
• Non-emancipated minors (as per local regulations)
• Evidence of chorioamnionitis or other infection requiring antibiotic therapy at time of eligibility *[women given single prophylactic antibiotics with no plans to continue after delivery will not be excluded]*
• Arrhythmia or known history of cardiomyopathy
• Allergy to azithromycin or other macrolides that is self-reported or documented in the medical record.
• Any use of azithromycin, erythromycin, or other macrolide in the 3 days or less prior to randomization.
• Plan for Cesarean delivery prior to enrollment
• Preterm labor undergoing management with no immediate plan to proceed to delivery
• Advanced stage of labor (>6 cm or 10 cm cervical dilation per local standards) and pushing or too distressed to understand, confirm, or give informed consent regardless of cervical dilation
• Not capable of giving consent due to other health problems such as obstetric emergencies (for example, antepartum hemorrhage) or mental disorder
• Any medical condition considered a contraindication per the judgement of site investigators
• Previous randomization in the trial
* A model written informed consent form is available as Appendix 2 of the trial protocol (see supplemental file 1)

For peer review only - http://bmjopen.bmj.com/site/about/guidelines.xhtml

Patient and Public Involvement

Patients and/or the public were not involved in study design, conduct, or reporting.

Primary Outcomes

The primary outcomes are:

- Maternal: Incidence of maternal death or sepsis within 42 days post-delivery in the intervention vs. placebo group.
- Neonatal: Incidence of intrapartum/neonatal death or sepsis with 28 days post-delivery in the intervention vs. placebo group.

We use the 2017 WHO definition of sepsis which includes a suspicion of infection and the presence of organ dysfunction based on clinical findings.[7]

Maternal sepsis is defined as organ dysfunction resulting from suspected or confirmed infection that occurs post-randomization during labor or the postpartum period. This WHO definition is operationalized as suspected or confirmed infection based on the presence of fever or hypothermia plus one or more signs of mild to moderate organ dysfunction including tachycardia, low blood pressure, tachypnea, altered mental status/confusion, reduced urinary output, jaundice, or renal failure.[7, 29-31] Components of peripartum infection considered in diagnosing suspected or confirmed infection include clinical chorioamnionitis, endometritis, wound infections (perineal or cesarean), abdominal or pelvic abscess, mastitis/breast abscess or infection, pyelonephritis, pneumonia, and other bacterial infections (**Table 2).**

Table 2. Specified Infections Considered for Maternal Sepsis Diagnosis

Type of Infection	A-PLUS Trial Definition
Chorioamnionitis	Fever (>100.4°F/38°C) in addition to one or more of the following: fetal tachycardia ≥160 bpm, maternal tachycardia >100 bpm, tender uterus between contractions, or purulent/foul smelling discharge from uterus **prior** to delivery.
Endometritis	Fever (>100.4°F/38°C) in addition to one or more of maternal tachycardia >100 bpm, tender uterine fundus, or purulent/foul smelling discharge from uterus **after** delivery.
Wound infection	Purulent infection (superficial or deep infection including necrotizing fasciitis) of a perineal or Cesarean wound with or without fever. In the absence of purulence, a wound infection requires presence of fever (>100.4°F/38°C) and at least one of the following signs of local infection: pain or tenderness, swelling, heat, or redness around the incision/laceration
Abdominopelvic abscess	Evidence of pus in the abdomen or pelvis noted during open surgery, interventional aspiration or imaging
pneumonia	Fever (>100.4°F/38°C) and clinical symptoms suggestive of lung infection including cough and/or tachypnea (>24 breaths/min) or radiological confirmation
pyelonephritis	Fever (>100.4°F/38°C) and one or more of the following: urinalysis/dip suggestive of infection, costovertebral angle tenderness, or confirmatory urine culture
mastitis/breast abscess or infection	Fever (>100.4°F/38°C) and one or more of the following: breast pain, swelling, warmth, redness, or purulent drainage

Neonatal sepsis is defined as proven or possible serious bacterial infection (pSBI) or pneumonia, or meningitis. PSBI will be determined using WHO criteria of severe chest in-drawing, fever, hypothermia, no movement at all or movement only on stimulation, feeding poorly or not feeding at all, and/or convulsions.[32] Clinical and laboratory signs of infection will also be considered for diagnosis.

For peer review only - http://bmjopen.bmj.com/site/about/guidelines.xhtml

Centralized adjudication of key infection outcomes will be implemented to standardize the results. Reported antibiotic treatment and culture status will also be considered for outcome adjudication.

The individual components of these primary outcomes will be analyzed.

Secondary maternal outcomes

- The primary maternal outcome in a high-risk for infection population
- Incidence of specific maternal infections (Table 2)
- Subsequent maternal antibiotic therapy after randomization to 42 days postpartum for any reason
- Time from drug administration until initial discharge after delivery
- Maternal readmissions and admissions to special care units within 42 days postpartum
- Maternal unscheduled visit for care
- Maternal gastrointestinal symptoms (nausea, vomiting, diarrhea) or other reported side effects
- Maternal death due to sepsis using the Global Network algorithm for cause of death[33]

Secondary Neonatal Outcomes

- Neonatal deaths due to sepsis and all-cause neonatal death in all participants and in laboring women at high risk of infection
- Other neonatal infections (e.g., eye infection, skin infection, omphalitis, urinary tract infection, respiratory rate ≥60 breaths/minute).
- Neonatal readmissions and admissions to special care units within 42 days postpartum
- Neonatal initial hospital length of stay, defined as time of delivery until initial discharge
- Neonatal unscheduled visit for care
- Neonatal death due to sepsis using the Global Network algorithm for cause of death[34]
- Incidence of pyloric stenosis within 42 days of delivery

Study Procedures

All sites will train research staff in standardized study procedures to administer the intervention and assess outcomes. The sequence of study activities is described (**Figure 1)**. Study procedures are also provided (**Table 3)**. A more detailed schedule of study procedures is available as Appendix 3 of the trial protocol (see supplemental file 1).

Site Preparation

Prior to the trial, sites conducted an observational pilot study using the RCT's planned infrastructure to characterize current practices and optimize identification of suspected infection. The pilot study data was used to validate estimates of intrapartum deaths, maternal sepsis, and neonatal sepsis for the RCT's sample size. Each site also met with local health authorities and conducted community sensitization to ensure that study procedures were appropriate for the local context and to encourage facility and community-level engagement.

Screening

Pregnant women admitted for delivery at pre-defined health facilities will be screened for eligibility by research staff, per harmonized criteria (**Table 1**). If a contraindication to participation is found, the woman will be excluded.

For peer review only - http://bmjopen.bmj.com/site/about/guidelines.xhtml

Table 3. Schedule of Study Procedures

	ANC Visits	During Labor/ Pre-delivery	Post-delivery/ Pre-discharge	3-day post-partum (pp)	7-day pp	14-day pp	28-day pp	42-day pp
**Community Sensitization**	X							
**Screening**								
▪ *Eligibility confirmation*		X						
▪ *Clinical assessment*		X						
**Consent**		X						
**Randomization**		X						
**Drug Administration**		X						
**Baseline Data Collection**								
▪ *Sociodemographic information*		X	X					
▪ *Medical history*		X	X					
▪ *Labor & delivery (L&D) information*			X					
**Monitoring**								
▪ *Drug side effects**		X	X	X	X			X
▪ *Maternal events during L&D*		X	X					
▪ *Neonatal events during L&D*			X					
▪ *Maternal infection/sepsis**			X	X	X			X
▪ *Neonatal infection/sepsis**			X	X	X			X
▪ *Maternal death**			X	X	X			X
▪ *Stillbirth or neonatal death < 28 days of birth**			X	X	X	X	X	X
▪ *Infant mortality ≥ 28 days of birth**								X
▪ *Pyloric stenosis**			X	X	X			X
▪ *Other maternal outcomes**			X	X	X			X
▪ *Other neonatal outcomes**			X	X	X			X
▪ *Unintended medical visits**			X	X	X			X
**Serious Adverse Events***			X	X	X			X
* Indicates events that may be reported by participant between scheduled study visits.								

Consent

Research staff at each site are responsible for obtaining informed consent prior to complete cervical dilation or dilation limit approved by local authorities. Participants who are randomly selected to participate in the ancillary study on antimicrobial resistance will also be consented at this time. To minimize burden on the laboring women, sites may obtain initial consent from potential participants during the antenatal period and reconfirm consent at screening. As literacy levels vary, the consent form will be reviewed verbally. All research staff responsible for consent procedures will be trained and certified in the protection of human subjects and the study-specific consent procedures.

Masking

Both the azithromycin and placebo will be procured from the same manufacturer (Idifarma, Spain). The packaging will be standardized across sites and labeled as: “Azithromycin 2 g or Placebo” with the expiration date and a unique identifier. Clinical and research staff, and participants, will be masked to treatment status unless a serious adverse event potentially related to the treatment requires unmasking. Each site’s study pharmacist will monitor randomization, drug supply, and safety.

For peer review only - http://bmjopen.bmj.com/site/about/guidelines.xhtml

Randomization

A computer-generated randomization scheme, created and known only by the DCC, will use a randomly permuted block design with randomly varied block to achieve a 1:1 randomization, stratified by site. Each site will receive study drug directly from the manufacturer, which is sequentially prepared per the randomization scheme in identical packaging. The study drug will be distributed to participating health facilities and dispensed sequentially by study staff.

Monitoring before Discharge (Baseline Assessment)

Routine post-delivery care will be provided by clinical providers, trained to contact research staff in case of suspected or confirmed infection. Research staff will collect demographic characteristics, key clinical measures, and outcomes from randomization until discharge. Data will be abstracted from medical records or collected directly from participants, as relevant. All data will be directly entered into a secure electronic data capture platform, Research Electronic Data Capture (REDCap). De-identified data will then be transmitted to the DCC. In addition, participants will be educated on signs and symptoms of infection and encouraged to call research staff with any concerns.

Monitoring after Discharge (Postpartum Follow-up Assessments)

After discharge, research staff will contact participants at the following timepoints:

- In-person visits at 3-, 7-, and 42-day postpartum to identify maternal or infant infection, unexpected medical visits, side effects, and other study outcomes. If suspected infection, research staff will collect and send relevant specimens to the local microbiology lab for analysis.
- Supplemental phone contacts at 14- and 28-day postpartum to review maternal and neonatal signs of infection (WHO criteria). If signs are identified, participants will visit a study facility for further assessment. These supplemental contacts will reinforce the participants' ability to self-assess maternal and neonatal infection and improve identification between the 7- and 42-day visits.

If indicated, records of non-study visits to health providers during the follow-up period will be reviewed to ascertain study outcomes. Providers may be called if clarification is needed. Readmissions and related diagnoses identified during follow-up visits will be validated through medical record review. All data will be entered into REDCap and de-identified data will be transmitted to the DCC.

Safety Monitoring

Surveillance of maternal side effects including nausea, vomiting, and diarrhea/loose stools, abdominal pain, vaginitis, and dizziness potentially associated with azithromycin will be conducted during labor and postpartum. For infants, findings suggestive of pyloric stenosis will be assessed during the follow up visits. Maternal and neonatal surveillance will also include assessment of unintended medical visits, maternal deaths, stillbirths, neonatal death <28 days of birth, and infant death >28 days. Additional maternal and neonatal risks associated with azithromycin use include anaphylaxis, allergic reactions (rash), liver failure, and arrhythmias. Although rare, these side effects will be monitored and reported as serious adverse events. All safety outcomes will be reviewed by the Data Monitoring Committee (DMC) appointed by NICHD.

Analytical Plan

Baseline demographic characteristics and key clinical measures will be compared between the treatment arms using contingency table approaches for categorical variables and analysis of variance models' continuous variables.

For peer review only - http://bmjopen.bmj.com/site/about/guidelines.xhtml

P-values presented will be based on two-sided tests unless otherwise specified, adjusted for site. For most analyses, the interaction between treatment and site will be assessed and if significant, also be presented by site. For continuous outcomes, distributional properties will be evaluated and if required, transformations or non-parametric tests employed. Additional details for potential covariate adjustments in secondary analyses or handling violations of analytic method assumptions will be detailed in the statistical analysis plan.

Three key populations are of interest for study analyses:

1. The Intention to Treat (ITT) population will be the primary analysis population and will include all women randomized and their infants. Analyses of this cohort will be conducted based on randomized treatment.
2. The high risk for infection sub-group will include all women in the ITT and their infants' meeting criteria for being high risk (i.e., prolonged labor and/or rupture of membranes) at randomization. Analyses of this cohort will be conducted based on randomized treatment.
3. The as-treated population will include all randomized participants that receive any study drug during the study and their infants. Analyses of this cohort will be conducted based on treatment received.

The final determination of analysis population membership will be via a masked data review prior to final study analyses to address any potential anomalous cases that may arise in this large study population (e.g., randomization/treatment of a woman who is discharged prior to delivery due to false labor or unresponsiveness to induction).

Primary Analysis

Incidence of maternal death or sepsis and intrapartum/neonatal death or sepsis will be compared separately between the treatment arms using generalized linear models fit with each binary outcome separately as the outcome measure. Estimates of RR and associated 95% CIs will be reported. The model will include terms for treatment and site. As randomization occurs at the pregnancy level and approximately 1-2% of pregnancies are anticipated to be multiple gestations, models for neonatal outcomes will account for correlation among multiples assuming an exchangeable covariance structure. For the two primary outcomes, analyses will be conducted using the ITT population and the p-values associated with the treatment term will be used to formally test each of the two primary hypotheses at the alpha=0.05 level.

As secondary analyses of the primary outcomes, assuming an overall treatment effect is observed, the models will be run including region (Africa, Latin America, or Asia) and a treatment by region interaction term. If the interaction term has a $p<0.1$, effects will be reported by region with treatment effect within region tested at the 0.025 level.

Additional exploratory models will also be run including individually: 1) a treatment-by-site interaction term, 2) any other antibiotic use during labor (yes or no) and its interaction with treatment, and 3) mode of delivery (cesarean or vaginal) and its interaction with treatment. If the interaction term for any of these models has a $p<0.1$, then effects will also be reported by the relevant subgroups.

From each final model, estimates of relative risk associated with treatment will be obtained including unadjusted estimates of risk from the primary model as well as estimates of risk adjusted for potential confounders from the secondary analyses.

For peer review only - http://bmjopen.bmj.com/site/about/guidelines.xhtml

Secondary Analyses: Women at high-risk for infection cohort

The major secondary aim is assessing the two primary outcomes (i.e., incidence of maternal death or sepsis and incidence of intrapartum/neonatal death or sepsis) in the high-risk cohort. These analyses will also assess if the treatment effect differs between the high-risk sub-group vs. non-high-risk women, which include all women and their infants in the ITT population that delivered prior to meeting high-risk criteria. Specifically, the maternal and neonatal primary outcomes will be modeled including a treatment by risk status interaction term and exclude data from individuals meeting high-risk criteria after randomization.

Secondary Analyses: Other Secondary Outcomes

Other maternal and neonatal binary outcomes will be analyzed using the approaches described for the primary analysis for the ITT population and approaches detailed for the secondary analysis for the HR cohort. A similar process with generalized linear models employing an appropriate link function will be used to analyze the outcomes of maternal and neonatal initial hospital length of stay. Binary safety outcomes will also be analyzed using the approaches detailed for the primary analysis. These analyses will be conducted using the as-treated population.

Sample Size for Primary Outcome

Sample size estimates were generated to evaluate the potential benefits of peripartum prophylactic azithromycin in two population cohorts of women (**Table 4**). The first population comprises all women delivering in facilities.

Table 4. Sample Sizes

**4A. Sample Size for the Overall Population (alpha=0.05)**				
		Evaluable Sample Size per Arm		
Baseline Risk	Risk Reduction	Power=0.80	Power=0.85	Power=0.90
3%	20%	11455	13103	15334
3%	25%	7133	8159	9548
3%	30%	4815	5508	6446
8%	20%	4096	4686	5483
8%	25%	2554	2921	3419
8%	30%	1727	1975	2311
14%	20%	2204	2521	2950
14%	25%	1377	1575	1842
14%	30%	932	1066	1247
**4B. Sample Size for High-risk Group (alpha = 0.05)**				
6%	20%	5568	6369	7453
6%	25%	3470	3969	4644
6%	30%	2344	2681	3138
**4C. Sample Size by Region (alpha =0.025)**				
8%	20%	4961	5607	6477
8%	25%	3093	3496	4038
8%	30%	2091	2363	2729
14%	20%	2669	3017	3485
14%	25%	1677	1884	2176
14%	30%	1129	1276	1474

For peer review only - http://bmjopen.bmj.com/site/about/guidelines.xhtml

Power calculations for the overall study population were generated for the primary maternal and neonatal outcome measures. For each, estimates of the required sample size needed to detect a risk reduction of 20%, 25%, and 30% were generated for power of 0.8, 0.85, and 0.9. The risk of sepsis or maternal death was assumed to be 3%, based on data collected from 2010-2018 through the Global Network's Maternal Newborn Health Registry (MNHR), a prospective, population-based registry of pregnant women and neonates receiving care in defined Global Network catchment areas [27], augmented with active surveillance. For the neonatal outcome, the underlying risk of the combined outcome was estimated to be between 8% and 14% based on Global Network data. [35] We assumed that the risk of sepsis not resulting in death is approximately 4% to 10%. We will also test the neonatal risk separately in south Asia and sub-Saharan Africa. Approximately 37.5% of randomized mothers will be from sub-Saharan Africa and 50% will be from Asia, reflecting the rates observed in the Global Network.[35,36].

For the primary neonatal outcome of interest of intrapartum/neonatal sepsis or death, assuming that the loss to follow-up will be in the 2% to 3% range, this sample size of 34,000 will provide 90% power to detect a 25% reduction in neonatal mortality and sepsis in the sub-Saharan African region and will provide 90% power to detect a 20% reduction in Asia assuming the baseline risk is at least 8%. For the primary maternal outcome of maternal death or sepsis, the sample size will provide 90% power to detect a 20% reduction from 3% in the population aggregated across all study sites. Each site will aim to recruit an equal number of participants (n=4,250 per site) but site-specific sample sizes may be adjusted if targets are not met. No site will be permitted to recruit more than 20% of the overall study sample site.

Data Monitoring Plan and Stopping Rules

Each site will report data, including adverse events, to the Global Network's DCC. The data will be used to evaluate protocol adherence and site performance (e.g., recruitment, loss to follow-up, data quality). The DCC will provide standardized progress reports to NICHD and the site investigators monthly to monitor outcome variables and adverse events.

Trial oversight will be handled by two principal groups: (1) a protocol-focused Steering Committee (**Table 5**); and (2) a DMC, designated by NICHD to ensure safe and ethical treatment of participants through bi-annual (at minimum) review of data on participant safety, study progress, and futility. One formal interim analysis of efficacy and futility will be conducted by the DMC during the study.

Table 5. A-PLUS Steering Committee

**A-PLUS CENTRAL STUDY TEAM**	
A-PLUS Lead Study Site (University of Alabama-Birmingham/University Teaching Hospital, Lusaka, Zambia)	
Waldemar A. Carlo, MD<br>Principal Investigator<br>University of Alabama at Birmingham, United States	Alan Tita, MD, PhD<br>A-PLUS Lead Investigator<br>University of Alabama at Birmingham, United States
Elwyn Chomba, MBChB, DCH, MRCP<br>Senior Foreign Investigator<br>University Teaching Hospital, Lusaka, Zambia	Musaku Mwenechanya<br>Country Coordinator<br>University Teaching Hospital, Lusaka, Zambia
Trecious Mweemba<br>A-PLUS Coordinator<br>University Teaching Hospital, Lusaka, Zambia	
RTI International (Global Network Data Coordinating Center)	
Elizabeth M. McClure, PhD	Tracy Nolen, DrPh

For peer review only - http://bmjopen.bmj.com/site/about/guidelines.xhtml

Principal Investigator<br>RTI International	Co-Principal Investigator and Senior Statistician<br>RTI International
Jennifer J. Hemingway-Foday, MPH, MSW<br>A-PLUS Protocol Manager<br>RTI International	
Eunice Kennedy Shriver National Institute of Child Health and Human Development (NICHD)	
Marion Koso-Thomas, MD, MPH<br>Medical Officer, Global Network for Women's and Children's Health	
**A-PLUS RESEARCH SITES**	
Democratic Republic of Congo (Kinshasa School of Public Health/University of North Carolina)	
Antoinette Tshefu, M.D, Ph.D., M.P.H<br>Senior Foreign Investigator<br>Kinshasa School of Public Health, Kinshasa, DRC	Carl L. Bose, M.D.<br>Principal Investigator<br>University of North Carolina School of Medicine, U.S.
Adrien Lokangaka, M.D., M.P.H.<br>Country Coordinator<br>Kinshasa School of Public Health	Gustave Lomendje<br>A-PLUS Coordinator<br>Kinshasa School of Public Health
Guatemala (Instituto de Nutricion de Centro America y Panama [INCAP]/University of Colorado)	
Manolo Mazariegos, MD, MPH<br>Senior Foreign Investigator,<br>INCAP, Guatemala City, Guatemala	Nancy F. Krebs, MD<br>Principal Investigator,<br>University of Colorado Health Science Center
Lester Figueroa<br>Country Coordinator<br>INCAP, Guatemala City, Guatemala	
**Bangladesh (International Centre for Diarrhoeal Disease Research [ICDDR,b]/University of Virginia)**	
Rashidul Haque, MD<br>Senior Foreign Investigator<br>ICDDR,b	William Petri, MD<br>Principal Investigator<br>University of Virginia
Sk Masum Billah<br>Country Coordinator<br>ICDDR,b	Md Shahjahan Siraj<br>A-PLUS Coordinator<br>ICDDR,b
**Belagavi, India (KLE University's JN Medical College/Thomas Jefferson University)**	
Shivaprasad S. Goudar MD, MHPE<br>Senior Foreign Investigator<br>KLE University's J N Medical College	Richard Derman, MD, MPH<br>Principal Investigator<br>Thomas Jefferson University
Avinash Kavi<br>A-PLUS Coordinator<br>KLE University's J N Medical College	Mrityunjay Metgud<br>A-PLUS Coordinator<br>KLE University's J N Medical College
**Pakistan (Aga Khan University/Columbia University)**	
Sarah Saleem, MD<br>Senior Foreign Investigator<br>Aga Khan University	Robert L. Goldenberg, MD<br>Principal Investigator<br>Columbia University
Shiyam Sunder Tikmani<br>A-PLUS Coordinator<br>Aga Khan University	
**Nagpur, India (Lata Medical Research Foundation/Boston University)**	
Archana Patel, MD, DNB, MSCE<br>Senior Foreign Investigator<br>Lata Medical Research Foundation	Patricia L. Hibberd, MD, PhD<br>Principal Investigator<br>Boston University School of Public Health

For peer review only

For peer review only - http://bmjopen.bmj.com/site/about/guidelines.xhtml

Prabir Das<br>Country Coordinator<br>Lata Medical Research Foundation	Kunal Kurhe<br>A-PLUS Coordinator<br>Lata Medical Research Foundation
**Kenya (Moi University School of Medicine/University of Indiana School of Medicine)**	
Fabian Esamai, MBChB, MMed, PhD<br>Senior Foreign Investigator<br>Moi University School of Medicine, Eldoret, Kenya	Edward A. Liechty, MD<br>Principal Investigator<br>Indiana University School of Medicine
Paul Nyongesa<br>Country Coordinator<br>Moi University School of Medicine, Eldoret, Kenya	Amos Sagwe<br>A-PLUS Coordinator<br>Moi University School of Medicine, Eldoret, Kenya
Osayame Austine Ekhaguere<br>Co-Investigator<br>Indiana University School of Medicine	

Adverse Event Monitoring Plan

Adverse events will be reported and submitted to the DCC (and IRBs) who will report these cumulative masked data to the DMC in the bi-annual safety reviews. Safety reports will be reviewed internally by the DCC quarterly and the DMC chair will be notified if any potential safety signals are identified to allow for more frequent DMC monitoring if needed.

ETHICS AND DISSEMINATION

Ethics Approval and Consent to Participate

The A-PLUS study protocol has been reviewed and approved by the relevant ethics committees and regulatory authorities at each research site, including the Institutional Review Board at the University of Alabama at Birmingham, which serves as the lead site, and the DCC at RTI International *[See supplemental file 1 for current version 1.6, dated 13 July 2022].* The DMC has reviewed the study protocol and will continue to review throughout the enrollment period. The study was registered (clinicaltrials.gov NCT03871491).

All research staff responsible for obtaining informed consent will be trained and certified in the protection of human subjects and the study-specific consent procedures. A model written informed consent form, developed according to the requirements of the U.S. Office for Human Research Protection (OHRP), can be found as Appendix 2 of the trial protocol (see supplemental file 1). The model consent may be modified by each site to conform to local standards, but the OHRP required elements must be maintained.

Potential Risks and Benefits to Participation

There are several potential direct and indirect benefits of this trial. Emerging data suggest that intrapartum azithromycin reduces maternal and neonatal infection. As infections are a frequent cause of maternal and neonatal deaths, there is a possibility that mortality could be reduced at the participating sites, as well as worldwide.

An ongoing concern for peripartum and perinatal antibiotic prophylaxis is the selection of resistant organisms including azithromycin-resistant organisms leading to resistant infections, and concern that disruption of gut and other flora (microbiome) in women and particularly in neonates may lead to adverse events including allergic reactions, rash and childhood asthma.[16,28,37] There is a paucity of data to address concerns that disturbances in the establishment of the indigenous intestinal microbiome

For peer review only - http://bmjopen.bmj.com/site/about/guidelines.xhtml

caused by antibiotic exposure in early life or CD, either directly or through modifications of breast microbiome, may increase risk of immune-mediated and inflammatory conditions later in life.[38-41] In response to these global public health concerns, this protocol includes an ancillary study to monitor for antimicrobial resistance and changes to the maternal and newborn microbiome.

Dissemination

Following completion of primary data collection, we will disseminate findings through international meetings and high impact peer-reviewed journals. Per NIH and Global Network Data Management and Sharing Policies, final research data will also be made publicly available through the NICHD Data and Specimen Hub (DASH) system within one year of publishing the primary manuscript. All data will be de-identified and shared under the assurance of confidentiality and approval from the relevant IRBs. Trial results will also be shared with community members, health workers, health officials, and other stakeholders from the areas where participants were recruited. If the results are positive, we will facilitate practice change at participating sites and approach the WHO to evaluate guideline updates based on study findings.

Figure 1. Study Flowchart

For peer review only

For peer review only - http://bmjopen.bmj.com/site/about/guidelines.xhtml

References

1. WHO recommendations for the prevention and treatment of maternal peripartum infections. [Internet]. World Health Organization. World Health Organization; 2015 [retrieved 2018 Aug 22]. Available from: https://www.who.int/publications/i/item/9789241549363
2. Liu L, Oza S, Hogan D, Perin J, Rudan I, Lawn JE, et al. Global, regional, and national causes of child mortality in 2000-13, with projections to inform post-2015 priorities: an updated systematic review. Lancet 2015;385(9966), 430-440.
3. Fleischmann-Struzek C, Goldfarb DM, Schlattmann P, Schlapbach LJ, Reinhart K, Kissoon N. The global burden of paediatric and neonatal sepsis: a systematic review. Lancet Respir Med. 2018;6(3):223–30.
4. Fleischmann C, Reichert F, Cassini A, Horner R, Harder T, Markwart R, et al. Global incidence and mortality of neonatal sepsis: a systematic review and meta-analysis. Arch Dis Child. 2021;106(8):745-52.
5. Baguiya A, Bonet M, Cecatti JG, Brizuela V, Curteanu A, Minkauskiene M, et al; WHO Global Maternal Sepsis Study (GLOSS) Research Group; GLOSS research group. Perinatal outcomes among births to women with infection during pregnancy. Arch Dis Child. 2021;106(10):946-53.
6. Brizuela V, Cuesta C, Bartolelli G, Abdosh AA, Abou Malham S, Assarag B, et al; WHO GLOSS Research Group. Availability of facility resources and services and infection-related maternal outcomes in the WHO Global Maternal Sepsis Study: a cross-sectional study. Lancet Glob Health. 2021;9(9):e1252-e1261.
7. World Health Organization. (2017). Statement on maternal sepsis. Retrieved August 22, 2018, from http://www.who.int/reproductivehealth/publications/maternal_perinatal_health/maternalsepsis-statement/en/
8. Tshefu, A., Lokangaka, A., Ngaima, S., Engmann, C., Esamai, F., Gisore, P., . . . African Neonatal Sepsis Trial (AFRINEST) group. Simplified antibiotic regimens compared with injectable procaine benzylpenicillin plus gentamicin for treatment of neonates and young infants with clinical signs of possible serious bacterial infection when referral is not possible: a randomised, open-label, equivalence trial. Lancet 2015; 385(9979), 1767-1776.
9. Baqui AH, Saha SK, Ahmed AS, Shahidullah M, Quasem I, Roth DE, et al. Projahnmo Study Group in Bangladesh. Safety and efficacy of alternative antibiotic regimens compared with 7 day injectable procaine benzylpenicillin and gentamicin for outpatient treatment of neonates and young infants with clinical signs of severe infection when referral is not possible: a randomised, open-label, equivalence trial. Lancet Global Health 2015; 3(5), e279-287.
10. Mir F, Nisar I, Tikmani SS, Baloch B, Shakoor S, Jehan F, et al. Simplified antibiotic regimens for treatment of clinical severe infection in the outpatient setting when referral is not possible for young infants in Pakistan (Simplified Antibiotic Therapy Trial [SATT]): a randomised, open-label, equivalence trial. Lancet Global Health 2017; 5(2), e177-e185.
11. Zaidi AK, Tikmani SS, Sultana S, Baloch B, Kazi M, Rehman H, et al. Simplified antibiotic regimens for the management of clinically diagnosed severe infections in newborns and young infants in first-level facilities in Karachi, Pakistan: study design for an outpatient randomized controlled equivalence trial. Pediatric Infectious Disease Journal 2013; 32 Suppl 1(Suppl 1), S19-25.
12. World Health Organization. WHO Statement on Caesarean Section Rates. 2015. Retrieved August 22, 2018, from

For peer review only - http://bmjopen.bmj.com/site/about/guidelines.xhtml

http://www.who.int/reproductivehealth/publications/maternal_perinatal_health/cs-statement/en/
13. Mackeen AD, Packard RE, Ota E, Berghella V, Baxter JK. Timing of intravenous prophylactic antibiotics for preventing postpartum infectious morbidity in women undergoing cesarean delivery. Cochrane Database of Systematic Reviews 2014. 12(12), CD009516.
14. Smaill FM, Grivell RM. Antibiotic prophylaxis versus no prophylaxis for preventing infection after cesarean section. Cochrane Database of Systematic Reviews 2014. 10, CD007482.
15. Gyte GML, Dou L, Vazquez JC. Different classes of antibiotics given to women routinely for preventing infection at caesarean section. Cochrane Database of Systematic Reviews. 2014; 11(11), CD008726.
16. Tita ATN, Rouse DJ, Blackwell S, Saade GR, Spong CY, Andrews WW. Evolving concepts in antibiotic prophylaxis for cesarean delivery: A systematic review. Obstetrics and Gynecology. 2009;113(3):675–82
17. Harrison MS, Saleem S, Ali S, Pasha O, Chomba E, Carlo WA, et al. A Prospective, Population-Based Study of Trends in Operative Vaginal Delivery Compared to Cesarean Delivery Rates in Low- and Middle-Income Countries, 2010-2016. Am J Perinatol. 2019;36(7):730-736.
18. Tita ATN, Boggess K, et al. Adjunctive azithromycin prophylaxis for cesarean delivery. New Eng J Med 2015. 375:1231-1241.
19. Oluwalana C, Camara B, Bottomley C, Goodier S, Bojang A, Kampmann B et al. Azithromycin in labor lowers clinical infections in mothers and newborns: a double-blind trial. Pediatrics 2017, 139(2).
20. Hume-Nixon M, Quach A, Reyburn R, Nguyen C, Steer A, Russell F. A Systematic Review and meta-analysis of the effect of administration of azithromycin during pregnancy on perinatal and neonatal outcomes. EClinicalMedicine. 2021;40:101123.
21. Hallamaa L, Cheung YB, Maleta K, Luntamo M, Ashorn U, Gladstone M, Kulmala T, Mangani C, Ashorn P. Child Health Outcomes After Presumptive Infection Treatment in Pregnant Women: A Randomized Trial. Pediatrics. 2018;141(3):e20172459.
22. Fitzgibbon A, Clooney L, Broderick D, Eogan M, McCallion N, Drew RJ. Erythromycin compared to amoxicillin and azithromycin for antimicrobial prophylaxis for preterm premature rupture of the membranes: a retrospective study. J Obstet Gynaecol. 2021 May;41(4):569-572.
23. Sutton AL, Acosta EP, Larson KB, Kerstner-Wood CD, Tita AT, Biggio JR. Perinatal pharmacokinetics of azithromycin for cesarean prophylaxis. Am J Obstet Gynecol 2018; 212(6), 812.
24. Russell NJ, Seale AC, O'Driscoll M, O'Sullivan C, Bianchi-Jassir F, Gonzalez-Guarin J, et al; GBS Maternal Colonization Investigator Group. Maternal Colonization With Group B Streptococcus and Serotype Distribution Worldwide: Systematic Review and Meta-analyses. Clin Infect Dis. 2017 Nov 6;65(suppl_2):S100-S111.
25. Rac H, Gould AP, Eiland LS, Griffin B, McLaughlin M, Stover KR, Bland CM, Bookstaver PB. Common Bacterial and Viral Infections: Review of Management in the Pregnant Patient. Ann Pharmacother. 2019 Jun;53(6):639-651.
26. Harper LM, Kilgore M, Szychowski JM, Andrews WW, Tita ATN. Economic evaluation of adjunctive azithromycin prophylaxis for cesarean delivery. Obstet Gynec 2017; 130(2), 328-334.
27. McClure EM, Garces AL, Hibberd PL, Moore JL, Goudar SS, Saleem S, et al. The Global Network Maternal Newborn Health Registry: a multi-country, community-based registry of pregnancy outcomes. Reprod Health. 2020;17(Suppl 2):184.

For peer review only - http://bmjopen.bmj.com/site/about/guidelines.xhtml

28. Chan A-W, Tetzlaff JM, Gøtzsche PC, Altman DG, Mann H, Berlin J, Dickersin K, Hróbjartsson A, Schulz KF, Parulekar WR, Krleža-Jerić K, Laupacis A, Moher D. SPIRIT 2013 Explanation and Elaboration: Guidance for protocols of clinical trials. BMJ. 2013;346:e7586
29. Bonet, M., Souza, J. P., Abalos, E., Fawole, B., Knight, M., Kouanda, S., et al. The global maternal sepsis study and awareness campaign (GLOSS): study protocol. Reprod Health 2018; 15(1), 16.
30. Albright, C.M., Has, P., Rouse D.J., Hughes B.L. Internal Validation of the Sepsis in Obstetrics Score to Identify Risk of Morbidity from Sepsis in Pregnancy. Obstetric Anesthesia Digest 2018; 38 (1).
31. Bowyer L., Robinson H.L., Barrett H., Crozier T.M., Giles M., Idel I., et al. SOMANZ guidelines for the investigation and management sepsis in pregnancy. Aust. N. Z. J. Obstet. Gynaecol. 2017;57:540–51.
32. World Health Organization. Guideline: Managing Possible Serious Bacterial Infection in Young Infants When Referral is not Feasible. 2015. (Retrieved August 18, 2020 from https://apps.who.int/iris/bitstream/handle/10665/181426/9789241509268_eng.pdf)
33. Pasha O, McClure EM, Saleem S, Tikmani SS, Lokangaka A, Tshefu A, et al. A prospective cause of death classification system for maternal deaths in low and middle-income countries: results from the Global Network Maternal Newborn Health Registry. BJOG. 2018;125(9):1137-43.
34. Garces AL, McClure EM, Pérez W, Hambidge KM, Krebs NF, Figueroa L, et al. The Global Network Neonatal Cause of Death algorithm for low-resource settings. Acta Paediatr. 2017;106(6):904-11.
35. Goudar SS, Goco N, Somannavar MS, Kavi A, Vernekar SS, Tshefu A, et al. Institutional deliveries and stillbirth and neonatal mortality in the Global Network's Maternal and Newborn Health Registry. Reprod Health. 2020 Dec 17;17(Suppl 3):179.
36. Hoffman MK, Goudar SS, Kodkany, B. S., Goco, N., Koso-Thomas, M., Miodovnik, M., et al. A description of the methods of the aspirin supplementation for pregnancy indicated risk reduction in nulliparas (ASPIRIN) study. BMC Pregnancy and Childbirth 2017; 17(1), 135.
37. Roca A, O. C., Bojang A, Camara B, Kampmann B, Bailey R, Demba A, Bottomley C, D'Alessandro U. (2016). Oral azithromycin given during labour decreases bacterial carriage in the mothers and their offspring: a double-blind randomized trial. *Clin Microbiol Infect. , 22*(6), e1-9.
38. Roberts, S., Maccato, M., Faro, S., & Pinell, P. (1993). The microbiology of post-cesarean wound morbidity. *Obstetrics and Gynecology, 81*(3), 383-386.
39. Rosene, K., Eschenbach, D. A., Tompkins, L. S., Kenny, G. E., & Watkins, H. (1986). Polymicrobial early postpartum endometritis with facultative and anaerobic bacteria, genital mycoplasmas, and Chlamydia trachomatis: treatment with piperacillin or cefoxitin. *Journal of Infectious Diseases, 153*(6), 1028-1037.
40. Andrews, W. W., Shah, S. R., Goldenberg, R. L., Cliver, S. P., Hauth, J. C., & Cassell, G. H. (1995). Association of post-cesarean delivery endometritis with colonization of the chorioamnion by Ureaplasma urealyticum. *Obstetrics and Gynecology, 85*(4), 509-514. https://doi.org/10.1016/0029-7844(94)00436-H
41. Keski-Nisula, L., Kirkinen, P., Katila, M. L., Ollikainen, M., Suonio, S., & Saarikoski, S. (1997). Amniotic fluid U. urealyticum colonization: significance for maternal peripartal infections at term. *American Journal of Perinatology, 14*(3), 151-156.

For peer review only - http://bmjopen.bmj.com/site/about/guidelines.xhtml

ACKNOWLEDGEMENTS

The authors would like to acknowledge the A-PLUS research teams for their many contributions to this work: *Kinshasa School of Public Health, Kinshasa, Democratic Republic of Congo*: Michel Kalonji and Miyalu Junior; *University of North Carolina, Chapel Hill, North Carolina, United States*: Jackie Patterson; *University Teaching Hospital, Lusaka, Zambia:* Ernest Banda, Mwansa Chimfwembe, Ruth Nakazwe, and Abigail Mwapule; *Instituto de Nutrición de Centroamérica y Panamá, Guatemala City, Guatemala*: Maynor Manrique; *University of Colorado School of Medicine, Denver, CO*, USA: Jamie Westcott; *International Centre for Diarrhoeal Disease Research, Dhaka, Bangladesh*: Qazi Sadeq-ur-Rahman, Amita Farzana, Farhana Jahan, Zarin Tasnim Maliha, and Lolit Singh; *University of Virginia, Charlottesville, Virginia, United States:* Chris Chisolm; *KLE Academy Higher Education and Research, J N Medical College Belagavi, Karnataka, India:* Sheetal Harakuni, Manjunath Somannavar, and Kadappa Beniwadi; *Thomas Jefferson University, Philadelphia, PA, USA:* Frances Jaeger; *Aga Khan University, Karachi, Pakistan:* Zaheer Habib, Imran Ahmed, Farnaz Naqvi, Naija Ghanchi, and Saleem Jessani; *Lata Medical Research Foundation, Nagpur, India:* Vaishali Khedikar, Savita Bhargav, Samreen Sadaf, and Chaitali Gedam; Moi University School of Medicine, Eldoret, Kenya; Milsort Kemboi, Anderson Misati, and Kevin Otieno; *Indiana School of Medicine, University of Indiana, Indianapolis, IN, USA*: Sheri Bucher; *RTI International, Research Triangle Park, North Carolina, United States:* Anna Aceituno, Kay Jackson, Jean Kim, Janet Moore, Suchita Parepalli, Marissa Trotta, and Alexis Williams.

Bill & Melinda Gates Foundation– Laura Lamberti

AUTHOR'S CONTRIBUTIONS

ATita and WAC conceived of the study and developed the protocol, with input from EMM, TLN, JHF, MKT, and PLH. TLN developed the statistical analyses plan; JHF wrote the first draft of the manuscript and subsequent revisions, with critical feedback from WAC, ATita, EMM, MKT, and TLN. EC, MMwenechanya, TM, AL, ATshefu, GL, PLH, AP, PD, KK, SSG, AK, MMetgud, SS, SST, FE, PN, AS, LF, MMazariegos, SMB, RH, MSS, RLG, MB, CLB, EAL, OAE, NFK, RJD, and WAP contributed to the refinement and finalization of the study protocol and trial implementation. All authors contributed to the preparation of this manuscript and have reviewed and approved the final version.

FUNDING STATEMENT

The A-PLUS study is supported through institutional grants from the *Eunice Kennedy Shriver* National Institute of Child Health and Human Development (NICHD) [RTI International (U01 HD040636), University of North Carolina at Chapel Hill (U10 HD076465), University of Alabama at Birmingham (U10 HD078437), University of Colorado (U10 HD076474), Thomas Jefferson University (U10 HD076457), Columbia University (U10 HD078438), Boston University (U10 HD078439), Indiana University (U10 HD076461)] and a grant from the Foundation for the National Institutes of Health [MCCL19APT] through the Maternal, Newborn & Child Health Discovery & Tools initiative of the Bill & Melinda Gates Foundation (BMGF) [INV-008973]. The views expressed in this manuscript are those of the authors and do not necessarily represent the views of the NICHD; the National Institutes of Health; or the U.S. Department of Health and Human Services or the BMGF.

COMPETING INTERESTS STATEMENT

The authors declare that they have no competing interests.

For peer review only - http://bmjopen.bmj.com/site/about/guidelines.xhtml

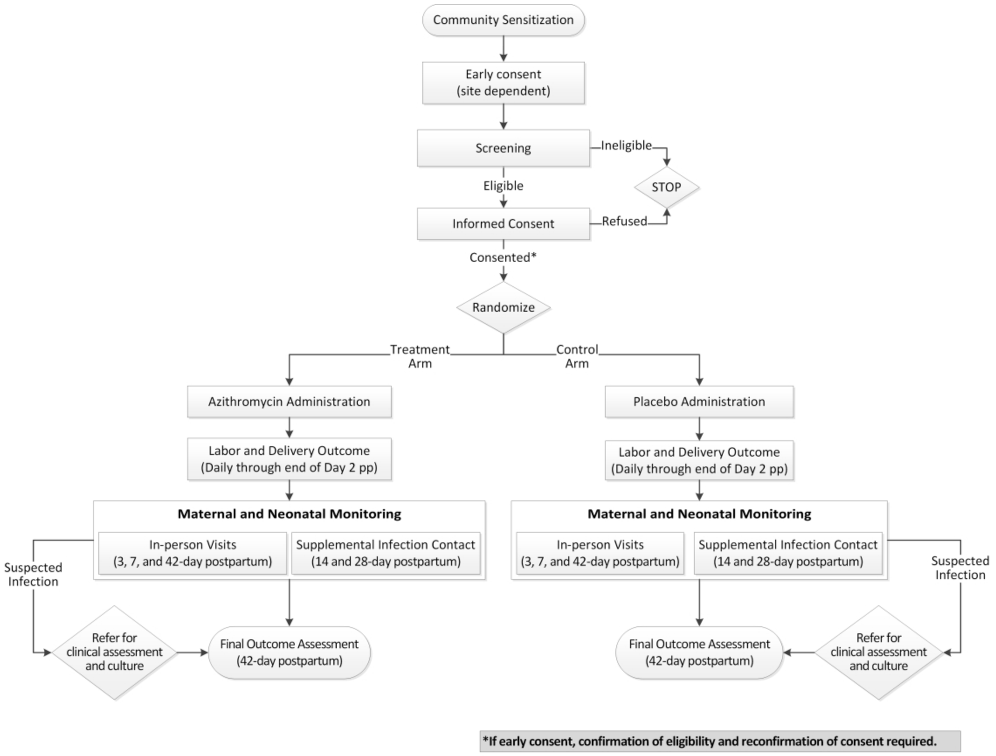

Figure 1. Study Flowchart

772x580mm (118 x 118 DPI)

Prevention of maternal and neonatal death/infections with a single oral dose of azithromycin in women in labor (in low- and middle-income countries): A randomized controlled trial

ClinicalTrials.gov number: NCT03871491

Lead Study Investigator(s): Alan T.N. Tita, MD, PhD, Center for Women's Reproductive Health and Maternal-Fetal Medicine Division, UAB
Wally Carlo, MD, Neonatology Division, UAB
Elwyn Chomba, MBChB, DCH, MRCP, University Teaching Hospital

Subcommittee Members: Marion Koso-Thomas, MD, NICHD
Tracy Nolen, DrPh, RTI International
Elizabeth McClure, PhD, RTI International
Jennifer J. Hemingway-Foday, MPH, RTI International

Version Number: Version 1.6

Version Date: July 15, 2022

Funding: Eunice Kennedy Shriver National Institute of Child Health and Human Development and The Bill & Melinda Gates Foundation

For peer review only

For peer review only - http://bmjopen.bmj.com/site/about/guidelines.xhtml

Version Tracking

Version	Date	Authors	Comments
1.0	Sep 27, 2018	A-PLUS WG	This version will be used for IRB/ERC submissions.
1.1	Dec 19, 2018	A-PLUS WG	Internal version to document and review proposed revisions from GN investigators.
1.2	Dec 20, 2018	A-PLUS WG and GN Investigators	At request of GN investigators, revisions made to the following sections: Inclusion and exclusion criteria (Section 2.2); Consent procedures (Section 2.4); Description of data forms (Section 4.1); Sample informed consent form (Appendix 2); and in-text references.<br>**This version will be used for IRB/ERC submissions after December 20, 2018.**
1.3	January 24, 2020	A-PLUS WG	Revisions made based on feedback from pilot study and requests from GN investigators. The following sections have been revised:<br>• Section 1 (Statement of Problem), including 1.2 and 1.3 (Primary and Secondary Specific Aims) and 1.5 (Rationale/Justification)<br>• Section 2 (Methods), including 2.1 (Inclusion Criteria); 2.4.7 (Monitoring after Discharge); 2.5 (Primary Outcomes); 2.6 (Other Maternal Outcomes); 2.7 (Other Neonatal Outcomes) and 2.8 (Safety Monitoring)<br>• Section 3 (Analytical Plan)<br>• Section 4.1 (Data Forms)<br>• Section 5 (References)<br>• Figure 1. Flowchart of Study Activities<br>• Appendix 2. Sample Informed Consent<br>• Appendix 3. Schedule of Study Procedures
1.4	January 29, 2020	A-PLUS WG	Additional revisions made to:<br>• Section 1.3 (Secondary Specific Aims)<br>• Section 2.5 (Primary outcomes)<br>• Section 2 (Methods), including 2.6 (Other Maternal Outcomes) and 2.7 (Other Neonatal Outcomes)<br>• Section 3 (Analytical Plan, including 1.2 (Secondary Analysis-Women at High Risk for Infection Cohort) 3.2.1 (Sample Size for Primary Outcome) and 3.2.2 (Sample Size for High-risk Women)<br>**This version will be submitted to IRBs/ERCs at all GN sites.**
1.5	February 09, 2021	A-PLUS WG	Updates made based on recommendations from the Data Monitoring Committee:<br>• In Section 2.5 (Primary Outcome), removed urinary tract infection, omphalitis, eye infection, and skin infection from neonatal sepsis definition, removed fast breathing from the pSBI definition, and changed the pSBI fever criteria to 38 C/100.4 F.<br>• In Section 2.7 (Other Neonatal Outcomes), added urinary tract infection, omphalitis, and fast breathing to the "other neonatal infection" outcome.<br>Other updates:<br>• In Section 3.5.1 (Reporting Adverse Events), made minor text changes to align with revised AE reporting procedures. [Refer to A-PLUS Technical Memo #2]<br>• In Section 3.5.4 (Interim/Adverse Event Monitoring Plan), made minor text revisions to clarify that the interim analysis will be conducted in both the all-comers and high-risk cohort. This revision was made at the request of the Boston University IRB.<br>• In list of Azithromycin Investigators, updated SFI for GN06-Guatemala to Manolo Mazariegos.<br>**This version will be submitted to IRBs/ERCs at all GN sites.**
1.6	12 July, 2022	A-PLUS WG	High-risk definition updated to "Prolonged labor [≥18 hours] and/or prolonged membrane rupture [≥8 hours]". The following sections have been updated to reflect this change:<br>• Abstract-*Secondary Outcomes*<br>• Section 1.3 (Secondary Specific Aims)<br>• Section 1.5 (Rationale/Justification)<br>• Section 1.6.1 (The Gambian and Cameroon Trials) *[Note: new reference added to this section, with full citation in reference list; as a result, all subsequent references have been renumbered]*<br>• Section 2.2.2 (Exclusion Criteria)<br>• Section 3 (Analytical Plan)<br>• Section 3.1.2 (Secondary Analysis-Women at High Risk for Infection Cohort)<br>• Section 3.2.2 (Sample Size for High-risk Women)<br>• Appendix 2 (Sample Consent)<br>Other minor updates were made to the list of "Azithromycin Investigators" to reflect staffing changes since protocol V. 1.5 was released.<br>**This version will be submitted to IRBs/ERCs at all GN sites.**

For peer review only - http://bmjopen.bmj.com/site/about/guidelines.xhtml

Table of Contents

Azithromycin Investigators 6

ACRONYMS 9

Abstract 10

1 Statement of Problem 11

1.1 Primary Hypotheses 11

1.2 Primary Specific Aims 11

1.3 Secondary Specific Aims 11

1.4 Background 12

1.4.1 Prevention of Maternal and Neonatal Deaths from Infections 12

1.4.2 Risks for Maternal and Neonatal Infections 12

1.4.3 Intrapartum Azithromycin to Prevent Maternal and Neonatal Infection 13

1.5 Rationale/Justification 13

1.6 Previous Studies 14

1.6.1 The Gambian and Cameroon Trials 14

1.6.2 The US Trial and Cost Analysis 14

1.6.3 Other Important Considerations 15

2 Methods 16

2.1 Study Design 17

2.2 Study Population/Location 17

2.2.1 Inclusion Criteria 17

2.2.2 Exclusion Criteria 17

2.3 Study Intervention and Comparison 18

2.4 Detailed Study Procedures 18

2.4.1 Community Sensitization 18

2.4.2 Screening 18

For peer review only - http://bmjopen.bmj.com/site/about/guidelines.xhtml

For peer review only

2.4.3 Consent 18
2.4.4 Masking 19
2.4.5 Randomization Procedures 20
2.4.6 Monitoring before Discharge 20
2.4.7 Monitoring after Discharge 20
2.4.8 Sequence of Study Activities 21
2.5 Primary Outcomes 22
2.6 Other Maternal Outcomes 22
2.7 Other Neonatal Outcomes 23
2.8 Safety Monitoring 23
2.9 Site Preparation 24
2.10 Potential Risks and Benefits to Participants 24
3 Analytical plan 25
3.1 Statistical Analysis Plan 25
3.1.1 Primary Analyses 25
3.1.2 Secondary Analyses – Women at High Risk for Infection Cohort 26
3.1.3 Secondary Analyses – Other Secondary Outcomes 26
3.2 Sample Size 27
3.2.1 Sample Size for Primary Outcome 27
3.2.2 Sample Size for High-Risk Women 28
3.3 Available Population 29
3.4 Projected Recruitment Time 29
3.5 Study Monitoring Plan 29
3.5.1 Reporting Serious Adverse Events 29
3.5.2 Method and Timing for Reporting Serious Adverse Events 30
3.5.3 Data Monitoring Plan and Stopping Rules 30
3.5.4 Interim/Adverse Event Monitoring Plan 31
3.5.5 Risks/Benefits 32
3.6 Quality Control 32

For peer review only - http://bmjopen.bmj.com/site/about/guidelines.xhtml

3.6.1 Training 32
3.6.2 Study Monitoring 32
3.6.3 Drug Quality Assurance and Monitoring 32
3.6.4 Plan for Sustaining Intervention 33
4 Data Management Procedures 33
4.1 Data Forms 33
5 References 35
Appendix 1. Description of Participating Global Network Sites 38
Appendix 2. Sample Informed Consent 40
Appendix 3. Schedule of Study Procedures 44

For peer review only

For peer review only - http://bmjopen.bmj.com/site/about/guidelines.xhtml

AZITHROMYCIN INVESTIGATORS

Central Team: Global Network Site 03 (Zambia)

Waldemar A. Carlo, MD
Principal Investigator
University of Alabama at Birmingham
Birmingham, Alabama
wcarlo@peds.uab.edu

Alan T.N. Tita, MD, PhD
Azithromycin Protocol Lead Investigator
University of Alabama at Birmingham
Birmingham, Alabama
atita@uabmc.edu

Elwyn Chomba, MBChB, DCH, MRCP
Senior Foreign Investigator
University Teaching Hospital
Lusaka, Zambia
chombaelwyn@gmail.com

Site Investigators

Global Network Site 02 (Democratic Republic of Congo)

Carl Bose, M.D.
Principal Investigator
University of North Carolina School of Medicine
Chapel Hill, North Carolina
cbose@med.unc.edu

Antoinette Tshefu, MD, PhD, MPH
Senior Foreign Investigator
Kinshasa School of Public Health
antotshe@yahoo.com

Global Network Site 03 (Zambia)

Wally Carlo, MD
Principal Investigator
University of Alabama at Birmingham
Birmingham, Alabama
Wcarlo@PEDS.UAB.EDU

Elwyn Chomba, MBChB, DCH, MRCP
Senior Foreign Investigator
University Teaching Hospital
Lusaka, Zambia
chombaelwyn@gmail.com

Global Network Site 06 (Guatemala)

Nancy Krebs, MD
Principal Investigator
UCHSC
Denver, Colorado
Nancy.krebs@ucdenver.edu

For peer review only

For peer review only - http://bmjopen.bmj.com/site/about/guidelines.xhtml

Manolo Mazariegos, MD
Senior Foreign Investigator
INCAP
Guatemala City, Guatemala
mmazariegos@incap.int

Global Network Site 07 (Bangladesh)

William Petri, MD
Principal Investigator
University of Virginia
Charlottesville, Virginia
wap3g@virginia.edu

Rashidul Haque, MD
Senior Foreign Investigator
ICDDR,B
Dhaka, Bangladesh
rhaque@icddrb.org

Global Network Site 08 (Belagavi, India)

Richard Derman, MD, MPH
Principal Investigator
Thomas Jefferson University
Philadelphia, PA
richard.derman@jefferson.edu

Shivaprasad S. Goudar MD, MHPE
Senior Foreign Investigator
KLE University's J N Medical College
Belgaum, India
sgoudar@jnmc.edu

Global Network Site 09 (Pakistan)

Robert L. Goldenberg, MD
Principal Investigator
Columbia University
New York, New York
rlg88@columbia.edu

Sarah Saleem, MD
Senior Foreign Investigator
Aga Khan University
Karachi, Pakistan
sarah.saleem@aku.edu

Global Network Site 11 (Nagpur, India)

Patricia L. Hibberd, MD, PhD
Principal Investigator
Boston University
Boston, Massachusetts
plh0@bu.edu

Archana Patel, MD, DNB, MSCE, PhD
Senior Foreign Investigator

For peer review only

Lata Medical Research Foundation
Nagpur, India
Dr_apatel@yahoo.com

Global Network Site 12 (Kenya)

Ed Liechty, MD
Principal Investigator
Indiana University School of Medicine
Indianapolis, Indiana
eliecht@iupui.edu

Fabian Esamai, MBChB, MMed, MPH, PhD
Senior Foreign Investigator
Moi University School of Medicine
Eldoret, Kenya
fesamai2007@gmail.com

National Institute of Child Health and Human Development (NICHD)

Marion Koso-Thomas, MD, MPH
Medical Officer, Global Network for Women's and Children's Health
Eunice Kennedy Shriver National Institute of Child Health and Human Development (NICHD)
kosomari@mail.nih.gov

RTI International

Elizabeth McClure, PhD
Principal Investigator, Data Coordinating Center
RTI International, Durham, NC
mcclure@rti.org

Tracy Nolen, DrPh
Senior Statistician, Data Coordinating Center
tnolen@rti.org

Jennifer J. Hemingway-Foday, MPH, MSW
Protocol Manager for Azithromycin Protocol, Data Coordinating Center
hemingway@rti.org

For peer review only

For peer review only - http://bmjopen.bmj.com/site/about/guidelines.xhtml

ACRONYMS

**AC**	All-comers
**ACOG**	American Congress of Obstetricians and Gynecologists
**ANC**	Antenatal Care
**BP**	Blood pressure
**DCC**	Data Coordinating Center
**DMC**	Data monitoring committee
**DMS**	Data Management System
**DRC**	Democratic Republic of Congo
**ERC**	Ethical review Committee
**FIGO**	International Federation of Gynecology and Obstetrics
**FDA**	Food and Drug Administration
**GA**	Gestational age
**GI**	Gastrointestinal
**GN**	Global Network for Women's and Children's Health Research
**HR**	High-risk
**IRB**	Institutional Review Board
**JAMA**	Journal of the American Medical Association
**ITT**	Intention to treat
**LIC**	Low-income country
**LMIC**	Low- and middle-income countries
**LMP**	Last menstrual period
**MNH**	Maternal and Newborn Health
**NEJM**	New England Journal of Medicine
**NICHD**	Eunice Kennedy Shriver National Institute of Child Health and Human Development
**NIH**	National Institutes of Health
**NNT**	Number needed to treat
**OHRP**	U.S. Office of Research Protections
**PI**	Principal Investigator
**pSBI**	possible serious bacterial infection
**RCT**	Randomized controlled trial
**RTI**	Research Triangle Institute International
**SAE**	Serious adverse event
**SC**	Steering Committee
**SFI**	Senior Foreign Investigator
**SMFM**	Society for Maternal-Fetal Medicine
**UAB**	University of Alabama at Birmingham
**WHO**	World Health Organization

For peer review only

For peer review only - http://bmjopen.bmj.com/site/about/guidelines.xhtml

ABSTRACT

Background: Maternal and neonatal infections are among the most frequent causes of maternal and neonatal deaths, and current antibiotic strategies have not been effective in preventing many of these deaths. Recently, a randomized clinical trial conducted in a single site in The Gambia showed that treatment with oral dose of 2 g azithromycin vs. placebo for all women in labor reduced certain maternal and neonatal infections. However, it is unknown if this therapy reduces maternal and neonatal sepsis and mortality.

Hypotheses: The trial includes two primary hypotheses, a maternal hypothesis and a neonatal hypothesis. First, a single, prophylactic intrapartum oral dose of 2 g azithromycin given to women in labor will reduce maternal death or sepsis. Second, a single, prophylactic intrapartum oral dose of 2 g azithromycin given to women in labor will reduce intrapartum/neonatal death or sepsis.

Study Design Type: Randomized, placebo-controlled, parallel multicenter clinical trial. Women in labor will be randomized with one-to-one ratio to intervention/placebo.

Population: Pregnant women in labor at ≥28 weeks gestational age with a live fetus pregnancy who plan to deliver vaginally in a facility. Women with evidence of chorioamnionitis or other infection requiring antibiotic therapy (not prophylaxis) at time of eligibility, allergy to azithromycin, use of azithromycin, erythromycin or other macrolide within 3 days of enrollment, known arrhythmia or cardiomyopathy, or plan for cesarean section delivery prior to enrollment will be excluded.

Intervention: A single, prophylactic intrapartum oral dose of 2 g azithromycin.

Comparison: A single intrapartum oral dose of an identical appearing placebo

Outcomes:
Primary outcomes: 1) Incidence of maternal death or sepsis and 2) incidence of intrapartum/neonatal death or sepsis.

Secondary outcomes: Individual components of the primary outcomes (maternal death, maternal sepsis, intrapartum/neonatal death, neonatal sepsis), neonatal death/sepsis, neonatal deaths due to sepsis, and all-cause neonatal death; the primary maternal and neonatal outcome in a high-risk for infection population; specific maternal infections; use of subsequent maternal antibiotic therapy; pyloric stenosis; health care resource utilization; culture-positive infections and resistance; in an ancillary surveillance study, incidence of antimicrobial resistance and microbiome diversity.

For peer review only - http://bmjopen.bmj.com/site/about/guidelines.xhtml

1 Statement of Problem

1.1 Primary Hypotheses

This study will have two primary hypotheses, one with a maternal focus and one with a neonatal focus. First, a single, prophylactic intrapartum oral dose of 2 g azithromycin given to women in labor in low and middle-income settings will reduce maternal death or sepsis. Second, a single, prophylactic intrapartum oral dose of 2 g azithromycin given to women in labor in low and middle-income settings will reduce intrapartum/neonatal death or sepsis.

1.2 Primary Specific Aims

To test the effectiveness of a single dose of prophylactic intrapartum azithromycin compared to placebo in reducing the risk of the composite outcome of maternal death or sepsis. To separately test the effectiveness of a single oral dose of intrapartum azithromycin prophylaxis (2 g) compared to placebo in reducing the risk of the composite outcome of intrapartum/neonatal death or sepsis. Both groups will receive the routine or usual care provided at the facility during and after labor.

1.3 Secondary Specific Aims

The single dose of intrapartum azithromycin prophylaxis (2 g) will be compared to placebo to accomplish the following secondary aims:

- Main Secondary Aim for Women at High-Risk for Infection Cohort:
  - a. To evaluate whether the risk of maternal death or sepsis differs among laboring women with and without high-risk for infection (high risk is defined as women with prolonged labor [≥18 hours] and/or prolonged membrane rupture [≥8 hours] at time of randomization).
- Other secondary specific Aims:
  - b. To evaluate the effectiveness in reducing the risk of individual components of the primary composite endpoints (i.e., maternal death, maternal sepsis, intrapartum/neonatal death, neonatal sepsis including specifically all-cause neonatal deaths and neonatal deaths due to sepsis).
  - c. To evaluate the effectiveness on the risk of intrapartum/neonatal death or sepsis in infants of laboring women at high-risk for infection because of prolonged labor (≥18 hours) and/or prolonged membrane rupture (≥8 hours).
  - d. To evaluate the effectiveness on the risk of maternal infections including clinical chorioamnionitis, endometritis, wound infections (perineal or Cesarean), abdominal or pelvic abscess, mastitis/breast abscess or infection, pyelonephritis, pneumonia and other bacterial infections in all laboring women as well as in those at high-risk for infection.
  - e. To evaluate the effectiveness in reducing the use of subsequent maternal antibiotic therapy from randomization to 42 days postpartum for any reason in all laboring women as well as in those at high-risk for infection.
  - f. To compare the use of health care resources. Use of health care resources will be measured in terms of maternal and neonatal duration of hospital stay, unscheduled visits, readmission, and admission to special care units/intensive care units in all laboring women and newborns as well as in those at high-risk for infection.
  - g. To assess maternal GI symptoms (e.g., nausea, vomiting, and diarrhea) and other reported side effects, as well as infant pyloric stenosis and type of culture positive infections

For peer review only - http://bmjopen.bmj.com/site/about/guidelines.xhtml

(antimicrobial resistance and maternal and infant microbiome diversity are compared in an ancillary study).

h. To determine whether the effect on each of the primary outcomes differs by region (Africa, Latin America or Asia), any other antibiotic use during labor, and mode of delivery (cesarean or vaginal). Note that antibiotic use will not include use to treat any infections diagnosed after randomization as those will be outcomes.

1.4 Background

Maternal and neonatal infections are among the most frequent causes of maternal and neonatal death. Maternal infection during pregnancy and the puerperium account for approximately 10% of the global burden of maternal deaths. This places maternal infection among the top five causes of maternal mortality worldwide.[1] Neonatal infection is the third most common cause of neonatal mortality and accounts for about 16% of neonatal mortality worldwide.[2] Furthermore, maternal and neonatal deaths from infections are not decreasing compared with deaths from other frequent causes of mortality.[1,2] There is a need for innovative simple effective interventions that can be scaled up to reduce the burden of both maternal and neonatal mortality due to infections.

1.4.1 Prevention of Maternal and Neonatal Deaths from Infections

Current strategies to prevent maternal and neonatal deaths from infections are insufficient. Current approaches to prevention, identification, and treatment of neonatal sepsis have had limited impact. According to the WHO, maternal deaths from infection have remained unchanged or increased in some instances, whereas deaths from other causes have reduced. The evidence backing current WHO guidelines for prevention and treatment of peripartum infections is generally graded as low or very low quality.[1] Efforts for early identification and treatment of neonatal infection are important but neonatal deaths due to infection continue to be very prevalent. Recent studies of alternative antibiotic regimens that do not require a full course of intravenous antimicrobial therapy have shown comparable effectiveness but have not reduced deaths from infections.[3-6] Among research priorities, a WHO guideline panel identified the evaluation of the role of routine prophylactic antibiotics in women who undergo a normal vaginal birth as well as in those at high risk for infection due to prolonged labor or membrane rupture.[1]

1.4.2 Risks for Maternal and Neonatal Infections

Maternal and neonatal infections by maternal risk including cesarean delivery. While the role of antibiotic prophylaxis for cesarean delivery is well-established, in many low-resource settings, many maternal infections occur after vaginal deliveries with high-risk for infections. Cesarean delivery especially after labor or membrane rupture is the strongest risk factor for maternal peripartum infection including endometritis, wound infection and sepsis (increasing risk by up to 5 to 10 times compared to vaginal delivery).[7] The fraction of maternal infection attributable to cesarean delivery is rather low because of the low cesarean delivery rate of 5-10% or lower, especially in the Africa region, compared with 20-30% or higher in the US and many high income countries.[8] Therefore, strategies that address maternal peripartum infection and sepsis in the developing world should focus as well on identifying and preventing (as well as treating) infection in women who have a vaginal birth, particularly those who are at high-risk. There is an increased risk of infection in women who undergo prolonged labor ≥18 hours (at least 2 fold) or membrane rupture ≥6 hours (at least 2-3 fold) compared to women who do not experience these risk factors.[1] These risk factors identify a large group of women who are at the highest

For peer review only - http://bmjopen.bmj.com/site/about/guidelines.xhtml

risk for maternal peripartum infections (including chorioamnionitis, endometritis, perineal wound infection and post-cesarean surgical site infections) and sepsis after a vaginal or cesarean delivery and also place newborns at increased risk for sepsis. However, even though 80-90% of pregnancies in labor are not at this highest-risk for infection depending on criteria used, they may account for about 50% of maternal and neonatal infections in LMICs. Thus, testing antibiotic strategies during labor in LMICs should include both the high-risk and lower risk women as highlighted in research priorities from the WHO guideline panel.[1]

Antibiotic Prophylaxis for Women who deliver by Cesarean section. The appropriate use of antibiotic prophylaxis for cesarean delivery and antiseptic agents are among the most effective preventive interventions as highlighted by the WHO guidelines for maternal peripartum infections.[1] Antibiotic prophylaxis, preferably prior to incision, in particular, among several strategies, effectively reduces the risk of infection and the associated high health care and personal costs.[1,9-11] However, the prevalence of cesarean sections is low, in many low-resource settings, particularly in sub-Saharan Africa, and thus, do not account for the large burden of the maternal and neonatal infections worldwide.

1.4.3 Intrapartum Azithromycin to Prevent Maternal and Neonatal Infection

Azithromycin: a novel approach to maternal and neonatal infections. A novel approach to maternal and neonatal infection is to target organisms that may be very frequent pathogens but that historically have not been the target of antimicrobial treatment.[12] A multicenter randomized clinical trial (RCT) of azithromycin prophylaxis added to the standard prophylactic regimen (cephalosporin) in women who underwent cesarean delivery following labor or membrane rupture for at least 4 hours in the US showed that maternal infection was reduced by about 50%.[13] A single center RCT in a low-income country (LIC) setting suggested that azithromycin prophylaxis may improve maternal and neonatal outcomes. In an RCT in The Gambia that included all women in labor, treatment with 2 g of azithromycin vs. placebo before delivery reduced maternal and neonatal infections.[14] Therefore, we propose to evaluate the effectiveness of a single oral dose of azithromycin as an intrapartum prophylactic agent for maternal and neonatal infection and death. We will also monitor the potential side effects of this intervention.

1.5 Rationale/Justification

Maternal infection and sepsis is a priority to reduce maternal and neonatal deaths. Compared to postpartum hemorrhage and preeclampsia/eclampsia, maternal infection has received less attention as a major cause of maternal death; proportionally it accounts for increasing deaths.[1] The WHO and other global health authorities identified maternal infection/sepsis as a priority problem to reduce maternal deaths. In addition, maternal infection significantly increases the risk of neonatal sepsis which is one of the leading causes of neonatal death in LICs.[15] Most recently, a NEJM perspective article highlighted the WHO resolution issuing a clarion call recognizing "Sepsis" as a global health priority.[16] Drawing from our findings on azithromycin prophylaxis for cesarean delivery in the US[13] and data from another preliminary trial in The Gambia, Africa,[14] we propose to evaluate the role of a single oral dose of azithromycin (plus usual care) to prevent maternal death or peripartum sepsis and intrapartum/neonatal death or sepsis in laboring women as well as the targeted sub-population of those at the highest risk for infection because they have prolonged labor (≥18 hours) and/or prolonged membrane rupture (≥8 hours).

Azithromycin is available as a generic agent with easy storage requirements. It has a bimodal half-life of up to 70 hours in the non-pregnant population. Although its pharmacokinetic characteristics are not as

For peer review only - http://bmjopen.bmj.com/site/about/guidelines.xhtml

well studied in the pregnant population, it is commonly used during pregnancy for treatment of chlamydia and other infections. Azithromycin covers a broad spectrum of bacteria (including gram-positive cocci, genital ureaplasmas and mycoplasmas, and certain gram-negative bacilli and anaerobes) that are associated with maternal infections which are often polymicrobial (chorioamnionitis, endometritis, and perineal/cesarean wound infection) and sepsis. In addition to the aforementioned organisms which may play a role in neonatal infection, azithromycin also has activity against Group B streptococcus which is a major cause of neonatal sepsis in developed countries and may be implicated in LICs and low-resources settings as well. Therefore, a successful prophylaxis intervention is likely to reduce infections and death and may also reduce health care costs and need for prolonged antibiotic therapy to treat infections which may be associated with resistance. Indeed, the recent WHO Guidelines on peripartum infection articulated the following among research priorities: what are the benefits of initiating prophylactic antibiotics among women undergoing uncomplicated vaginal birth and among those at high risk such as after prolonged rupture of membranes?[1] A JHPIEGO consultative meeting on enhancing the focus on maternal infections suggested that "Attention to identification and prompt management of prolonged labor and prolonged rupture of membranes is critical to reduce disease and death due to maternal sepsis."[15,16,17]

1.6 Previous Studies

1.6.1 The Gambian and Cameroon Trials

Data from a single center trial in a LIC setting suggest the potential for azithromycin prophylaxis to improve maternal and neonatal outcomes. Among 829 Gambian mothers (randomized to 2 g of azithromycin vs. placebo before delivery) and their 830 newborns, maternal infections were lower in the azithromycin group (3.6% vs 9.2%; relative risk [RR], 0.40; 95% confidence interval [CI], 0.22-0.71; P = 0.002).[14] Among newborns, the overall prevalence of infections was also lower in the azithromycin group (18.1% vs 23.8%; RR, 0.76; 95% CI, 0.58-0.99; P = 0.052).[14] Maternal and neonatal carriage of infectious organisms was lower in the azithromycin group.[18] A test of concept trial focusing on high-risk women in Cameroon (labor ≥18 hours or membrane rupture ≥8 hours at randomization was underpowered by suggested lower maternal infection in women who received azithromycin.[19]

1.6.2 The US Trial and Cost Analysis

In a multicenter US RCT, it was demonstrated a further 50% reduction in the risk of maternal peripartum infection by adding a single 500 mg intravenous dose of azithromycin to the standard prophylactic regimen (a single intravenous dose of cefazolin 1-2 g or ampicillin) in the highest-risk group of women who undergo cesarean delivery following labor or membrane rupture for at least 4 hours.[13] These results were observed despite universal use of other antibiotics in both arms of the trial. Specifically, the 95% without penicillin allergy had a cephalosporin (mainly cefazolin) for usual cesarean prophylaxis; the remainder that gentamicin and clindamycin. In addition, 25-30% received a penicillin for GBS prophylaxis. Infection was significantly lower in the azithromycin group compared to the placebo group; 6.1% vs. 12%; RR =0.51 (95 % CI 0.38 to 0.68); p<0.001. Specifically, adjunctive azithromycin use was associated with significant reductions in the risks of endometritis (3.8% vs. 6.1%, RR=0.62, 95% CI: 0.42-0.92; p=0.02) and wound infections (2.4% vs. 6.6%, RR=0.35, 95% CI: 0.22-0.56; p<0.001). The number needed to treat (NNT) to prevent one infection was 17 for any infection, 43 for endometritis and 24 for wound infection. Other maternal outcomes including need for readmission or unscheduled visits for any reason or specifically for infection (decreased by up to 50%), serious adverse events, postpartum fever,

For peer review only - http://bmjopen.bmj.com/site/about/guidelines.xhtml

or subsequent treatment with antibiotics were also significantly less common with azithromycin prophylaxis. Short-term perinatal/infant outcomes including deaths, sepsis, and other serious neonatal morbidities were rare in this developed country population and did not differ between groups.[13] In a related cost-analysis report, it was estimated that use of adjunctive azithromycin saves approximately $360 for each use in unscheduled (high-risk) cesarean deliveries such as those studied in the RCT and $143 per use in scheduled or pre-labor cesarean delivery.[20] The results suggesting cost-savings were robust across wide ranges of baseline risk of infection and treatment effect size.[20]Thus, in the US alone, adjunctive azithromycin prophylaxis for cesarean delivery could lead to $350M in cost-savings/year due to avoided infection. These works built upon, and are supported by, over 20 years of research on maternal infections at UAB and elsewhere.[21-31] Azithromycin provides coverage against the most common pathogens identified in association with peripartum infections including genital mycoplasmas and ureaplasmas (when specific methods are utilized to identify them).

1.6.3 Other Important Considerations

A recurrent concern regarding the potential routine use of azithromycin in a large population is antibiotic resistance. Monitoring for characteristics suggestive of resistant infection will be important to incorporate in the protocol. However, a number of factors mitigate concerns about antibiotic resistance: (a) the design of the trial using a single prophylactic dose (as opposed to recurrent treatment doses) of antibiotic, (b) surveillance of maternal clinical cultures up to 6 weeks in the trial in cesareans in the US revealed that positive wound cultures overall and those positive for resistant organisms were significantly less frequent in the azithromycin group;[13] (c) if successful, prophylaxis will reduce the risk of infection and actually reduce the overall frequency of use of antibiotics to treat infection (a result in the US trial as stated above). It is estimated that women in the high-risk for infection group as defined will account for about 10-20% of laboring women. In principle, the use of single prophylactic dose (as opposed to multiple) minimizes the likelihood of antimicrobial resistance but samples will be taken to determine antimicrobial resistance.[32]

Azithromycin is currently recommended to treat or prevent several infections in pregnancy including gonorrhea (1 g po), chlamydia (1 g po), and Mycobacterium avium complex prophylaxis (600 mg twice/week or 1.2 g weekly po). Azithromycin is sometimes used for perioperative prophylaxis in patients at risk for endocarditis (500 mg po). Considering the success with a single dose of 2 g in the Gambian trial, our success with 500 mg IV for cesarean prophylaxis, and the 40% bioavailability of oral azithromycin, we propose to use 2 g po of azithromycin for the proposed intervention. The best approach to this evaluation in order to influence future uptake into clinical practice is an RCT. The primary maternal outcome will be maternal death or sepsis within 42 days after delivery. The primary neonatal outcome will be intrapartum/neonatal death or sepsis within 28 days after birth (defined by WHO criteria).[33]

Azithromycin is *pregnancy category B* – animal studies using maternally toxic doses showed no fetal harm. Limited studies suggest azithromycin is excreted in human milk in a sustained fashion.[34] There are no specific drug-drug interactions warranting dose adjustments when given with other medications. Elimination is by both hepatic and renal route, and no specific adjustments are mandated for patients with renal or hepatic insufficiency. The long elimination 1/2-life of 68 hours is due to extensive uptake and subsequent release of drugs from tissues. The only absolute contraindications are rare: known hypersensitivity reaction to azithromycin, erythromycin or other macrolide antibiotic or history of

For peer review only - http://bmjopen.bmj.com/site/about/guidelines.xhtml

cholestatic jaundice due to azithromycin. Potential adverse events include very rare (<1%) allergic hypersensitivity (mild and severe skin reactions – Stevens Johnson Syndrome and toxic epidermal necrolysis, angioedema and anaphylaxis) and clostridium difficile-associated diarrhea. With multi-day azithromycin therapy, 0.6% of patients discontinued azithromycin due to side effects. With single 1-2 gram doses, gastrointestinal symptoms (nausea 5-18%, diarrhea/loose stools 7-14%, abdominal pain 5-7%, vomiting 2-7%, and dyspepsia 1%) as well as vaginitis 2% and dizziness (1%) were the most commonly reported side effects. No other side effect occurred with a frequency greater than 1%.

Azithromycin has been associated with hypertrophic pyloric stenosis in some observational studies. In a retrospective study, oral azithromycin exposure during the first 14 days after birth was associated with an increased incidence of pyloric stenosis.[35] However, an increased risk of pyloric stenosis was not reported in infants following a single dose of azithromycin to women in labor in randomized trials in the US and Gambia.[13-14, 16] The much larger sample size of the proposed study affords the opportunity to further explore this question.

The FDA in 2013 issued an advisory regarding concerns about potential for rare life-threatening arrhythmias with azithromycin use particularly among those with preexisting cardiovascular risk. The information was based on an observational study of older, ill patients who received multiple courses of oral azithromycin over 5 days.[36] The findings are not applicable to our current study population for several reasons: the population is much younger, generally without cardiac co-morbidities, a single dose rather than cumulative doses of oral azithromycin over 5 days is being studied, additional studies of younger, healthy patients did not suggest an increased cardiovascular risk[37]; and patients with arrhythmia or known history of cardiomyopathy will be excluded. In addition, the prior study of 2013 women in the US did not suggest a cardiovascular safety signal, and the potential reduction in severe infection may exceed the excess risk of severe arrhythmia with a single dose of azithromycin.[13]

2 Methods

Pregnant women in labor at study health facilities who appear to be ≥28 weeks gestational age by best clinical estimate with a live fetus pregnancy will be screened for eligibility (see **Section 2.2**) by research staff starting at the time of admission and continuously during the hospitalization. Gestational age will be determined using the "best estimate" algorithm that is currently used in the GN MNH registry. Those eligible will be consented. Those consented will be randomized to receive a single dose of azithromycin 2 g or identical placebo to be given and directly observed during labor. Because of the ease of storage of azithromycin, sequentially prepared medications in identical packages following the randomization sequence will be available and dispensed by the study staff. The rest of the care will be provided according to the local standard of care. Women will be followed up until discharge and surveillance maintained (in-person), with visits at 3 days, 7 days, and 42 days after delivery as has generally been done in the Global Network to ascertain study outcomes.

We will apply the recently recommended "simple and actionable" WHO definition of sepsis in a 2017 statement endorsed by multiple international organizations. The definition of sepsis includes a suspicion of infection and the presence of organ dysfunction based on clinical findings.[15]

To further ensure more objective ascertainment of the sepsis outcome, we propose that the DCC will prospectively define a process for defining criteria for a masked centralized review and adjudication of all

For peer review only - http://bmjopen.bmj.com/site/about/guidelines.xhtml

cases of infection to ensure conformity with the proposed definition. This approach of adjudication is applied to validate infection in many trials including our recently completed ASPIRIN trial.[38] This approach to adjudication has some similarities to the near-miss classification for adjudication in women delivering in the Global Network sites that was done recently.[39]

2.1 Study Design

This study is a masked, placebo-controlled RCT. The investigational regimen is 2 g of azithromycin and the comparison arm is an identical placebo which is given orally. Both groups will also receive the standard of care during labor, delivery and postpartum per local standards.

2.2 Study Population/Location

Pregnant women laboring in health facilities of the eight Global Network sites/other health facilities will be eligible. The Global Network sites are described in **Appendix 1**. Health facilities may include any hospitals and health centers where women routinely deliver within the study sites.

2.2.1 Inclusion Criteria

- Pregnant women in labor ≥28 weeks GA (by best estimate) with a pregnancy with one or more live fetuses who plan to deliver vaginally in a facility.
- Admitted to health facility with clear plan for spontaneous or induced delivery.
- Live fetus must be confirmed via presence of a fetal heart rate prior to randomization.
- ≥18 years of age or minors 14-17 years of age in countries where married or pregnant minors (or their authorized representatives) are legally permitted to give consent.
- Have provided written informed consent [Note: written informed consent may be obtained during antenatal care, but verbal re-confirmation may be needed (per local regulations) at the time of randomization].

2.2.2 Exclusion Criteria

- Non-emancipated minors (as per local regulations)
- Evidence of chorioamnionitis or other infection requiring antibiotic therapy at time of eligibility (however, women given single prophylactic antibiotics with no plans to continue after delivery should not be excluded).
- Arrhythmia or known history of cardiomyopathy.
- Allergy to azithromycin or other macrolides that is self-reported or documented in the medical record.
- Any use of azithromycin, erythromycin, or other macrolide in the 3 days or less prior to randomization.
- Plan for cesarean delivery prior to randomization.
- Preterm labor undergoing management with no immediate plan to proceed to delivery.
- Advanced stage of labor (>6 cm or 10 cm cervical dilation per local standards) and pushing **or** too distressed to understand, confirm, or give informed consent **regardless** of cervical dilation.
- Are not capable of giving consent due to other health problems such as obstetric emergencies (for example, antepartum haemorrhage) or mental disorder.
- Any other medical conditions that may be considered a contraindication per the judgment of the site investigator.
- Previous randomization in the trial.

For peer review only - http://bmjopen.bmj.com/site/about/guidelines.xhtml

Sites may choose to obtain written consent during antenatal care from pregnant women of the age of consent who plan to deliver vaginally in a facility and have no known medical exclusions. In this case, study staff must fully assess and confirm eligibility at the time of randomization. It may also be necessary to verbally re-confirm consent at the time of randomization, if required by local regulations. If it is determined that the study needs to be extended to accrue the planned sample size for HR cohort, then women who deliver prior to meeting HR criteria for randomization will be excluded.

2.3 Study Intervention and Comparison

The study intervention is a single 2 g dose of directly observed oral azithromycin, to be administered as four 500 mg pills or tablets directly after randomization. By random allocation, participants will receive 2 g of oral azithromycin vs. placebo. We will design the placebo with the assistance of a reputable pharmacy/pharmaceutical company, using identical capsules or pills containing azithromycin 2 g or a matching placebo (non-antimicrobial agent) to accomplish masking. All of the participants' obstetric care will be similar for all both arms and consist of the routinely available care at each center.

2.4 Detailed Study Procedures

2.4.1 Community Sensitization

Local health providers will receive sensitization about the study to foster communication and collaboration at the facilities where enrollment will take place. In addition, pregnant women and their families in the enrollment area will receive information about the study during antenatal care (ANC) visits to facilitate recruitment and comprehension during the consenting process. Sites may choose to consent women during these sensitization sessions, prior to labor; however, confirmation of eligibility and consent will be required during the screening process described in **Section 2.4.2 and 2.4.3**.

2.4.2 Screening

Women in labor in defined health facilities (both hospitals and health centers) will be identified by research staff. A brief review of eligibility will be made to determine whether the patient is in labor and does not meet any of the exclusion criteria. If a contraindication to participation in the trial is found, the woman will be excluded from the trial at this point.

2.4.3 Consent

Before a woman participates in the trial, the research staff must obtain her informed consent to voluntarily take part in the study. Consent will be obtained from women ≥18 years of age or minors 14-17 years of age in countries where married or pregnant minors (or their authorized representatives) are legally permitted to give consent. When enrolling minors, we will follow the in-country policies for human research protection and the guidelines approved by the local ethical review committees (ERCs). In the case of pregnant minors, this may require that written consent is obtained from her parents/guardians or husband, with written assent from the minor.

Potential participants will be screened and enrolled in the study upon admission for delivery; therefore, consent will be obtained during labor. Consent should be obtained as early as possible during the intrapartum period and must be obtained prior to the cervical dilation limit approved by local authorities (e.g. either > 6 cm or 10 cm) and/or pushing, as assessed from clinical exam by health facility staff. It is not feasible to wait because the intervention must be given before delivery. Research sites

For peer review only - http://bmjopen.bmj.com/site/about/guidelines.xhtml

may choose to obtain initial consent during ANC; however, in this situation, confirmation of consent will be required during the screening and enrollment process.

If consent is obtained during labor, study staff will make necessary accommodations to ensure that the laboring women can comprehend the information presented during the consent process. Potential participants who present in labor can take as much time as needed to consider participation while in labor and will be able to discuss the study with family/friends if desired before deciding on participation. If the participant cannot read, the form will be read aloud to her by a person unaffiliated with the study. Alternatively, the Research Coordinator or a designate may read the consent, but in the presence of a witness who is unaffiliated with the research study. Potential participants will be given an opportunity to discuss the study procedures and ask questions. Additional details are provided in the study Manual of Procedures.

Fair balance will be maintained while describing the risks and benefits of participation in the study. No undue pressure will be placed on the potential participant to enroll in the trial. It will further be explained that lack of participation will not affect the usual and anticipated standard of care. As the literacy levels will vary and may be a challenge, the consent process will include a verbal review of the consent form.

After the potential participant has read the consent form, but before she signs, the research staff will show her a sample study pill and confirm that she is willing and able to take the study pill as prescribed. Only if she is willing to commit to taking the pill will she be enrolled; otherwise, this will be recorded as a refusal of consent. Following review of the consent, the potential participant (or parent/guardian) will be asked to sign the form. If the potential participant (or parent/guardian) is unable to sign her name, she will be asked to use her thumbprint to indicate written approval. In both cases, the unaffiliated person will also sign the consent form. Both the research staff and the study participant retain signed copies of the form.

An eligible woman may refuse to participate in the trial at the time of recruitment. This will be recorded in the Screening and Recruitment Form. She may also choose to withdraw from the study at any time after enrollment. This will be recorded on the Withdrawal/Termination Form.

All research staff responsible for obtaining consent will be trained and certified in the protection of human subjects and the study-specific consent procedures. A model written informed consent form, developed according to the requirements of the U.S. Office of Research Protections (OHRP), is found in **appendix 2**. Each site may modify the model consent to conform to local standards, but the OHRP required elements must be maintained. The research sites will also be responsible for translating the consent form into the appropriate language(s) for their local context.

Global Network countries with legislation regarding the need to videotape consents will comply with the country regulations; however, this is not part of the consent form requirements. This will not be required by protocol but rather decided by each site so as to comply with local rules and regulations.

2.4.4 Masking

Both the azithromycin and placebo will be procured from the same manufacturer. The packaging will be standardized across sites and will be labeled as: "Azithromycin 2 g or Placebo", with the expiration data and a unique identifier. A certificate of authenticity will likewise be provided.

For peer review only - http://bmjopen.bmj.com/site/about/guidelines.xhtml

Clinical and research staff as well as the women will be masked to treatment status unless there is a serious adverse event potentially related to the treatment modality that requires unmasking for safety reasons. There will be one pharmacist at each site who will monitor randomization, drug supply, and safety. Under the instruction of the DCC, the study pharmacist will be trained and authorized to apply un-masking procedures, if concerns about randomization or participant safety are identified.

2.4.5 Randomization Procedures

Randomization of participants will be carried out to obtain a 1:1 allocation ratio between the treatment and placebo arms. Randomization will be stratified by site. A computer algorithm generated by the data coordinating center (DCC) will create the random assignment to one of the treatment arms based on randomly permuted block design with randomly varied block sizes. The block sizes will be known only by the DCC personnel. Each site will receive a lot of the study drug to be distributed sequentially at the participating health facilities which are randomizing women for the study site.

2.4.6 Monitoring before Discharge

Routine post-delivery care will be provided to participants by their clinical providers who will be masked to the study interventions. Research staff (also masked to study medications) trained in obstetric and perinatal outcomes abstraction will be responsible for collecting research data from participant medical records or directly from participants, as relevant, before discharge. Data needed to determine sepsis will include temperature (fever or hypothermia) essentially, plus one or more of heart rate (tachycardia), blood pressure (hypotension), respiratory rate (tachypnea or distress), clinical exam (jaundice or altered mental status) and urine output (low) and a suspicion of infection by the clinical provider or research team. Maternal and neonatal outcomes will be evaluated in the hospital following the delivery (on an ongoing basis until discharge). All participants will be asked to agree to maternal/infant medical record release to abstract information for outcome assessment as part of the consent form. In addition, participants will be educated about the signs and symptoms of infection and other study outcomes and encouraged to call the research team with any concerns.

2.4.7 Monitoring after Discharge

After discharge, study staff will contact participants at the following timepoints:

- In-person visits will be conducted at 3-day, 7-day, and 42-day postpartum to identify maternal or infant infection, unexpected medical visits, side effects, and other study outcomes. WHO criteria for infections will be used and included in the data collection forms. In the case of suspected infection, research staff will collect specimens (e.g. blood, urine, or pus from wound or drained abscesses), as is feasible, in order to identify individual infectious agents.
- Supplemental phone contacts will be conducted at 14-day and 28-day postpartum to review maternal and neonatal signs of infection using WHO criteria. If signs of infection are identified during the review, participants will be asked to visit a study facility for further assessment. These supplemental contacts will reinforce the participants' ability to self-assess for signs of maternal and neonatal infection and improve identification of infection between the 7-day and 42-day postpartum visits. If phone contact is not feasible, in-person visits may be conducted.

If indicated, records of unscheduled visits to any health facility prior to the routine study visits will be obtained and reviewed to ascertain study outcomes. Treating providers may also be called if clarification is needed. Based on the scheduled visits, study staff will identify any unscheduled visits or readmissions

For peer review only - http://bmjopen.bmj.com/site/about/guidelines.xhtml

that occurred in the interim. Readmissions and related diagnoses identified during follow-up visits will be then be validated through medical record review.

2.4.8 Sequence of Study Activities

The sequence of study activities is described in **Figure 1**. A detailed scheduled of study procedures is found in **Appendix 3**.

Figure 1. Flowchart of Study Activities

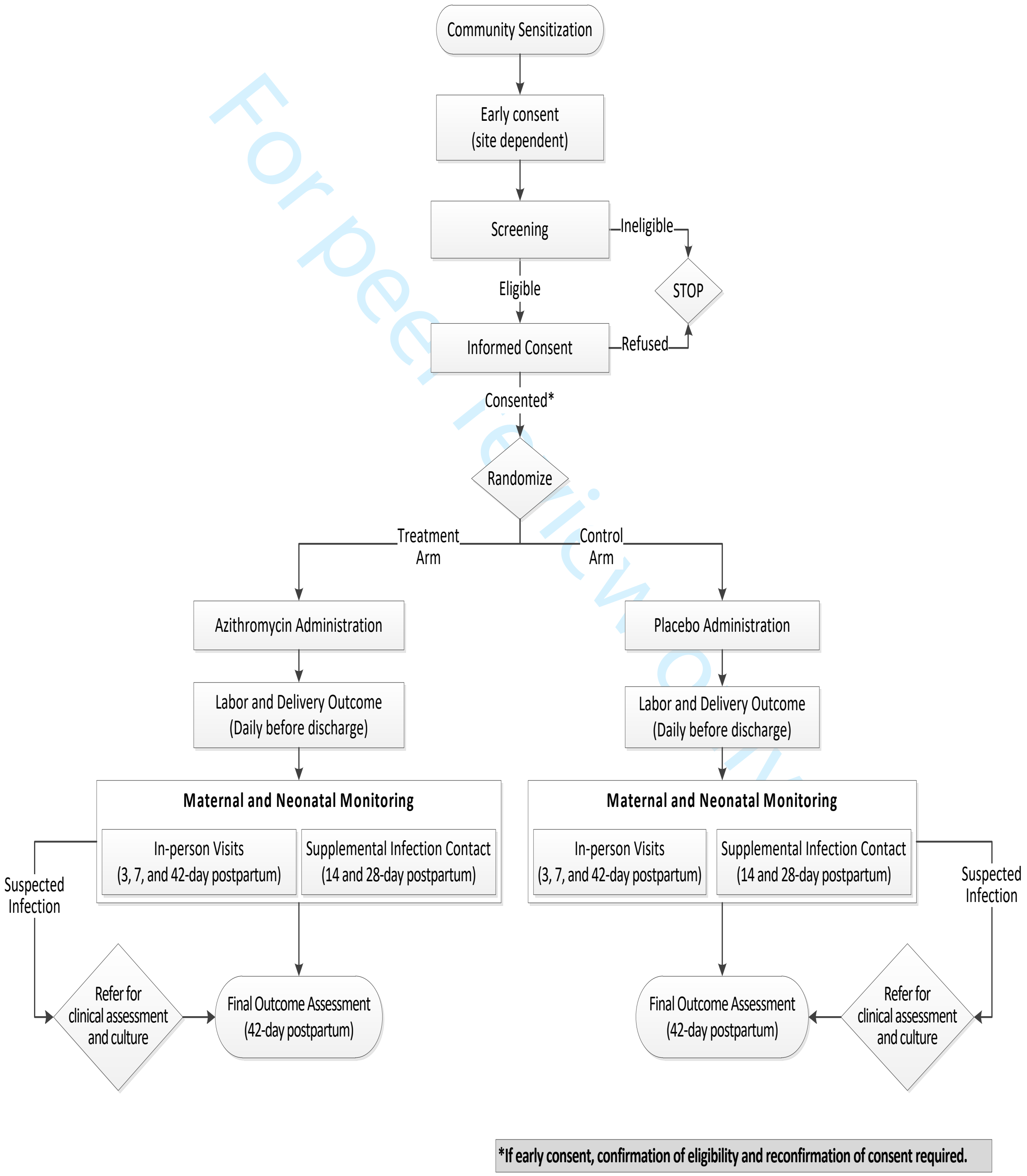

For peer review only - http://bmjopen.bmj.com/site/about/guidelines.xhtml

2.5 Primary Outcomes

The primary outcomes are:

- Maternal: Incidence of maternal death or sepsis within 6 weeks (42 days) post-delivery in intervention vs. placebo group.
- Neonatal: Incidence of intrapartum/neonatal death or sepsis within 4 weeks (28 days) post-delivery in intervention vs. placebo group.

Maternal sepsis is a life-threatening condition defined as organ dysfunction resulting from suspected or confirmed infection that occurs post-randomization during labor or the postpartum period. This WHO definition, endorsed by multiple global health organizations, will be operationalized as suspected or confirmed infection based on the presence of fever (>100.4° F/38° C) or hypothermia (<96.8° F/36° C) plus one or more signs of mild to moderate organ dysfunction including tachycardia (≥120 bpm), low BP (systolic <90 mm Hg), tachypnea (>24 breaths/min), altered mental status/confusion, reduced urinary output (<500 ml over 24 hours), jaundice, or renal failure (>1.2 mg/dl). [15],[40,41,42] Components of peripartum infection which will be considered in making the diagnosis of suspected or confirmed infection include clinical chorioamnionitis, endometritis, wound infections (perineal or Cesarean), abdominal or pelvic abscess, mastitis/breast abscess or infection, pyelonephritis, pneumonia, and other bacterial infection as defined in Section 2.6 below.

Neonatal sepsis is defined as a newborn with proven or possible serious bacterial infection (pSBI) or pneumonia, or meningitis. Possible serious bacterial infection will be determined using WHO criteria of pSBI defined as severe chest in-drawing, fever (temperature ≥ 100.4° F/38.0 °C), hypothermia (temperature <95.9°F/35.5 °C), no movement at all or movement only on stimulation, feeding poorly or not feeding at all, and/or convulsions.[33] Clinical and laboratory signs of infection will also be considered in making the diagnosis of suspected or confirmed infection.

Centralized masked adjudication of key infection outcomes will be implemented by the DCC and investigators during the trial to standardize and enhance the reproducibility of trial results. In addition to the above specified criteria, reported antibiotic treatment and culture status will also be considered as part of the adjudication process in making the diagnoses for both maternal and neonatal sepsis. Local site investigators will review and validate the diagnosis for all participants identified to have the primary outcomes applying pre-specified criteria. These reviews will be conducted masked to treatment group. A second investigator from a different site will review the participant's information and make a final outcome determination. If this does not agree with the determination of the local site investigator, then the adjudicating investigator will discuss with the local investigator to reach a consensus, failing which the study PIs will make the final call. This process will be coordinated by the DCC.

The individual components of these primary outcomes are also of interest and will be analyzed.

2.6 Other Maternal Outcomes

a. Chorioamnionitis: Fever (>100.4°F/38°C) in addition to one or more of the following: fetal tachycardia ≥160 bpm, maternal tachycardia >100 bpm, tender uterus between contractions, or purulent/foul smelling discharge from uterus **prior** to delivery.
b. Endometritis: Fever (>100.4°F/38°C) in addition to one or more of maternal tachycardia >100 bpm, tender uterine fundus, or purulent/foul smelling discharge from uterus **after** delivery.

For peer review only - http://bmjopen.bmj.com/site/about/guidelines.xhtml

c. Other infections: Wound infection refers to purulent infection (superficial or deep infection including necrotizing fasciitis) of a perineal or Cesarean wound with or without fever. In the absence of purulence, a wound infection requires presence of fever (>100.4°F/38°C) and at least one of the following signs of local infection: pain or tenderness, swelling, heat, or redness around the incision/laceration; abdominopelvic abscess is evidence of pus in the abdomen or pelvis noted during open surgery, interventional aspiration or imaging; pneumonia refers to fever (>100.4°F/38°C) and clinical symptoms suggestive of lung infection including cough and/or tachypnea (>24 breaths/min) or radiological confirmation; pyelonephritis refers to fever (>100.4°F/38°C) and one or more of the following: urinalysis/dip suggestive of infection, costovertebral angle tenderness, or confirmatory urine culture; Mastitis/breast abscess or infection refers to fever (>100.4°F/38°C) and one or more of the following: breast pain, swelling, warmth, redness, or purulent drainage.
d. Use of subsequent maternal antibiotic therapy after randomization to 42 days postpartum for any reason.
e. Time from drug administration until initial discharge after delivery (time may vary by site).
f. Maternal readmission within 42 days of delivery.
g. Maternal admission to special care units.
h. Maternal unscheduled visit for care.
i. Maternal GI symptoms including nausea, vomiting, and diarrhea and other reported side effects.
j. Maternal death due to sepsis using the Global Network algorithm for cause of death.

2.7 Other Neonatal Outcomes

a. Other neonatal infections (e.g., eye infection, skin infection, omphalitis, urinary tract infection, respiratory rate ≥60 breaths/minute).
b. Neonatal initial hospital length of stay, defined as time of delivery until initial discharge (time may vary by site).
c. Neonatal readmission within 42 days of delivery.
d. Neonatal admission to special care units.
e. Neonatal unscheduled visit for care.
f. Neonatal death due to sepsis using the Global Network algorithm for causes of death.
g. Pyloric stenosis within 42 days of delivery, defined as clinical suspicion based on severe vomiting leading to death, surgical intervention (pyloromyotomy) as verified from medical records, or radiological confirmation.

2.8 Safety Monitoring

Surveillance of maternal side effects including nausea, vomiting, and diarrhea/loose stools, abdominal pain, vaginitis, and dizziness potentially associated with azithromycin will be conducted during labor and postpartum. For infants, findings suggestive of pyloric stenosis will be assessed during the follow up visits. Maternal and neonatal surveillance will also include assessment of unintended medical visits, maternal deaths, stillbirths, neonatal death within 28 days of birth, and infant death after 28 days of birth. Additional maternal and neonatal risks associated with azithromycin use include anaphylaxis, allergic reactions (rash), liver failure, and arrhythmias. Although rare, these side effects will be monitored and reported as a serious adverse event. All safety outcomes will be reviewed at least twice a year by the Data Monitoring Committee (DMC) appointed by NICHD.

For peer review only - http://bmjopen.bmj.com/site/about/guidelines.xhtml

2.9 Site Preparation

In preparation for study implementation, the site investigators will meet with local health authorities and conduct community sensitization activities to ensure that study procedures are appropriate for the local context and to encourage commitment and engagement at the facility and community level. Site preparation activities will focus on:

- Disseminating study objectives to local health authorities and other stakeholders
- Identifying and hiring study staff;
- Developing site-specific procedures for safety monitoring procedures;
- Exploring locally-acceptable methods to monitor and improve follow-up visit compliance;
- Identifying potential implementation challenges and developing culturally-appropriate solutions;
- Training research staff in the implementation of the study procedures, follow-up and ascertainment of infections.

2.10 Potential Risks and Benefits to Participants

There are several potential direct and indirect benefits of this trial. In developing countries, including those of GN partners, fetal and neonatal deaths due to infections and maternal and neonatal infections are common. If intrapartum azithromycin reduces maternal and fetal/neonatal mortality or infections, many deaths could be reduced in the GN sites as well as worldwide.

Emerging data suggest that intrapartum azithromycin reduces maternal and neonatal infection. It is not known if deaths could be prevented but as infections are one of the most frequent causes of maternal and neonatal deaths, there is a possibility that mortality could be reduced.

An ongoing concern for peripartum and perinatal antibiotic prophylaxis is the selection of resistant organisms including azithromycin-resistant organisms leading to resistant infections, and there is concern that disruption of gut and other flora (microbiome) in women and particularly in neonates may lead adverse events including increased allergic reactions, rash and childhood asthma.[12,18,40] In the trial from Gambia of a single oral dose of azithromycin during labor, higher prevalence of colonization with S. aureus azithromycin resistance observed among women and their babies four weeks after treatment had waned 12 months later and azithromycin did not induce other antibiotic resistance to S. pneumoniae or S. aureus; resistance data from actual infections were not available. There was a 7% vs. 21% prevalence of any bacteria in breast milk in those receiving azithromycin vs. placebo [4-5]. In the US trial of 2013 of adjunctive azithromycin in 2013 women who underwent unscheduled cesarean delivery, culture positive maternal infections (1.4% vs. 3.6%) and infections with at least one resistant bacterial species (1% vs. 2.4%) were significantly less frequent in the azithromycin group. Azithromycin-resistant organisms were identified in only 7 (3 vs. 4) participants. There is a paucity of data to address theoretical concerns that disturbances in the establishment of the indigenous intestinal microbiome caused by antibiotic exposure in early life or cesarean delivery, either directly or through modifications of breast microbiome, may increase risk of immune-mediated and inflammatory conditions such as atopic disorders, inflammatory bowel disease and obesity later in life.[27-30] While the potential for important benefits in both the mother and infant likely outweighs the likelihood and effects of antibiotic resistance and microbiome changes, these should be monitored. In addition to monitoring resistance patterns in isolates from clinical infections, antimicrobial and microbiome surveillance of a subset of enrolled participants are included in an ancillary protocol.

For peer review only - http://bmjopen.bmj.com/site/about/guidelines.xhtml

3 ANALYTICAL PLAN

Baseline demographic characteristics and key clinical measures will be compared between the women in the two treatment arms using contingency table approaches for categorical variables and analysis of variance models' continuous variables.

For summaries of study data, categorical measures will be summarized in tables listing the frequency and the percentage of participants; continuous data will be summarized by presenting mean, standard deviation, median and range; and ordinal data will be summarized by only presenting median and range. P-values presented will be based on two-sided tests unless otherwise specified and generally adjusted for randomization factor of site. For most analyses, the interaction between treatment and site will be assessed and if significant, results will also be presented by site. For continuous outcomes, distributional properties will be evaluated and if required, transformations or non-parametric tests will be employed. Additional details for potential covariate adjustments in secondary analyses or handling violations of analytic method assumptions will be detailed in the statistical analysis plan.

Three key populations are of interest for study analyses:

1. The Intention to Treat (ITT) population will include all women randomized and their infants. Analyses of this cohort will be conducted based on randomized treatment.
2. The High-Risk for Infection (HR) sub-group will include all women in the ITT and their infants meeting criteria for being high risk (i.e., prolonged labor [≥18 hours] and/or rupture of membranes [≥8 hours]) at the time of randomization. Analyses of this cohort will be conducted based on randomized treatment.
3. The As Treated population will include all randomized participants that receive any study drug during the study and their infants. Analyses of this cohort will be conducted based on treatment received.

The final determination of analysis population membership will be via a masked data review prior to final study analyses in order to address any potential anomalous cases that may arise in this large of a study population (e.g., randomization/treatment of a woman who is discharged prior to delivery due to false labor or unresponsiveness to induction).

3.1 Statistical Analysis Plan

3.1.1 Primary Analyses

Incidence of maternal death or sepsis and intrapartum/neonatal death or sepsis will be compared between the two treatment arms using generalized linear models. These generalized linear models will be fit with each binary outcome separately as the outcome measure. Estimates of relative risk and associated 95% confidence intervals will be reported. The model will include terms for treatment and site. As randomization occurs at the pregnancy level and approximately 1-2% of pregnancies are anticipated to be multiple gestations, models for neonatal outcomes will account for correlation among multiples assuming an exchangeable covariance structure. For the two primary outcomes, these analyses will be conducted using the ITT population and the p-values associated with the treatment term will be used to formally test each of the two primary hypotheses at the alpha = 0.05 level.

As secondary analyses of the primary outcomes, assuming an overall treatment effect is observed, the models will be run including region (Africa, Latin America, or Asia) and a treatment by region interaction

For peer review only - http://bmjopen.bmj.com/site/about/guidelines.xhtml

term. If the interaction term has a p<0.1, then effects will be reported by region with treatment effect within region tested at the 0.025 level.

Additional exploratory models will also be run including individually: 1) a treatment by site interaction term, 2) any other antibiotic use during labor (yes or no) and its interaction with treatment, and 3) mode of delivery (cesarean or vaginal) and its interaction with treatment. If the interaction term for any of these models has a p<0.1, then effects will also be reported by the relevant subgroups. These exploratory secondary models will also include any demographic or clinical variables found to differ significantly between the treatment arms in the preliminary analyses described above.

From each final model, estimates of relative risk associated with treatment will be obtained including unadjusted estimates of risk from the primary model as well as estimates of risk adjusted for potential confounders from the secondary analyses.

3.1.2 Secondary Analyses – Women at High Risk for Infection Cohort

The major secondary aim is assessing the two primary outcomes (i.e., incidence of maternal death or sepsis and incidence of intrapartum/neonatal death or sepsis) in the women at high risk for infection cohort. These analyses will also assess if the treatment effect differs between the HR cohort vs. non-high-risk women where non-high-risk women comprise all women and their infants in the ITT population that delivered prior to meeting criteria for high-risk (i.e. they delivered after < 18 hours of labor and < 8 hours post-rupture of membranes relative to time of randomization). Specifically, the model for the primary analysis of both the maternal and neonatal primary outcomes will be run including a treatment by risk status interaction term and excluding any data from individuals that meet high-risk criteria after randomization.

The exclusion of data from individuals meeting high risk criteria between randomization and delivery is intended to provide the most distinct groups regarding the HR risk cohort to non-high-risk women comparison. As this exclusion is based on a post-randomization event, sensitivity analyses will be conducted to examine any treatment group differences in meeting high risk criteria post randomization and if there is a difference, to determine the potential magnitude of impact this difference has on treatment effect. An exploratory analysis will also be completed that assesses if there is a difference in treatment effect between women randomized prior to high-risk classification vs. those randomized after high-risk classification. Finally, an exploratory analysis will be conducted that assesses the definition of high-risk to determine if there is a different cut-off that identifies a group of individuals with a greater treatment effect both in relation to randomization and delivery.

3.1.3 Secondary Analyses – Other Secondary Outcomes

Other maternal and neonatal binary outcomes including: the individual components of the primary outcomes, neonatal deaths due to sepsis, maternal infections (clinical chorioamnionitis, endometritis, wound infections (perineal or subsequent cesarean), pyelonephritis and pneumonia), use of subsequent maternal antibiotic therapy, pyloric stenosis and occurrences of maternal or neonatal readmission or admission into special care unit will be analyzed using the approaches detailed in **Section 3.1.1** for the ITT population and approaches detailed in **Section 3.1.2** for the HR cohort. A similar process with generalized linear models employing an appropriate link function will be used to analyze the outcomes of maternal and neonatal initial hospital length of stay.

For peer review only - http://bmjopen.bmj.com/site/about/guidelines.xhtml

Binary safety outcomes, e.g., nausea, vomiting, and diarrhea, will also be analyzed using the approaches detailed in **Section 3.1.1**. These analyses will be conducted using the As Treated population.

3.2 Sample Size

3.2.1 Sample Size for Primary Outcome

Sample size estimates were generated to evaluate the potential benefits of peripartum prophylactic azithromycin to reduce the risk of adverse maternal and neonatal outcomes in two population cohorts of women in low and low-middle income settings. The first population of interest comprises all women delivering in facilities (overall).

Power calculations for the study in the overall study population were generated for two primary outcome measures, one being the risk of maternal death or sepsis among women in the target population and the other being intrapartum /neonatal death or sepsis in infants delivered by women in the target population. For each of these outcome measures estimates of the required sample size needed to detect a risk reduction of 20%, 25%, and 30% were generated for power of 0.8, 0.85, and 0.9. The risk of sepsis or maternal death was assumed to be 3%. That number is slightly higher than the current risk in the GN population, which is slightly less than 2%. However, we anticipate that with active surveillance rather than passive reporting based on the new WHO definition of maternal sepsis (designed to catch more cases of sepsis), the risk will be at least 3%. For the neonatal outcome, the underlying risk of the combined outcome of intrapartum stillbirth, neonatal death, or sepsis was assumed to be between approximately 8% and 14%. This estimate was based on recent data from the GN indicating that the risk of intrapartum stillbirth is approximately 1.8% and the risk of neonatal death during the first 28 days after delivery is 2.3%; we assumed that the risk of sepsis not resulting in death is approximately 4% to 10%. The resulting required evaluable sample sizes are shown in **Table 1** below.

Table 1. Sample Sizes for the Overall Population, Alpha=0.05

		Evaluable Sample Size per Arm		
Baseline Risk	Risk Reduction	Power=0.80	Power=0.85	Power=0.90
3%	20%	11455	13103	15334
3%	25%	7133	8159	9548
3%	30%	4815	5508	6446
8%	20%	4096	4686	5483
8%	25%	2554	2921	3419
8%	30%	1727	1975	2311
14%	20%	2204	2521	2950
14%	25%	1377	1575	1842
14%	30%	932	1066	1247

The sample sizes shown above assume that the hypothesis test of interest is for the overall population and that the Type 1 error is controlled at the neonatal and maternal hypothesis level via testing each at an alpha = 0.05 level. Interest has been expressed for the overall study for being able to test the neonatal risk separately in south Asia and sub-Saharan Africa. Controlling the Type I error rate at the 0.025 level for each of those two areas within this sub-analysis of neonatal risk will be conducted to avoid multiple comparison concerns. The resulting sample sizes for that comparison are shown in Table 2 below. Note that this sample size would be the size required separately for the African and Asian sites.

For peer review only - http://bmjopen.bmj.com/site/about/guidelines.xhtml

It is planned that each site will enroll approximately equal number of participants. As such, approximately 37.5% of randomized mothers will be from sub-Saharan Africa and 50% will be from Asia. This planned enrollment distribution is approximately equivalent to the rates of in-facility deliveries observed in the GN registry database. Specifically, approximately a third of the facility deliveries are conducted in sub-Saharan Africa and between 45% and 50% of the facility deliveries are conducted in Asia. These estimates are also consistent with the enrollment rates for the ASPIRIN study that is currently being conducted at these sites. Therefore, to get the total study sample size required, the numbers in the table would need to be multiplied by 3 to achieve reasonable power for the African sites.

Table 2. Sample Sizes Within Region, Alpha=0.025

		Evaluable Sample Size per Arm		
Baseline risk	Risk Reduction	Power=0.80	Power=0.85	Power=0.90
8%	20%	4961	5607	6477
8%	25%	3093	3496	4038
8%	30%	2091	2363	2729
14%	20%	2669	3017	3485
14%	25%	1677	1884	2176
14%	30%	1129	1276	1474

Given the above information, we propose a sample size of 34,000 participants for the overall study. For the primary neonatal outcome of interest of intrapartum/neonatal sepsis or death, assuming that the loss to follow-up will be in the 2% to 3% range (consistent with the current ASPIRIN trial and the Global Network Maternal and Newborn Health registry), this sample size will be sufficient to provide 90% power to detect a 25% reduction in neonatal mortality and sepsis in the sub-Saharan African region and will provide 90% power to detect a 20% reduction in Asia assuming the baseline risk is at least 8%. For the primary maternal outcome of maternal death or sepsis, the sample size will provide 90% power to detect a 20% reduction from 3% in the population aggregated across all study sites.

3.2.2 Sample Size for High-Risk Women

The second population of interest comprises the cohort of high-risk population of women delivering in facilities with high risk being defined as term and preterm pregnant women who experience prolonged labor or prolonged membrane rupture. As a major secondary aim, sample size estimates for the high-risk cohort were also obtained to ensure adequate power for this analysis.

The primary objective of the high-risk component of the study is to test the effectiveness of a single oral dose of intrapartum azithromycin prophylaxis compared to placebo (all receive usual care) in reducing the risk of maternal sepsis or death in high-risk laboring women. To estimate the sample size required for this component of the study, we assumed conservatively that the underlying risk of the combined outcome in the target population is 6%. Comparable to the other study estimates of the required sample size needed to detect a risk reduction of 20%, 25%, and 30% were generated for power of 0.8, 0.85, and 0.9. Because the interest for this study is in testing the hypothesis overall rather than by region, the estimates were generated using an alpha of 0.05. The results of these calculations are shown in **Table 3** below.

For peer review only - http://bmjopen.bmj.com/site/about/guidelines.xhtml

Table 3. Sample Sizes for the High-Risk Cohort, Alpha=0.05

Baseline risk	Risk Reduction	Evaluable Sample Size per Arm		
		Power=0.80	Power=0.85	Power=0.90
6%	20%	5568	6369	7453
6%	25%	3470	3969	4644
6%	30%	2344	2681	3138

Because a risk reduction of at least 30% is expected in this population, we propose a sample size for this HR cohort of 5,500 women. That sample size will be sufficient to detect a 30% risk reduction with a power of 0.85 with an assumed 2% to 3% loss to follow-up. Assuming that 20% of the women are at high risk, the overall study sample size of 34,000 should allow for sufficient enrollment into the HR cohort.

If the proportion of "high-risk" women is smaller than anticipated, the target enrollment may be modified. A smaller sample size should still provide sufficient power for plausible potential underlying scenarios. For example, a sample size of approximately 4,000 high risk women will be sufficient to detect a 33% risk reduction with a power of 0.82 assuming a baseline risk of at least 6% as well as a 35% risk reduction with a power of 0.80 assuming a baseline risk of at least 5%.

3.3 Available Population

There are no competing protocols ongoing in the GN. Assuming conservatively that 50% of women in labor (30,000 facility births per year) meet eligibility criteria for entry and are enrolled in the trial, approximately 15,000 women/infant dyad will be enrolled per year.

To reach the enrollment target of 34,000, each study site will aim to recruit an equal number of study participants (n=4,250 per site); however, recruitment will be monitored and if a site does not meet targets, adjustments may be made. No site will be permitted to recruit more than 20% of the overall study sample site.

Based on historic GN data, we anticipate that the target of 5,500 high-risk women will be enrolled during the trial. However, through ongoing monitoring, the DCC will assess the number of women enrolled in the "high-risk" group and may modify target enrollment as needed to ensure at least 5,500 high-risk women are enrolled.

3.4 Projected Recruitment Time

The projected study timeline is 36 months or less. This includes the following:

- 0-6 months: Finalize protocol, forms. Obtain approvals, train staff, and obtain/ship study drug/placebo to sites.
- 7-31 months: Enroll participants (exact period of enrollment may vary by site)
- 32-36 months: Complete follow-up; data cleaning and primary analyses.

3.5 Study Monitoring Plan

3.5.1 Reporting Serious Adverse Events

Serious Adverse events (SAEs) will be monitored continuously for any event that meets the following criteria:

- Results in neonatal/fetal or maternal death;
- Is life-threatening;

For peer review only - http://bmjopen.bmj.com/site/about/guidelines.xhtml

- Requires hospitalization or prolongs existing hospitalization;
- Results in persistent or significant disability or incapacity;
- Suspicion of pyloric stenosis within 42 days postpartum (prolonged vomiting leading to death or surgery)
- Any other serious or unexpected adverse event that the study investigator(s) feels should be reported.

3.5.2 Method and Timing for Reporting Serious Adverse Events

The Senior Foreign Investigator (SFI) must report the following SAEs by emailing or faxing a copy of the appropriate study form to RTI as follows:

Within 48 hours of SFI's notification of the event:

- All deaths (maternal, intrapartum stillbirths, neonatal)
- All SAEs with a definite or suspected/probable relationship to the intervention

Within 7 days of SFI's notification of the event:

- All life-threatening events;
- All SAEs considered to have a probable or possible relationship to the intervention;
- All emailed or faxed forms should also be entered into the DMS and transmitted within 7 days as a back-up to ensure no SAE is missed.

Additional reporting procedures include:

- RTI will forward all SAEs to the US-based Principal Investigator (PI) and NIH for further assessment of relationship to study intervention. The PI and SFI will be responsible for reporting to their respective IRB and other regulatory authorities per their institutional policy.
- RTI will be responsible for reporting SAEs to the DMC bi-annually at a minimum. The frequency of reporting to the DMC may be increased if the reported events or interim data reviews by the DMC indicate that more frequent safety monitoring is needed.
- Any SAE considered unrelated to the intervention is not required to be reported in an expedited manner. These events should be entered into the data management system and transmitted per routine procedures.

3.5.3 Data Monitoring Plan and Stopping Rules

All the Global Network sites will report data to the Global Network Data Coordinating Center, located at RTI International. The data will be used to evaluate protocol adherence and site performance (e.g., recruitment, loss to follow-up, data quality). The DCC will provide standardized progress reports to NICHD and the site investigators on a monthly basis to monitor outcome variables and adverse events.

Oversight of the trial will be handled by two principal groups with different focuses:

1. **Protocol-focused Steering Committee (SC):** The SC is comprised of the Central Study Team from the University of Alabama at Birmingham and the University of Zambia, NICHD, the DCC, and investigators from each of the participating sites (see Azithromycin Investigators on page 8). The Central Study Team, with assistance from NICHD and the DCC, will have primary responsibility for overall study design, development of study materials and procedures, and oversight of study implementation. They will meet via conference call bi-weekly to monitor study progress and ensure

For peer review only - http://bmjopen.bmj.com/site/about/guidelines.xhtml

proper implementation of the trial. The Site Investigators will be responsible for providing guidance on study design, developing site-specific implementation plans, ensuring study staff are properly trained, and providing oversight of the study at the site level. The SC will convene via conference call at least once per quarter and will meet in person twice a year to discuss study design and implementation issues. Members of the Central Study team, NICHD, and RTI will also conduct site visits, as the budget allows, to bolster enthusiasm, provide hands-on training and education to the participating staff, and address site-specific issues, if any.

2. **Data Monitoring Committee (DMC)**: The DMC, a standing group that monitors all NICHD-funded Global Network studies, will be responsible for ensuring safe and ethical treatment of study participants through monitoring of the study. The membership will include, at a minimum, a statistician, obstetrician, pediatrician, and an expert in international health. The DMC designated by NICHD will review the data collected at approximate 6-month intervals throughout the course of the study. The DMC reports, which are prepared by the Data coordinating center, will include information on study enrollment rates and participant progress through the study, participant compliance with protocol-specified treatment regimens, protocol violations, adverse events, and efficacy outcomes. The focus of the DMC review will be on monitoring participant safety and study progress/futility. Data on treatment effectiveness will also be presented to frame the DMC discussions on safety and futility. Additionally, one formal interim analyses of efficacy is planned as detailed in **Section 3.5.4**. The DMC will be charged with monitoring adverse events and side effects from azithromycin. All known associated side effects and specific obstetric, fetal, or neonatal concerns will be considered reportable to the DMC. The study will be reviewed by the DMC bi-annually at a minimum but may be reviewed more frequently if concerns are raised about participant safety or about adequate process of the study.

3.5.4 Interim/Adverse Event Monitoring Plan

The DMC designated by NICHD will complete safety reviews of the data bi-annually at minimum during the intervention phase or as often as they decide. Safety reports will be reviewed internally by the DCC quarterly and the DMC chair will be notified if any potential safety signals are identified to allow for more frequent DMC monitoring if needed. Adverse events will the reported and submitted to the DCC (and IRBs) who will report these cumulative masked data to the DMC in the safety reviews. The DMC recommendations about study continuation will be distributed to the IRBs. The DMC will forward their monitoring activities to the project officer representing the NICHD.

Additionally, one formal interim analysis of efficacy and futility will be conducted during the study. Interim analyses will be conducted in both all-comers and high-risk cohort assessing both the primary maternal and neonatal outcomes use the primary analytic approach detailed in **Section 3.1.1 and 3.1.2**. As the maternal and neonatal hypotheses are both of equal importance as well as the treatment effect within the two cohorts, the DMC will not be able to recommend early termination for efficacy unless significance is observed for both outcomes in all comers and the direction and magnitude of effect in the high-risk cohort is consistent with the effect observed in all comers. Cut-off p-values for testing for efficacy at this interim analysis will be determined based on a correction for multiple comparisons to ensure an overall alpha of 0.05. The futility assessment will be based on an analysis of conditional power. The details of the timing of the interim analysis as well as the approaches for correcting for multiplicity and analyzing conditional power will be determined in collaboration with the DMC prior to initiation of study enrollment. Safety and efficacy of the azithromycin will be reviewed and compared

For peer review only - http://bmjopen.bmj.com/site/about/guidelines.xhtml

with data from the placebo group according to a data center plan. The primary outcome rate will be calculated, and the data center will develop guidelines for interim analysis decision-making (e.g. the O'Brien-Fleming boundary table).

3.5.5 Risks/Benefits

Based on the preliminary data, there are few risks to this intervention especially given the single oral dose. Severe risks include anaphylaxis and allergic reactions (rash) and liver failure which are rare with azithromycin based on the product insert and our long history of use of prophylactic azithromycin at UAB since 2000. In 2013, the FDA issued an advisory regarding concerns about potential for rare life-threatening arrhythmias with azithromycin use particularly among those with preexisting cardiovascular risk. To minimize the risk of life-threatening side effects, those with arrythmia, known history of cardiomyopathy, or a known allergy to azithromycin will be excluded. With single 1-2 gram doses, gastrointestinal symptoms (nausea 5-18%, diarrhea/loose stools 7-14%, abdominal pain 5-7%, vomiting 2-7%, and dyspepsia 1%) as well as vaginitis 2% and dizziness (1%) were the most commonly reported side effects. These side effects will be monitored.

3.6 Quality Control

3.6.1 Training

All study personnel must participate in training on the proper implementation of study procedures and the ethics of conducting research with human subjects before beginning any research activity. The SFI and project coordinator will ensure that all study personnel receive the appropriate training and obtain the required certification. RTI will be responsible for developing a certification test. The SFI and project manager will be responsible for overseeing the certification process.

3.6.2 Study Monitoring

Major monitoring responsibilities of the PI/SFI, assisted by the country coordinator, are (1) confirming proper IRB approval; (2) monitoring delivery of the study intervention; (3) assessing and evaluating quality of study implementation; (4) ensuring compliance with the intervention, including proper randomization; (5) evaluating accuracy, precision, and completeness of data collected, entered, and transmitted, along with the DCC; (6) ensuring that all personnel are fulfilling their obligations; (7) maintaining staff morale and enthusiasm; (8) maintaining communication and handling ad hoc problems; (9) ensuring inter-site consistency; and (10) proposing improvements to monitoring activities.

NICHD and the DCC staff will conduct site visits as needed. These visits will include review of individual participant records, including supporting data, to ensure protection of study participants, compliance with the protocol, and accuracy and completeness of records. The SFI/PI will make study documents (e.g., logbooks, data forms, staff training certificates) and pertinent hospital/clinic records readily available for inspection by the local IRB, site monitors, and the NICHD for confirmation of the study data.

3.6.3 Drug Quality Assurance and Monitoring

The study drug manufacturer will have a Good Manufacturing Practices designation vetted by the FDA and a certificate of authenticity will be provided. Each site will adapt best practice guidelines for drug shipment and storage to the needs and infrastructure of their local environment. Study staff will be trained in on the drug shipment and storage plan to ensure that best practices are maintained at all times. Additionally, participants will receive detailed instruction on proper storage of the study drug at

For peer review only

For peer review only - http://bmjopen.bmj.com/site/about/guidelines.xhtml

home. Drug stability information will be maintained throughout the study. For quality assurance, a sample of pills from each site will be randomly selected and tested for bioavailability at multiple time points during the study period. A sample from each batch will be tested.

3.6.4 Plan for Sustaining Intervention

We will plan to present abstracts to reputable international obstetric meetings (e.g. SMFM, ACOG or FIGO) and manuscripts within 3-6 months of completion of the primary data collection to high impact journals such as the New England Journal of Medicine, JAMA, or the Lancet. If the results are positive, we will facilitate the change in practice at participating sites and also approach the WHO to instigate guideline updates to reflect the study findings.

4 DATA MANAGEMENT PROCEDURES

Data will be collected both prospectively and from existing medical records, using hard copy forms or Android Tablets. Regardless of data capture methodology, all data will be kept confidential. Each participant will be assigned a unique study ID which will be used to identify the participant. Only the screening log will contain the name (which is not transmitted). If hard copy forms are used, they will be retained in a secure location for possible editing or queries at the central data entry site. Data will be entered into computers using the Data Management System (DMS) developed by RTI and the assigned study number. The DMS will also allow site staff to produce project reports and backup the study database. Electronic data will be transferred from each data management computer to a single Research Unit Data Center in each country, creating a complete data repository. At least once a week, data will be transmitted from the Research Unit Data Center to the DCC at RTI, where the central database will be located. The DCC will conduct training on data collection procedures and the DMS system, as needed.

Precision and accuracy of actual data collected will be checked by chart review (random 5%) and internal procedures using the computer program. Monthly audits and incomplete data reports will be performed by a review team consisting of at least the SFI and country coordinator. Data editing and error resolution will be performed monthly. In addition, a sample of participants will be visited to confirm participation, with procedures determined per site. These activities will be shared between the site and the DCC. The timing of data collection is found in the schedule of study procedures (**Appendix 3).**

4.1 Data Forms

The following forms will be used for this study:

Form Name	Purpose	Key Data Elements	Data Source
**Contact and Scheduling Form**	To facilitate participant follow-up by documenting contact information and projected study visit schedule	Contact information, date of enrollment, date of follow-up visits	Participant interview, study calendar
**Screening and Enrollment Form**	To determine eligibility and record consent status	Screening date, review and confirmation of inclusion/exclusion criteria, consent status/date.	Participant interview/report, clinical assessment, provider report, medical records
**Randomization Form**	To confirm eligibility and track randomization	Eligibility confirmation, randomization date/time, drug administration date/time, any problems with drug administration (e.g., vomiting, dropped med, etc).	Study records, participant interview and observation

For peer review only

Form Name	Purpose	Key Data Elements	Data Source
**Maternal Baseline Data Form**	To collect additional maternal information	• Demographic and baseline clinical data: admission date/time, EDD, GA, age, height, weight, pregnancy and medical history, etc.<br>• Events during labor and delivery: timing of onset of labor, prolonged labor, premature rupture of membranes, date/time/type of membrane rupture, date/time/type of delivery, indication for induction (if applicable), vital signs during labor (temp, HR, RR, BP), complications, etc.<br>• Events after delivery: complications, vital signs, etc.<br>• Infection assessment pre- and post-delivery<br>• Antibiotic treatment pre- and post-delivery<br>• Discharge information	Participant interview/report, provider report, medical records
**Neonatal Baseline Data Form**	To collect information about the infant directly after delivery.	• Sex<br>• Birthweight<br>• Delivery outcome (live or stillbirth)<br>• Complications<br>• Infection assessment<br>• Discharge information: date/time, status (discharged, transferred, died)	Participant interview/report, provider report, medical records
**Maternal and Neonatal Follow-up Form**	To collect maternal and infant health status during follow-up visits at 3 days, 7 days and 42 days after delivery.	• Timing follow-up<br>• Maternal and infant status since discharge: general status, symptoms, indication of infection, clinic visits, hospitalizations, antibiotic use<br>• Temperature	In-person participant interview/report and clinical exam
**Supplemental Infection Contact Form**	To document signs of maternal or neonatal infection and provide referrals if indicated.	• Maternal and neonatal infection danger signs.<br>• Referral information.	Participant interview/report by phone
**Maternal and Neonatal Unscheduled Medical Visit**	To collect information about clinical events reported during follow-up visits	Reason for medical visit, details about medical visit	Participant interview/report, provider report, clinical exam, medical records
**Specimen Collection and Result Form**	To track specimen collection when there is indication of maternal or infant infection	Date/time of specimen collection; reason for collection; location of wound/infection; tracking information for shipping/storing specimen; results	Study documentation if collected and/or tested by study; medical records if collected and/or tested by health facility.
**Serious Adverse Events**	To record fatal, life-threatening, or any other serious, unexpected adverse event	Date/time of event, date/time of resolution, nature of adverse event, management of adverse event, attribution to study (yes/no).	Participant interview/report, provider report, medical records
**Final Study Status**	To document final study status	Final study status (e.g., completion, withdrawal, lost-to-follow-up), Date of final study status. If withdrawal, provide reason.	Participant report, medical records
**Protocol Deviation**	To record protocol deviations and corrective actions	Date and nature of deviation/violation, corrective action.	Participant report, study records, medical records
**Outcome Adjudication**	To validate cases of infection using standard study definitions	Primary and secondary outcomes	Study documentation, provider reports/interviews, medical records

For peer review only - http://bmjopen.bmj.com/site/about/guidelines.xhtml

5 REFERENCES

1. World Health Organization. (2015). WHO recommendations for the prevention and treatment of maternal peripartum infections. Retrieved August 22, 2018, from http://www.who.int/reproductivehealth/publications/maternal_perinatal_health/peripartum-infections-guidelines
2. Liu, L., Oza, S., Hogan, D., Perin, J., Rudan, I., Lawn, J. E., . . . Black, R. E. (2015). Global, regional, and national causes of child mortality in 2000-13, with projections to inform post-2015 priorities: an updated systematic review. *The Lancet, 385*(9966), 430-440.
3. Tshefu, A., Lokangaka, A., Ngaima, S., Engmann, C., Esamai, F., Gisore, P., . . . African Neonatal Sepsis Trial (AFRINEST) group. (2015). Simplified antibiotic regimens compared with injectable procaine benzylpenicillin plus gentamicin for treatment of neonates and young infants with clinical signs of possible serious bacterial infection when referral is not possible: a randomised, open-label, equivalence trial. *Lancet, 385*(9979), 1767-1776.
4. Baqui, A. H., Saha, S. K., Ahmed, A. S., Shahidullah, M., Quasem, I., Roth, D. E., . . . Projahnmo Study Group in Bangladesh. (2015). Safety and efficacy of alternative antibiotic regimens compared with 7 day injectable procaine benzylpenicillin and gentamicin for outpatient treatment of neonates and young infants with clinical signs of severe infection when referral is not possible: a randomised, open-label, equivalence trial. *Lancet Global Health, 3*(5), e279-287.
5. Mir, F., Nisar, I., Tikmani, S. S., Baloch, B., Shakoor, S., Jehan, F., . . . Zaidi, A. K. (2017). Simplified antibiotic regimens for treatment of clinical severe infection in the outpatient setting when referral is not possible for young infants in Pakistan (Simplified Antibiotic Therapy Trial [SATT]): a randomised, open-label, equivalence trial. *Lancet Global Health, 5*(2), e177-e185.
6. Zaidi, A. K., Tikmani, S. S., Sultana, S., Baloch, B., Kazi, M., Rehman, H., . . . Cousens, S. (2013). Simplified antibiotic regimens for the management of clinically diagnosed severe infections in newborns and young infants in first-level facilities in Karachi, Pakistan: study design for an outpatient randomized controlled equivalence trial. *Pediatric Infectious Disease Journal, 32 Suppl 1*(Suppl 1), S19-25. https://doi.org/10.1097/INF.0b013e31829ff7aa
7. Gibbs, R. S. (1980). Clinical risk factors for puerperal infection. *Obstetrics and Gynecology, 55*(5 Suppl), 178S-184S.
8. World Health Organization. (2015). WHO Statement on Caesarean Section Rates. Retrieved August 22, 2018, from http://www.who.int/reproductivehealth/publications/maternal_perinatal_health/cs-statement/en/
9. Mackeen, A. D., Packard, R. E., Ota, E., Berghella, V., & Baxter, J. K. (2014). Timing of intravenous prophylactic antibiotics for preventing postpartum infectious morbidity in women undergoing cesarean delivery. *Cochrane Database of Systematic Reviews, 12*(12), CD009516. https://doi.org/10.1002/14651858.CD009516.pub2
10. Smaill, F. M., & Grivell, R. M. (2014). Antibiotic prophylaxis versus no prophylaxis for preventing infection after cesarean section. *Cochrane Database of Systematic Reviews, 10*, CD007482. https://doi.org/10.1002/14651858.CD007482.pub3
11. Gyte, G. M. L., Dou, L. X., & Vazquez, J. C. (2014). Different classes of antibiotics given to women routinely for preventing infection at caesarean section. *Cochrane Database of Systematic Reviews, 11*(11). https://doi.org/10.1002/14651858.CD008726.pub2
12. Tita, A. T. N., Rouse, D. J., Blackwell, S., Saade, G. R., Spong, C. Y., & Andrews, W. W. (2009). Evolving concepts in antibiotic prophylaxis for cesarean delivery: a systematic review. *Obstetrics and Gynecology, 133*(3), 675-682.
13. Tita ATN, S. J., Boggess K, et al. . (2016). Adjunctive azithromycin prophylaxis for cesarean delivery. *New England Journal of Medicine, 375*, 1231-1241.
14. Oluwalana, C., Camara, B., Bottomley, C., Goodier, S., Bojang, A., Kampmann, B., . . . Roca, A. (2017). Azithromycin in labor lowers clinical infections in mothers and newborns: a double-blind trial. *Pediatrics, 139*(2). https://doi.org/10.1542/peds.2016-2281

For peer review only

For peer review only - http://bmjopen.bmj.com/site/about/guidelines.xhtml

15. World Health Organization. (2017). Statement on maternal sepsis. Retrieved Ausust 22, 2018, from http://www.who.int/reproductivehealth/publications/maternal_perinatal_health/maternalsepsis-statement/en/
16. Reinhart K, D. R., Kissoon N, Machado FR, Schachter RD, Finfer S. . (2017). Recognizing sepsis as a global health priority - a WHO resolution. *New England Journal of Medicine, 377*(5), 414-417.
17. van Dillen, J., Zwart, J., Schutte, J., & van Roosmalen, J. (2010). Maternal sepsis: epidemiology, etiology and outcome. *Current Opinion in Infectious Diseases, 23*(3), 249-254. https://doi.org/10.1097/QCO.0b013e328339257c
18. Roca A, O. C., Bojang A, Camara B, Kampmann B, Bailey R, Demba A, Bottomley C, D'Alessandro U. (2016). Oral azithromycin given during labour decreases bacterial carriage in the mothers and their offspring: a double-blind randomized trial. *Clin Microbiol Infect. , 22*(6), e1-9.
19. Subramaniam A, Ye Y, Mbah R, Mbunwe DM, Pekwarake S, Bunwi EY, Fondzeyuf A, Ngong MG, Dionne-Odom J, Harper LM, Jauk VC, Carlo WA, Halle-Ekane G, Szychowski JM, Tih P, Tita AT. Single Dose of Oral Azithromycin With or Without Amoxicillin to Prevent Peripartum Infection in Laboring, High-Risk Women in Cameroon: A Randomized Controlled Trial. Obstet Gynecol. 2021;138(5):703-713. PMID: 34619734.
20. Harper, L. M., Kilgore, M., Szychowski, J. M., Andrews, W. W., & Tita, A. T. N. (2017). Economic evaluation of adjunctive azithromycin prophylaxis for cesarean delivery. *Obstetrics and Gynecology, 130*(2), 328-334. https://doi.org/10.1097/AOG.0000000000002129
21. Andrews, W. W., Hauth, J. C., Cliver, S. P., Savage, K., & Goldenberg, R. L. (2003). Randomized clinical trial of extended spectrum antibiotic prophylaxis with coverage for Ureaplasma urealyticum to reduce post-cesarean delivery endometritis. *Obstetrics and Gynecology, 101*(6), 1183-1189.
22. Tita AT, H. J., Grimes A, Owen J, Stamm AM, Andrews WW. . (2008). Decreasing incidence of post-cesarean endometritis with extended-spectrum antibiotic prophylaxis. *Obstetrics and Gynecology, 111*(1), 51-56.
23. Tita, A. T., Owen, J., Stamm, A., Hauth, J. C., & Andrews, W. W. (2008). Impact of extended-spectrum antibiotic prophylaxis on incidence of post-cesarean surgical wound infection. *American Journal of Obstetrics and Gynecology, 199*(3), 303.
24. Watts, D. H., Eschenbach, D. A., & Kenny, G. E. (1989). Early postpartum endometritis: the role of bacteria, genital mycoplasmas, and Chlamydia trachomatis. *Obstetrics and Gynecology, 73*(1), 52-60.
25. Hoyme, U. B., Kiviat, N., & Eschenbach, D. A. (1986). Microbiology and treatment of late postpartum endometritis. *Obstetrics and Gynecology, 68*(2), 226-232.
26. Emmons, S. L., Krohn, M., Jackson, M., & Eschenbach, D. A. (1988). Development of wound infections among women undergoing cesarean-section. *Obstetrics and Gynecology, 72*(4), 559-564.
27. Roberts, S., Maccato, M., Faro, S., & Pinell, P. (1993). The microbiology of post-cesarean wound morbidity. *Obstetrics and Gynecology, 81*(3), 383-386.
28. Rosene, K., Eschenbach, D. A., Tompkins, L. S., Kenny, G. E., & Watkins, H. (1986). Polymicrobial early postpartum endometritis with facultative and anaerobic bacteria, genital mycoplasmas, and Chlamydia trachomatis: treatment with piperacillin or cefoxitin. *Journal of Infectious Diseases, 153*(6), 1028-1037.
29. Andrews, W. W., Shah, S. R., Goldenberg, R. L., Cliver, S. P., Hauth, J. C., & Cassell, G. H. (1995). Association of post-cesarean delivery endometritis with colonization of the chorioamnion by Ureaplasma urealyticum. *Obstetrics and Gynecology, 85*(4), 509-514. https://doi.org/10.1016/0029-7844(94)00436-H
30. Keski-Nisula, L., Kirkinen, P., Katila, M. L., Ollikainen, M., Suonio, S., & Saarikoski, S. (1997). Amniotic fluid U. urealyticum colonization: significance for maternal peripartal infections at term. *American Journal of Perinatology, 14*(3), 151-156.
31. Yoon, B. H., Romero, R., Park, J. S., Chang, J. W., Kim, Y. A., Kim, J. C., & Kim, K. S. (1998). Microbial invasion of the amniotic cavity with Ureaplasma urealyticum is associated with a robust host response in fetal, amniotic, and maternal compartments. *American Journal of Obstetrics and Gynecology, 179*(5), 1254-1260. https://doi.org/Doi 10.1016/S0002-9378(98)70142-5
32. Ledger, W. J. (2006). Prophylactic antibiotics in obstetrics-gynecology: a current asset, a future liability? *Expert Review of Anti-Infective Therapy, 4*(6), 957-964. https://doi.org/10.1586/14787210.4.6.957
33. World Health Organization. (2015). Guideline: Managing Possible Serious Bacterial Infection in Young Infants When Referral is not Feasible. Retrieved August 22, 2018, from

For peer review only - http://bmjopen.bmj.com/site/about/guidelines.xhtml

http://apps.who.int/iris/bitstream/handle/10665/181426/9789241509268_eng.pdf;jsessionid=F74B437689D1CB960E2544DDF53FE5A6?sequence=1

34. Sutton, A. L., Acosta, E. P., Larson, K. B., Kerstner-Wood, C. D., Tita, A. T., & Biggio, J. R. (2015). Perinatal pharmacokinetics of azithromycin for cesarean prophylaxis. *American Journal of Obstetrics and Gynecology, 212*(6), 812.
35. Eberly, M. E., Eide, M. B., Thompson, J. L., & Nylund, C. M. (2015). Azithromycin in Early Infancy and Pyloric Stenosis. *Pediatrics, 135*(3), 483. https://doi.org/10.1542/peds.2014-2026
36. Ray, W. A., Murray, K. T., Hall, K., Arbogast, P. G., & Stein, C. M. (2012). Azithromycin and the risk of cardiovascular death. *New England Journal of Medicine, 366*(20), 1881-1890.
37. Svanström, H., Pasternak, B., & Hviid, A. (2013). Use of azithromycin and death from cardiovascular causes. *New England Journal of Medicine, 368*(18), 1704-1701. https://doi.org/10.1056/NEJMoa1300799
38. Hoffman, M. K., Goudar, S. S., Kodkany, B. S., Goco, N., Koso-Thomas, M., Miodovnik, M., . . . Derman, R. J. (2017). A description of the methods of the aspirin supplementation for pregnancy indicated risk reduction in nulliparas (ASPIRIN) study. *BMC Pregnancy and Childbirth, 17*(1), 135. https://doi.org/10.1186/s12884-017-1312-x
39. Goldenberg, R. L., Saleem, S., Ali, S., Moore, J. L., Lokangako, A., Tshefu, A., . . . McClure, E. M. (2017). Maternal near miss in low-resource areas. *International Journal of Gynaecology and Obstetrics, 138*(3), 347-355. https://doi.org/10.1002/ijgo.12219
40. Bonet, M., Souza, J. P., Abalos, E., Fawole, B., Knight, M., Kouanda, S., . . . Metin Gulmezoglu, A. (2018). The global maternal sepsis study and awareness campaign (GLOSS): study protocol. *Reprod Health, 15*(1), 16. https://doi.org/10.1186/s12978-017-0437-8
41. Albright, C.M., Has, P., Rouse D.J., Hughes B.L. (2018). Internal Validation of the Sepsis in Obstetrics Score to Identify Risk of Morbidity from Sepsis in Pregnancy. Obstetric Anesthesia Digest, 38 (1). https://doi.org/10.1097/01.aoa.0000529967.01647.fb.
42. Bowyer L., Robinson H.L., Barrett H., Crozier T.M., Giles M., Idel I., Lowe S., Lust K., Marnoch C.A., Morton M.R., et al. SOMANZ guidelines for the investigation and management sepsis in pregnancy. Aust. N. Z. J. Obstet. Gynaecol. 2017;57:540–551. doi: 10.1111/ajo.12646.

For peer review only

For peer review only - http://bmjopen.bmj.com/site/about/guidelines.xhtml

Appendix 1. Description of Participating Global Network Sites

The Global Network for Women's and Children's Health Research (GN) was created as a private-public partnership between the U.S. National Institutes of Health (NIH) and the Bill and Melinda Gates Foundation in response to the alarming rates of morbidity and mortality in women and children and the lack of research expertise and infrastructure in the developing world. Its mission is to expand scientific knowledge, develop research infrastructures, and improve health outcomes by building research partnerships to conduct research on feasible, cost-effective, sustainable interventions to address the major causes of perinatal morbidity and mortality of women and children in the developing world. It is currently funded only by the Eunice Kennedy Shriver National Institute of Child Health and Human Development (NICHD).

The current configuration of the GN is comprised of eight multidisciplinary research sites in seven developing countries (Bangladesh, Democratic Republic of Congo, Guatemala, India, Kenya, Pakistan, and Zambia), each with an established collaboration between an institution in the U.S. and one or more in the developing country. Each site has a U.S.-based senior principal investigator (PI) and a senior foreign investigator (SFI) based in the developing world, who lead a team of in-country research staff.

In 2005, the GN implemented its first multicounty protocol, the First Breath (FB) study, a community-based cluster trial to determine whether education and training in the American Academy of Pediatrics Neonatal Resuscitation Program (NRP) and the Essential Newborn Care Program (ENC) of the World Health Organization (WHO) reduced neonatal mortality (7 days) more than education and training in ENC alone. More than 3,700 birth attendants from 100 GN communities with > 150,000 deliveries were taught the appropriate procedures and provided with the necessary equipment to resuscitate infants at birth. Communities were randomized to ENC plus NRP or continued ENC.

The GN has continued to build on the capacity developed in the FB trial through the implementation of more than 10 additional multicounty protocols to address priority research needs to improve maternal and child health in low-resource settings. Current projects include:

- **<u>The Maternal Newborn Health Registry</u>** is a prospective, population-based study of pregnancies and their outcomes in low-middle income countries (DRC, Guatemala, India, Pakistan, Bangladesh, Zambia and Kenya). All pregnant women in participating clusters are registered and their outcomes tracked for 6 weeks post-delivery. The primary purpose of this prospective, population-based observational study of approximately 60,000 women per year is to quantify and understand the trends in pregnancy services and outcomes over time in defined, low-resource geographic clusters. The goal is to provide population-based statistics on stillbirths, neonatal and maternal mortality as the basis of health care policy. The data from the registry also provide the mortality and morbidity outcomes for Global Network trials and help investigators plan future studies for the Global Network.
- Preterm birth remains the leading cause of neonatal mortality and long-term disability throughout the developed and developing world. A growing body of evidence suggests that 1st

For peer review only - http://bmjopen.bmj.com/site/about/guidelines.xhtml

trimester administration of low dose aspirin can reduce the rate of PTB substantially. The **ASPIRIN Study** is a prospective, randomized, placebo-controlled, double-masked, multi-center clinical trial to examine whether low dose aspirin initiated between 6 0/7 weeks- 12 6/7 weeks gestation reduces the risk of preterm birth. The study has enrolled 11,920 women across seven sites in Africa, Asia, and Latin America.

- Attention is increasingly directed to the role of maternal nutrition during the 1st trimester for normal growth and development during the first thousand days, from conception to the child's second birthday. The primary hypothesis of the **Women First: Preconception Maternal Nutrition study** is that for women in poor communities, a comprehensive maternal nutrition intervention commencing at least 3 months prior to conception and continuing throughout pregnancy, will be associated with a significantly greater newborn length than for offspring whose mothers start to receive the same intervention at 12 weeks gestation or who do not receive the intervention at all. The results of this trial will make a major contribution to refining evidence-based strategies for maternal nutrition supplementation and evaluating the cost-benefits of extending such strategies beyond pregnancy to virtually all women of child-bearing age, including adolescent girls.

For peer review only

For peer review only - http://bmjopen.bmj.com/site/about/guidelines.xhtml

APPENDIX 2. SAMPLE INFORMED CONSENT

Global Network for Women's & Children's Health Research

Single oral dose of azithromycin 2 gm in laboring women to prevent neonatal infection/death and maternal peripartum infection/death

INVESTIGATORS:
[LIST SITE INVESTIGATORS]

SPONSOR:
The Eunice Kennedy Shriver National Institutes of Child Health and Human Development (NICHD)

You are being asked to participate in a research study for pregnant mothers. This study is funded by the U.S. National Institutes of Health and the Bill and Melinda Gates Foundation. This form provides you with information about the study so that you can decide whether you would like to participate. A member of the research team will describe the study to you and answer all of your questions. Please read the information below and ask questions about anything you don't understand before deciding whether or not to take part. You may also request that the research staff read the form to you.

What is the purpose of the study?
The purpose of this study is to learn whether an antibiotic, called azithromycin, given orally (by mouth) to pregnant women during labor can reduce the risk of infection for the woman and her baby.

Who will be in the study?
A total of 34,000 women will be enrolled in this study from eight sites in sub-Saharan Africa, South Asia, and Latin America. If needed, up to 2000 additional women across all sites who meet high-risk criteria will be enrolled (defined as 8 hours or more of membrane rupture before delivery or 18 hours or more of labor). In [insert site name], no more than [insert max sample size] will be enrolled.

You qualify for this study if you are a pregnant woman of the legal age of consent who is in labor with one or more live fetuses and a pregnancy ≥28 weeks, plan to deliver vaginally in a health facility, have no known infections that require antibiotics, have no known problems taking azithromycin or similar antibiotics (such as amoxicillin), and have not used azithromycin, erythromycin, or similar antibiotic in the past 3 days. We will ask you some questions about your pregnancy and health status to make sure you qualify to participate.

What will happen if I join this study?
Before participating, you will be provided with information about the study procedures and given an opportunity to ask questions. If you qualify and agree to participate, you will be asked to sign this form to indicate your consent.

If you agree to participate, you will be assigned to either the treatment group or the control group. The assignment is made randomly, like flipping a coin or choosing a grain of rice from a bag. The women in the treatment group will take four 500 mg azithromycin pills. For comparison, the women in the control group will take four pills that look identical to azithromycin but do not contain any medication. This will

For peer review only - http://bmjopen.bmj.com/site/about/guidelines.xhtml

allow the researchers to compare how well azithromycin works to prevent infection in mothers and their babies. Neither you nor the study staff will know whether you are assigned to the treatment or control group.

After you have been assigned to a treatment group, you will be given 4 small 500 mg pills to take by mouth. A member of the study team will watch you take the pills. After you take the pills, you will receive care during your labor, delivery, and recovery from the health facility staff, according to the local standard of care. It will take approximately 30 minutes to complete the consent form and take the pills.

While you are at the health facility to deliver your baby, the study team will also collect:

- Information about your health status during labor, at the time of delivery, and after delivery. This includes collecting your temperature and vital signs, such as heartrate and blood pressure;
- Information about your baby's health status during and after delivery. This includes collecting your baby's temperature and vital signs, such as heart rate and blood pressure;
- If you or your baby develop an infection before you are discharged from the health facility, a specimen will be collected to help identify the bacteria that is causing the infection. This may be blood sample, a urine sample, or a sample taken from the site of the infection (for example, pus from a wound or breast milk in the case of a breast infection).

It will take no more than 30 minutes to collect health information about you and your baby. In the case of suspected infection, it may take an additional 30 minutes to collect samples from you or your baby.

After delivery, a member of the study team will visit you a total of three times to assess the health status of you and your baby. These visits will be scheduled to take place at your home or a health facility at 3 days, 1 week (7 days) and 6 weeks (42 days) after delivery. If you or your baby are hospitalized at the scheduled time, the study team will visit you in the hospital. During the visit, the study team will assess you and your baby for current or past signs of infection. This will include collecting health information and measuring temperature for you and your baby. Each visit will take approximately 30 minutes.

A member of the study team will also contact you by phone at 14 days and 28 days after delivery to review the signs and symptoms of infection. If you do not have a phone, the study team will arrange to visit you at home or at the study facility. Each phone contact will take approximately 15 minutes.

If a sign of infection is identified in you or your baby during the in-person visits or phone contact, you will be referred a health facility for further assessment. You will also be asked to provide a sample, such as blood, pus, or urine, to help identify the bacteria causing the infection. The samples will be labelled with a study identification number in place of your name or your baby's name. Only dedicated members of our study team will have access to the samples.

To ensure that we have accurate and complete information about the health of you and your baby, we will access and collect information from the medical records at the health facilities where you and your baby have received care. By agreeing to participate in this study, you are also agreeing to give permission for the study staff to access your medical records. We will take precautions to protect the information that is collected from your medical records. Only study staff will have access to this information. To further protect you and your baby, all of your information will be coded with a number in place of your name.

For peer review only - http://bmjopen.bmj.com/site/about/guidelines.xhtml

For peer review only

The local research staff have been selected because of their skills, knowledge, and familiarity with your community. The research staff are here to support you during the study and should be contacted between visits if you have any questions or concerns.

What are the risks and discomforts

Azithromycin is a sometimes used to treat infections in pregnant women and children. Research shows that the risks of taking azithromycin are minimal. A commonly reported side effect is gastric discomfort (nausea, stomach pain, diarrhea, vomiting); however, you will be given pills with a special coating which will help prevent stomach discomfort. There is also a small but rare risk that azithromycin could cause arrythmia (irregular or abnormal heartbeat) or an allergic reaction; therefore, you will not be able to participate if you have a known history of heart problems or have had a bad reaction to azithromycin or a similar drug in the past.

If it is necessary to take a sample because of infection, you or your baby may feel temporary discomfort, but this will only last a few seconds. To minimize this, we will ensure research staff are well trained in the procedure.

Another possible risk of participating in this study is that your name and personal information may be seen by persons who are not part of the project. To prevent this, you will be given an identification number that will be used in place of your name on all study documents.

Information from this research study will be retained by [local institution] and RTI International in the United States (U.S.) and in the future may be included in a de-identified public use database managed by NICHD Data and Specimen Hub (DASH) in compliance with the U.S. National Institutes of Health (NIH) Public Access Policy. De-identified means that you and your baby will not be individually identified by name or other personal identifiers in the database. Your full name or any address details will not be included. Information released will not identify you or your baby's participation in this research study.

What are the benefits of participating?

You will not receive any money from participating in this study, but your participation may provide important information that can be used in the future to prevent infection in mothers and babies. Also, there is preliminary information suggesting that the use of azithromycin in pregnancy can reduce the risk of maternal and infant infection.

If new information about the benefits or risks of azithromycin use in pregnancy becomes available during the study, this information will be given to you by [Insert name of Senior Investigator] or his/her staff.

Will I have to pay for anything?

It will not cost you anything to be in the study.

Is my participation voluntary?

Taking part in this study is voluntary. You have the right to refuse to participate or to withdraw your participation at any time. If you refuse or decide to withdraw, you will not lose any benefits or rights to which you are entitled. These actions will not have any negative effect on the health care you receive from your local health providers. You will still receive your normal medical care.

Can I be removed from this study?

For peer review only - http://bmjopen.bmj.com/site/about/guidelines.xhtml

You will be withdrawn from the study if the research staff thinks that your participation may cause harm to you or your baby. The research staff may also remove you from the study for other reasons at their discretion. Also, the sponsor may stop the study at any time.

What will happen if you are injured by this research?
Although the risk of injury is expected to be very low, all research involves a chance that something bad might happen to you. Despite all safety measures, your participation could result in a reaction or injury. If you or your infant is injured as a result of your participation, you will be provided with emergency care by the study and referred to a doctor for ongoing care, if needed. Ongoing care will not be paid for by the study. [Insert name of Research Institution] and NICHD have not set aside funds to pay you for any such reactions, injuries or related medical care. However, by signing this form, you do not give up any of your legal rights.

What should you do if you have additional questions?
If you have questions about this study or a project-related injury, you should contact [investigator contact]. If you have questions about your or your baby's rights as a project participant, please contact [insert ethics committee contact].

Agreement to be in this study
I have read this paper about the study or it was read to me. I understand the possible risks and benefits of this study. I know that being in this study is voluntary and I choose to be in this study. I understand I will get a copy of this consent form.

Signature (or thumbprint):______________________________________ Date:_______________
(Mother)

Print Name:____
(Mother)

Signature (or thumbprint):______________________________________ Date:_______________
(Parent/Guardian/Husband)

Print Name:____
(Parent/Guardian/Husband)

Future Contact
We may wish to contact you in the future about participating in other related research studies. Please indicate below if you are willing to be contacted.

☐ Yes, I agree to be contacted

☐ No, I do not agree to be contacted

For peer review only - http://bmjopen.bmj.com/site/about/guidelines.xhtml

APPENDIX 3. SCHEDULE OF STUDY PROCEDURES

	ANC Visits	During Labor/ Before Delivery	After Delivery/ Before Discharge	3-day postpartum (pp)	7-day pp	14-day pp	28-day pp	42-day pp	As Needed
**Community Sensitization**	X								
**Screening**									
• *Eligibility confirmation*		X							
• *Clinical assessment*		X							
**Consent**		X							
**Randomization**		X							
**Drug Administration**		X							
**Baseline Data Collection**									
• *Sociodemographic information*		X	X						
• *Medical history*		X	X						
• *Labor and delivery information*			X						
**Monitoring**									
• *Drug side effects*		X	X	X	X			X	X
• *Maternal events during labor/delivery*		X	X						
• *Neonatal events during labor/delivery*			X						
• *Maternal infection/sepsis assessment*			X	X	X			X	X
• *Neonatal infection/sepsis assessment*			X	X	X			X	X
• *Maternal death*			X	X	X			X	X
• *Stillbirth or neonatal death within 28 days of birth*			X	X	X	X	X	X	X
• *Infant mortality after 28 days of birth*								X	X
• *Pyloric stenosis*			X	X	X			X	X
• *Other maternal outcomes*			X	X	X			X	X
• *Other neonatal outcomes*			X	X	X			X	X
• *Unintended medical visits*			X	X	X			X	X
**Serious Adverse Events**			X	X	X			X	X

For peer review only - http://bmjopen.bmj.com/site/about/guidelines.xhtml

Reporting checklist for protocol of a clinical trial.

Based on the SPIRIT guidelines.

Instructions to authors

Complete this checklist by entering the page numbers from your manuscript where readers will find each of the items listed below.

Your article may not currently address all the items on the checklist. Please modify your text to include the missing information. If you are certain that an item does not apply, please write "n/a" and provide a short explanation.

Upload your completed checklist as an extra file when you submit to a journal.

In your methods section, say that you used the SPIRITreporting guidelines, and cite them as:

Chan A-W, Tetzlaff JM, Gøtzsche PC, Altman DG, Mann H, Berlin J, Dickersin K, Hróbjartsson A, Schulz KF, Parulekar WR, Krleža-Jerić K, Laupacis A, Moher D. SPIRIT 2013 Explanation and Elaboration: Guidance for protocols of clinical trials. BMJ. 2013;346:e7586

		Reporting Item	Page Number
**Administrative information**			
Title	#1	Descriptive title identifying the study design, population, interventions, and, if applicable, trial acronym	1, 4
Trial registration	#2a	Trial identifier and registry name. If not yet registered, name of intended registry	2, 14
Trial registration: data set	#2b	All items from the World Health Organization Trial Registration Data Set	N/A (Not registered with WHO)
Protocol version	#3	Date and version identifier	14
Funding	#4	Sources and types of financial, material, and other support	19

For peer review only

Roles and responsibilities: contributorship	#5a	Names, affiliations, and roles of protocol contributors	12-14
Roles and responsibilities: sponsor contact information	#5b	Name and contact information for the trial sponsor	13, 19
Roles and responsibilities: sponsor and funder	#5c	Role of study sponsor and funders, if any, in study design; collection, management, analysis, and interpretation of data; writing of the report; and the decision to submit the report for publication, including whether they will have ultimate authority over any of these activities	9, 12, 15, 19
Roles and responsibilities: committees	#5d	Composition, roles, and responsibilities of the coordinating centre, steering committee, endpoint adjudication committee, data management team, and other individuals or groups overseeing the trial, if applicable (see Item 21a for data monitoring committee)	12-14
**Introduction**			
Background and rationale	#6a	Description of research question and justification for undertaking the trial, including summary of relevant studies (published and unpublished) examining benefits and harms for each intervention	4, 5
Background and rationale: choice of comparators	#6b	Explanation for choice of comparators	4, 5
Objectives	#7	Specific objectives or hypotheses	5
Trial design	#8	Description of trial design including type of trial (eg, parallel group, crossover, factorial, single group), allocation ratio, and framework (eg, superiority, equivalence, non-inferiority, exploratory)	4, 5
**Methods: Participants,**			

For peer review only

**interventions, and outcomes**			
Study setting	#9	Description of study settings (eg, community clinic, academic hospital) and list of countries where data will be collected. Reference to where list of study sites can be obtained	5
Eligibility criteria	#10	Inclusion and exclusion criteria for participants. If applicable, eligibility criteria for study centres and individuals who will perform the interventions (eg, surgeons, psychotherapists)	5
Interventions: description	#11a	Interventions for each group with sufficient detail to allow replication, including how and when they will be administered	4-11
Interventions: modifications	#11b	Criteria for discontinuing or modifying allocated interventions for a given trial participant (eg, drug dose change in response to harms, participant request, or improving / worsening disease)	N/A (single dose intervention)
Interventions: adherance	#11c	Strategies to improve adherence to intervention protocols, and any procedures for monitoring adherence (eg, drug tablet return; laboratory tests)	9-11
Interventions: concomitant care	#11d	Relevant concomitant care and interventions that are permitted or prohibited during the trial	N/A (standard of care)
Outcomes	#12	Primary, secondary, and other outcomes, including the specific measurement variable (eg, systolic blood pressure), analysis metric (eg, change from baseline, final value, time to event), method of aggregation (eg, median, proportion), and time point for each outcome. Explanation of the clinical relevance of chosen efficacy and harm outcomes is strongly recommended	7-9
Participant timeline	#13	Time schedule of enrolment, interventions (including any run-ins and washouts), assessments, and visits for participants. A schematic diagram is highly recommended (see Figure)	8, Figure 1

For peer review only

Sample size	#14	Estimated number of participants needed to achieve study objectives and how it was determined, including clinical and statistical assumptions supporting any sample size calculations	11, 12
Recruitment	#15	Strategies for achieving adequate participant enrolment to reach target sample size	7-9
**Methods: Assignment of interventions (for controlled trials)**			
Allocation: sequence generation	#16a	Method of generating the allocation sequence (eg, computer-generated random numbers), and list of any factors for stratification. To reduce predictability of a random sequence, details of any planned restriction (eg, blocking) should be provided in a separate document that is unavailable to those who enrol participants or assign interventions	8, 9
Allocation concealment mechanism	#16b	Mechanism of implementing the allocation sequence (eg, central telephone; sequentially numbered, opaque, sealed envelopes), describing any steps to conceal the sequence until interventions are assigned	8, 9
Allocation: implementation	#16c	Who will generate the allocation sequence, who will enrol participants, and who will assign participants to interventions	8, 9
Blinding (masking)	#17a	Who will be blinded after assignment to interventions (eg, trial participants, care providers, outcome assessors, data analysts), and how	8
Blinding (masking): emergency unblinding	#17b	If blinded, circumstances under which unblinding is permissible, and procedure for revealing a participant's allocated intervention during the trial	8
**Methods: Data collection, management, and analysis**			

For peer review only

Data collection plan	#18a	Plans for assessment and collection of outcome, baseline, and other trial data, including any related processes to promote data quality (eg, duplicate measurements, training of assessors) and a description of study instruments (eg, questionnaires, laboratory tests) along with their reliability and validity, if known. Reference to where data collection forms can be found, if not in the protocol	6-9
Data collection plan: retention	#18b	Plans to promote participant retention and complete follow-up, including list of any outcome data to be collected for participants who discontinue or deviate from intervention protocols	6-9
Data management	#19	Plans for data entry, coding, security, and storage, including any related processes to promote data quality (eg, double data entry; range checks for data values). Reference to where details of data management procedures can be found, if not in the protocol	9
Statistics: outcomes	#20a	Statistical methods for analysing primary and secondary outcomes. Reference to where other details of the statistical analysis plan can be found, if not in the protocol	9-12
Statistics: additional analyses	#20b	Methods for any additional analyses (eg, subgroup and adjusted analyses)	9-12
Statistics: analysis population and missing data	#20c	Definition of analysis population relating to protocol non-adherence (eg, as randomised analysis), and any statistical methods to handle missing data (eg, multiple imputation)	9-10
**Methods: Monitoring**			
Data monitoring: formal committee	#21a	Composition of data monitoring committee (DMC); summary of its role and reporting structure; statement of whether it is independent from the sponsor and competing interests; and reference to	9, 12, 14

For peer review only

		where further details about its charter can be found, if not in the protocol. Alternatively, an explanation of why a DMC is not needed	
Data monitoring: interim analysis	#21b	Description of any interim analyses and stopping guidelines, including who will have access to these interim results and make the final decision to terminate the trial	12
Harms	#22	Plans for collecting, assessing, reporting, and managing solicited and spontaneously reported adverse events and other unintended effects of trial interventions or trial conduct	9, 14
Auditing	#23	Frequency and procedures for auditing trial conduct, if any, and whether the process will be independent from investigators and the sponsor	N/A (no auditing planned)
**Ethics and dissemination**			
Research ethics approval	#24	Plans for seeking research ethics committee / institutional review board (REC / IRB) approval	8, 14
Protocol amendments	#25	Plans for communicating important protocol modifications (eg, changes to eligibility criteria, outcomes, analyses) to relevant parties (eg, investigators, REC / IRBs, trial participants, trial registries, journals, regulators)	14, 15
Consent or assent	#26a	Who will obtain informed consent or assent from potential trial participants or authorised surrogates, and how (see Item 32)	8
Consent or assent: ancillary studies	#26b	Additional consent provisions for collection and use of participant data and biological specimens in ancillary studies, if applicable	8
Confidentiality	#27	How personal information about potential and enrolled participants will be collected, shared, and maintained in order to protect confidentiality before, during, and after the trial	8, 14

For peer review only

Declaration of interests	#28	Financial and other competing interests for principal investigators for the overall trial and each study site	20
Data access	#29	Statement of who will have access to the final trial dataset, and disclosure of contractual agreements that limit such access for investigators	15
Ancillary and post trial care	#30	Provisions, if any, for ancillary and post-trial care, and for compensation to those who suffer harm from trial participation	N/A
Dissemination policy: trial results	#31a	Plans for investigators and sponsor to communicate trial results to participants, healthcare professionals, the public, and other relevant groups (eg, via publication, reporting in results databases, or other data sharing arrangements), including any publication restrictions	15
Dissemination policy: authorship	#31b	Authorship eligibility guidelines and any intended use of professional writers	N/A
Dissemination policy: reproducible research	#31c	Plans, if any, for granting public access to the full protocol, participant-level dataset, and statistical code	15
**Appendices**			
Informed consent materials	#32	Model consent form and other related documentation given to participants and authorised surrogates	14
Biological specimens	#33	Plans for collection, laboratory evaluation, and storage of biological specimens for genetic or molecular analysis in the current trial and for future use in ancillary studies, if applicable	14

Notes:

- 11d: N/A (standard of care) The SPIRIT Explanation and Elaboration paper is distributed under the terms of the Creative Commons Attribution License CC-BY-NC. This checklist was completed on 19. September 2022 using https://www.goodreports.org/, a tool made by the EQUATOR Network in collaboration with Penelope.ai

For peer review only
